# Supplementary material for: GD2-CAR T cell therapy for H3K27M-mutated diffuse midline gliomas
Source: Nature. 2022 Feb 7;603(7903):934–41. doi: 10.1038/s41586-022-04489-4 (PMC8967714; doi:10.1038/s41586-022-04489-4)
Supplement: Supplementary file 1 — This file contains the Clinical Trial Protocol. [file 41586_2022_4489_MOESM1_ESM.pdf]

nature portfolio

https://doi.org/10.1038/s41586-022-04489-4

Supplementary information

GD2-CAR T cell therapy for H3K27M-mutated diffuse midline gliomas

In the format provided by the authors and unedited

This document contains the following items:

1. Original IRB/FDA approved protocol [127 pages]
2. Amended protocol (Amendment 2) [135 pages]

Phase 1 Clinical Trial of Autologous GD2 Chimeric Antigen Receptor (CAR) T cells (GD2CART) for Diffuse Intrinsic Pontine Gliomas (DIPG) and Spinal Diffuse Midline Glioma (DMG)

DEPARTMENT OF PEDIATRICS- HEMATOLOGY AND ONCOLOGY,

and DEPARTMENT OF NEUROLOGY - NEUROONCOLOGY

STANFORD CANCER INSTITUTE

Principal Investigator:	**Michelle Monje, M.D., Ph.D.**
Lead Associate Investigator:	**Robbie Majzner, M.D.**
IND-holder:	**Crystal L Mackall, MD**

Study Agent:	**Autologous T-Cells transduced with retroviral vector (14g2a-CD8.BB.z.iCasp9) expressing GD2 chimeric antigen receptor ; and chemotherapy**
Protocol Version:	**29 April 2020**

Confidentiality Statement

This document contains confidential information of Stanford University School of Medicine. Do not copy or distribute without written permission of the sponsor.

Agent: GD2.BB.z.iCasp9-chimeric antigen receptor (GD2 CAR) retroviral transduced autologous peripheral blood lymphocytes; following fludarabine and cyclophosphamide

Document History	Notes
Amd 0 Version Date: 23 March 2020	Initial IRB and FDA submission
Amd 0 Version Date: 29 April 2020	Amendment based on FDA reviewer comments. Significant changes include:<br>• Update IND number and name of investigational agent<br>• Add Section 4.2.5 ‘Unacceptable Toxicity’<br>• Add to DLT definition any new Grade 3 neurotoxicity lasting longer than 28 days [Section 5.4.5]<br>• Change expedited safety reporting to FDA [Section 7.5.1] to include any new Grade 3 neurotoxicity lasting longer than 96 hours.<br>• Correct typo in Table 3, Cohort Level -1<br>• Edit/formatting changes to eligibility criteria for clarity. Update correlative sample collection calendar.
Version Date:	

PRINCIPAL INVESTIGATOR'S SIGNATURE PAGE

Michelle Monje, MD, PhD

Title: Associate Professor of Neurology and Neurological Sciences

265 Campus Drive, G3077, MC5461
Stanford, CA 94305-5461
T: 650-721-5750
F: 650-724-5824
mmonje@stanford.edu

Signature ______________________ **Date** ______________

Protocol: GD2CART in DIPG and Spinal DMG

Agent: GD2.BB.z.iCasp9-chimeric antigen receptor (GD2 CAR) retroviral transduced autologous peripheral blood lymphocytes; following fludarabine and cyclophosphamide

SPONSOR DETAILS

**Name of IND Sponsor:**	Crystal L. Mackall, M.D.
Address:	265 Campus Dr. G3141A, MC5456<br>Stanford, California, 94305
Telephone Number:	1-650-725-9670

STANFORD CO-INVESTIGATORS

Liora Schultz, M.D.<br>Clinical Assistant Professor of Pediatrics<br>725 Welch Road<br>Palo Alto, CA 94304<br>T: 650-497-8953<br>F: 650-736-8092<br>lioras@stanford.edu	Kara Davis, D.O.<br>Assistant Professor of Pediatrics<br>265 Campus Dr, G2078<br>Stanford, California, 94305<br>T: 650-724-8073<br>F: 650-736-8092<br>kardavis@stanford.edu
Robbie Majzner, M.D.<br>Instructor of Pediatrics<br>1000 Welch Rd, Suite 300 Palo Alto, CA 94304<br>T: 650-723-5535<br>F: 650-723-5231<br>rmajzner@stanford.edu	Crystal Mackall, M.D.<br>Professor of Pediatrics and Medicine<br>265 Campus Dr, G3141A<br>Stanford, California, 94305<br>T: 650-725-9670<br>F: 650-736-8092<br>cmackall@stanford.edu
Sneha Ramakrishna MD<br>Instructor of Pediatrics<br>1000 Welch Rd.,<br>Suite 300 Palo Alto, CA 94304<br>T: 650-497-8953<br>ramakrs@stanford.edu	Gerald Grant, M.D., FACS<br>Endowed Professor in Pediatric Neurosurgery and Professor of Neurology<br>300 Pasteur Drive, Room R211, MC 5327<br>Stanford, CA 94304<br>T: 650-497-8775<br>Ggrant2@Stanford.edu
Sonia Partap M.D.<br>Clinical Associate Professor, Neurology & Neurological Sciences<br>750 Welch Road, Suite 317<br>Palo Alto, CA 94304<br>T: 650-723-0993<br>spartap@Stanford.edu	Cynthia Campen, M.D.<br>Clinical Associate Professor, Neurology & Neurological Sciences<br>750 Welch Road, Suite 317<br>Palo Alto, CA 94304<br>T: 650-723-0993<br>ccampen@Stanford.edu
Meena Kadapakkam, M.D.<br>725 Welch Rd.<br>Palo Alto, CA 94304<br>T: 650-497-8953<br>mkadapak@stanford.edu	Lindsey Rasmussen, M.D.<br>Clinical Assistant Professor, Pediatrics-Critical Care<br>770 Welch Rd, Ste 435, MC 5876<br>Palo Alto, CA 94304<br>T:920-915-1013<br>lkrasmus@stanford.edu
Paul Fisher, M.D.<br>Professor of Pediatric Neuro-Oncology, Professor of Pediatrics<br>750 Welch Road, Suite 317<br>Palo Alto, CA 94304<br>T. 650-736-0885<br>pfisher@Stanford.edu	Susan Hinicker M.D.<br>Assistant Professor of Radiation Oncology<br>300 Pasteur Dr. Rm A099, MC 5302<br>Stanford, CA 94305<br>T. 650-725-2209<br>shiniker@stanford.edu

Timothy Cornell M.D.<br>Professor of Pediatric Critical Care Medicine<br>770 Welch Rd, Ste 435, MC 5876<br>Palo Alto, CA 94304<br>T: 734-904-5467<br>tcornell@stanford.edu	**STUDY COORDINATORs**<br>Anne Cunniffe Marcy, MSBH, CCRP<br>800 Welch Road, FC329<br>Palo Alto, CA 94304<br>T: 650-721-9994<br>acmarcy@stanford.edu<br><br>Christina Baggott, RN, PhD, PPCNP-BC, CPON<br>800 Welch Road, FC319<br>Palo Alto, CA 94304<br>T: 650-497-7659<br>baggott@stanford.edu
Kristen Yeom, M.D.<br>Associate Professor of Radiology<br>725 Welch Rd, MC 5654<br>Palo Alto, CA 94304<br>T. 721-2388<br>kyeom@Stanford.edu	**BIOSTATISTICIAN**<br>John Tamaresis<br>Room T101F, Redwood Bldg M/C 5405<br>150 Governor's Lane<br>Stanford, CA 94305<br>T: 650-498-6425<br>jtamares@stanford.edu
**Coordinating Center/Participating Sites:**	Single Site

SYNOPSIS

**Protocol Number**	IND# 19801/ e-Protocol # IRB-52934 / Oncore: PEDSCCT6005
**Protocol Title**	Phase 1 Clinical Trial of Autologous GD2 Chimeric Antigen Receptor (CAR) T cells (GD2CART) for Diffuse Intrinsic Pontine Gliomas (DIPG) and Spinal Diffuse Midline Glioma (DMG)
**IND Sponsor**	Crystal L. Mackall, M.D.
**Principal Investigator**	Michelle Monje, M.D., Ph.D.
**Name of Investigational Product**	**GD2CART**: Autologous T-Cells transduced with retroviral vector (14g2a-CD8. BB.z.iCasp9) expressing GD2 chimeric antigen receptor; and chemotherapy
**Clinical Phase**	Phase 1
**Background and Rationale**	**<u>Diffuse Midline Glioma (DMG) and Diffuse Intrinsic Pontine Gliomas (DIPG)</u>**<br><br>Diffuse Midline Gliomas (DMG) harboring the H3 K27M mutation, including diffuse intrinsic pontine glioma (DIPG) are lethal, high-grade pediatric brain tumors that are inoperable and pose significant challenges for treatment. Pediatric DMGs were categorized separately in the 2016 WHO classification of CNS tumors from adult gliomas based on the distinct underlying genetic abnormalities[1]. Pediatric DMG is characterized by K27 M mutations in the histone H3 gene *H3F3A,* or less commonly in the related *HIST1H3B* gene, a diffuse growth pattern, and a midline location (e.g. thalamus, brain stem and spinal cord) [2], [3]. The majority of pediatric DMGs arise in the brainstem (> 90% DIPG)[4].<br><br>Diffuse Intrinsic Pontine Glioma (DIPG) is a devastating, aggressive brain tumor arising in the ventral pons and occurring chiefly during childhood. Though brainstem tumors are infrequent among adults, diffuse gliomas of the brainstem comprise approximately 10-15% of pediatric brain tumors, with half of all pediatric malignant gliomas occurring in the brainstem[5]. With an estimated 200-400 children affected by DIPG annually in the United States, it is the second most common malignant brain tumor of childhood[6]. The prognosis is bleak: in the absence of effective therapies, DIPG is uniformly fatal and is the leading cause of childhood brain tumor-related death. Median age at diagnosis is 6.3 years, with median overall survival of 11.2 months[7]; 90% of children will die from the disease within 2 years of initial diagnosis, with less than 1% surviving after 5 years[8]. Many clinical trials over the past three decades have explored the use of various therapeutic agents for DIPG, employing conventional and high-dose chemotherapies as well as targeted agents. Chemotherapy has been attempted at time points before, during and after radiation therapy. Despite all efforts, no improvement in overall survival has been demonstrated to date[9],[10],[11],[12],[13],[14],[15],[16].<br><br>**<u>CAR Therapies</u>**<br><br>The field of cancer immunotherapy has exploded in recent years in part due to successes created by the application of chimeric antigen receptor (CAR) T cell therapy in B cell malignancies. CARs are non-native receptors that link an antigen-binding domain to cell signaling domain(s). When expressed in T cells, CARs endow MHC-unrestricted antigen specificity. Dramatic clinical responses in acute lymphoblastic leukemia (ALL) and lymphoma have led to U.S. Food and Drug Administration (FDA) approval of Kymriah for ALL and lymphoma [17] and YESCARTA™ for lymphoma[18]. A growing number of clinical trials have recently focused on solid tumors, targeting a variety of surface antigens,

including EGFR806, EGFRt, and EGFRvIII, carcinoembryonic antigen (CEA), human epidermal growth factor receptor 2 (HER2), fibroblast activation protein (FAP), and the disialoganglioside GD2.

GD2 has already been credentialed as an immune target for neuroblastoma. Dinutuximab, an anti-GD2 monoclonal antibody (mAb), improves overall survival among high-risk neuroblastoma patients when administered as part of a multimodal regimen for upfront disease[19]. More recently, the combination of dinutuximab with irinotecan/temozolomide was demonstrated to mediate objective responses in 53% of patients with recurrent neuroblastoma, compared to a 6% response rate in patients receiving irinotecan/temozolomide plus a targeted, non-immune agent (temsirolimus)[20]. Our group recently discovered that GD2 is also highly overexpressed in DIPG, at levels on early passage DIPG cell lines that exceed expression in neuroblastoma[21].

CAR Therapies Targeting GD2+ Pediatric Tumors

For cancers with high mutational burdens, such as melanoma, non-small cell lung cancer and colorectal cancers with microsatellite instability, blockade of PD-1, a T cell inhibitory signal, is often sufficient to unleash naturally acquired antitumor immunity[22];[23];[22]. Current concepts hold that mutated proteins create neoantigens that drive antitumor immune responses after PD-1 blockade, since cancers with low mutational burdens do not typically respond to checkpoint blockade [24-26]. Diffuse intrinsic pontine glioma demonstrates a low mutation burden[27], [28], low PDL1 expression and low numbers of tumor-infiltrating lymphocytes and therefore immunotherapy focused on checkpoint blockade is unlikely to demonstrate significant benefit. CAR T cells, in contrast, can effectively clear tumors with low mutational burden. Furthermore, unlike monoclonal antibodies, CAR T cells readily traffic across the blood:brain barrier. For these reasons, our efforts to develop immunotherapies for DIPG have focused on developing CAR T cells.

To identify potential targets for CAR T-cell immunotherapy in DIPG, we screened cell surface antigens using an antibody array in patient-derived DIPG cultures. Significant overlap between independent patient-derived cultures suggests conservation of a core group of surface markers across DIPG patients. From these common targets, we observed that the disialoganglioside GD2 was expressed at high levels on nearly all cells in each of the patient-derived DIPG cultures screened[21]. Unlike monoclonal antibodies, which do not efficiently cross the blood-brain barrier, activated T-cells can infiltrate the CNS following adoptive transfer[29]. We generated human GD2-targeting CAR T-cells incorporating a 4-1BBz costimulatory domain (GD2-CAR)[30] and observed significant GD2-dependent killing and cytokine generation upon exposure to patient-derived DIPG cultures relative to control CD19-CAR T-cells incorporating 4-1BBz (CD19-CAR)[21].

To evaluate *in vivo* efficacy of GD2CART against DIPG, we prepared orthotopic mouse xenografts of DIPG cultures derived from post-mortem patient tissue. DIPG cultures were transduced with a luciferase-expressing construct to enable longitudinal monitoring of tumor burden. These xenograft models faithfully recapitulate the diffusely infiltrating histology of DIPG[31], [32]. Mice were distributed by tumor burden into equivalent treatment and control groups before receiving 1e7 GD2-CAR or CD19-CAR T-cells by a single intravenous injection 7-8 weeks after establishment of pontine xenografts. Within 40 days post-treatment (DPT), marked reductions in tumor burden were observed across two independent GD2-CAR T-cell treated cohorts of mice bearing SU-DIPG6 xenografts[21]. All GD2-CAR treated animals demonstrated complete tumor clearance by bioluminescence imaging. By contrast, no mice in the CD19-CAR T-cell control groups exhibited significant tumor regression[21]. This striking tumor clearance replicated in a second patient-derived model of H3K27M+ DIPG (SU-DIPG-13FL) and in a third, particularly aggressive patient-derived H3K27M+ DIPG model (SU-DIPG-13P*). A dramatic survival advantage was also observed in these xenografted mice[21].

<table>
<tr><td></td><td>Given these data we propose a single institution, Phase I dose escalation trial of GD2.BB.z-iCasp9-CAR T cells (GD2CART) following cyclophosphamide/fludarabine lymphodepleting therapy in children with H3K27M+ DIPG and spinal H3 K27M-mutant DMG following completion of standard, up front radiotherapy. We propose to conduct the safety assessment in subjects with DIPG of any age, rather than initially targeting adolescents or adults, because pontine disease is rare in adolescents and adults and evaluation of safety in spinal DMGs, which occur more commonly in adolescents and young adults, will not inform safety for pontine DMG, since much of the risk of toxicity relates to the location of the tumor. Furthermore, enrollment of adolescents or adults prior to children is not necessary since first, second and third generation CARs targeting GD2 and incorporating the same scFv have already been tested in several clinical trials and have demonstrated safety and significant clinical activity[33],[34, 35].

Patients are eligible for enrollment when they are at least 6 weeks from completion of standard upfront radiotherapy, regardless of documented evidence of progression, and if all other eligibility criteria are met. Given that manufacturing and completion of release testing takes approximately 14 days, patients will receive the treatment approximately 2-3 months following completion of standard upfront radiotherapy. This eligibility criterion was chosen based of the following considerations: 1) A requirement for documented progression prior to enrollment is not feasible, since radiographic progression often cannot be reliably distinguished from radionecrosis in patients. 2) Post-progression survival is very short (median 2.3 months) and may not be long enough for patients to benefit from the effects of the GD2CART. 3) Median progression-free survival following radiotherapy is 7.0 months (80.8% demonstrating progression within 12 months), therefore the risk of progression beyond 3 months is sufficiently high to justify the risks and morbidity associated with the investigational treatment regimen 4) Preclinical models demonstrate that bulky disease is a risk factor for treatment-related morbidity and mortality due to hydrocephalus[21], therefore enrolling patients prior to documented clinical or radiographic progression will increase the likelihood that the therapy can be rendered safely. The study will evaluate safety of administration, feasibility of manufacturing, identify the recommended phase 2 dose (RP2D) and conduct a preliminary assessment of anti-tumor activity. The CAR vector will incorporate an inducible Caspase 9 that can lead to efficient T cell apoptosis following exposure to AP1903 should toxicity require inactivation of the cell product.</td></tr>
<tr><td>Objectives</td><td>Primary Objectives:
✓ Determine the feasibility of manufacturing autologous T cells transduced with 14g2a-CD8-BBz-iCasp9 retroviral vector expressing GD2 Chimeric Antigen Receptor (GD2CART) for intravenous administration in children and young adults with H3K27M DIPG and spinal H3 K27M-mutant DMG using a retroviral vector and dasatinib in the Miltenyi CliniMACS Prodigy® system.
✓ Assess the safety and identify the MTD and/or recommended phase 2 dose (RP2D) of GD2CART in subjects with H3K27M DIPG administered after cyclophosphamide/fludarabine-based lymphodepletion regimen using the following dose escalation schedule: DL1: 1e6 transduced T cells/kg; DL2: 3e6 transduced T cells/kg; DL3: 10e6 transduced T cells/kg.
✓ Assess the safety of the MTD/RP2D of GD2CART in children and young adults with spinal H3K27M mutant DMG.
Secondary Objectives
✓ In a preliminary manner, assess clinical benefit of GD2CART at the RP2D in children and young adults with H3K27M DIPG and in children and young adults with spinal H3 K27M-mutant DMG.
✓ If unacceptable toxicity (as defined in Section 12.5), occurs that is possibly, probably or likely related to GD2CART, assess the capacity for AP1903, a dimerizing agent, to</td></tr>
</table>

	mediate clearance of the genetically engineered cells and resolve toxicity.<br>Exploratory Analyses:<br>✓ Measure expansion/persistence of adoptively transferred GD2CART in the CSF and blood and correlate this with antitumor effects.<br>✓ Conduct analyses of the manufactured T cell product and blood and CSF post-infusion to identify biomarkers associated with enhanced CAR T cell expansion and/or persistence.<br>✓ Assess whether changes in the level of ctDNA in the cerebrospinal fluid can provide prognostic information and/or information regarding clonal evolution of DIPG or DMG over time.<br>✓ Evaluate whether antigen expression or tumor microenvironment are correlated with response to CAR T cell infusion.
**Primary Endpoint(s)**	The primary endpoints for this study are:<br>- Feasibility defined by the rate of successful manufacture of the GD2CART produced with retroviral vector and dasatinib in the Miltenyi CliniMACS Prodigy® system to satisfy the targeted dose level and meet the required release specifications.<br>- Identify the MTD and/or RP2D in subjects with H3K27M DIPG as evidenced by the number of DLTs in 3 dose cohorts (DL1: 1e6 transduced T cells/kg; DL2: 3e6 transduced T cells/kg; DL3: 10e6 transduced T cells/kg). MTD is defined as the dose below that in which 2/6 subjects experienced DLTs.<br>-Assess safety of GD2CART as evidenced by the incidence and severity of dose limiting toxicities (DLT), adverse events, serious adverse events, laboratory abnormalities, changes in vital signs, and changes in physical examination following infusion of GD2CART graded according to the Common Terminology Criteria for Adverse Events (CTCAE) Version 5.0 in children and young adults with H3K27M DIPG and children and young adults with DMG.
**Secondary and Exploratory Endpoints**	-Measure clinical outcomes such as Overall Survival (OS), also including Post Progression Survival (PPS), Progression Free Survival (PFS), Time to Progression (TTP), and radiographic and clinical response following administration of a lymphodepleting preparative regimen and GD2CART in two groups of subjects: H3K27M DIPG and spinal H3 K27M-mutant DMG.<br>-In the event of unacceptable toxicity possibly, probably or definitely related to GD2CART, assess the efficacy of AP1903 in eliminating the genetically engineered cells and in resolving the toxicity.
**Study Centers**	This is a single site, Stanford University, investigator initiated protocol conducted at Lucile Packard Children's Hospital (LPCH) Stanford.
**Sample Size**	This study has a 3 part analysis to establish sample size:<br>1. Feasibility of manufacture;<br>2. Safety and MTD/RP2D in subjects with H3K27M+ DIPG and safety of MTD/RP2D in subjects with spinal H3 K27M-mutant DMG after administration of GD2CART following a lymphodepletion chemotherapy regimen ; and<br>3. Preliminary assessment of efficacy of GD2CART in children and young adults with H3K27M DIPG or with spinal H3 K27M-mutant DMG following standard radiotherapy.<br>If feasibility of cell production is not met (i.e. 3 of the first 6 subjects' cells at each dose level cannot be produced to meet the established release criteria) further enrollment will be paused

<table>
<tr><td></td><td>pending evaluation of the manufacturing process, and modifications made appropriate to improving feasibility prior to continuing enrollment or a decision will be made to establish the RP2D as the dose level wherein manufacturing was feasible. The study will allow for up to 6 subjects to be replaced due to inability to achieve the target cell product.

Safety will include a determination of MTD/RP2D in treated subjects with DIPG using a standard 3 + 3 dose escalation design at 3 dose levels (DL1: 1e6 transduced T cells/kg; DL2: 3e6 transduced T cells/kg; DL3: 10e6 transduced T cells/kg). A minimum of 4 and a maximum of 18 evaluable subjects may be needed to determine RP2D.

Once RP2D is established, up to a total of 20 evaluable subjects with DIPG (including any evaluable subjects from the dose escalation phase) and 10 evaluable subjects with spinal H3 K27M-mutant DMG will be treated at the RP2D dose to further assess safety and perform a preliminary analysis of clinical activity. In addition, we will allow for 6 inevaluable subjects (subjects enrolled but who cannot receive cells, either due to physical deterioration or withdrawn consent during cell growth).

Thus, a maximum of 54 (18 in dose escalation + 24 in expansion cohorts + 6 replaced for manufacturing feasibility + 6 replaced for inevaluability following enrollment) subjects may be enrolled to determine safety, feasibility, and preliminary efficacy.</td></tr>
<tr><td>Overall Duration of the Study</td><td>During the dose escalation phase accrual rate will be limited by observational periods dictated by the study, and thus a maximum of 1-2 subjects accrued/month is expected. During the expansion phase, at least 2 subjects will be accrued per month. Therefore this study may require up to 2.5 years to complete accrual. The study primary and secondary objectives will be completed in approximately 3.5 years.</td></tr>
<tr><td>Duration of Study per Subject</td><td>Subject’s active participation in this study is expected to be 5 years, with a total of 15 years of long term follow up from the time of cell infusion on this study or on a long term follow up study, according to the U.S. Food and Drug Administration (FDA) Guidance for Industry: Gene Therapy Clinical Trials – Observing Participants for Delayed Adverse Events.</td></tr>
<tr><td>Subject Population</td><td>Subjects ≥ 2 year of age and ≤ 30 years of age, with biopsy documented H3K27M mutant DIPG or spinal DMG enrolled following standard upfront radiation therapy, who meet the eligibility criteria.</td></tr>
<tr><td>Eligibility criteria</td><td>Inclusion Criteria
✓ Disease Status
o <u>Dose escalation phase and DIPG expansion cohort</u>: Tissue diagnosis of H3K27M mutant Diffuse Intrinsic Pontine Glioma (DIPG) with radiographically evident tumor restricted to the brainstem, OR
o <u>Spinal DMG expansion cohort</u>: Tissue diagnosis of H3K27M mutant Diffuse Midline Glioma (DMG) of the spinal cord
✓ Age: Greater than or equal to 2 year of age and less than or equal to 30 years of age
✓ Prior Therapy:
o At least 6 weeks following completion of front line radiation therapy.
o At least 3 weeks post chemotherapy or 5 half-lives, whichever is shorter must have elapsed since any prior systemic therapy, except for systemic</td></tr>
</table>

inhibitory/simulatory immune checkpoint therapy, which requires 5 half-lives..

- ✓ Performance Status: Subjects > 16 years of age: Karnofsky ≥ 60% OR Eastern Cooperative Oncology Group (ECOG) performance status of 0 or 1; Subjects ≤ 16 years of age: Lansky scale ≥ 60% (See section **13.1**, Appendix A)
- ✓ Normal Organ and Marrow Function (supportive care is allowed per institutional standards, i.e. filgrastim, transfusion)
  - a) ANC ≥ 1,000/uL
  - b) Platelet count ≥ 100,000/uL
  - c) Absolute lymphocyte count ≥ 150/uL
  - d) Hemoglobin ≥ 8 g/dL
  - e) Adequate renal, hepatic, pulmonary and cardiac function defined as:
    - o Creatinine within institutional norms for age (i.e. ≤ 2 mg/dL in adults or according to table below in children <18 years) OR creatinine clearance (as estimated by Cockcroft Gault Equation) ≥ 60 mL/min

Age (Years)	Maximum Serum Creatinine (mg/dL)
≤5	0.8
5 < age ≤ 10	1.0
>10-18	1.2
>18	2.0

    - o Serum ALT/AST ≤ 3.0 ULN (grade 1)
    - o Total bilirubin ≤ 1.5 mg/dl, except in subjects with Gilbert's syndrome.
    - o Cardiac ejection fraction ≥ 45%, no evidence of physiologically significant pericardial effusion as determined by an ECHO, and no clinically significant ECG findings
    - o Baseline oxygen saturation > 92% on room air
- ✓ Pregnancy Test

  Females of childbearing potential must have a negative serum or urine pregnancy test (females who have undergone surgical sterilization are not considered to be of childbearing potential).
- ✓ Contraception

  Subjects of child-bearing or child-fathering potential must be willing to practice birth control from the time of enrollment on this study and for four (4) months after receiving the preparative lymphodepletion regimen or for as long as GD2CART are detectable in peripheral blood or CSF.
- ✓ Ability to give informed consent. Pediatric subjects will be included in age appropriate discussion and verbal assent will be obtained for those ≥ 7 years of age, when appropriate.

Exclusion Criteria

- ✓ Tumor involvement of cerebellar vermis or hemispheres (pontocerebellar peduncle involvement is allowed), thalamic lesions, or supratentorial lesions.
- ✓ Clinically significant swallowing dysfunction as judged by clinical assessment.
- ✓ Current systemic corticosteroid therapy

	✓ Prior CAR therapy.<br>✓ Uncontrolled fungal, bacterial, viral, or other infection. Previously diagnosed infection for which the patient continues to receive antimicrobial therapy is permitted if responding to treatment and clinically stable.<br>✓ Ongoing infection with HIV or hepatitis B (HBsAg positive) or hepatitis C virus (anti-HCV positive). A history of hepatitis B or hepatitis C is permitted if the viral load is undetectable per quantitative PCR and/or nucleic acid testing.<br>✓ Clinically significant systemic illness or medical condition (e.g. significant cardiac, pulmonary, hepatic or other organ dysfunction), that in the judgement of the principal investigator is likely to interfere with assessment of safety or efficacy of the investigational regimen and its requirements.<br>✓ In the investigator's judgment, the subject is unlikely to complete all protocol-required study visits or procedures, including follow-up visits, or comply with the study requirements for participation.<br>✓ Known sensitivity or allergy to any agents/reagents used in this study.<br>✓ Primary immunodeficiency or history of autoimmune disease (e.g. Crohns, rheumatoid arthritis, systemic lupus) requiring systemic immunosuppression/systemic disease modifying agents within the last 2 years
**Investigational Product, Dose, and Mode of Administration**	Autologous peripheral blood mononuclear cells (PBMC) will be obtained by leukapheresis and cryopreserved. Cryopreserved PBMC stored from participation in other institutional cell therapy or cell collection studies may be used to generate the cellular product on this study as long as they meet the criteria established in this IND. PBMC will be transported to the manufacturing facility where they will undergo selection, activation, transduction with the retroviral vector, expansion, supplemented with dasatinib and formulated for the manufacture of GD2CART. The product will be cryopreserved and returned to Stanford's Cell Therapy Facility (CTF), from which the product will be distributed to the patient care unit for infusion. A conditioning lymphodepletion chemotherapy regimen with cyclophosphamide and fludarabine will be administered prior to cell infusion.<br>Lymphodepletion prior to GD2CART infusion will occur as follows:<br>• Fludarabine 25 mg/m^2 per day IV for days -4, -3, -2<br>• Cyclophosphamide 500 mg/m^2 per day IV for days -4, -3, -2<br>Autologous GD2CART will be administered intravenously in 3 escalating doses (starting at Dose level 1) on Day 0 in hospitalized subjects with DIPG.<br>▪ Dose Level -1: 3e5 transduced T cells/kg (± 20%)<br>▪ Dose Level 1: 1e6 transduced T cells/kg (± 20%)<br>▪ Dose Level 2: 3e6 transduced T cells/kg (± 20%)<br>▪ Dose Level 3: 10e6 transduced T cells/kg (± 20%)
**Study Design and Methodology**	The dose escalation phase utilizes a 3 + 3 study design and will test safety in subjects with H3K27M DIPG enrolled at least 6 weeks following standard first line radiation therapy. With substantial data supporting safety of GD2 CAR T cells in children and adults and the age of the vast majority of subjects with DIPG, enrollment in this dose escalation portion will allow subjects of any age.<br>Three planned dose levels of GD2CART will be administered in escalating cohorts starting at dose level 1: 1e6 transduced T cells/kg (± 20%). Infusions will be staggered between subjects to evaluate safety; 28 days must elapse after infusion of GD2CART in the first subject in each dose cohort; 14 days must elapse after cell infusion in the second and

	subsequent subjects in each dose cohort; and 28 days must elapse after the last subject in each dose cohort to fully access DLTs. If 2/6 subjects experience DLT at dose level 1, dose -1 will be explored (3e5 transduced T cells/kg (± 20%)). MTD will be defined as the dose level in which <30% (2 out of 6) subjects experience DLTs. The MTD, or highest cell dose studied if MTD is not reached, will be considered the RP2D.<br><br>Once the RP2D is established in subjects with DIPG, enrollment will be expanded to a total of 20 subjects with DIPG to further investigate safety and to obtain preliminary data regarding efficacy. In addition, given the established safety data of GD2-CAR T cell therapy, an initial safety lead in with an expansion cohort of a total of 10 subjects with spinal H3 K27M-mutant DMG will be evaluated. During conduct of the two expansion cohorts (DIPG and DMG), safety boundaries will be used to monitor a DLT rate of 30%; however, given the expected toxicity rate we do not anticipate crossing these boundaries. If the number of DLTs in either expansion cohort exceeds 30%, the MTD/RP2D dose level will be de-escalated to the next lower dose and that dose expansion cohort will complete enrollment with the updated RP2D dose level to further assess safety at this dose level and gather preliminary evidence for efficacy.<br><br>Subjects with DIPG will have an intraventricular catheter (Ommaya catheter) placed following enrollment and prior to T cell infusion to allow monitoring, and treatment if necessary, of increased intracranial pressure (ICP).<br><br>Subjects will receive a conditioning lymphodepletion chemotherapy regimen of fludarabine and cyclophosphamide, followed by intravenous infusion of GD2CART (Day 0). Subjects will be monitored closely as an inpatient or outpatient with close proximity to Stanford Clinic, at the investigators' discretion for at least 28 days post-T cell infusion (or longer if the principal investigator or her designee deems necessary) for safety assessments, according to the following schedule:<br><br>Neurological exam: Daily from D0 to Day 28, with increased frequency as clinically indicated.<br><br>Measurement of ICP in subjects with DIPG via Ommaya Reservoir: baseline D0 (prior to infusion), Day 4, Day 7, Day 11, Day 14, Day 21, Day 28. If evidence of neurotoxicity, including ICANS≥2, increased intracranial pressure or clinical deterioration suspected due to neurologic compromise, the patient will be treated according to the neurotoxicity management algorithm (section **13.2.3.5**) which includes emergent imaging and transfer to the ICU for intensive monitoring.<br><br>Radiographic imaging in subjects with DIPG: MRI at baseline (within 7 days prior to D-4), D7, D14, D21 and D28. If clinical condition post-infusion prevents MRI, a CT will be obtained on those days. Sequences detailed in section **5.1.k.**<br><br>Disease evaluations of subjects with spinal H3 K27M-mutant DMG will be specific to the subject's location of disease and will include MRIs with and without gadolinium.<br><br>Neurologists, neuro-oncologists, neurosurgeons and neurointensivists will be consulted and involved in every case and neurointensive care provided for management of increased intracranial pressure or neurologic compromise. The period of DLT assessment will be 28 days after infusion of GD2CART (Day 0). Subjects will be evaluated closely for all toxicities, antitumor effects and for persistence of CAR expressing T cells in blood and CSF, when feasible.

SCHEMA

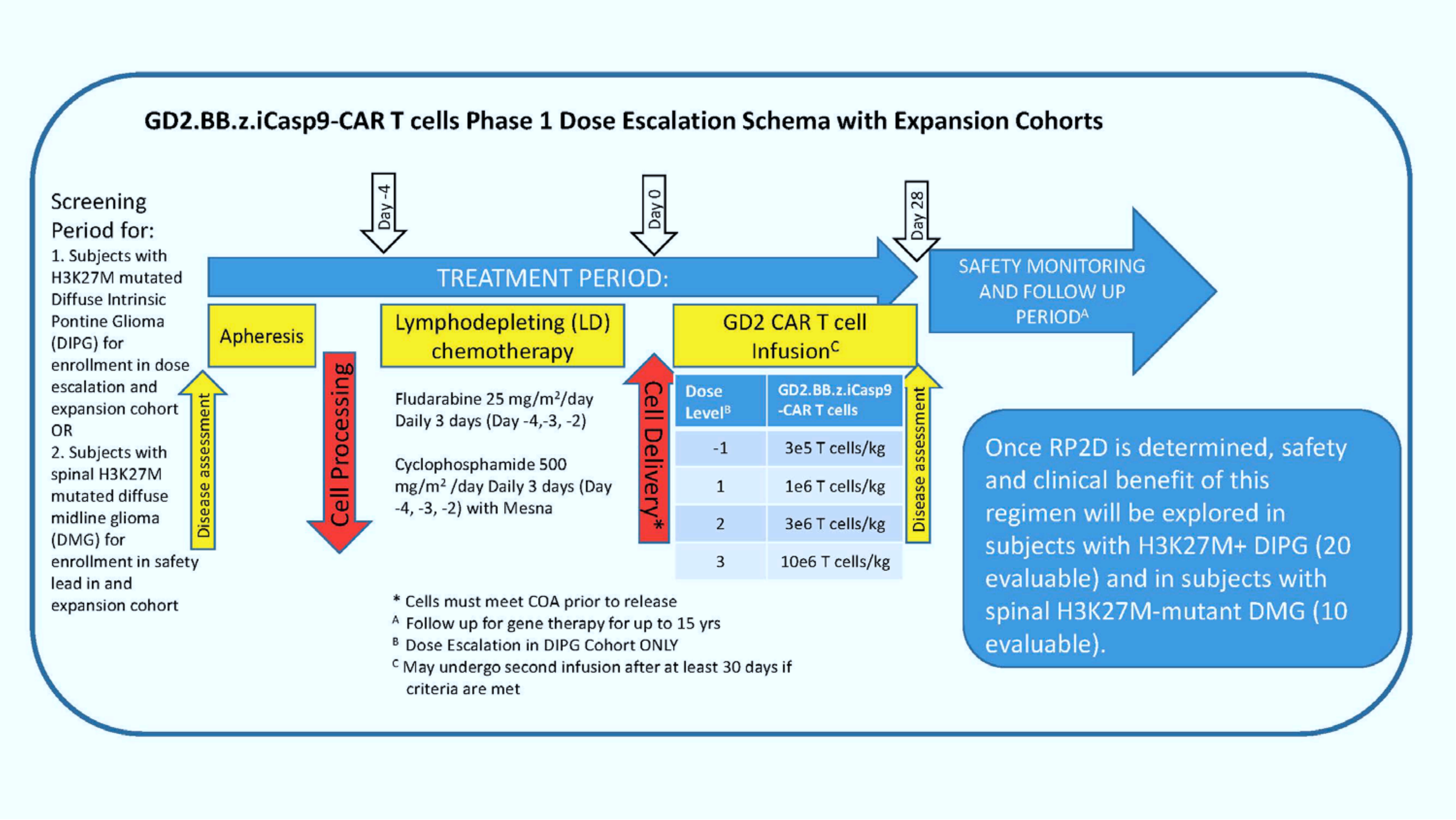
GD2.BB.z.iCasp9-CAR T cells Phase 1 Dose Escalation Schema with Expansion Cohorts
Screening Period for:
1. Subjects with H3K27M mutated Diffuse Intrinsic Pontine Glioma (DIPG) for enrollment in dose escalation and expansion cohort
OR
2. Subjects with spinal H3K27M mutated diffuse midline glioma (DMG) for enrollment in safety lead in and expansion cohort
Day -4
Day 0
Day 28
TREATMENT PERIOD:
SAFETY MONITORING AND FOLLOW UP PERIOD^A
Disease assessment
Apheresis
Cell Processing
Lymphodepleting (LD) chemotherapy
Fludarabine 25 mg/m²/day Daily 3 days (Day -4,-3, -2)
Cyclophosphamide 500 mg/m² /day Daily 3 days (Day -4, -3, -2) with Mesna
Cell Delivery*
GD2 CAR T cell Infusion^C
Dose Level^B
GD2.BB.z.iCasp9 -CAR T cells
-1
3e5 T cells/kg
1
1e6 T cells/kg
2
3e6 T cells/kg
3
10e6 T cells/kg
Disease assessment
Once RP2D is determined, safety and clinical benefit of this regimen will be explored in subjects with H3K27M+ DIPG (20 evaluable) and in subjects with spinal H3K27M-mutant DMG (10 evaluable).
* Cells must meet COA prior to release
A Follow up for gene therapy for up to 15 yrs
B Dose Escalation in DIPG Cohort ONLY
C May undergo second infusion after at least 30 days if criteria are met

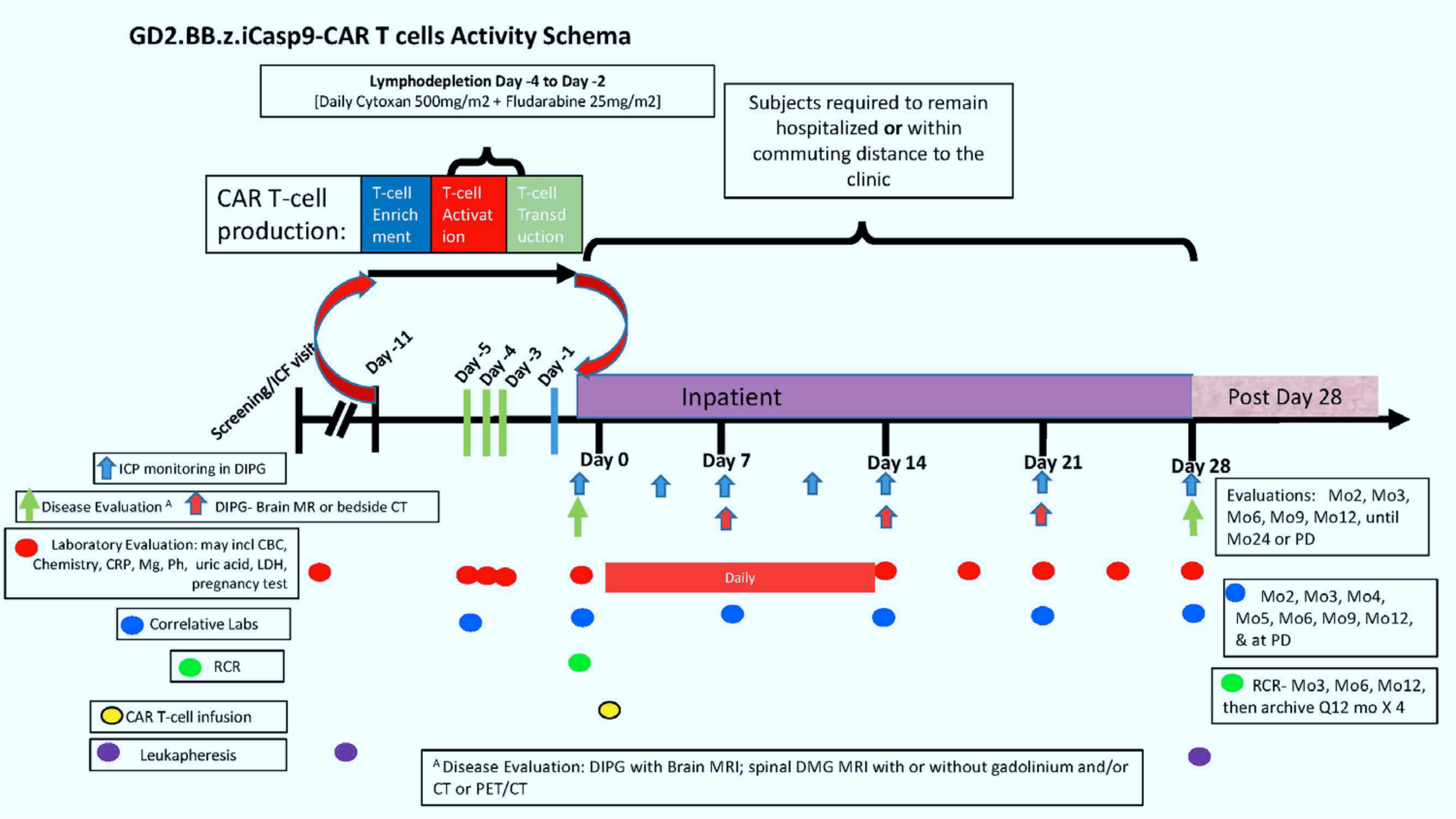
GD2.BB.z.iCasp9-CAR T cells Activity Schema
Lymphodepletion Day -4 to Day -2
[Daily Cytoxan 500mg/m2 + Fludarabine 25mg/m2]
Subjects required to remain hospitalized or within commuting distance to the clinic
CAR T-cell production:
T-cell Enrichment
T-cell Activation
T-cell Transduction
Screening/ICF visit
Day -11
Day -5
Day -4
Day -3
Day -1
Inpatient
Post Day 28
Day 0
Day 7
Day 14
Day 21
Day 28
ICP monitoring in DIPG
Disease Evaluation A
DIPG- Brain MR or bedside CT
Laboratory Evaluation: may incl CBC, Chemistry, CRP, Mg, Ph, uric acid, LDH, pregnancy test
Daily
Correlative Labs
RCR
CAR T-cell infusion
Leukapheresis
Evaluations: Mo2, Mo3, Mo6, Mo9, Mo12, until Mo24 or PD
Mo2, Mo3, Mo4, Mo5, Mo6, Mo9, Mo12, & at PD
RCR- Mo3, Mo6, Mo12, then archive Q12 mo X 4
A Disease Evaluation: DIPG with Brain MRI; spinal DMG MRI with or without gadolinium and/or CT or PET/CT

TABLE OF CONTENTS

PRINCIPAL INVESTIGATOR'S SIGNATURE PAGE 3
SPONSOR DETAILS 4
STANFORD CO-INVESTIGATORS 4
SYNOPSIS 6
SCHEMA 14
TABLE OF CONTENTS 16
TABLE OF FIGURES 19
TABLE OF TABLES 19
LIST OF ABBREVIATIONS AND DEFINITION OF TERMS 20
1 OBJECTIVES 22
1.1 PRIMARY OBJECTIVE 22
1.2 SECONDARY OBJECTIVES 22
1.3 EXPLORATORY ANALYSES: 22
2 BACKGROUND AND RATIONALE 22
2.1 DIFFUSE MIDLINE GLIOMAS (DMG) 22
2.2 DIFFUSE INTRINSIC PONTINE GLIOMAS (DIPG) 23
2.3 GD2 ANTIGEN 23
2.4 CAR THERAPIES 24
2.5 PREVIOUS HUMAN EXPERIENCE WITH GD2-CAR T CELL THERAPIES 30
2.6 SAFETY CONSIDERATIONS FOR GD2 CAR THERAPY 36
2.7 RISK FOR ON-TARGET TOXICITY AND RATIONALE FOR THE INTEGRATED SUICIDE DOMAIN (ICASP9) 41
2.8 CORRELATIVE STUDIES BACKGROUND 42
2.9 STUDY DESIGN 42
2.10 PROTOCOL RATIONALE AND SUMMARY 42
3 PARTICIPANT SELECTION AND ENROLLMENT PROCEDURES 47
3.1 SCREENING PARTICIPANT ELIGIBILITY CHECKLIST 48
3.2 INFORMED CONSENT PROCESS 52
3.3 SUBJECT SCREENING ASSESSMENTS AND REGISTRATION 52
4 TREATMENT PLAN 53
4.1 OVERVIEW 53

4.2 GENERAL CONCOMITANT MEDICATION AND SUPPORTIVE CARE GUIDELINES 55

4.3 CRITERIA FOR REMOVAL FROM PROTOCOL THERAPY AND OFF STUDY CRITERIA 57

5 SUBJECT STUDY PROCEDURES 58

5.1 SUBJECT SCREENING 58

5.2 LEUKAPHERESIS FOR CELL ACQUISITION: 60

5.3 CONDITIONING LYMPHODEPLETION CHEMOTHERAPY REGIMEN 61

5.4 INVESTIGATIONAL AGENT ADMINISTRATION AND RESEARCH PROCEDURES 63

5.5 EVALUATIONS AND FOLLOW UP 71

5.6 OPTION FOR ADDITIONAL DOSE(S) OF GD2CART 77

6 INVESTIGATIONAL AGENT AND COMMERCIAL DRUG INFORMATION...... 79

6.1 GD2.BB.Z.ICASP9-CHIMERIC ANTIGEN RECEPTOR (GD2-CAR) RETROVIRAL TRANSDUCED AUTOLOGOUS PERIPHERAL BLOOD LYMPHOCYTES (GD2CART); FOLLOWING FLUDARABINE AND CYCLOPHOSPHAMIDE 79

6.2 FLUDARABINE 80

6.3 CYCLOPHOSPHAMIDE 81

6.4 ACETAMINOPHEN (TYLENOL): 82

6.5 DIPHENHYDRAMINE (BENADRYL): 82

6.6 ANTIMICROBIAL PROPHYLAXIS 82

6.7 LEVETIRACETAM (KEPPRA): 82

6.8 MESNA 82

6.9 AP1903 DIMERIZING AGENT 83

7 ADVERSE EVENTS AND REPORTING PROCEDURES 84

7.1 POTENTIAL ADVERSE EVENTS 84

7.2 ADVERSE EVENT DEFINITIONS 85

7.3 ADVERSE EVENT REPORTING 87

7.4 IND SPONSOR REPORTING CRITERIA 88

7.5 FDA REPORTING CRITERIA 89

8 CORRELATIVE/SPECIAL STUDIES 91

8.1 SAMPLE COLLECTION SCHEDULE 92

8.2 BLOOD DRAWING LIMITS FOR RESEARCH PURPOSES 92

9 STUDY CALENDAR 94

10 MEASUREMENTS 97

10.1 OUTCOME MEASURES ... 97
10.2 EXPLORATORY OBJECTIVES ... 98
10.3 INSTITUTIONAL REVIEW OF PROTOCOL ... 99
10.4 DATA AND SAFETY MONITORING PLAN ... 99
10.5 DATA MANAGEMENT PLAN ... 100
11 COLLABORATIVE AGREEMENTS ... 100
12 STATISTICAL CONSIDERATIONS ... 100
12.1 STATISTICAL DESIGN ... 100
12.2 PRIMARY OBJECTIVES: ... 101
12.3 SAFETY ENDPOINTS ... 104
12.4 EFFICACY ENDPOINT ... 105
12.5 CAPACITY FOR AP1903 TO MEDIATE CLEARANCE OF GENETICALLY ENGINEERED CELLS AND RESOLVE TOXICITY ... 105
12.6 PROTOCOL STOPPING RULES ... 106
12.7 EXPLORATORY ANALYSIS ... 106
12.8 SAMPLE SIZE ... 107
13 APPENDICES ... 108
13.1 APPENDIX A: APPENDIX A: PERFORMANCE STATUS CRITERIA ... 108
13.2 APPENDIX B: GUIDELINES TOXICITY ASSESSMENT AND MANAGEMENT ... 109
13.3 APPENDIX C: CALCULATION OF WEIGHT FOR CELL DOSE CALCULATION IN MORBIDLY OBESE CANDIDATES ... 117
13.4 APPENDIX D: MONITORING GENE THERAPY TRIALS: REPLICATION COMPETENT RETROVIRUS (RCR) ... 118
13.5 APPENDIX E: DRAFT LETTER AND QUESTIONNAIRE TO SUBJECTS FOR LONG TERM FOLLOW-UP FOR DELAYED ADVERSE EVENTS ... 119
13.6 APPENDIX F: PHYSICIAN (LOCAL MEDICAL PROVIDER) LETTER ... 121
13.7 APPENDIX G: CORRELATIVE SAMPLE SCHEDULE ... 122
14 REFERENCES ... 123

TABLE OF FIGURES

Figure 1: GD2 is an immunotherapy target in DIPG 26

Figure 2: GD2-CAR T cells mediate potent and lasting antitumor response in DIPG orthotopic xenografts 28

Figure 3: GD2-CAR T cell therapy improves survival in DIPG orthotopic xenografts 29

Figure 4: GD2-CAR (OX40.28.z) 30

Figure 5: Effects of Dasatinib on GD2CART 34

Figure 6: GD2 CAR T Culture Expansion on the Prodigy 35

Figure 7: Improved GD2 CAR T Function with addition of dasatinib. 36

TABLE OF TABLES

Table 1: Conditioning Lymphodepletion Chemotherapy Regimen 62

Table 2: GD2CART Product Rapid Release Criteria 64

Table 3: GD2CART Dose Levels 66

Table 4: Dose Escalation Rules 68

LIST OF ABBREVIATIONS AND DEFINITION OF TERMS

ADL	Activities of daily living
AE	Adverse event
BID	Twice daily
BMT-CTF	Stanford Bone Marrow Transplant – Cellular Therapeutics Facility
BSA	Body surface area
CAPD	Cornell Assessment of Pediatric Delirium
CAR	Chimeric Antigen Receptor
CBC	Complete blood count
CI	Confidence interval
CLS	Capillary Leak Syndrome
CMAX	Maximum concentration of drug
CMV	Cytomegalovirus
CNS	Central nervous system
CRF	Case report/Record form
CR	Complete response
CRS	Cytokine release syndrome
CSF	cerebral spinal fluid
CTCAE	Common Terminology Criteria for Adverse Events
DIPG	Diffuse Intrinsic Pontine Glioma
DLCO	Diffusing capacity of the lungs for carbon monoxide
DLT	Dose Limiting Toxicity
DMG	Diffuse Midline Glioma
DMSO	Dimethyl Sulfoxide
DPT	Days post treatment
DSMB	Data Safety Monitoring Board
EBV	Epstein-Barr virus
ECG	Electrocardiogram
EFS	Event free survival
ELISA	enzyme-linked immunosorbent assay
GI	Gastrointestinal
GMP	Good Manufacturing Practices
GvHD	Graft versus Host Disease
HbsAG	Hepatitis B surface antigen
Hgb	Hemoglobin
HIV	Human Immunodeficiency Virus
HLH	Hemophagocytic lymphohistiocytosis
HPF	High-power field
HSV	herpes simplex virus
HSCT	Hematopoietic Stem Cell Transplant
HTN	Hypertensions
HUS	Hemolytic Uremic Syndrome

ICANS	Immune effector Cell-Associated Neurotoxicity Syndrome
ICE	Immune effector Cell-associated Encephalopathy
ICP	Intracranial pressure
IDB	ideal body weight
IRB	Institutional Review Board
IV	Intravenous
LCGM	Stanford's Laboratory for Cell and Gene Medicine
LLN	Lower limit of normal
MAS	macrophage activation syndrome
MRD	Minimal residual disease
MRI	Magnetic resonance imaging
MTD	Maximum tolerated dose
MUGA scan	multigated acquisition scan
NHL	Non-Hodgkin lymphoma
OR	Overall response
OS	Overall survival
PBMC	Peripheral blood mononuclear cells
PCR	polymerase chain reaction
PD	Progressive disease
PFS	Progression free survival
PFTs	pulmonary function tests
PLT	Platelet
PPS	Post progression survival
RP2D	Recommended phase 2 dose
PR	Partial response
QD	Once daily
RECIST	Response evaluation criteria in solid tumors
RR	Response rate
R/R	Relapsed/refractory
SAE	Serious adverse event
SCT	stem cell transplant
SD	Stable disease
TCR	T-cell Receptor
TLS	Tumor lysis syndrome
TTP	Time to progression
ULN	Upper limit of normal
UNK	Unknown
VZV	varicella zoster virus
WBC	White blood cell
WHO	World Health Organization

1 OBJECTIVES

1.1 PRIMARY OBJECTIVE

1. Determine the feasibility of manufacturing autologous T cells transduced with 14g2a-CD8-BBz-iCasp9 retroviral vector expressing GD2 Chimeric Antigen Receptor (GD2CART) for administration in subjects with H3K27M+ diffuse intrinsic pontine glioma (DIPG) or subjects with spinal H3 K27M-mutant diffuse midline glioma (DMG) using a retroviral vector and dasatinib in the Miltenyi CliniMACS Prodigy® system.
2. Assess the safety and identify the maximum tolerated dose (MTD) and/or recommended phase 2 dose (RP2D) of GD2CART in subjects with H3K27M+ DIPG administered after cyclophosphamide/fludarabine-based lymphodepletion regimen using the following dose escalation schedule: DL1: 1e6 transduced T cells/kg; DL2: 3e6 transduced T cells/kg; DL3: 10e6 transduced T cells/kg.
3. Assess the safety of the MTD/RP2D of GD2CART in subjects with spinal H3K27M mutant DMG.

1.2 SECONDARY OBJECTIVES

1. In a preliminary manner, assess clinical benefit of GD2CART at the RP2D in subjects with H3K27M DIPG or spinal H3 K27M-mutant DMG.
2. If unacceptable toxicity (as defined in section 12.5) occurs that is possibly, probably or likely related to GD2CART, assess the capacity for AP1903, a dimerizing agent, to mediate clearance of the genetically engineered cells and resolve toxicity.

1.3 EXPLORATORY ANALYSES:

1. Measure expansion/persistence/phenotype of adoptively transferred GD2CART in the CSF and blood and correlate this with antitumor effects.
2. Conduct analyses of the manufactured T cell product and blood and CSF post-infusion to identify biomarkers associated with enhanced CAR T cell expansion, persistence and/or phenotype.
3. Assess whether changes in the level of ctDNA in the cerebrospinal fluid can provide prognostic information and/or information regarding clonal evolution of DIPG over time.
4. Evaluate whether antigen expression or tumor microenvironment are correlated with response to CAR T cell.

2 BACKGROUND AND RATIONALE

2.1 DIFFUSE MIDLINE GLIOMAS (DMG)

The 2016 World Health Organization Classification of Tumors of the Central Nervous System combined histopathological and molecular features to standardize the diagnosis and nomenclature of CNS tumors[1]. Previously pediatric diffuse gliomas were grouped with their adult counterparts despite known differences. Understanding the distinct underlying genetic abnormalities has aided this separation of entities. One defined group of tumors primarily occurring in children, is

characterized by K27M mutations, a diffuse growth pattern and a midline location, such as the thalamus, brain stem and spinal cord[1]. Over 70% of patients with DMG harbor the H3K27M mutation, which correlates with a poorer outcome[36]. Two types of DMG with the H3K27M mutation will be the focus of this phase 1 clinical trial, spinal DMG and DIPG.

2.2 Diffuse Intrinsic Pontine Gliomas (DIPG)

Diffuse Intrinsic Pontine Glioma (DIPG) is a devastating, aggressive brain tumor of childhood arising in the ventral pons. Though brainstem tumors are rare among adults, they comprise approximately 10-15% of pediatric brain tumors, with half of all pediatric malignant gliomas occurring in the brainstem[5]. DIPG is the most common tumor subtype in this anatomical region, constituting 80% of brainstem gliomas[37]. With an estimated 200-400 children affected by DIPG annually in the United States, it is the second most common malignant brain tumor of childhood[6, 38]. The prognosis is bleak: in the absence of effective therapies, DIPG is uniformly fatal and is the leading cause of childhood brain tumor death. Median age at diagnosis is 6.3 years, with median overall survival of 11.2 months[7]; 90% of children will die from the disease within 2 years of initial diagnosis, with less than 1% surviving after 5 years[8].

Because DIPG grows diffusely and infiltrates critical brainstem structures, surgical resection is not possible. Radiation therapy has remained the mainstay of treatment for the past three decades. At most treatment centers, the standard recommendation is conventionally fractionated local field radiotherapy with dose range of 54-60 Gy for a period of 6 weeks[39]. Radiotherapy provides temporary improvement or stabilization of symptoms and extends overall survival by an average of 3 months; median survival is less than 5 months without radiation[40]. Though both clinical and radiographic responses are initially observed, local recurrence invariably occurs.

Many clinical trials of the past three decades have explored the use of various therapeutic agents for DIPG, employing conventional and high-dose chemotherapies as well as targeted agents. Chemotherapy has been attempted at time points before, during and after radiation therapy. Despite all efforts, no improvement in overall survival has been demonstrated to date[9],[10],[11],[12],[13],[14],[15],[16].

2.3 GD2 Antigen

Tumor targets sought in the development of novel immune therapies require that the antigen is not widely expressed on normal human cells, or differential levels of surface expression on tumors versus normal tissue, thus enabling a therapeutic window. Surface disialoganglioside GD2 is one such target, and is overexpressed on the surface of a variety of cancer tumors including neuroectodermal or epithelial cells, including melanoma, neuroblastoma and osteosarcoma compared to normal tissue. Data generated in Dr. Monje's laboratory at Stanford shows very high overexpression of GD2 in virtually all samples of H3 K27M mutant gliomas[21].

GD2 has already been credentialed as an immune target for neuroblastoma. Dinutuximab, an anti-GD2 mAb improves overall survival among high risk patients as part of multimodal regimen for therapy of upfront disease[19]. More recently, the combination of dinutuximab with irinotecan/temozolomide was demonstrated to mediate objective responses in 53% of patients with recurrent neuroblastoma, compared to a 6% response rate in patients receiving irinotecan/temozolomide plus a target, non-immune agent (temsirolimus)[20]. GD2 targeted therapy has not yet been tested in DIPG. Anti-GD2 antibodies are not good candidates however,

due to limited trafficking across the blood:brain barrier. In contrast, because CAR T cells demonstrate robust trafficking across the blood:brain barrier, we have focused our efforts in developing a CAR targeting GD2 for treatment of DIPG.

2.3.1 GD2 as a target in DIPG and *in vitro* data

To identify potential targets for CAR T-cell immunotherapy in DIPG, we screened cell surface antigens using an antibody array in patient-derived DIPG cultures (**Figure 1a**). Significant overlap between independent patient-derived cultures (**Figure 1b**), suggests conservation of a core group of surface markers across DIPG patients. From these common targets, we observed that the disialoganglioside GD2 was expressed at high levels on each of twelve patient-derived DIPG cultures screened (**Figure 1a**)[21], but not on two samples of H3 wild type glioma. Hit validation by flow cytometry in six H3K27M+ DIPG cultures confirmed uniform, high GD2 expression in all H3K27M+ DIPG cultures examined, including those with the H3F3A K27M mutation (SU-DIPG-6, 13, 17, 25, 29) and the less-common HIST1H3B K27M mutation (SU-DIPG-21)[3],[2],[41] (**Figure 1c**). GD2 expression was far lower in two histone-3 WT pediatric high-grade gliomas (pHGG), including a case of H3WT DIPG (**Figure 1c**). Double immunostaining of primary human DIPG tissue for H3K27M to identify infiltrating malignant cells and GD2 confirmed local expression of GD2 in the native tumor context (**Figure 1d**). Importantly, GD2 expression on DIPG cells was found to be higher than any previous malignancy screened, including neuroblastoma for which anti-GD2 antibodies are part of the standard of care (data not shown) Double immunostaining of primary human DIPG tissue for H3K27M to identify infiltrating malignant cells and GD2 confirmed significant overexpression of GD2 in the native tumor context compared to non-malignant neural tissue (**Figure 1d**).

2.4 CAR THERAPIES

The field of cancer immunotherapy has exploded in recent years with the successes created by the application of chimeric antigen receptor (CAR) T cell therapy in cancers, primarily hematologic malignancies. CARs are non-native receptors that link an antigen-binding domain to cell signaling domain(s). When expressed in T cells, CARs endow MHC-unrestricted antigen specificity. Dramatic clinical responses observed in acute lymphoblastic leukemia (ALL) and lymphoma have led to U.S. Food and Drug Administration (FDA) approval of Kymriah™[17] for ALL and lymphoma and YESCARTA™[18] for lymphoma. A growing number of clinical trials have recently focused on solid tumors, targeting a variety of surface antigens, including EGFR806, EGFRt, and EGFRvIII, carcinoembryonic antigen (CEA), human epidermal growth factor receptor 2 (HER2), fibroblast activation protein (FAP), and the diganglioside GD2.

2.4.1 GD2-41BBζ CAR T cells are active against H3K27M DIPG

2.4.1.1 GD2 CAR T cells mediate potent anti-tumor activity *in vitro*

We generated human GD2-targeting CAR T-cells incorporating a 4-1BBz costimulatory domain (GD2-CAR)[30] (**Figure 1e**) and observed significant GD2-dependent killing (**Figure 1f**) and cytokine generation (**Figure 1g**) upon exposure to patient-derived DIPG cultures relative to control CD19-CAR T-cells incorporating 4-1BBz (CD19-CAR)[21]. Notably, GD2-CAR T-cells do not produce significant cytokines or induce cell killing when exposed to the H3WT, GD2-negative VUMC-DIPG10 patient-derived DIPG culture, providing evidence of therapeutic specificity of

GD2-CAR T-cells toward H3K27M DIPG. To further confirm the targeting specificity of GD2-CAR T-cells, we used CRISPR-Cas9-mediated deletion of GD2 synthase (B4GALNT1) in patient-derived DIPG cells to generate GD2 knockout DIPG cells. Loss of GD2 antigen expression eliminated cytokine production by the GD2-CAR T-cells in comparison to untreated or DIPG cells electroporated with a control guide sequence targeting the AAVS1 locus (**Figure 1h**).

This CAR is delivered via a retroviral vector that was generated by cloning the 14g2a scFv into a CAR with a CD8a transmembrane and hinge region, 4-1BB costimultory domain, and CD3ζ.

Figure 1: GD2 is an immunotherapy target in DIPG

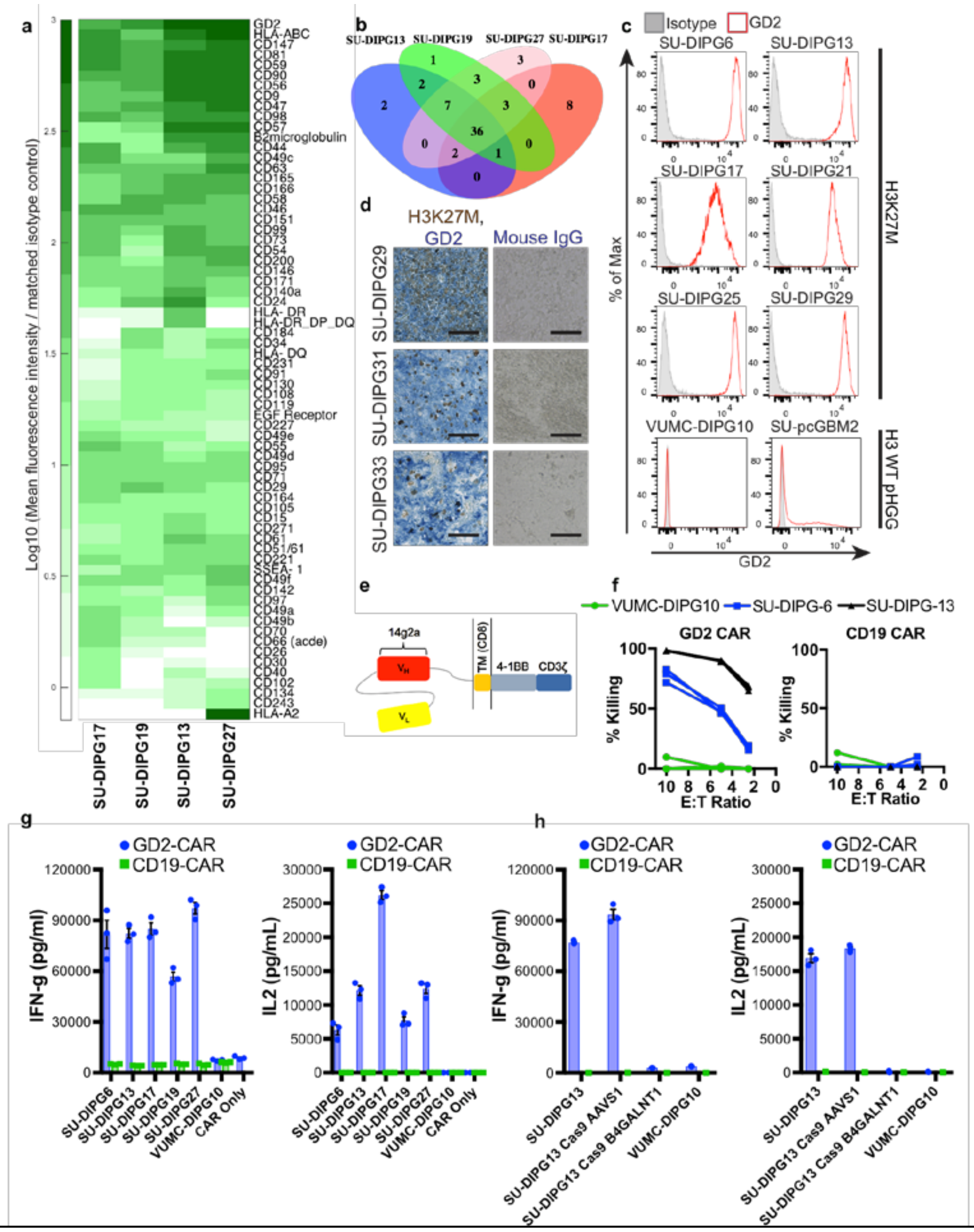

Figure 1: (**a**) Top 68 cell surface antigens expressed on DIPG as determined using flow cytometry screening of a monoclonal antibody panel in patient-derived DIPG cell cultures. (**b**) Assessment of hit overlap between screened cultures identified a total of 36 hits present at an median fluorescence intensity (MFI) of at least 10 times isotype control in all screened cultures. (**c**) Flow cytometry staining of histone 3 K27M DIPGs reveals high, generally homogeneous GD2 expression in contrast to histone 3 WT pediatric high-grade glioma cultures VUMC-DIPG10, diagnosed as a DIPG, and SU-pcGBM2, which arose in cortex. (**d**) Double immunohistochemistry of primary DIPG tumor specimens utilizing an antibody against mutant H3K27M (brown) to identify tumor cells and the anti-GD2 mAb 14g2a (blue) reveals extensive local GD2 expression in primary DIPG (scale bar = 100 microns). (**e**) Schematic of the GD2.4-1BB.z-CAR utilized in functional experiments. (**f/g**) GD2-CAR, but not CD19-CAR T cells, mediate potent lysis (**f**) and produce high levels of IFNγ and IL-2 (**g**) following co-culture with $GD2^{hi}$ H3K27M DIPG cells, but not $GD2^{lo/neg}$ H3WT tumor cells. (**h**) GD2-CAR T cells do not produce substantial levels of IFNγ or IL-2 following co-culture with H3K27M $GD2^{neg}$ line generated using CRISPR/Cas9 to knockout GD2 synthase compared with unmodified control cells or Cas9 targeting the control AAVS1 locus. Data as shown are mean±SEM.

2.4.1.2 GD2 CAR T cells mediate potent anti-tumor activity *in vivo*

To evaluate *in vivo* efficacy of GD2-CAR T-cells against DIPG, we prepared orthotopic mouse xenografts of DIPG cultures derived from post-mortem patient tissue. DIPG cultures were transduced with a luciferase-expressing construct to enable longitudinal monitoring of tumor burden. These xenograft models faithfully recapitulate the diffusely infiltrating histology of DIPG[31], [42]. Mice were distributed by tumor burden into equivalent treatment and control groups before receiving $1x10^7$ GD2-CAR or CD19-CAR T-cells by a single intravenous injection 7-8 weeks after establishment of pontine xenografts. Within 40 days post-treatment (DPT), marked reductions in tumor burden were observed across two independent GD2-CAR T-cell treated cohorts of mice bearing SU-DIPG6 xenografts[27],[21] (**Figure 2a)**. Similar results were observed in a second patient-derived xenograft model, SU-DIPG13FL[42] (**Figure 2e**). All GD2-CAR treated animals demonstrated complete tumor clearance by bioluminescence imaging. By contrast, no mice in the CD19-CAR T-cell control groups exhibited significant tumor regression[21]. At 50 DPT brains were harvested, and immunostaining for the mutant histone H3K27M – present in all engrafted tumor cells – revealed that GD2-CAR treated tumors had been largely eradicated (**Figure 2c,d,g,h,i**).

Most patient-derived orthotopic DIPG xenograft models require many months for lethality, limiting the ability to monitor survival benefit due to development of xenogeneic graft versus host disease (GVHD) after treatment with human T-cells[43]. We therefore used SU-DIPG-13P*, a model that exhibits a dense pattern of growth histologically[44], and is consistently lethal within one month. Substantial improvement in survival was seen in GD2-CAR treated animals compared with CD19-CAR treated controls (**Figure 3a**). GD2-CAR treated animals that survived the initial phase of glioma clearance returned to a visibly healthy state indistinguishable from untreated immunodeficient mice until the onset of GVHD symptoms 4+ weeks after CAR administration that invariably triggered endpoint criteria. Histologic analysis of the brains of endpoint GD2-CAR treated animals reveals clearance of this high-burden tumor while surrounding neural tissues appear grossly normal (**Figure 3b**).

Figure 2: GD2-CAR T cells mediate potent and lasting antitumor response in DIPG orthotopic xenografts

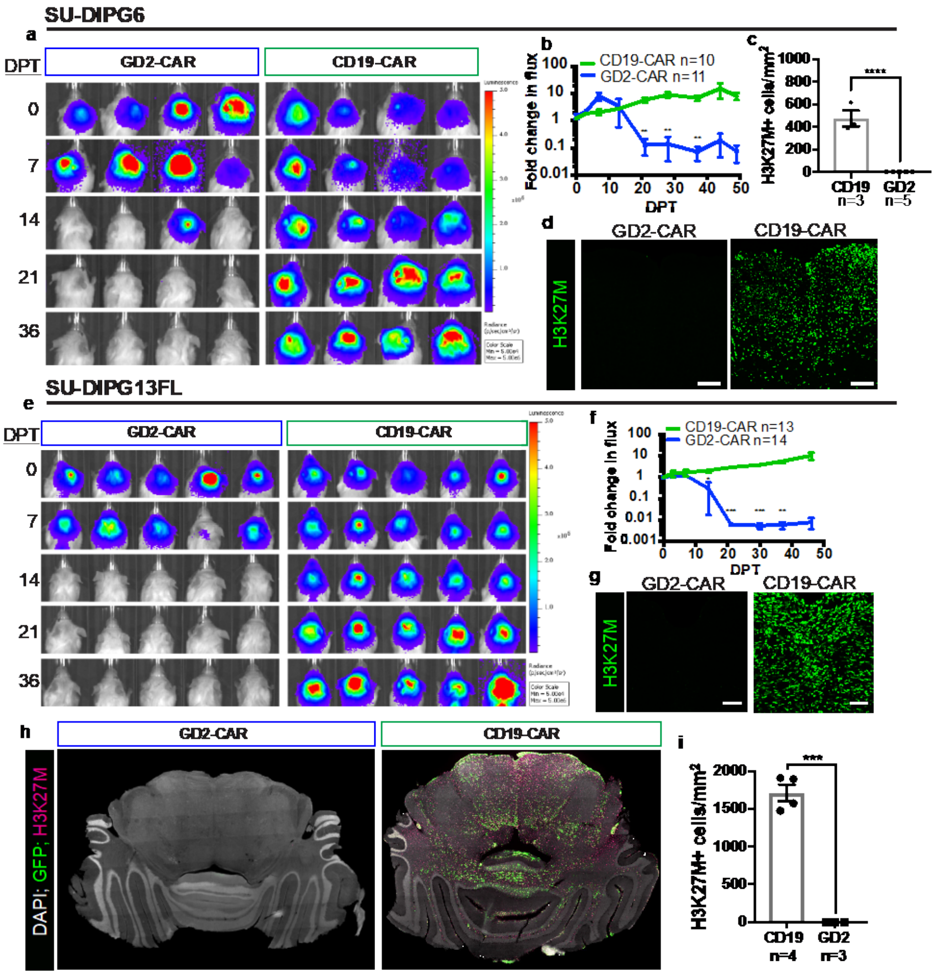

Figure 2: (**a**) Bioluminescence imaging of NSG mice xenografted with luciferase-expressing SU-DIPG 6 into the pons (color map for all images: radiance, min = 5E4, max = 5E6) and infused intravenously with 1E7 GD2-CAR or CD19-CAR T cells as designated. Between 14 and 28 days post treatment (DPT), a dramatic and universal antitumor response is observed in GD2-CAR T cell treated mice, while regression was not observed in those treated with CD19-CAR T cells. (**b**) Substantial reduction in luminescence flux at the tumor site occurred within 28 DPT. (**c**) Quantification of H3K27M+ tumor cell density within infiltrated brainstem regions of SU-DIPG6 GD2-CAR (n=5) vs. CD19-CAR (n=3) T cell treated mice. No region with even marginal tumor density at the injection site or elsewhere was found within GD2-CAR T cell treated animals. (**d**) Representative immunofluorescence confocal microscopy of CD19-CAR and GD2-CAR treated SU-DIPG6 tumors staining for the mutant histone H3K27M demonstrates clearance of tumor in GD2-CAR T cell treated animals. (**e,f**) In an additional patient-derived orthotopic xenograft model of DIPG, SU-DIPG13FL, tumor burden is substantially reduced within 21 DPT. (**g**) Representative immunofluorescent confocal microscopy of SU-DIPG13FL xenografts treated with CD19- or GD2-CAR T cells reveals clearance of H3K27M+, GFP+ tumor cells. (**h**) Tiled immunofluorescence images across infiltrating tumor regions identify no localized residual tumor burden. (**i**) Quantification of H3K27M+ tumor cell density within infiltrated brainstem regions of SU-DIPG13FL. Data as shown are mean±SEM. ****p<0.0001, ***p<0.001, **p<0.01, *p<0.05 by unpaired 2-tailed Student's t-test with Holm-Sidak correction for multiple comparisons applied for bioluminescence imaging data. Scale bars = 100 microns.

To better understand the etiology of treatment-related toxicity in these DIPG xenograft models, we examined the brains of treated SU-DIPG6 xenograft-bearing mice acutely at DPT14 (**Figure 3c**). GD2-CAR treatment was accompanied by a widespread inflammatory infiltrate involving brain parenchyma, meninges and ventricles that was most prominent in the brainstem. Ventriculomegaly was observed, consistent with hydrocephalus. We observed histologically normal-appearing neurons present throughout the pons, hippocampus, and cortex of GD2-CAR T-cell-treated animals with no evidence of neuronal cell killing nor other tissue destruction in this model (**Figure 3c**). Thus, neuropathological evaluation indicates that the toxicity described above results from brainstem inflammation and hydrocephalus due to fourth ventricular compression during the tumor-clearing interval and not on-target, off-tumor toxicity of GD2-CAR T-cells.

Figure 3: GD2-CAR T cell therapy improves survival in DIPG orthotopic xenografts

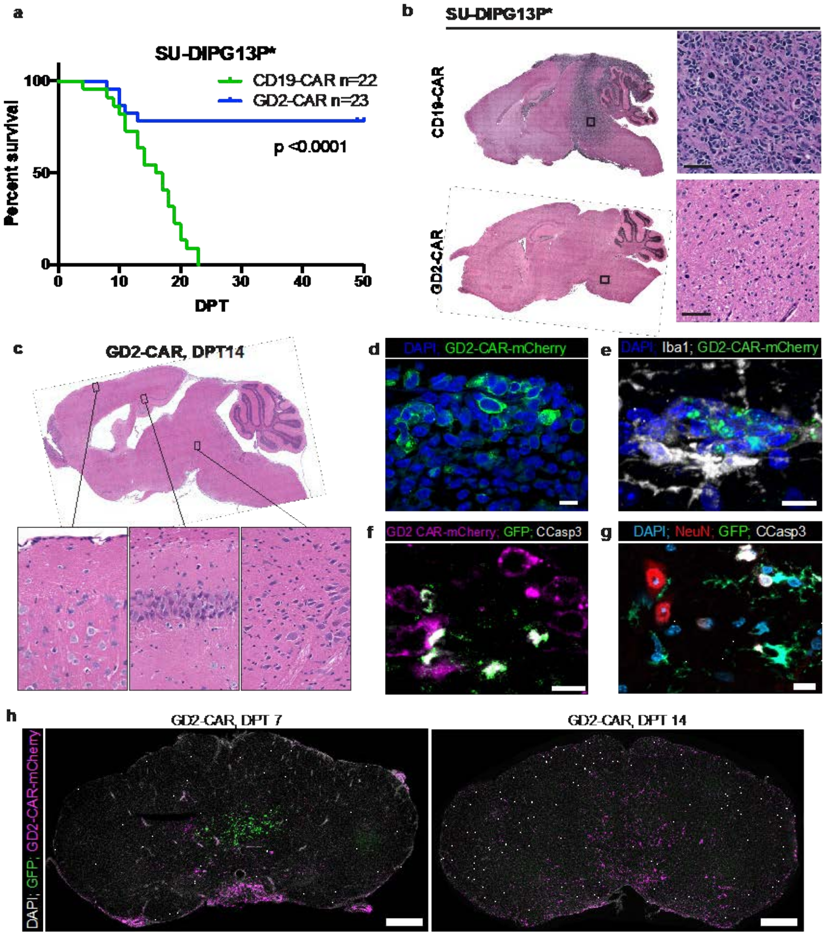

Figure 3: (**a**) Survival analysis of GD2-CAR T cell treated orthotopic xenografts in SU-DIPG-13P*, a particularly aggressive patient-derived xenograft model of DIPG that is lethal within one month of engraftment, reveals a robust survival improvement in GD2-CAR T cell treated animals (p<0.0001 Log-rank (Mantel-Cox) test,n=22 CD19-CAR and 23 GD2 CAR across 3 independent cohorts). While CD19-CAR T cell treated xenografts were universally lethal by study endpoint, all GD2-CAR T cell-treated animals that survived the acute toxicity of therapy survived to study endpoint at which time they manifested GVHD-like symptoms. (**b**) Hematoxylin-eosin staining of SU-DIPG13P* xenografts at DPT50 demonstrate clearance by GD2-CAR T cells of highly-infiltrative parenchymal tumor observed throughout the brain in CD19-CAR T cell-treated controls and normal gross tissue architecture. **(c)** Hematoxylin-eosin staining of SU-DIPG6 GD2-CAR T cell-treated xenograft analyzed at DPT14 demonstrates ventriculomegaly but histologically normal-appearing neurons in cortex, hippocampus, and brainstem (inset images). (**d**) Fluorescence microscopy of DPT7 SU-DIPG13FL xenografts reveals intravenously-administered GD2-CAR-mCherry T cells infiltrating the engrafted tumor.

Figure 3: (**e**) Representative image of infiltrating GD2-CAR-mCherry T cells at DPT14 in a SU-DIPG13FL xenografted medulla demonstrates spatial association with Iba1+ macrophages. (**f**) Representative image of GD2-CAR-mCherry T cell-mediated tumor cell killing with apoptosis of GFP+ tumor cells as evidenced by co-localization with cleaved caspase 3+ occurs in proximity to non-apoptotic NeuN+ neurons (**g**) in the xenografted pons, shown here at DPT7. (**h**) Representative images of GD2-CAR-mCherry T cells infiltrating the parenchyma of SU-DIPG13FL xenografts during the period of acute antitumor activity.

To visualize CAR T-cell infiltration into the parenchyma and tumor, we generated GD2-4-1BBz-mCherry and CD19-4-1BBz-mCherry fusion constructs (**Figure 3d**). By DPT7, GD2-CAR T-cells are extensively distributed throughout the leptomeninges of treated animals, leptomeningeal tumor has been largely eradicated, and few mCherry+ cells are present within the brain parenchyma (**Figure 3h**). By DPT14, mCherry+ GD2-CAR T-cells had widely infiltrated throughout the parenchyma and numerous foci of Iba1+ macrophages (**Figure 3e**) are present in the xenografted site, along with extensive apoptotic cleaved caspase 3+ cells (**Figure 3f**). Notably, very few cleaved caspase 3+ apoptotic cells are neurons as identified by NeuN double immunostaining (10 total apoptotic neurons identified across 4 mice; **Figure 3g**. This supports a model in which intravenously administered GD2-CAR T-cells enter through the meningeal lymphatic system[45], then subsequently infiltrate brain parenchyma, although the mechanism of CAR T-cell trafficking to the tumor remains to be defined. Given that resolution of tumor clearance and ventriculomegaly temporally coincide in treated animals, it is likely that antigen-specific antitumor activity, rather than on-target, off-tumor cell killing, precipitates neuroinflammation and edema during active tumoricidal activity that results in hydrocephalus.

2.5 Previous Human Experience with GD2-CAR T cell Therapies

Several previous and ongoing clinical trials have tested CARs targeting the disialoganglioside GD2. The first trial at Baylor College of Medicine utilized the scFv derived from dinutuximab in a CAR comprised of a TCR-ζ signaling domain but no embedded costimulatory endodomain[33]. This 1st generation GD2-CAR showed limited expansion *in vivo*, consistent with results using CARs targeting other antigens that did not integrate a costimulatory endodomain into the CAR[46]. Nonetheless, the 1st generation GD2 CAR was effective in inducing tumor regression and long-term disease control in a subset of patients. Low level persistence of these engineered T cell CARs were found up to 192 weeks after infusion and were associated with longer survival[33],[34]. These findings suggest that both the target GD2 and the approach have strong merit and that modifying the product based on scientific insights could improve outcome for more patients.

Figure 4: GD2-CAR (OX40.28.z)

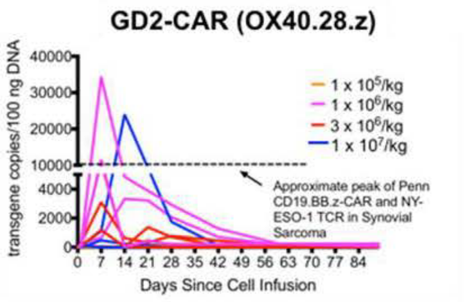

Figure 4: GD2-CAR.OX40.28.z undergo significant dose dependent expansion but show poor persistence *in vivo*. Shown is quantitative PCR based measurement of CAR expressing T cells in the peripheral blood of patients following infusion of CAR engineered T cells for patients enrolled on NCI 14-C-0059.

The Pediatric Oncology Branch NCI and others sought to develop a more potent GD2CART by incorporating costimulatory endodomains into the receptor and two clinical trials have tested GD2-CAR.28.OX40.z.iCasp9 for patients with osteosarcoma and neuroblastoma. Due to the risk for the GD2CART to mediate neurotoxicity due to low level GD2 expression on peripheral nerves and the central nervous system, and the significant peripheral neurotoxicity observed following treatment with dinutuximab for neuroblastoma[19], an inducible caspase-9 gene to was integrated into the vector allowing use of the small molecule dimerizing agent, AP1903, as a safety switch in the event of untoward toxicity[47]. It is important to note, however, that

significant toxicity was not observed in this trial and therefore utilization of this safety switch was not necessary.

Results from the Baylor experience using the GD2-CAR.OX40.28.z.iCasp9 CAR after cyclophosphamide/fludarabine conditioning demonstrated robust CAR T cell expansion without toxicity but no objective responses were observed and GD2-CAR persistence and CAR expansion was not improved by co-treatment with PD-1 blockers[48]. Additionally, they found expansion of myeloid cell populations in patients that may contribute to the limited efficacy and persistence of this CAR[48].

Fifteen patients were treated by investigators on the NCI trial (NCT#02107963) with T cells engineered to express GD2-CAR.OX40.28.z.iCasp9 (osteosarcoma n=12, neuroblastoma, n=3). The preparative regimen employed cyclophosphamide as a single agent and a very cautious dose escalation scheme starting at 1×10^5 GD2CART/kg. No dose limiting toxicity and no evidence for neurotoxicity, or other on-target, off-tumor toxicity was observed. AP1903 was never administered. Grade 1 cytokine release syndrome was observed consistent with expansion of GD2CART *in vivo*. Expansion/persistence of GD2CART in patients enrolled on this trial is shown in **Figure 4**. For the purpose of comparison, we include the maximal expansion observed using NY-ESO-1 engineered TCRs in synovial sarcoma[49] and CD19.BB.z CAR for leukemia at Penn (maximal expansion using CD19.28z CAR at NCI is approximately 100,000 copies/100mcgDNA). We conclude from these results that the GD2-CAR.OX40.28.z.iCasp9 T cells undergo substantial expansion *in vivo* but that they do not persist beyond 60 days. This data provides important evidence for the safety of GD2-CAR therapy since no significant toxicity was observed despite expansion of the engineered T cells *in vivo* to levels associated with clinically meaningful anti-cancer effects in other diseases. The data also illustrates the limited tumor exposure to the GD2CART accomplished using this platform due to their short persistence. Given the slow pace of responses seen with immunotherapy in solid tumors, we hypothesize that engineering the GD2-CAR platform to enhance persistence and/or functionality is essential if we are to increase the likelihood that meaningful antitumor effects will be induced against osteosarcoma and neuroblastoma.

2.5.1 Overcoming T Cell Exhaustion

Preclinical data suggests that the basis for the limited persistence/functionality of 14g2a GD2-CARs tested thus far is the development of T cell exhaustion as a result of tonic signaling of the GD2-CAR incorporating the CD28 costimulation domain[30]. This phenomena is substantially reduced, although not eliminated, by incorporation of the 4-1BB costimulatory domain, which appears to protect T cells from the detrimental effects of low level, chronic stimulation. We have demonstrated that the development of early exhaustion due to tonic signaling is a fundamental feature limiting the efficacy of a GD2-CAR incorporating the 14g2a scFv and a CD28 costimulatory domain[21]. We further demonstrated in this manuscript that functionality of the 14g2a CAR is substantially enhanced by switching the costimulatory domain from CD28 to 4-1BB, due to the ability for 4-1BB to provide “anti-exhaustion” effects while CD28 induces “pro-exhaustion” effects. Interestingly, in a follow-up manuscript, when both CD28 and 4-1BB are present, we demonstrated that the “pro-exhaustion” effect of CD28 is dominant [30]. Thus, our preclinical data provides strong rationale for eliminating CD28 costimulation in CARs incorporating 14g2a and for including 4-1BB as the preferred costimulatory domain. Note that we

have performed extensive efforts in an attempt to eliminate tonic signaling by mutations in the 14g2a scFv but these have been unsuccessful at creating a scFv that retains good antigen binding properties but lacks tonic signaling properties. Clinical experience with CD28-based versus 4-1BB-based CD19-CARs confirms the propensity for exhaustion since CD19.28.z-CAR show very limited persistence whilst CD19.BB.z-CARs often persist.

We have also attempted to change the linker between heavy and light chain (from a Whitlow linker as in our CD19 CAR to a GS4 x3 linker) but this did not significantly alter cytokine production by the CAR *in vitro*. We also found that adding a long spacer (CH2CH3 domain) in the GD2-4-1BB CAR abrogated its *in vitro* activity (for unknown reasons). In summary, smaller alterations in CAR architecture have not resulted in a convincingly superior product, but altering the costimulatory domain has.

Several trials have previously utilized the [30]14g2a binder and have demonstrated safety and efficacy. The first trials conducted at Baylor utilized the 14g2a binder with no costimulatory domain[33, 34]; in this trial significant antitumor effects were observed with long-term follow-up and no evidence for acute or chronic toxicity. The follow-up trial conducted at Baylor and NCI utilized the 14g2a binder with CD28 plus OX40 costimulatory domains and an Italian trial, conducted under the director of Professor Franco Locatelli, utilizes the 14g2A binder with CD28 and 4-1BB costimulatory endodomains. Fifteen patients have been treated with stage IV neuroblastoma (ages 2 – 18 years of age; 3 at DL1:1e6 cells/kg; 3 at DL2: 2e6 cells/kg; 3 at DL3: 3e6 cells/kg; 7 at the MTD/RD of 10e6 cells/kg) with no DLTs reported during dose escalation. The most common toxicities were grade 1/2 CRS, grade 3/4 neutropenia, thrombocytopenia and anemia[35]. AP1903 was administered in one patient for grade 4 CRS and was associated with clearance of > 90% of CAR-T cells within 48 hours (*personal communication, Locatelli*). Clinical responses (PR or CR) were observed in 10/15 patients infused (3 PD, 1 SD and 1 patient was NED at infusion)[35].

There is an ongoing University College London (UCL) trial which uses the KM666 scFv and a CD28 costimulatory domain. A Chinese trial is underway but the binder utilized is not known. This trial uses a so-called fourth generation CAR (CD28, 4-1BB, and CD27 costimulation). Both the ongoing UCL trial, the Italian trial and the Chinese trial have seen transient responses, but the UCL trial has been marked by limited T cell persistence, consistent with the development of early T cell exhaustion. We are in close contact with Dr. John Anderson, the principal investigator (PI) of the trial ongoing at UCL (NCT#02761915). His trial has climbed to high dose levels without toxicity and they are continuing to accrue (DL1: 1e7 1RG-CART/m^2 on Day 0; DL2: cyclophosphamide (300 mg/ m^2/day X 4 days followed by 1e7 1RG-CART/m^2 on Day 0; DL3: cyclophosphamide (300 mg/ m^2/day X 4 days) and fludarabine (25 mg/m^2/day X 5 days) followed by 1e7 1RG-CART/m^2 on Day 0; DL4: cyclophosphamide (300 mg/ m^2/day X 4 days) and fludarabine (25 mg/m^2/day X 5 days) followed by 1e8 1RG-CART/m^2 on Day 0; DL5: cyclophosphamide (300 mg/ m^2/day X 4 days) and fludarabine (25 mg/m^2/day X 5 days) followed by 5-10e8 1RG-CART/m^2 on Day 0. As more data becomes available from his trial, i.e. the safety and possible efficacy of higher dose levels, we will consider adding additional levels to our trial as necessary.

The trial proposed here will be the only clinical study using the the 14g2a scFv and 4-1BB costimulation exclusively, which based upon the studies presented in Long et al.[30], will substantially enhance persistence compared to those that also incorporate a CD28 signaling

endodomain. Given our significant findings surrounding T cell exhaustion resulting in loss of potency and persistence, the strong activity of the GD2-BBz CAR we have demonstrated in multiple papers and disease types[30],[1],[21], we feel it is warranted to use only 4-1BB costimulation.

2.5.2 GD2-CAR Viral Construct

This CAR proposed in this clinical trial is delivered via a retroviral vector that was generated by cloning the 14g2a scFv into a CAR with a CD8a transmembrane and hinge region, 4-1BB costimulatory domain, and CD3ζ as well as Bellicum's iCasp9 safety switch, separated by a ribosomal skip sequence (T2A); manufactured by Bellicum Pharmaceuticals. The viral construct is included in the IND submitted to the FDA.

2.5.3 GD2CART Cells Cultured in Dasatinib

While the 4-1BB costimulatory domain reduces the level of GD2 CAR exhaustion, it does not completely abrogate the effect. The Mackall laboratory has discovered that dasatinib, which is a known inhibitor of lymphocyte-specific tyrosine kinase (LCK), prevents CAR T cell signaling and inhibits CAR signaling[50] through a known mechanism of inhibition of LCK and other SRC tyrosine kinases[51]. In order to limit the exhaustive effects of CAR T cell tonic signaling, we have cultured GD2CART in the presence of dasatinib and found that at the end of the culture period, these cells express lower levels of exhaustion markers (TIM3 and LAG-3) and higher levels of CD62L and CCR7, markers associated with a healthy stem cell memory T cell subset (**Figure 5**a-b). In a xenograft model of osteosarcoma, GD2CART that were cultured in dasatinib until they were administered to mice significantly enhanced anti-tumor efficacy compared to those cultured in media without dasatinib (**Figure 5**).

Figure 5: Effects of Dasatinib on GD2CART

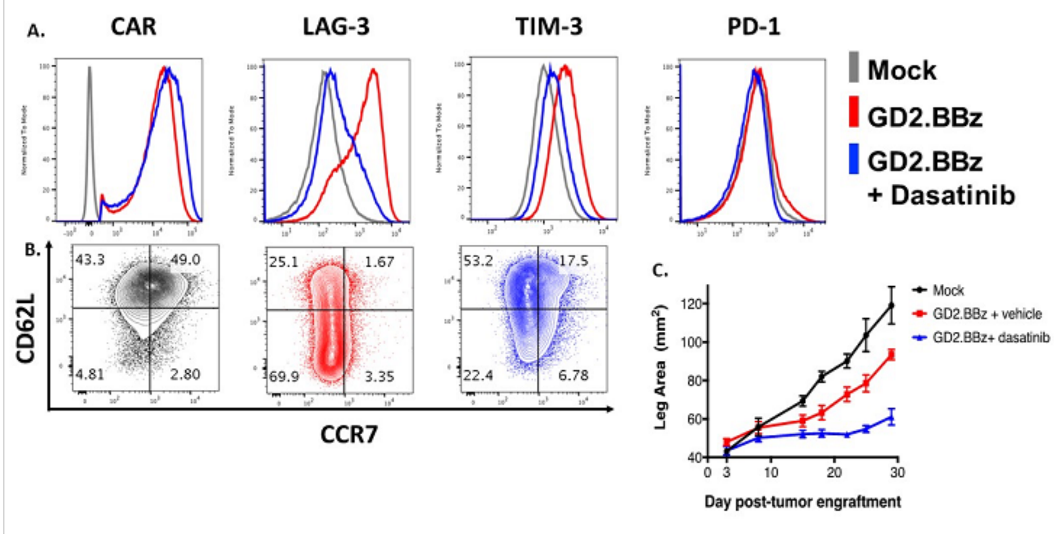

Figure 5: Effects of Dasatinib on GD2.BB.z CAR T Cells. (A) GD2.BB.z CAR T cells cultured in the presence of dasatinib express (A) lower levels of exhaustion markers and (B) higher levels of markers of T stem cell memory on day ten of culture than those cultured in the absence of the drug. (C) Mice were orthotopically injected with osteosarcoma cell line 143b and then treated with GD2.BB.z CAR T cells that were cultured in media with or without dasatinib. (C) Tumor growth was significantly delayed in mice treated with CAR T cells that had been cultured in dasatinib containing media compared to those that had not.

GD2CART cultured in dasatinib are protected from the detrimental effects of tonic signaling, are less exhausted, expand better (**Figure 6**), and we propose, are more effective upon adoptive transfer (**Figure 7**). GD2CART that are cultured in dasatinib have a memory like phenotype while those cultured without the drug are more slanted towards effector cells. GD2CART will therefore be manufactured with IL-7 and IL-15 in the presence of dasatinib (added on Day 3 and 5) with the goal of infusing a highly potent, non-exhausted CAR.

Figure 6: GD2 CAR T Culture Expansion on the Prodigy

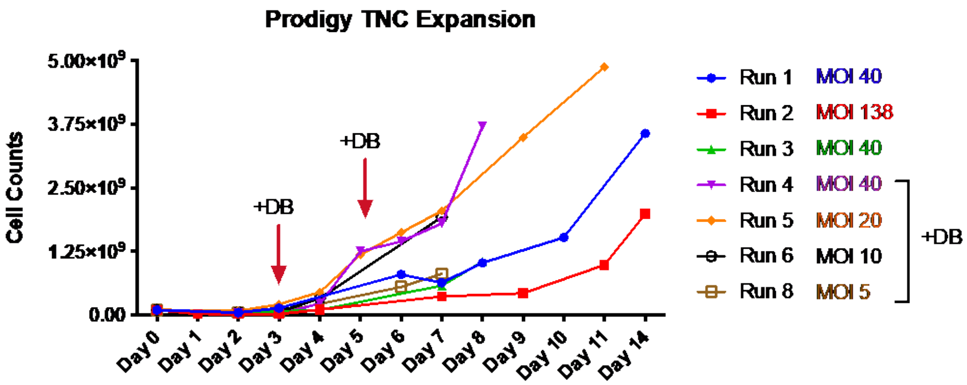

Figure 6: GD2 CAR T Culture Expansion on the Prodigy. Culture treatment with dasatinib improves expansion of GD2 CAR T cultures on the Prodigy, compared to control untreated GD2 CAR T Prodigy runs, which demonstrated less expansion by Day 7 harvest, the target for clinical manufacturing. Runs 1-3 were conducted without the addition of Dasatinib. Runs 4 -- 8 were conducted with the addition of Dasatinib (DB) (1 uM).

Figure 7: Improved GD2 CAR T Function with addition of dasatinib.

A)

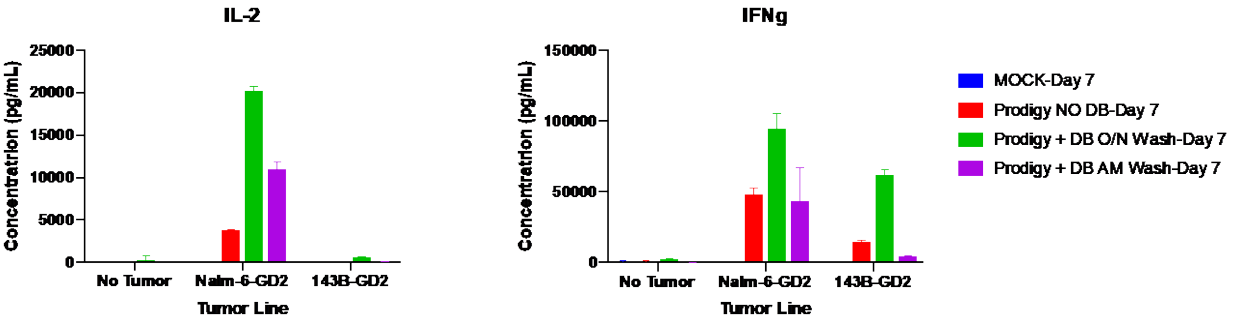

B)

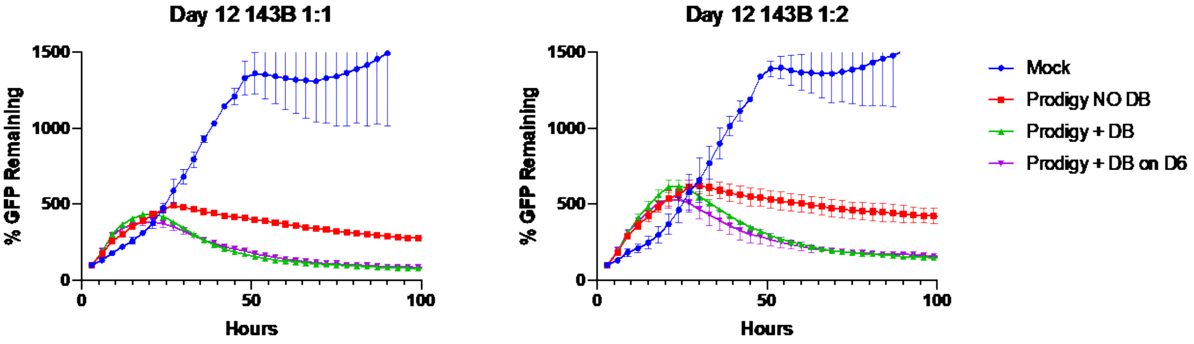

Figure 7: Improved GD2 CAR T Function with addition of dasatinib. A) The addition of dasatinib to the GD2 CAR T cells grown on the Prodigy (green and purple), improved the IL-2 and IFN-gamma cytokine secretion of GD2 CAR T cells against Nalm6-GD2 and 143B-GD2 tumor lines, compared to untreated GD2 CAR T cells (red). Green indicates GD2 CAR T cells grown on the Prodigy were washed out of dasatinib overnight, while purple indicates the cells were washed out the morning of the co-culture. All conditions were tested on Day 7, the target harvest day for clinical manufacturing. **B)** The addition of dasatinib to GD2 CAR T cells grown on the Prodigy (green and purple), improves the killing ability compared to untreated GD2 CAR T cells (red) against a 143B-GD2-GFP^{+} osteosarcoma line. The Prodigy + DB (green) condition received dasatinib treatment at Day 3 and Day 6, while the Prodigy + DB on D6 (purple) only received dasatinib treatment at Day 6 (1 uM dasatinib). 1:1 and 1:2 represent effector-to-target (E:T) ratios of GD2 CAR T:143B. Mock T cells (blue) exhibited no tumor killing. %GFP remaining represents % Tumor cells remaining from initial co-culture.

2.6 Safety Considerations for GD2 CAR Therapy

2.6.1 Risk of chemotherapy:

Toxicities resulting from fludarabine and cyclophosphamide in the doses proposed in the current study are well known and are what have been used in the prior CAR T cell therapy protocols. The

preparative regimen is designed to decrease the number of endogenous T cells, including T regulatory cells that may suppress CAR T cell mediated activity, and to induce increased availability of homeostatic cytokines thereby allowing for better engraftment of the transferred CAR T cells. The dose limiting toxicity for both fludarabine and cyclophosphamide is myelosuppression, however myelosuppressive effects are expected to be transient using the doses proposed. Other toxicities including fever, nausea, vomiting, stomatitis, diarrhea, anorexia, edema, skin rashes, myalgias, headache, agitation, and fatigue should be easily managed with appropriate supportive care. Hemorrhagic cystitis can occur in subjects who receive cyclophosphamide, but is unlikely given the relatively low dose administered in this trial and given that continuous intravenous infusion of normal saline will be used prophylactically as a uroprotective agent. Tumor lysis syndrome (TLS) following fludarabine and cyclophosphamide administration can occur in subjects with advanced bulky disease but this has not been described in the setting of brain tumors. Finally, opportunistic infections (protozoan, viral, fungal, and bacterial) have been observed post-fludarabine and cyclophosphamide, especially in heavily pre-treated individuals. Subjects will receive appropriate antimicrobial prophylaxis (e.g., Bactrim for PCP and acyclovir for HSV and VZV prophylaxis) during and after treatment as per institutional standards.

2.6.2 Risk of Autoimmunity:

Autoimmune toxicity is a theoretical risk of adoptive cell therapy trials for cancer and could occur if the transferred populations recognize the target antigen on normal tissues. Thus far, on-target, off-tumor toxicity of both CD19-CAR, CD19/CD22-CAR and CD22-CAR T cells has been restricted to B cell aplasia, which can be managed with immunoglobulin replacement therapy. There were no on-target, off-tumor toxicities noted in the previous GD2-CAR T cell therapy trials. Given that the GD2-CAR T cells tested in this study incorporates scFvs that have already been tested in clinical trials, it is unlikely that unexpected autoimmune toxicity will occur, but subjects will be monitored closely for the occurrence of unexpected toxicity and if it is observed, appropriate supportive care, or if severe, administration of AP1903 to ablate the CAR T cells.

2.6.3 Risks of Gene Therapy:

Risks of gene therapy include insertional mutagenesis or emergence of replication-competent retrovirus (RCR). While insertional mutagenesis is theoretically possible using retroviral vectors, this has only been observed in the setting of infants treated for X-SCID using retroviral vector-mediated gene transfer into CD34+ bone marrow stem cells. In the case of retroviral or lentiviral vector-mediated gene transfer into mature T-cells, there has been no evidence of long-term toxicities associated with these procedures since the first NCI sponsored gene transfer study in 1989.

The proposed protocol will test the cellular product for RCR, and patient follow up will comply with all current FDA guidelines. As the viral vectors used have been engineered to minimize the risk of emergence of replication competent retrovirus, subjects will be tested for RCR with long term follow-up as per FDA Guidance (July 2018). All participants will be followed for several years following receipt of this therapy and in the case of the development of a second malignancy, all efforts will be made to determine whether replication competent retrovirus has emerged.

2.6.4 Risk of Cytokine Release Syndrome:

Cytokine release syndrome has recently been defined as "a supraphysiologic response following any immune therapy that results in the activation or engagement of endogenous or infused T cell and/or immune effector cells"[52]. Symptoms are varied and can be progressive, must include fever at the onset and may include hypotension, capillary leak (hypoxia) and end organ dysfunction"[52], including arrhythmia, cardiomyopathy, heart block, renal failure, pleural effusions, transaminitis and coagulopathy[52]. Cytokine release syndrome (CRS) has been the topic of several reports and reviews[53],[54],[52]. In brief, CRS comprises a febrile, sepsis-like picture that results from hemodynamic and organ effects of supraphysiologic levels of inflammatory cytokines produced directly or indirectly by the activated T cells. Among the most important of these cytokines are IL-6 and IFN-gamma[29],[55],[53]. Laboratory alterations that commonly occur with CRS include C-reactive protein (CRP) and ferritin. One rare toxicity observed in CAR T cell patients, which is thought to overlap with CRS is hemophagocytic lymphohistiocytosis or macrophage activation syndrome (HLH/MAS), as it shares many features with CRS.

CRS can be safely managed with supportive care and in some cases, immunosuppression using anti-IL6R mAbs therapy and corticosteroids[54]. CRS is limited in subjects with low tumor burdens, as CRS severity correlates with the degree of CAR T cell expansion. CRS has been limited in the setting of CAR T cells for solid tumors, likely due to the relatively lower rate and degree of CAR T cell expansion that occurs in this setting. Recently forty-nine experts from experts in immune effector cell therapies, including CAR T therapy met at a meeting supported by the American Society for Transplant and Cell Therapy (ASTCT) and reached consensus on the grading definitions for immune effector cell-associated CRS[52] contained in section **13.2**, Appendix B.

2.6.5 Risk of Neurotoxicity

2.6.5.1 Immune Effector Cell-Associated Neurotoxicity Syndrome [ICANS]

Neurotoxicity is observed in a significant fraction of patients treated with CD19-CAR therapies [29],[55],[53] with toxicity ranging from mild to severe. Symptoms tend to be more diverse than observed with CRS[52]. The pathobiology of CD19-CAR associated neurotoxicity is not fully understood, but the clinical syndrome is associated with increased expansion of CAR T cells and identification of CAR T cells in the cerebrospinal fluid. Neurotoxicity may be seen more frequently in subjects with CNS leukemia[29], however subjects without documented CNS leukemia or lymphoma can also develop symptoms of neurotoxicity that range from mild to severe. Hence, no direct association between CNS disease burden and neurotoxicity severity has been observed in the context of CD19-CAR therapies for leukemia[29]. The syndrome typically manifests clinically as tremors, dysgraphia, impaired attention, confusion, aphasia and/or dysmetria, and occasionally seizures. Expressive aphasia appears to be a characteristic feature. Radiographic changes are variable and the syndrome typically resolves in 1-2 weeks and appears fully reversible in the vast majority of patients. The prevailing hypothesis regarding the pathophysiology of this syndrome is that it reflects non-specific neurotoxic effects of cytokines and/or activated T cells, rather than a direct on-target effect of CAR T cells. Indeed, the dose limiting toxicity of IL-6 when administered in a Phase 1 trial was neurotoxicity and transient aphasia was observed[56]. In further support of this hypothesis, the Jensen laboratory at Seattle Children's Hospital has recently developed a

rhesus model of neurotoxicity that utilizes a CD20-CAR rather than the CD19-CAR platform and the model appears to model the human syndrome well[57]. This provides further evidence against a direct, on-target effect involving CD19 in brain tissue. Lethal neurotoxicity has occurred in <1% of patients treated with CD19-CAR T cells and is associated with cerebral edema and herniation in patients with severe cytokine release syndrome. The occurrence of lethal neurotoxicity is reduced at lower doses of CD19-CAR T cells.

Based upon this experience, the ASTCT consensus group convened in 2017 defined ICANS as "a disorder characterized by a pathologic process involving the central nervous system following any immune therapy that results in the activation or engagement of endogenous or infused T cell and/or other immune effector cells. Symptoms or signs can be progressive and may include aphasia, altered level of consciousness, impairment of cognitive skills, motor weakness, seizure and cerebral edema"[52]. As CARs targeting non-CD19 antigens have not been associated with severe neurotoxicity in clinical trials, we are optimistic that cerebral edema associated with cytokine release syndrome will not occur in this trial, but patients will be observed carefully as discussed below.

2.6.5.2 Neurotoxicities due to tumor location

The neurotoxicities associated with GD2CART in subjects with DIPG could manifest differently than prior cell therapies due to the nature of the GD2 disialoganglioside on cell surfaces of tumors of neuroectodermal origin, as well was the nature and location of the tumor itself. To better understand the etiology of treatment-related neurotoxicity in DIPG xenograft models, we examined the brains of treated SU-DIPG6 xenograft-bearing mice acutely at DPT14 (**Figure 3**c). GD2-CAR treatment was accompanied by a widespread lymphocytic infiltrate involving brain parenchyma, meninges and ventricles that was most prominent in the brainstem. Ventriculomegaly was observed, consistent with hydrocephalus. Despite T cell infiltration, we observed histologically normal-appearing neurons present throughout the pons, hippocampus, and cortex of GD2-CAR T-cell-treated animals with no evidence of neuronal cell killing nor other tissue destruction in this model (**Figure 3**c). Thus, neuropathological evaluation indicates that the toxicity described above results from brainstem inflammation and hydrocephalus due to fourth ventricular compression during the tumor-clearing interval and not on-target, off-tumor toxicity of GD2-CAR T-cells. The uniqueness of the tumor location in this patient population requires the phase 1 dose escalation design be implemented in this patient population, separate and apart from patients with spinal DMG.

To mitigate the neurotoxicity risks in the DIPG patient population we plan to insert an intraventricular catheter (Ommaya catheter) prior to cell infusion for monitoring and if necessary, rapid and efficient treatment of increased intracranial pressure (ICP). Increased intracranial pressure will be treated by neurologists, intensivists and neurosurgeons who are familiar with the trial using the guidelines shown in section **13.2.3.5**. Briefly, upon onset of symptoms of increased ICP or evidence for increased ICP upon routine Ommaya monitoring, patients with DIPG will be appropriately positioned, imaged using MRI if possible or CT scan if MRI is not possible. Therapeutic interventions will generally include hypertonic saline, CSF removal via the Ommaya reservoir, and mannitol and dexamethasone. If these measures fail to control the increased ICP, consideration will be given to administering dasatinib to inhibit CAR function[50] and/or administration of AP1903 to permanently ablate the CAR T cells. Avastin may also be utilized as

it has shown some efficacy in the setting of tumor associated edema. An algorithm that provides an systematic approach to neurotoxicity management for patients enrolled on this trial is shown in in section **13.2.3.5**. The algorithm will serve as a guideline but is not meant to supercede clinical judgment in the management of neurotoxicity on this trial, and deviations from the algorithm will not be considered protocol deviations.

All subjects will receive a conditioning lymphodepletion chemotherapy regimen of fludarabine and cyclophosphamide (which may occur as an outpatient as determined by clinical judgment), followed by hospitalization for intravenous infusion of GD2CART on Day 0 and inpatient or outpatient monitoring with close proximity to the Stanford clinic until at least Day 28 according to the following guidelines.

1. Subjects with DIPG will have an intraventricular catheter (Ommaya catheter) placed following enrollment and prior to T cell infusion to allow monitoring, and treatment if necessary, of increased intracranial pressure (ICP).

2. All subjects will receive a conditioning lymphodepletion chemotherapy regimen of fludarabine and cyclophosphamide, followed by infusion of (Day 0). Subjects will be monitored closely as an inpatient for at least 28 days post-T cell infusion or as an outpatient in close proximity to the clinic if all toxicities are resolving/have resolved, at investigator's discretion, according to the following schedule:

3. Neurological exam: Daily from D0 to Day 28. Increase as clinically indicated.

4. Measurement of ICP via Ommaya Reservoir in subjects with DIPG: baseline D0 (prior to infusion), Day 4, Day 7, Day 11, Day 14, Day 21, Day 28. If any evidence of increased intracranial pressure or clinical deterioration suspected due to neurologic compromise, patient will be transferred to the ICU for more intensive ICP monitoring.

5. Radiographic imaging of subjects with DIPG: MRI at baseline (within 7 days of Day -4), D7, D14, D21 and D28. If clinical condition post-infusion prevents MRI, a CT will be obtained on those days. The standard MR parameters are listed on the PBTC NIC web page located at http://www.childrenshospital.org/research/centers-departmental-programs/pediatric-brain-tumor-consortium-neuroimaging-center under Neuroimaging Studies/ Specific MR Imaging Sequences- Open PBTC Protocols.

6. Neurooncologists, neurosurgeons and neurointensivists will be consulted and involved in every case and neurointensive care provided for management of neurologic deterioration and/or subclinical increase in intracranial pressure, guided by the algorithm shown in section **13.2.3.5**. The algorithm will serve as a guideline but is not meant to supercede clinical judgment in the management of neurotoxicity on this trial, and deviations from the algorithm will not be considered protocol deviations.

7. Consideration may also be given to administering dasatinib to inhibit CAR function[50] and/or administration of AP1903 to permanently ablate the CAR T cells if the patient develops uncontrolled, life threatening toxicity.

2.7 Risk for On-Target Toxicity and Rationale for The integrated suicide domain (iCasp9)

Anti-GD2 monoclonal antibodies cause pain requiring continuous infusion of narcotics for analgesia due to their interaction with peripheral nerves and possibly engagement of the complement system[58],[59],[19]. Notably however, clinical trials of CAR T cells targeting GD2 have not resulted in significant toxicity despite significant CAR T cell expansion and signs of on-tumor efficacy. Neither CNS toxicity nor peripheral neuropathy has been observed following GD2-CAR therapy in clinical trials. Furthermore, our murine model, which is relevant for assessing toxicity since GD2 is identical in mice and humans, demonstrated no evidence for on-target toxicity on normal neural tissue.

Richman, et al.[60] reported poorly characterized toxicity following administration of a CAR that incorporated the E101K scFv which is a mutated form of 14g2a. We published a response to this manuscript detailing major concerns regarding the conclusions published in this work[61]. To summarize, the relevance of toxicity seen with the E101K binder to that expected with 14g2a is questionable since the affinity is higher and it remains possible that this mutated binder could also acquire cross-reactivity with other gangliosides. To add to the concerns, our group also made a CAR consisting of the same E101K mutated “high-affinity” binder and demonstrated substantial antitumor activity in mice with no evidence for CNS toxicity[62]. This is across experiments in more than 200 mice (including those in our Letter to the Editor[61]. The authors of the cited manuscript also attempted to implicate a 3F8 (alternative scFv) based CAR as causing the same symptoms in mice, but the authors of a different manuscript on a 3F8 based CAR saw no such neurotoxicity[63]. It is unclear what is causing the toxicity in the models at this one center, but we offer some possible explanations in our letter (CRS/CRES, cross reactivity of the mutated binder with a different ganglioside, etc.)[61].

As described above, several clinical trials of 14g2a based CAR T cells, including those resulting in robust T cell expansion and clinically significant remissions and no patients have developed neurotoxicity. No patients in the ongoing UCL trial have developed neurotoxicity either and none were reported in the Chinese trial. Nonetheless, given that the GD2.BB.z-CAR has been designed to be more potent than those incorporating the CD28 costimulatory domain previously tested in clinical trials due to diminished T cell exhaustion, it is possible that we could observe on-target neurotoxicity related to low levels of GD2 on normal neural tissues. Furthermore, as noted above, even in the absence of on-target toxicity involving normal neural tissues, we could also observe toxicity related to tumor swelling and subsequent hydrocephalus or swelling of the brainstem itself, which could adversely impact vital functions. Due to the potential risk for toxicity therefore, we have incorporated a “suicide switch” within the CAR T cell receptor to allow for rapid ablation in the event of unacceptable toxicity. The iCasp9 system has been vetted in clinical trials for allogeneic transplant and results in efficient and rapid ablation of T cells and is the [60, 61, 63, 64] most effective suicide domain available to date, which provides an important measure of safety. We have also recently demonstrated that dasatinib can inhibit CAR signaling and could be utilized in the context of untoward toxicity[56], which may be utilized in this trial if untoward toxicity is observed. This trial will enroll patients with H3K27M diffuse pontine glioma and patients with spinal H3 K27M-mutant DMG who lack any curative options, thus justifying the administration of a therapeutic with potential risk.

2.8 CORRELATIVE STUDIES BACKGROUND

1. Measure expansion/persistence/phenotype of adoptively transferred GD2CART in the CSF and blood and correlate this with antitumor effects.
2. Conduct analyses of the manufactured T cell product and blood and CSF post-infusion to identify biomarkers associated with clinical benefit and/or enhanced CAR T cell expansion and/or persistence.
3. Assess whether changes in the level of ctDNA in the cerebrospinal can provide prognostic information and/or information regarding clonal evolution of DIPG over time.
4. Evaluate whether antigen expression or tumor microenvironment are correlated with response to CAR T cell.

2.9 STUDY DESIGN

2.9.1 Short Title for Study

GD2CART in DIPG or spinal DMG

2.9.2 Interventional model

One arm, open label single treatment

2.10 PROTOCOL RATIONALE AND SUMMARY

Diffuse Midline Gliomas (DMG) harboring the H3 K27M mutation, including diffuse intrinsic pontine glioma (DIPG) are lethal, high-grade pediatric brain tumors that are inoperable and pose significant challenges for treatment. Diffuse Intrinsic Pontine Glioma (DIPG) is a devastating, aggressive brain tumor of childhood arising in the ventral pons and comprises approximately 10-15% of pediatric brain tumors, with half of all pediatric malignant gliomas occurring in the brainstem[5]. While radiation therapy constitutes the mainstay of treatment, it only provides temporary improvement or stabilization of symptoms, extending overall survival by only 3 months. Prognosis is bleak with 90% of children expiring due to disease within 2 years of diagnosis. Hence there is an urgent need for novel effective therapies in this disease.

Chimeric Antigen Receptor (CAR) expressing T-cells is a new therapy wherein a subject's own T-cells are harvested and subsequently genetically modified in order to target cell surface antigens on specific cancer cells. In addition to their specificity, these CAR T-cells can be modified to be highly proliferative and possess the ability to negate immunosuppressive mechanisms making them ideal agents against highly aggressive cancers. We screened cell surface antigens in DIPG cultures in an attempt to identify potential targets for CAR T cell immunotherapy as a potential effective therapy. We observed that the disialoganglioside GD2 was expressed at high levels on each of twelve patient-derived DIPG cultures screened.

The primary goal of this trial is to evaluate the feasibility of generating GD2CART to meet the manufacturing specifications in children and young adults with H3K27M DIPG or spinal H3 K27M-mutant DMG, to establish the safety and RP2D of GD2CART in subjects with H3K27M DIPG and assess the safety of GD2CART in subjects with spinal H3 K27M-mutant DMG. In a preliminary fashion, this study will assess clinical benefit in an expanded cohort of subjects with DIPG and with DMG treated at RP2D.

Because GD2CART have not been previously administered to individuals with DIPG, a dose finding 3 + 3 dose escalation design will initially be used. Given the dismal survival prognosis of this disease with a median age at diagnosis of 6.3 years and the fact that children usually die from the disease within 2 years of initial diagnosis, children will be eligible for this first-in-DIPG cell therapy clinical trial. A conservative staggering protocol between patients will allow investigators to mitigate risk to the numbers of patients treated. Doses of GD2-CAR T cells of 1 x 10^7/kg have been administered in other clinical trials without toxicity, but to mitigate risk we have chosen a conservative starting cell dose of 1e6/kg, with a careful dose escalation (dose level 2: 3e6 transduced T cells/kg; dose level 3; 10e6 transduced T cells/kg). Once the MTD/RP2D is determined, an expansion cohort of subjects with H3K27M DIPG will be treated to further explore safety and conduct a preliminary evaluation of efficacy. In addition, a small cohort of children and young adults with spinal H3 K27M-mutant DMG will be treated with the GD2CART regimen at MTD/RP2D. Given prior experience in children with osteosarcoma and neuroblastoma receiving GD2 CAR T cells, a dose escalation design is not warranted in this patient population, but a safety lead in will ensure safety in this patient population. The initial 3 subjects will be staggered by 14 days, and should 1/3 subjects experience a DLT, the safety 'lead in' will be expanded to 6. If an additional subject in the safety lead in experiences a DLT the dose will be de-escalated and a safety assessment will be conducted at the lower dose of GD2CART.

To maximize safety of administration in the DIPG patient population, subjects will be carefully selected to minimize the risk of tumor swelling induced herniation or cord compression. Specifically, patients with thalamic lesions will not be eligible to reduce the risk of herniation as a result of tumor swelling. Subjects with biopsy documented H3K27M DIPG or with spinal H3 K27M-mutant DMG will be eligible if at least 6 weeks have elapsed since completion of first line radiation therapy.

A secondary objective will assess the capacity of AP9013 to mediate clearance of the genetically engineered cells to resolve toxicity, if unacceptable toxicity occurs (see section **12.5**) that is possibly, probably or likely related to the GD2CART.

Correlative analyses on this protocol or a companion study are defined above.

The precedent for conducting clinical trials of this scope was established by the ongoing clinical trials CCT5001/IRB-41382 and CCT5007/IRB-41383 conducted by Stanford Center for Cancer Cell Therapy.

This study will be conducted at Stanford by the principal investigator Michelle Monje, M.D., Ph.D. The Sponsor of this study will be Crystal Mackall, MD, Professor Pediatrics & Medicine, Associate Director, Stanford Cancer Institute.

2.10.1 Primary Outcome Type

Safety and Feasibility and RP2D

2.10.2 Investigational Agent

GD2CART: Autologous T cells transduced with retroviral vector (GD2.BB.z.iCasp9) Chimeric Antigen Receptor (GD2-CAR) cultured with dasatinib; following Fludarabine and Cyclophosphamide

2.10.3 IND number:

IND # 19801, Sponsor: Crystal L. Mackall, M.D.

2.10.4 Primary outcome measures:

This study will be registered on ClinicalTrials.gov but is not subject to the results reporting requirement.

1. Title: Rate of successful manufacture of GD2CART using a retroviral vector in the Miltenyi CliniMACS Prodigy ® system
    - Outcome Measure1: The percentage of apheresis samples (fresh or frozen) that are successfully processed and expanded to manufacture GD2CART that satisfy the target dose level and meet release specifications will be determined for each dose cohort.
    - Outcome Timeframe1: 14 days after apheresis or thawing of cryopreserved peripheral blood mononuclear cells (PBMCs).
2. Title: MTD/RP2D of GD2CART in subjects with H3K27M DIPG
    - Outcome Measure2: Incidence and severity of dose limiting toxicities (DLTs) following chemotherapy preparative regimen and infusion of GD2.BB.z.iCasp9-CAR T cells (GD2CART), as recorded and graded according to Common Terminology Criteria for Adverse Events (CTCAE) version 5.0 and Appendix B, section **13.2**, at each dose level tested in subjects with H3K27M DIPG following standard upfront radiation therapy.
    - Outcome Timeframe2: 28 days after infusion of GD2CART
3. Safety of GD2CART in subjects with spinal H3 K27M-mutant DMG treated at the RP2D
    - Outcome Measure3: Suspected adverse events and serious adverse events following chemotherapy preparative regimen and infusion of GD2CART, as recorded and graded according to Common Terminology Criteria for Adverse Events (CTCAE) version 5.0 and Appendix B, section **13.2**.
    - Outcome Timeframe3: 28 days after infusion of GD2CART

2.10.5 Secondary Outcome Measure

1. Title: Clinical benefit of GD2CART at RP2D in children and young adults with H3K27M DIPG and with spinal H3 K27M-mutant DMG

- Outcome Measure4: Primary determinant of clinical activity in subjects with DIPG and subjects with spinal H3 K27M-mutant DMG will be overall survival (OS) at 12 months post-diagnosis compared to historical controls.
  In addition, progression free survival (PFS), post-progression survival (PPS), and radiographic and clinical response will be assessed as best response (i.e. complete response [CR], partial response [PR], stable disease [SD], or progressive disease [PR]) at Day 28, 3 months and 6 months post-infusion.
- Timeframe4: Day 28, 3 months, 6 months, 9 months and 12 months and 24 months post GD2CART.

2. Title: Ability of AP1903 to eliminate persistence of genetically engineered cells, and allow resolution of toxicity in the event unacceptable toxicity considered possibly, probably or definitely related to GD2CART.

- Outcome Measure5: Resolution of toxicity to ≤ grade 2 within 72 hours of administration of AP1903
- Timeframe5: 72 hours after administration of AP1903

2.10.6 General Study Design

This is a phase 1, open label, single site dose escalation trial of GD2CART following cyclophosphamide/fludarabine lymphodepleting chemotherapy in subjects with H3K27M DIPG following at a minimum, completion of standard, up front radiotherapy. Given the universally fatal outcome in these patients, the very short post-progression survival and the increased risk with bulky disease identified in murine models[7], patients will be eligible for enrollment when they are at least 6 weeks from completion of standard upfront radiotherapy, regardless of documented evidence of progression, if all other eligibility criteria are met. In addition, given the established human safety data in administration of GD2-CARs and the median age of presentation of DIPG, the dose escalation cohort will not be restricted by patient age. The eligibility criteria for dose escalation in DIPG was chosen based of the following considerations:

1) A requirement for documented progression prior to enrollment is not feasible, since radiographic progression cannot be reliably distinguished from radionecrosis in all patients.
2) Post-progression survival is very short (median 2.3 months) and may not be long enough for patients to benefit from the effects of the GD2CART.
3) Median progression free survival following radiotherapy is 7.0 months (80.8% demonstrating progression within 12 months), therefore the risk of progression beyond 3 months is sufficiently high to justify the risks and morbidity associated with the investigational treatment regimen.
4) Preclinical models of DIPG demonstrate that bulky disease is a risk factor for treatment related morbidity and mortality due to hydrocephalus, therefore enrolling patients prior to documented clinical or radiographic progression will increase the likelihood that the therapy can be rendered safely.

Once the MTD/RP2D is established, the study will evaluate safety of administration, feasibility of manufacturing, and conduct a preliminary assessment of clinical benefit in children and young adults with H3K27M DIPG and spinal H3 K27M-mutant DMG. The CAR vector will incorporate an inducible Caspase 9 that can lead to efficient T cell apoptosis following exposure to AP1903 should toxicity require inactivation of the cell product.

Eligible subjects will undergo leukapheresis to obtain starting material for the CAR T cells. Cryopreserved PBMC stored from participation in other institutional cell therapy or cell collection studies or performed as standard collections may be used to generate the cellular product on this study as long as they meet the criteria established in this IND. In brief, cryopreserved PBMC will undergo selection, activation, transduction with the retroviral vector, expansion, and formulation in a GMP Facility using the Miltenyi CliniMACS Prodigy® system for the manufacture of

GD2.BB.z.iCasp9-CAR T cells. The product will be cryopreserved and transferred to Stanford's Cell Therapy Facility (CTF), from which the product will be distributed to the patient care unit for infusion.

Prior to infusion, patients will receive a lymphodepleting chemotherapy preparative regimen of fludarabine and cyclophosphamide (fludarabine 25 $mg/m^2/d$ x 3 days and cyclophosphamide 500 $mg/m^2/d$ x 3 days) on Days -4, -3, -2, followed by infusion of GD2CART on Day 0. Subjects will be closely monitored for cell therapy toxicities, including monitoring of intracranial pressure in subjects with DIPG (see secton **13.2.3.5**). Subjects will be evaluated after cell infusion for toxicity, antitumor effects and for persistence of CAR in blood samples and functionality of transduced T cells. Additional blood and CSF (if feasible) will be collected to complete correlative study analysis. Although GD2-CAR T cells have previously been administered in children, the product proposed here uses a unique costimulatory endodomain, and undergoes a slightly different manufacturing process and will be administered for the first time in subjects with DIPG. Hence a standard 3 + 3 dose escalation design will be used, enrolling 3 to 6 subjects sequentially using 3 dose levels of GD2CART (1e6 transduced T cells/kg (± 20%), 3e6 transduced T cells/kg (± 20%), and 10e6 transduced T cells/kg (± 20%)) to establish MTD/RP2D in children with DIPG. Once the RP2D is established, additional subjects will be enrolled to two groups: children with H3K27M DIPG (up to 20) or children and young adults with spinal H3 K27M-mutant DMG (up to 10), to further evaluate the safety and clinical activity of this regimen as outlined in Section **12.4.** The initial 3-6 subjects with DMG will undergo safety evaluations with 14 day stagger between subjects. If the DLT rate ≥ 30%, the dose will be de-escalated to ensure safety.

2.10.7 Number of Subjects

Initially 3-6 evaluable subjects with H3K27M DIPG may be enrolled sequentially in 3 dose levels of GD2CART to establish MTD/RP2D, for a minimum of 4 subjects and a maximum of 18. Once RP2D is established, up to 20 evaluable subjects with H3K27M DIPG (including those from the dose escalation phase) and up to 10 subjects with spinal H3 K27M-mutant DMG will be treated at the RP2D dose to further assess safety and perform a preliminary analysis of clinical activity. Up to 6 subjects may be enrolled to replace subjects with feasibility issues, and up to 6 subjects may replace inevaluable subjects. Thus, 18 + 14 + 10 + 6 +6 yields a maximum of 54 subjects with H3K27M DIPG or spinal H3 K27M-mutant DMG who may be enrolled to determine safety, feasibility, and preliminary efficacy of GD2CART therapy.

2.10.8 Study Duration

2.10.8.1 Primary Completion:

Up to 1-2 subjects will be accrued per month, and therefore this study may require up to 2.5 years to complete accrual. The study primary and secondary objectives will be completed in approximately 3.5 years.

2.10.8.2 Study Completion:

Subjects will be followed after treatment to evaluate toxicities, track disease progression, and to monitor for gene therapy effects. Short-term follow-up includes evaluations up until disease progression or subsequent alternative disease-directed therapy is initiated. In addition, long term

follow up for gene therapy according to the U.S. Food and Drug Administration (FDA) ***Guidance for Industry: Gene Therapy Clinical Trials – Observing Participants for Delayed Adverse Events*** will be conducted on every infused subject for the required 15 years post infusion of gene-edited cells on this study or an alternative long-term follow up protocol.

3 PARTICIPANT SELECTION AND ENROLLMENT PROCEDURES

All subjects must sign and date the Institutional Review Board (IRB) and Administrative Panel on Biosafety (APB) approved consent form before initiating any study specific procedures or activities that are not part of a subject's routine care.

The Screening Participant Eligibility Checklist on the following page must be completed in its entirety for each subject prior to registration. The completed, signed, and dated checklist must be retained in the subject's study file and the study's Regulatory Binder or an electronic version completed within the subject's medical record.

The study coordinator, treating physician, and an independent reviewer must verify that the participant's eligibility is accurate, complete, and legible in source records, as required by the CCTO SOP 'Confirmation of Participant Eligibility in Clinical Trials'. A description of the eligibility verification process should be included in the EPIC or other Electronic Medical Record progress note.

The protocol-specific checklist is **required** by the SRC and must be approved by the IRB.

3.1 Screening Participant Eligibility Checklist

Protocol Title:	**Phase 1 Clinical Trial of Autologous GD2 Chimeric Antigen Receptor (CAR) T cells (GD2CART) for Diffuse Intrinsic Pontine Glioma (DIPG) and Spinal Diffuse Midline Glioma (DMG)**
Protocol Number:	**CCT6005 / IRB-52934**
Sponsor Investigator:	**Michelle Monje, M.D., Ph.D.**

II. Subject Information:

Subject Name/ID:
Gender: ☐ Male ☐ Female

III. Study Information:

SRC Approved ☐ IRB Approved ☐ Contract signed ☐

IV. Inclusion/Exclusion Criteria- STAGE 1

**Inclusion Criteria**<br>(From IRB approved protocol)	**Yes**	**No**	**Supporting Documentation***
1. Disease Status<br>**For Dose Escalation**<br>Diagnosis of H3K27M mutated Diffuse Intrinsic Pontine Glioma (DIPG)<br>**For Dose Expansion**<br>• Diagnosis of H3K27M mutated Diffuse Intrinsic Pontine Glioma (DIPG), OR<br>• Diagnosis of spinal H3K27M mutated diffuse midline glioma (DMG)	☐<br>☐	☐<br>☐	or NA ☐
2. Age:<br>Greater than or equal to 2 year of age and less than or equal to 30 years of age.	☐	☐	
3. Prior Therapy:<br>• At least 6 weeks following completion of standard upfront radiation therapy.<br>• At least 3 weeks post chemotherapy or 5 half-lives, whichever is shorter, must have elapsed since any prior systemic therapy, except for systemic inhibitory/stimulatory immune checkpoint therapy that requires 5 half-lives.	☐	☐	
4. Performance Status:<br>Subjects > 16 years of age: Karnofsky ≥ 60% OR Eastern Cooperative Oncology Group (ECOG)	☐	☐	

performance status of 0 or 1; Subjects ≤ 16 years of age: Lansky scale ≥ 60% (See section 13.1, Appendix A)			
5. Normal Organ and Marrow Function (supportive care is allowed per institutional standards, i.e. filgrastim, transfusion)<br>i. ANC ≥ 1000/uL<br>ii. Platelet count ≥ 100,000/uL<br>iii. Absolute lymphocyte count ≥ 150/uL<br>iv. Hemoglobin ≥ 8 g/dL	ANC<br>Platelet<br>ALC<br>Hgb		
v. Adequate renal, hepatic, pulmonary and cardiac function defined as:<br>• Creatinine within institutional norms for age (i.e. ≤ 2 mg/dL in adults or according to table below in children <18 years) OR creatinine clearance (as estimated by Cockcroft Gault Equation) ≥ 60 mL/min	Creatinine	☐	

Age (Years)	Maximum Serum Creatinine (mg/dL)
≤5	0.8
5 < age ≤ 10	1.0
>10-18	1.2
>18	2.0

• Serum ALT/AST ≤ 3.0 ULN (grade 1)	AST/ALT	☐	
• Total bilirubin ≤ 1.5 mg/dl, except in subjects with Gilbert's syndrome.	Bilirubin	☐	
• Cardiac ejection fraction ≥ 45%, no evidence of physiologically significant pericardial effusion as determined by an ECHO, and no clinically significant ECG findings	LVEF	☐	
• Baseline oxygen saturation > 92% on room air	O2 Sat	☐	
6. Pregnancy Test<br>Females of childbearing potential must have a negative serum or urine pregnancy test (females who have undergone surgical sterilization are not considered to be of childbearing potential)	☐	☐	or NA ☐
7. Contraception<br>Subjects of child-bearing or child-fathering potential must be willing to practice birth control from the time of enrollment on this study and for four (4) months after receiving the preparative regimen or for as long as GD2CART cells are detectable in peripheral blood or CSF.	☐	☐	or NA ☐
8. Ability to give informed consent.<br>All subjects ≥ 18 years of age must be able to give informed consent. For subjects <18 years old their legal authorized representative (LAR) (i.e. parent or guardian) must give informed consent. Pediatric	☐	☐	

subjects will be included in age appropriate discussion and verbal assent will be obtained for those > 7 years of age, when appropriate. If a minor becomes of age during participation of this study, he/she will be asked to reconsent as an adult.			

**Exclusion Criteria**	**Yes**	**No**	**Supporting Documentation***
1) Tumor involvement of cerebellar vermis or hemispheres (pontocerebellar peduncles involvement is acceptable), thalamic lesions, or supratentoral lesions.	☐	☐	
2) Clinically significant swallowing dysfunction or prominent dysphagia, as determined by the clinical investigator.	☐	☐	
3) Current systemic corticosteroid therapy.	☐	☐	
4) Prior CAR therapy.	☐	☐	
5) Uncontrolled fungal, bacterial, viral, or other infection. Previously diagnosed infection for which the patient continues to receive antimicrobial therapy is permitted if responding to treatment and clinically stable.	☐	☐	
6) Ongoing infection with: • HIV, • Hepatitis B (HBsAg positive) or • Hepatitis C virus (anti-HCV positive). A history of hepatitis B or hepatitis C is permitted if the viral load is undetectable per quantitative PCR and/or nucleic acid testing.	☐	☐	
7) Clinically significant systemic illness or medical condition (e.g. significant cardiac, pulmonary, hepatic or other organ dysfunction), that in the judgement of the principal investigator is likely to interfere with assessment of safety or efficacy of the investigational regimen and its requirements.	☐	☐	
8) Women who are pregnant or breastfeeding.	☐	☐	or NA ☐
9) In the investigator's judgment, the subject is unlikely to complete all protocol-required study visits or procedures, including follow-up visits, or comply with the study requirements for participation.	☐	☐	
10) Known sensitivity or allergy to any agents/reagents used in this study.	☐	☐	
11) Primary immunodeficiency or history of autoimmune disease (e.g. Crohns, rheumatoid arthritis, systemic lupus) requiring systemic immunosuppression/systemic disease modifying agents within the last 2 years	☐	☐	

*All subject files must include supporting documentation to confirm subject eligibility. The method of confirmation can include, but is not limited to, laboratory test results, radiology test results, subject self-report, and medical record review.

IV. Statement of Eligibility

By signing this eligibility form, I verify that this subject is [☐ **eligible** / ☐ **ineligible**] for participation in this clinical trial. This study is approved by the Stanford Cancer Institute Scientific Review Committee, the Stanford IRB, and has finalized financial and contractual agreements as required by Stanford School of Medicine's Research Management Group.

Treating Physician Signature:	Date:
Printed Name:	
Secondary Reviewer Signature:	Date:
Printed Name:	
Study Coordinator Signature:	Date:
Printed Name:	

3.2 Informed Consent Process

All participants must be provided a consent form describing the study with sufficient information for participants to make an informed decision regarding their participation. The investigational nature and research objectives of this trial, the procedures and treatments involved and their attendant risks and discomforts and potential benefits, and alternative therapies will be carefully explained to the subject, and asked to review it and to ask questions prior to agreeing to participate in this protocol. The subject is reassured that participation on trial is entirely voluntary and that he/she can withdraw or decide against treatment at any time without adverse consequences.

Pediatric subjects will be included in age appropriate discussion. Verbal assent will be obtained for those ≥ 7 years of age when deemed appropriate by the clinician and the child's parents or legally authorized representative(s). Should a minor subject reach the age of majority during participation in this trial (active therapy or follow up) they will be asked to complete the informed consent as an adult.

The original signed copy of the consent document must be retained in the research file and a copy placed in the medical record.

3.3 SUBJECT SCREENING ASSESSMENTS AND REGISTRATION

3.3.1 General considerations for Subject Screening

The screening period begins on the date the subject and/or the subject's LAR signs the IRB/IEC approved ICF and continues through confirmation of enrollment (the date triple review eligibility sign off is completed to verify that the participant's eligibility is accurate, complete, and legible in source records). Informed consent must be obtained before completion of any non-standard of care study specific procedures. Procedures that are part of standard of care are not considered study specific procedures and may be performed prior to obtaining consent and used to confirm eligibility.

After written informed consent has been obtained, subjects will be screened to confirm study eligibility and participation. Only subjects who meet the eligibility criteria listed in sections **3.1** will be enrolled in the study. If at any time prior to enrollment the subject fails to meet the eligibility criteria, the subject should be designated as a screen failure on the subject screening log with the reasons for failing screening.

All subjects will undergo the screening procedures, which includes a comprehensive history and physical exam performed by a study physician or nurse practitioner. Imaging and organ specific studies will be performed as per institutional guidelines. Confirmation of this data must occur within 28 days of enrollment, unless specified otherwise.

3.3.2 Study Enrollment

Before enrollment of a subject into the study, the responsible physician must ensure the subject meets all eligibility criteria using the Study Screening Procedures outlined in section **5.1**. Eligibility criteria will be reviewed and confirmed by the Principal Investigator or designee prior to any subject being enrolled into the study (section **3.1**).

Enrollment will be defined as the date triple review eligibility sign off is completed to verify that the participant's eligibility is accurate, complete, and legible in source records. At time of enrollment, each subject will receive a unique subject identification number. This number will be used to identify the subject throughout the study and must be used on all study documentation related to the subject. Furthermore, the subject identification number must remain constant throughout the entire clinical study, it must not be changed after enrollment or if the subject is rescreened or retreated.

4 TREATMENT PLAN

4.1 OVERVIEW

This is a single site, open label Phase I study in subjects with H3K27M DIPG who have undergone front-line radiation therapy. In light of the previous clinical experience in which GD2-CAR T cells were administered safely in children and adult subjects with osteosarcoma and neuroblastoma, and because DIPG occurs essentially exclusively in children <12 years, we plan enrollment of pediatric subjects during the dose escalation phase. Because GD2CART have not been previously administered in subjects with DIPG, and because preclinical models demonstrate that a significant toxicity risk related to disease within the pons which may swell during effective therapy, we will conduct a standard 3 + 3 dose escalation design to establish MTD/RP2D using 3 dose levels (1e6 transduced T cells/kg (± 20%), 3e6 transduced T cells/kg (± 20%), and 10e6 transduced T cells/kg (± 20%)) in the cohort of subjects with DIPG. Once the RP2D is defined, we will expand this cohort to assess efficacy (n=20 evaluable subjects) and further determine safety. In addition we will also enroll a cohort of 10 children and young adults with spinal DMG. These patients are not as at great of risk for tumor swelling induced toxicity and therefore would not be informative during the dose escalation phase. But to ensure safety in this cohort, a safety 'lead-in' will be conducted in 3-6 subjects with DMG and if ≥ 30% experience DLT, the dose will be de-escalated

Subjects are eligible for enrollment when they are at least 6 weeks from completion of standard upfront radiotherapy, regardless of documented evidence of progression, and if all other eligibility criteria are met. This eligibility criterion was chosen based of the following considerations:

1) A requirement for documented progression prior to enrollment is not feasible, since radiographic progression cannot be reliably distinguished from radionecrosis in all patients.

2) Post-progression survival is very short (median 2.3 months) and may not be long enough for patients to benefit from the effects of the GD2CART.

3) Median progression free survival following radiotherapy is 7.0 months (80.8% demonstrating progression within 12 months), therefore the risk of progression beyond 3 months is sufficiently high to justify the risks and morbidity associated with the investigational treatment regimen.

4) Preclinical models of DIPG demonstrate that bulky disease is a risk factor for treatment related morbidity and mortality due to hydrocephalus, therefore enrolling patients prior to documented clinical or radiographic progression will increase the likelihood that the therapy can be rendered safely.

An Ommaya catheter will be inserted after enrollment and prior to GD2CART infusion in subjects with DIPG. Non-mobilized autologous PBMC will be obtained by leukapheresis in all subjects

and transduced with GD2.BB.z.iCasp9 retroviral vector. Cryopreserved PBMC stored from participation in other institutional cell therapy or cell collection studies may be used to generate the cellular product on this study as long as they meet the criteria established in this IND. Subjects will receive a lymphodepleting chemotherapy preparative regimen with fludarabine and cyclophosphamide, followed by infusion of GD2CART at Stanford Lucile Packard Children's Hospital (LPCH). The study will evaluate safety of administration, feasibility of manufacturing, identify the recommended phase 2 dose (RP2D) and conduct a preliminary assessment of clinical activity in subjects with DIPG by evaluating overall survival (OS) compared to historical controls and in subjects with DMG by describing overall survival (OS). In addition, radiographic response, progression free survival (PFS) will be evaluated in all subjects . The CAR vector will incorporate an inducible Caspase 9 that can lead to efficient T cell apoptosis following exposure to AP1903 should toxicity require inactivation of the cell product.

The feasibility of generating GD2CART using a retroviral vector in the Miltenyi CliniMACS Prodigy® 'all-in-one' cell processing system will be evaluated as a primary objective. If feasibility of cell production is not met (i.e. 3 of the first 6 subjects' cells cannot be produced to meet the established release criteria) further enrollment will be paused pending evaluation of the manufacturing process, and modifications made appropriate to improving feasibility prior to continuing enrollment. This may require a protocol or IND amendment. In any event, if 3 of the first 6 cell products manufactured do not meet established criteria, it will be reported to the IRB.

While this CAR has been administered to subjects with neuroblastoma and osteosarcoma, it has not been previously administered to subjects with DIPG, which offers new challenges to management of potential CAR therapy-related toxicities. To mitigate risk in this patient population, subjects will be carefully selected to avoid large disease burden, and subjects with DIPG will have an intraventricular catheter (Ommaya catheter) placed prior to cell infusion for monitoring and if necessary, rapid and efficient treatment of increased intracranial pressure. Increased intracranial pressure will be treated by neurologists, intensivists and neurosurgeons who are familiar with the trial using the guidelines shown in section **13.2.3.5**.

4.1.1 Determination of Maximum Tolerated Dose (MTD)/Recommended Phase 2 Dose (RP2D)

Initially the MTD/RP2D will be determined in the pediatric DIPG population by treating 3 to 6 subjects sequentially in 3 dose finding cohorts of GD2CART (1e6 transduced T cells/kg (± 20%), 3e6 transduced T cells/kg (± 20%), and 10e6 transduced T cells/kg (± 20%)). The DLT assessment period is defined as 28 days. A 28 day safety assessment will follow infusion of GD2CART in the first subject in each dose cohort prior to cell infusion of the second subject. Fourteen (14) day safety assessment will follow infusion of GD2CART of subsequent subjects in each dose cohort. Four weeks (28 days) must elapse after completion of cell infusion in the final subject in each dose cohort to allow for safety assessment before treating subjects at the next higher dose cohort. Therefore, infusion at the next higher dose will not proceed until the last subject infused on the completed dose cohort has been observed for at least 28-days after infusion of GD2CART. If more than one (1) DLT occurs in the first dose level, (2 out of 6) the dose will be de-escalated to Dose level -1 (3e5 transduced T cells/kg (± 20%)) for safety evaluation. If ≥ 2 DLTs occur at dose level -1, the study will be suspended pending discussion with the FDA, IRB, APB to improve safety. Otherwise, dose escalation will proceed as outlined in section **5.5.4.**

4.1.2 Dose Expansion Cohorts

Once the MTD/RP2D is established (MTD is defined as the dose level below that in which 2/6 subjects experience DLTs), additional subjects will be enrolled to further evaluate the safety and conduct a preliminary evaluation of clinical benefit of this regimen in two cohorts:

1. Subjects with H3K27M DIPG (n=20 total)
2. Subjects with spinal H3K27M-mutant DMG (n=10 total).

Subjects with H3K27M DIPG: The trial will continue to evaluate safety in the dose-expansion cohort. Safety boundaries will be used to monitor a DLT rate of 30%. A dose-expansion cohort of subjects with H3K27M DIPG will enroll up to 20 evaluable subjects to monitor safety and indicate efficacy. The trial will evaluate safety profile after the first 4 and 9 subjects with DIPG are evaluable in the dose-expansion cohort, corresponding to a total of 10 and 15 evaluable subjects treated at the MTD/RP2D with 6 subjects from the dose escalation cohort.

Subjects with spinal H3K27M-mutant DMG: In addition, a safety 'lead-in' will be conducted to evaluate the first 3 - 6 subjects with H3K27M-mutant DMG, whereby, the first 3 subjects will be staggered by 14 days between infusions and if 1/3 subjects experiences a DLT, the staggering will continue in the next 3 subjects, for a total of 6 subjects. If 2/6 subjects develop DLT in this cohort, the GD2CART dose will be de-escalated in this population. If de-escalation occurs, safety boundaries will be repeated after every 3 additional subjects. If the de-escalated dose is found to be unsafe in subjects with DMG, the trial will stop enrollment for this disease population.

NOTE: If indications of clinical benefit are observed at a dose level prior to reaching MTD, this dose level may be expanded to explore efficacy signals in an attempt to reduce the risk of DLTs in this patient population.

4.2 GENERAL CONCOMITANT MEDICATION AND SUPPORTIVE CARE GUIDELINES

4.2.1 Infection Prophylaxis

Any temperature of >38° C will require initiation of the fever work-up and treatment according to institutional standards.

4.2.1.1 Viral Prophylaxis

All subjects will be treated as high risk and will receive viral prophylaxis according to institutional standards.

4.2.1.2 Fungal Prophylaxis

All subjects will be treated as high risk and will receive fungal prophylaxis according to institutional standards.

4.2.2 Blood Product Support for Anemia and Thrombocytopenia

Using CBC's as a guide, the subject will receive platelets and packed red blood cells (PRBC's) as needed. Attempts will be made to keep Hb > 8.0 gm/dl, and plts > 50,000/mm3. All blood products with the exception of the lymphocyte product will be irradiated. Leukocyte filters will be utilized

for all blood and platelet transfusions to decrease sensitization to transfused WBC's and decrease the risk of CMV infection.

In subjects with coagulopathy, attempts will be made to keep fibrinogen ≥ the lower limit of normal.

4.2.3 Cytokine Release Syndrome

Cytokine release syndrome, a toxicity associated with infusion of CAR T cell therapy, has been described in section **2.6.4.** Grading and management of CRS in this protocol will follow the guidelines in section **13.2**, Appendix B[29],[52] which includes diligent supportive care and evaluations for infection, with immunosuppression using anti-IL6R mAbs and/or corticosteroids reserved for more severe cases. Because understanding of the constellation of symptoms defining CRS is evolving, AE CRFs will capture both the syndrome and the individual symptomatology of CRS.

4.2.4 Neurotoxicity

Neurotoxicity (e.g., encephalopathy, somnolence, aphasia) has been observed with CAR T cell therapies (immune effector cell therapies) and will be scored using the tools available in section **13.2,** Appendix B, using a combination of the ASTCT Immune effector Cell-Associated Neurotoxicity Syndrome (ICANS) Consensus Grading for Adults with the Immune effector Cell-associated Encephalopathy (ICE) assessment tool; or ASTCT Immune effector Cell-Associated Neurotoxicity Syndrome (ICANS) Consensus Grading for Children with either the ICE assessment tool or the Cornell Assessment of Pediatric Delirium (CAPD)[52].

Administration of GD2CART will occur after placement of an Ommaya catheter in subjects with DIPG to monitor intracranial pressure. Neurooncologists, neurosurgeons and neurointensivists will be consulted and involved in every case for management of neurologic deterioration and/or subclinical increase in intracranial pressure. If patients develop uncontrolled toxicities, they will be given AP1903 to ablate the CAR T cell product.

All subjects will receive levetiracetam (Keppra) beginning the day before cell infusion. For good clinical practice, clinicians and staff will evaluate the ICE or CAPD score or standard neuro checks every 8 hours (± 2 hours) or as clinically indicated. Neurologic evaluations will be assessed with the scheduled clinical evaluation daily.

Evaluation of any new onset of neurotoxicity should consider recommended interventions in Appendix B section **13.2.3.** These recommendations should serve as guidance for toxicity management, but deviation from the guidance will not be considered a protocol deviation, as all toxicity therapy should be at the treating physician's discretion. If increased ICP is suspected, recommendations for management consideration are provided in section **13.2.3.5.**

Medications with sedative properties should be avoided if possible unless required to manage seizures, i.e. benzodiazepines. Subjects and their families/caregivers should be warned of the risk of late neurotoxicity thru day 28 and told to seek immediate medical attention for any new symptoms of neurotoxicity.

4.2.5 Unacceptable Toxicity

GD2CART incorporates an inducible Caspase 9 gene (iCasp9) that can act as a suicide safety switch in the event of unacceptable toxicity, defined as life-threatening/grade 4 toxicity believed by the investigators to cause substantial risk to the subject, which is possibly, probably or definitely related to the cellular therapy. In the presence of the small molecule AP1903, the iCasp9 promolecule dimerizes and activates the intrinsic apoptotic pathway, leading to cell death. Should subjects encounter unacceptable toxicity that cannot be safely managed with supportive care as described within this protocol, AP1903 may be administered to rapidly deplete GD2CART levels (see section 6.9 for more information on AP1903).

4.3 CRITERIA FOR REMOVAL FROM PROTOCOL THERAPY AND OFF STUDY CRITERIA

4.3.1 Criteria for removal prior to GD2CART cell infusion

Subjects will be taken off treatment and followed until effects of leukapheresis or chemotherapy have reversed and all toxicities are resolved to Grade 1 or baseline for any of the following:

- ✓ General or specific changes in the subject's condition render the subject unacceptable for cell infusion on this study in the judgment of the investigator.
- ✓ Pregnancy in a female of child-bearing potential.
- ✓ Cells do not meet infusion release criteria (criteria other than targeted dose).

Once toxicities and effects of leukapheresis or chemotherapy resolve, subjects who are unable to undergo cell infusion will be removed from this study.

4.3.2 Criteria for removal from the option for a 2nd cell infusion:

Subjects will not be eligible for further therapy (2nd cell infusion as outlined in section 5.6) (and will be followed until off-study criteria are met) for the following:

- ✓ Dose limiting toxicity (DLT) after first infusion. The definition of DLT is in section 5.4.5, unless approved by the FDA and IRB in advance of the second infusion.
- ✓ Pregnancy in a female of child-bearing potential.
- ✓ No cells available for re-treatment.

4.3.3 Off-Study Criteria

- ✓ Subject withdrawal of consent (in which case the reason will be documented, if possible). Subjects who withdraw consent for additional procedures will be requested to participate in long-term follow up.
- ✓ Subject who meet any of the withdrawal criteria listed in Section 4.3.1 and who have recovered from all study-induced toxicity.
- ✓ Subject lost to follow-up. Should a subject fail to return to the clinic for a scheduled protocol specific visit, site will need to make 2 attempts by a combination of

telephone and mail to contact the subject. Site must document both attempts to contact the subject. If a subject does not respond within 1 month after the second contact the subject will be considered lost to follow-up and no additional contact will be required.

- ✓ Death
- ✓ Conclusion of the 15 years of follow up, or subject enrolls in a separate long-term follow up protocol for subjects receiving gene transfer.

4.3.4 Off-Study Procedure

Off study date and reason should be documented in the study CRFs. For subjects withdrawing consent, the investigator should inquire whether the subject agrees to allow chart review of normal medical care procedures and/or long term follow-up of gene therapy research participants. Cell products and biological samples from off-study subjects may continue to be used for process development and correlative studies, as outlined in the consent form.

5 SUBJECT STUDY PROCEDURES

All subjects will undergo the screening procedure, which includes a comprehensive history and physical exam performed by a study physician as outlined below. Imaging studies will be performed as per institutional guidelines. The following screening tests must be performed within 28 days prior to enrollment unless specified otherwise.

5.1 Subject Screening

The screening period begins on the date the subject/LAR signs the IRB and APB approved consent form and continues through confirmation of enrollment. Procedures that are to be performed as part of the practice of medicine and which would be done whether or not study entry was contemplated, such as for diagnosis or treatment of a disease or medical condition, may be performed and the results subsequently used for determining study eligibility without first obtaining consent. Informed consent must be obtained prior to initiation of any clinical screening procedures that are performed solely for the purpose of determining eligibility for research, i.e. withdrawal from medication (wash-out period). Only subjects who meet the eligibility criteria listed in section **3** will be enrolled in the study.

Screening will proceed as outlined in section 3.3; the following screening evaluations must be completed **within 28 days of enrollment unless otherwise specified**.

(a) Medical history

The subject's complete history through review of medical records and by interview will be collected and recorded. Concurrent medical signs and symptoms must be documented to establish baseline severities. A disease history, including the date of initial diagnosis, verification of diagnosis of H3K27M DIPG or spinal DMG (H3K27M mutation may be determined at any point since diagnosis), and prior radiotherapy, or other disease directed therapies and the disease response, and duration of response to the radiotherapy or prior treatment also will be recorded.

(b) Physical examination

A complete physical examination will be performed. The exam will include general appearance of the subject, height and weight, examination of the skin, eyes and ears, nose, throat, lungs, heart, abdomen, extremities, musculoskeletal system, and a thorough nervous system evaluation.

(c) Vital signs, including blood pressure, heart rate, oxygen saturation and temperature will be recorded.

(d) Performance status (ECOG or Lansky or Karnofsky) see Appendix A, Section **13.1.**

(e) Electrocardiogram (ECG)

(f) Evaluation for HIV seropositivity to consist of ELISA and, if positive, confirmation by Western blot within the time requirements for autologous apheresis donation for standard of care collections, or within 28 days prior to the leukapheresis procedure for procedures performed on this study. The investigator, in the event of a positive finding, will make appropriate counseling available.

(g) Evaluation for Hepatitis B core antibody (HBcAb), Hepatitis B surface antibody (HBsAb), Hepatitis B surface antigen (HBsAg) and Hepatitis C Virus (HCV) (anti-HCV Antibody) within the time requirements for autologous apheresis donation for standard of care collections, or within 28 days prior to leukapheresis procedure for procedures performed on this study.

(h) β-HCG pregnancy test on all women of child-bearing potential (within 28 days of enrollment)

(i) General Laboratory Tests: The following will be obtained during the screening process:

- ✓ Chemistries: (sodium, potassium, chloride, bicarbonate, BUN, creatinine, glucose, calcium, AST/ ALT, alkaline phosphatase, bilirubin, albumin, total protein) **(**A measured 24 hour urine creatinine clearance test may be performed if the serum creatinine is elevated, and the measured value will be recorded in the CRF and may be used to qualify the subject for study participation)
- ✓ Phosphorus and magnesium
- ✓ CBC with differential
- ✓ C-reactive protein (CRP), ferritin
- ✓ Urinalysis

(j) ECHO, MUGA or Cardiac MRI for LVEF and pericardial effusion assessment

Testing for cardiac performance and ejection fraction to confirm eligibility may be performed anytime within 180 days prior to enrollment.

(k) Disease Evaluation: Disease evaluations will be specific to the subject's location of disease and may include imaging studies: Brain MRI, MRI with and without gadolinium, CT or PET/CT. Disease evaluation of subjects with DIPG will include Standard MR imaging with Sagittal T1 MPRAGE, axial DWI, axial T2 FLAIR, axial T2, and post gadolinium sagittal T1 MPRAGE (with reconstructions) images. The standard MR parameters are listed on the PBTC NIC web page located at http://www.childrenshospital.org/research/centers-departmental-programs/pediatric-brain-tumor-consortium-neuroimaging-center under Neuroimaging Studies/ Specific MR Imaging Sequences- Open PBTC Protocols.

(l) Concomitant Medication

A list of concomitant medications will be captured at the end of screening at the time of confirmation of eligibility. Medications that are stopped during the screening period prior to eligibility sign off do not need to be recorded. The concomitant medication list will be updated at each clinic visit.

(m) Additional Tests:

The PI may order additional tests in some subjects if needed to fully assess clinical status and obtain baseline results.

5.2 LEUKAPHERESIS FOR CELL ACQUISITION:

For subjects who do not have a leukapheresis product stored from a previous procedure that meets requirements for utilization on this study, following enrollment, they will undergo leukapheresis for cell collection. Leukapheresis (apheresis) will be performed according to institutional standards with the goal of obtaining adequate cells to generate the planned cell doses of transduced T cells/kg. Prophylactic intravenous $CaCl_2$ and $MgSO_4$ infusions may be administered by the apheresis clinical team per standard operating procedures. Institutional guidelines will be followed for venous access and apheresis procedures. If PBMCs have been cryopreserved for a different cell therapy or cell collection study, or standard of care, and meet the requirements for this study, they may be used to generate GD2CART on this study if they meet criteria outlined in the IND.

The patient may have a second apheresis collection to obtain the CD3+ cell target and the required minimum number of cells, at the investigator's discretion. If the patient cannot be scheduled for a subsequent apheresis collection and is unable to meet the required number of cells, the patient will be discontinued from the study. If, after enrollment and leukapheresis, the decision is made to not proceed with GD2CART cell manufacturing, the collected apheresis product will be made available to the subject for alternative CAR-T cell production, if requested.

The criteria for initiating leukapheresis is as follows:

- ✓ Subjects must have no evidence of a clinically significant uncontrolled infection prior to leukapheresis.
- ✓ No systemic corticosteroid therapy within 2 weeks prior to leukapheresis.

- ✓ No new signs or symptoms of kidney or liver dysfunction outside eligibility criteria within last 7 days
- ✓ Must not be pregnant

Any changes to eligibility criteria after enrollment will not affect eligibility/enrollment on this study, unless in the physician's estimation they affect the safety of the subject moving forward with the investigational regimen.

The following procedures/requirements will occur on the leukapheresis collection day (+2 days) (unless otherwise specified) and as outlined in the section **9** Study Calendar:

- Vital signs, including blood pressure, heart rate, respiratory rate, oxygen saturation, and temperature
- Weight (day of leukapheresis or day before) including body mass index (BMI) calculation. If BMI > 30, manufactured cell dose will be based on practical weight rather than actual weight (see section **13.3**, Appendix C).
- Labs (to be drawn within 7 days prior to leukapheresis)
    - Chemistry panel (sodium, potassium, chloride, bicarbonate, BUN, creatinine, glucose, calcium, AST and/or ALT, alkaline phosphatase, bilirubin, albumin, total protein)
    - Phosphorus and magnesium
    - CBC with differential
    - C-reactive protein (CRP)
    - Pregnancy test (in child-bearing females, if screening pregnancy test was performed > 7 days prior to leukapheresis)
- Leukapheresis
- Adverse/Serious Adverse Event reporting related to the leukapheresis procedure
- Concomitant medications documentation
- Correlative studies sample collection, as detailed in Appendix G, section **13.8**

5.3 CONDITIONING LYMPHODEPLETION CHEMOTHERAPY REGIMEN

Subjects will receive a conditioning lymphodepletion chemotherapy regimen consisting of cyclophosphamide and fludarabine in order to induce lymphocyte depletion and create an optimal environment for expansion of GD2CART *in vivo*. Subjects will initiate conditioning chemotherapy with cyclophosphamide and fludarabine beginning on Day -4 through Day -2. The 3-day conditioning chemotherapy regimen may be administered in an outpatient setting per investigator's discretion. The dose calculation for the chemotherapy administration (on Days -4 through -2) will be based on the height and weight measured on day -5 or -6. At the investigator's discretion, the timing of the 3-day regimen of conditioning lymphodepletion chemotherapy may be adjusted based on the best interest of the patient.

In order to initiate the conditioning lymphodepletion regimen, there must be 14 days or more than five half-lives from the last dose of standard chemotherapy and at least 2 weeks since the last dose of corticosteroids. The subject must meet the criteria established in Section **5.3.1.**

5.3.1 Eligibility criteria for initiating conditioning lymphodepletion chemotherapy regimen

Routine anti-emetic prophylaxis and treatment should be employed. The criteria for initiating the conditioning regimen is as follows:

Subjects must have:

- no evidence of uncontrolled infection,
- no clinically significant cardiac dysfunction,
- serum creatinine must be < 2 x ULN,
- no change in neurologic status that in the investigator's assessment puts the patient at unacceptable risk of toxicity or is a significant indication of disease progression,
- negative pregnancy test in child-bearing females.
- no systemic corticosteroids for 14 days prior to chemotherapy

Should an event exceed these criteria immediately prior to conditioning chemotherapy, conditioning chemotherapy must be delayed until the event resolves to ≤ Grade 1 or baseline.

5.3.2 Conditioning Lymphodepletion Chemotherapy Regimen Procedures

The procedures for Day -5 to Day -2 are noted in the Study Calendar (see section **9**). Females of childbearing potential must have a negative pregnancy test within 7 days prior to starting chemotherapy.

5.3.2.1 Fludarabine and Cyclosphosphamide Administration

Each subject will receive the lymphodepleting regimen as follows:

Table 1: Conditioning Lymphodepletion Chemotherapy Regimen

Drug	Dose	Days
Cyclophosphamide	500 mg/m^2 per day IV infusion over approximately 60 minutes, daily for 3 days.	-4, -3, -2
Fludarabine	After conclusion of cyclophosphamide infusion, 25 mg/m^2 per day IV infusion in 50 mL of 0.9% sodium chloride over approximately 30 minutes, daily for 3 days.	-4, -3, -2

There will be no dose adjustment of chemotherapy agents for weight considerations or abnormal lab values. If subjects are eligible for the trial, then the full dose of lymphodepletion agents will be administered.

5.3.2.1.1 Supportive Care and Premedications for Lymphodepletion Chemotherapy

- Fluid and electrolyte balance will be maintained as per institutional guidelines and may include as guidance (modifications from these guidelines will be based on investigator's best clinical judgement and will not constitute a protocol deviation): Hydration may be initiated 2 hours prior to cyclophosphamide using 0.45% sodium chloride with 5% dextrose

continuous until discharge. If able to tolerate oral fluids, subject may be discharged after 4 hours of hydration. When discharged to home, include patient instructions: patient to drink 8 ounces/m^2/2hr = xx ounces) every 2 hours and void every 2 hours until bedtime. If unable to tolerate oral fluids, continue D5 0.45%NS (125 mL/m^2/hr) until 8 hours post-hydration complete.

- Mesna may be administered at a dose of 540 mg/m^2 per day by continuous IV infusion at the investigator's discretion. If administered, the first 180 mg/m^2 of mesna that day will be mixed with the cyclophosphamide in an appropriate amount of fluid based on the hydration rate. Immediately upon completion of this initial infusion, mesna at a dose of 360 mg/m^2 in 0.45% sodium chloride and 5% dextrose (or other composition appropriate for the clinical situation) may be infused over 15 minutes or as tolerated given approximately 4 hours (± 30 min) after the start of cyclophosphamide.
- Furosemide: During conditioning lymphodepletion chemotherapy regimen, IV furosemide (0.5 - 1 mg/kg/dose to a maximum of 20 mg per dose) may be administered as needed to maintain normal urine output and fluid balance. Hydration will be adjusted to maintain fluid balance, urine output and urine specific gravity. In addition, serum electrolyte levels will be monitored routinely and IV fluid content including potassium chloride supplementation will be adjusted to maintain normal serum electrolyte levels.
- Anti-emetics

  Routine anti-emetic prophylaxis and treatment should be employed. Corticosteroids may not be used.

5.3.2.1.2 Correlative Samples

Collection of blood, tissue, or cerebrospinal fluid samples for correlative research will occur as outlined in section 13.7 Appendix G: Correlative Sample Schedule. Samples may be omitted if limited by institutional research blood draw volume limits or in the best interest of the subject.

5.3.2.1.3 Adverse Event Reporting

All adverse events (AEs) and serious adverse events (SAEs) will be recorded from start of leukapheresis procedure for subjects undergoing leukapheresis collection on this study, or from the start of lymphodepletion chemotherapy for subjects with a frozen apheresis sample that meets the IND criteria, and conclude 30 days after last study treatment, unless attributable to the gene therapy cell product as described in section 2.6.

5.4 Investigational Agent Administration and Research Procedures

5.4.1 Cell Processing:

Cellular Product: GD2.BB.z.iCasp9-chimeric antigen receptor (GD2-CAR) retroviral transduced autologous peripheral blood lymphocytes (called GD2CART)

Autologous GD2CART will be generated from fresh or cryopreserved PBMCs under GMP conditions using Miltenyi CliniMACS Prodigy® system. Apheresis products from participating subjects will be sent to the manufacturing site through Stanford Bone Marrow Transplant –

Cellular Therapeutics Facility (BMT-CTF); final cell products will be received through and final formulated product distributed by the BMT-CTF.

Any prepared cells not required for the first infusion or for research or regulatory purposes (including sufficient QA retention vials for testing cryopreserved product stability at time of additional infusions) will be cryopreserved by standard techniques and will be made available should the subject be eligible for a second infusion as outlined in section **5.6**. Additional product in excess of that needed for infusion may be utilized for research.

Fresh or cryopreserved peripheral blood mononuclear cells (PBMC) (depending on the timing of apheresis relative to cell culture, subject condition and scheduling availability) will be used for cell manufacturing. Final product will be required to meet standard release criteria as detailed below. All procedures will take place using good manufacturing process (GCP) guidelines.

The release criteria will be based upon analyses of GD2CART and will include:

Table 2: GD2CART Product Rapid Release Criteria

Test	Criteria
Cell viability	≥ 70%
Cell number	within 20% of planned dose level
% CAR+ cells	≥ 10%
Endotoxin	≤ 5 EU/kg
Mycoplasma	≤ 10 CFU/mL
RCR	Negative
Sterility testing	Negative

In the event that a product does not meet one or more of the release criteria but infusion is deemed to be in the best interest of the subject, the investigator, with approval of FDA, IRB and APB and consent of the subject/LAR, may elect to infuse the product, with the exception of dose which will be handled according to section **12.2.1** and does not require additional approvals.

5.4.1.1 Determination of product administration: fresh or cryopreserved

GD2CART will be administered on Day 0. Cells may be cryopreserved after harvest according to Standard Operating Procedures in the manufacturing facility in an appropriately-sized CryoMACS Freezing Bag, or administered fresh (depending on cell culture growth, patient condition, etc). If cells are cryopreserved, infusion will be scheduled when final sterility culture results are available unless the subject's clinical condition requires immediate intervention. In addition, unavoidable delays to the infusion date may include any of the following:

- Subject develops a health condition, significant disease progression or infection prior to cell administration, such that the infusion criteria in Section **5.4.2.1** cannot be met; or
- Schedule conflict arises (patient or patient's family, intervening holidays, within manufacturing facility, etc.)

If it becomes necessary to infuse cells prior to the final sterility culture results, sterility release of the product will be completed based on preliminary reading of the culture results (Day 4-5) and negative gram stain. The Action Plan outlined in section **7.5.4,** will be followed in the unlikely event that a positive sterility or mycoplasma test is obtained after administration of the product.

5.4.2 GD2.BB.z.iCasp9-CAR T cell Infusion

Subjects will be hospitalized to receive treatment with GD2CART, if not previously hospitalized. An Ommaya intraventricular catheter will be inserted, if not already present, for close monitoring of intracranial pressure in subjects with DIPG. Subjects will undergo pre-cell infusion evaluation as per section **5.5.1** and section **9**. Subjects may remain hospitalized for 28 days or may be discharged if treatment-related AEs have resolved to Grade 1 or better at the discretion of the treating physician, but must stay in close proximity to Stanford, for at least 28 days post-infusion.

5.4.2.1 Cell Infusion Criteria

Subjects must meet the following criteria in order for cells to be infused (based on labs obtained within 24 hrs of cell infusion):

5.4.2.1.1 GD2CART must have met release criteria (section **5.4.1**), except cell dose (see section **5.4.3**).

5.4.2.1.2 Subject has no evidence of hemodynamic instability

5.4.2.1.3 Subject has not developed a new requirement for supplemental oxygen therapy

5.4.2.1.4 Subject has not developed symptoms concerning for new, systemic infection, significant disease progression, or any condition that in the opinion of the PI may pose an unacceptable risk to the subject

5.4.2.1.5 There is no evidence of clinically significant cardiac dysfunction, serum creatinine > 2 x ULN, and no new onset acute neurological toxicity > Grade 1.

5.4.2.1.6 No systemic corticosteroid therapy for at least 7 days prior to infusion.

If these criteria are not met, measures will be taken to resolve the underlying condition(s) and the cell infusion must be delayed until the event resolves. Fresh GD2CART cells may be infused within 72 hours of planned infusion or frozen for later administration. If the GD2CART infusion is delayed > 2 weeks, conditioning chemotherapy may be repeated**, at investigator discretion**.

5.4.2.2 Premedications

Subjects will receive levetiracetam 20 mg/kg/dose orally twice a day (maximum dose of 1000 mg/dose) beginning the day before cell infusion (Day -1) unless clinically contraindicated.

Subjects will receive the following medications 30-120 (±10) minutes prior to cell infusion:

- Diphenhydramine: 1 mg/kg/dose PO or IV
- Acetaminophen: 10-15 mg/kg/dose PO

5.4.2.3 Cell Infusion

Cell infusion will be scheduled when final sterility culture results are available unless the subject's disease status/clinical condition requires more urgent intervention. In addition, unavoidable delays to the infusion date may include any of the following:

- Subject develops a health condition or infection prior to cell administration, such that the infusion criteria in section **5.4.2.1** cannot be met; or
- Schedule conflict arises (patient or patient's family, intervening holidays, within the manufacturing facility, etc.)

Cells are delivered to the subject care unit by a staff member from BMT-CTF. Prior to infusion, the cell product identity label is double-checked by two authorized staff (MD or RN), an identification of the product and documentation of administration are entered in the subject's chart, as is done for blood banking protocols. Cell products should NOT be infused unless the product identification matches the subject's identification.

Cells are to be infused intravenously (IV) over 10-30 minutes (or as fast as tolerated based on volume and/or DMSO toxicity) via non-filtered tubing, gently agitating the bag during infusion to prevent cell clumping. After infusing cells, rinse the infusion tubing to ensure complete cell infusion according to institutional procedures. Documentation in the medical record should include the volume of cell infusion, the thaw start/stop time (if cells are cryopreserved), and cell product infusion time start/stop times.

5.4.3 Determination of Cell Dose

Cell dose will be body weight-based according to the following rules:

The dose of GD2CART for each dose cohort will be based on doses calculated using actual body weight, unless subject has BMI > 30, within 7 days of starting cell manufacturing. Subjects with BMI greater than 30 may have doses calculated using ideal body weight (IBW) plus 50% of the difference between actual weight and IBW (see section **13.3,** Appendix C for calculation).

If cell growth limitations preclude administration of the number of cells targeted for the assigned cohort level, the subject will receive as many cells as possible, as the efficacious dose is not yet known, and the subject will be evaluable for feasibility of manufacturing (as per section **12.2.1)** but will not be evaluable for safety in that dose cohort, and may be replaced in the numbers for that dose cohort.

5.4.4 Dose Escalation to Determined MTD/RP2D

There will be a Phase 1 dose-escalation design with three dose cohorts of subjects with DIPG to determine the MTD/RP2D. The number of GD2CART for each dose level cohort described in **Table 1**:

Table 3: GD2CART Dose Levels

Dose Escalation Schedule	
**Dose Level**	**Dose of GD2-CART**
Cohort Level -1	3 x 10^5 transduced T cells/kg (± 20%)
Cohort Level 1	1 x 10^6 transduced T cells/kg (± 20%)
Cohort Level 2	3 x 10^6 transduced T cells/kg (± 20%)
Cohort Level 3	10 x 10^6 transduced T cells/kg (± 20%)

Each dose cohort will initially include a minimum of 3 subjects. We propose to conduct the safety assessment in subjects with DIPG at any age, rather than initially targeting adolescents or adults, or including subjects with spinal DMG for three specific reasons:

1. Pontine disease is rare in adolescents and adults
2. Evaluation of safety in spinal DMG, which occur more commonly in adolescents and young adults, will not inform safety for pontine DMG since much of the risk of toxicity relates to the location of the tumor.
3. Enrollment of adolescents or adults prior to children is not necessary since first, second and third generation CARs targeting GD2, incorporating the same scFv have already been tested in several clinical trials and have demonstrated safety and significant clinical activity[33],[34],[35].

A 28 day safety assessment period will follow infusion of GD2CART of the first subject in each dose cohort. Subsequent subjects in that cohort and subsequent cohorts may be treated after a two week (14 day) safety assessment period. Four weeks (28 days) must elapse after cell infusion in the final subject in each dose cohort to allow for safety assessment of DLTs (as defined in section **5.4.5**) before treating subjects on the next higher dose cohort level. Therefore, treatment in the next higher dose cohort will not proceed to a higher dose level until all subjects have been treated in the prior dose cohort and the last subject treated on the completed dose cohort has been observed for at least 28-days after infusion of GD2CART.

If more than one DLT occurs in the first dose level, (2 out of 6) the dose will be de-escalated to Dose level -1 (3e5 transduced T cells/kg (± 20%)) for safety evaluation. If cell growth limitations preclude administration of the number of cells targeted for the assigned cohort level, the subject will receive as many cells as possible, as the efficacious dose is not yet known, and the subject will be evaluable for feasibility of manufacturing but will not be evaluable for safety in that dose cohort, and may be replaced in the numbers for that dose cohort. If the 3rd dose level is completed without DLT, an MTD may not be determined. This will be considered the ‘highest cell dose’ studied. The RP2D may be the MTD or may be a lower dose chosen by the investigator, in collaboration with the sponsor, based upon feasibility, tolerability and clinical activity. If clinical activity is observed in a dose cohort, despite no observed DLTs, the investigator, in collaboration with the sponsor, may elect to expand that dose cohort to further explore the clinical activity while minimizing the risk of toxicities.

Dose escalation will follow the rules outlined in the **Table 4**.

Table 4: Dose Escalation Rules

Number of Subjects with DLT at a Given Dose Level	Escalation Decision Rule
0 out of 3	Enter up to 3 subjects at the next dose level. If 0 out of 3 occur, dose may escalate.
≥ 2	Dose escalation will be stopped. This dose level will be declared the maximally administered dose (highest dose administered). Up to three (3) additional subjects will be entered at the next lowest dose level if only 3 subjects were treated previously at that dose.
1 out of 3	If DLT develops in the first subject at Dose Level 1, the dose will be de-escalated to Dose Level -1 (3×10^5 transduced T cells/kg (± 20%)), and accrual to Dose Level -1 will proceed to evaluate safety. If DLT develops in any subject at Dose Level -1, accrual will be temporarily stopped while consultation with the IRB, APB and FDA occurs.<br><br>If DLT develops in one of the 3 subjects at Dose Levels 2 or 3, the cohort will be expanded to 6 subjects. If no additional subjects develop DLT, MTD will not have been exceeded and the next dose level can be administered after the 28 day safety assessment period of the last subject at this dose level.<br><br>If 1 or more of this group suffer DLT, then dose escalation is stopped, and this dose is declared the maximally tolerated dose. Up to three (3) additional subjects will be entered at the next lowest dose level if only 3 subjects were treated previously at that dose.
$\leq$1 out of 6 at highest dose level below the maximally administered dose	This is the MTD and is generally the recommended phase 2 dose. At least 6 subjects must be entered at this dose level.

5.4.5 Dose Limiting Toxicity

Adverse events that are considered disease-related (not suspected of relationship to GD2CART) will not be considered dose-limiting toxicities. Only those AEs suspected to be related to the investigational agent, GD2CART will be used in the definition of DLT. Toxicities occurring after initiation of the conditioning lymphodepletion chemotherapy regimen but prior to GD2CART

infusion, will primarily be attributable to the chemotherapy administration or disease, if not extraneous causes. After cell infusion, toxicities will be evaluated for temporal and causal relationship to conditioning lymphodepletion chemotherapy regimen versus cell infusion. Some symptoms may overlap and attribution will not be clearly definable, in which case, toxicities will be attributed as possibly related to both preparative regimen and cell infusion. Toxicities will be attributed to the T cells if: 1) they were NOT present before T cell infusion; OR 2) they increase in Grade in temporal association with the T cell infusion; AND 3) they are not clearly explained by other factors.

Subjects who are fully assessable for DLT are those that have completed the conditioning lymphodepletion chemotherapy regimen and received the GD2CART infusion. Chemotherapy-related toxicities experienced by subjects who are unable to receive GD2CART *will not* be considered evaluable in the definition of DLT.

See section **7.2** Causality for definitions of 'suspected'. The definition of DLT in these studies uses NCI's Common Terminology Criteria for Adverse Events (CTCAEv5.0), the grading system for CRS and neurotoxicity in Appendix B, section **13.2**.

5.4.5.1 Definition of DLT:

Adverse events that are at least possibly related to the investigational agent (GD2CART) with onset within the first 28 days following GD2CART infusion will be considered DLTs as follows:

Hematological Toxicity:

Hematologic toxicities are common after CAR T cell therapies therefore, the following criteria will be considered:

- Subjects with abnormal counts at baseline will not be evaluable for hematological toxicity
- Subjects evaluable for hematologic toxicity: Any grade 4 neutropenia or thrombocytopenia lasting > 14 days despite best supportive care is a DLT.

Non-hematologic Toxicity:

- Any grade 5 toxicity
- CRS toxicity Grade 4 in severity, or Grade 3 in severity for greater than 7 days (see Appendix B, section **13.2**.)
- Infusion reactions ≥ Grade 2 in severity lasting more than 24 hours despite standard supportive care.
- Grade 4 neurotoxicity, (as defined in CTCAEv5 as life threatening, urgent intervention needed) for greater than 96 hours (see Appendix B, section **13.2)**.
- Any new Grade 3 neurotoxicity (not present at baseline and excluding cranial neuropathies and ataxia, which are common in this disease) lasting longer than 28 days.
- Any other Grade 3 or greater, non-hematological toxicity which is possibly, probably, or definitely attributed to investigational product (GD2CART) with exclusion of transient, laboratory abnormalities without clinical consequence, and occurring within 28 days of

investigational product administration will be considered a DLT with the following **exceptions**:

- Grade 3 neurotoxicity
- Grade 3 diarrhea that resolves to $\leq$ grade 2 within 4 days;
- Grade 3 low electrolyte levels that are correctable and asymptomatic, Grade 3 hypoalbuminemia;
    - Hypocalcemia toxicity grade should be assigned based on the calcium level corrected for degree of hypoalbuminemia according to the following formula: For every albumin decrease of 1 gm/dL a total calcium increase of 0.2 mmol/L is to be made;
- Abnormal coagulation parameters in subjects on anticoagulant therapy or with pre-existing coagulopathy;
- Grade 3 transaminase, alkaline phosphatase, bilirubin or other liver function test elevation, provided there is resolution to $\leq$ grade 2 or baseline within 14 days. Grade 4 transaminitis lasting < 72 hours will also not be considered DLT;
- Grade 3 or 4 fever lasting $\leq$ 14 days, as this is common and expected with CAR therapy and may be a biomarker for an effective immunotherapeutic regimen;
- Grade 3 or 4 infection or neutropenic fever unless infection is not considered likely related to cyclophosphamide/fludarabine and relationship to GD2CART is suspected (Note: Grade 4 infection uncontrolled for > 7 days will be considered DLT);
- Grade 3 nausea and/or anorexia.

As noted in section **2.5.4** CRS will be graded according to the ASTCT CRS Consensus Grading[52] (See Appendix B, section **13.2**).

5.4.5.2 Definition of Maximal Tolerated Dose (MTD)

The MTD is a dose level immediately below the level at which the enrollment is stopped due to DLTs, as explained specifically below:

- If **more than one subject** in the first three subjects in a dose level experience DLT as defined above, MTD will have been exceeded.
- If DLT develops in **one** of the 3 subjects included in a cohort, the cohort will be then expanded up to six:
- If 2 or more of these 6 included subjects develop DLT, the MTD will have been exceeded.

If no additional subject develops a DLT, the MTD will not have been exceeded and the next dose level can be administered after the 28-day safety assessment period of the last subject at the dose level.

If MTD is exceeded at any dose level, three subjects will be added to the immediate lower dose level, unless it has been previously expanded to six. If less than 2 of 6 subjects develop DLT at that level, it will be defined as the MTD.

5.4.6 Dose Expansion Cohort

To gain further experience with the safety, feasibility and clinical activity of the GD2CART in subjects with DIPG and subjects with spinal H3 K27M-mutant DMG, the RP2D cohort will expand in two groups to include a total of 20 subjects with DIPG and 10 subjects with DMG treated, including those subjects treated at the RP2D during the dose escalation phase as described in section **4.1.2**. The main goal for the dose expansion portion of the trial is to collect data about safety and tolerability from a larger group of patients receiving treatment at the RP2D. A secondary goal is to collect data about efficacy, such as overall survival, in order to compute point, interval, and quantile estimates in subjects with DIPG as per section **12.4**. The number of subjects with spinal H3 K27M-mutant DMG is not anticipated to be significant, hence the clinical activity (OS) in up to 10 subjects treated at the RP2D will be reported separately and descriptively.

5.5 Evaluations and Follow UP

Subjects will be hospitalized prior to receipt of GD2CART, if not before administration of conditioning lymphodepletion chemotherapy regimen. Subjects will be monitored closely inpatient or in close proximity to Stanford for at least 28 days, at the investigator's discretion. For all patients, neurooncologists, neurosurgeons and neurointensivists will be consulted and involved in every case and neurointensive care provided for management of neurologic deterioration and/or increase in intracranial pressure.

During this post cell infusion period, procedures will be completed at the following time points as outlined in the Section **9** Study Calendar:

5.5.1 Evaluation Prior to Cell infusion (within 24 hours)

Prior to cell infusion subjects will undergo safety evaluations with general laboratory tests, physical exam, neurologic exam, vital signs, adverse event collection, concomitant medication monitoring, and correlative sample collection.

Once, any time between enrollment and start of initial cell infusion, a blood sample for replication competent lentivirus (RCL) testing will be collected and sent to Indiana Gene Therapy Testing Laboratory (IU GTTL), as described in section **13.4**.

Please refer to the section **9** Study Calendar for a list of all procedures, including the following:

- Measurement of ICP via Ommaya Reservoir (baseline) prior to cell infusion in patients with DIPG
- Disease evaluation with Brain MRI will be performed in subjects with DIPG within 7 days prior to cell infusion (at the investigator's discretion).

5.5.2 Required monitoring during cell infusion:

Monitoring will include vital signs (blood pressure, heart rate, respiratory rate, oxygen saturation, and temperature) prior to infusion, within 15 minutes after start of infusion (± 10 min), and then 30 (± 10 min), 60 (± 10 min), and 120 (± 10 min) minutes after infusion. ICP monitoring will be performed in the ICU and emergency measures for treatment of increased intracranial pressure will be available.

- ✓ Supplemental oxygen will be available at the bedside.
- ✓ If an allergic or other acute reaction occurs, studies appropriate for investigation of a transfusion reaction will be performed (urinalysis, CBC, Coomb's test).
- ✓ Acute reactions or increased intracranial pressure will be treated according to institutional standards of care, while avoiding steroid administration if possible.

5.5.3 Evaluations Post cell Infusion Day 1 to Day 27

After completing GD2CART infusion all subjects will be followed in the post treatment assessment period. Counting from Day 0 (GD2CART infusion), subjects will undergo the following evaluations:

- Day 1 to Day 14: Daily during hospitalization or 5 times per week if outpatient (with no more than 48 hours between evaluations)
- Day 15 to Day 27, Daily during hospitalization or twice per week if outpatient (± 4 days)
- Any time the subject's clinical condition changes, clinical best practices will be used to determine the frequency of evaluation

Please refer to the section **9** Study Calendar for a list of all procedures at each visit.

Procedures will include safety evaluations with general laboratory tests, targeted physical exams (as clinically indicated), neurologic exams with measurement of ICP via Ommaya Reservoir in subjects with DIPG, adverse event collection, concomitant medication monitoring, and correlative sample collection. Vital sign monitoring (including blood pressure, heart rate, oxygen saturation, respiratory rate, and temperature) should be performed every shift (± 2 hours) or as clinically indicated during hospitalization and with each visit after discharge. Neurotoxicity evaluations in the form of ICE score/ CAPD evaluations should be performed every shift (± 2 hours) and with any change in neurologic functioning during the initial hospitalization for cell infusion.

- If any evidence of increased intracranial pressure or clinical deterioration suspected due to neurologic compromise, patient will be transferred to the ICU (see section **13.2.3.5** Appendix B) or re-admitted to the hospital if previously discharged.

- Disease evaluations will be specific to the subject's location of disease and may include imaging studies as follows:

  - Subjects with DIPG will undergo brain MRI on D7, D14, and D21. If clinical condition post-infusion prevents MRI, a bedside CT will be obtained on those days. Standard MR imaging will include Sagittal T1 MPRAGE, axial DWI, axial T2 FLAIR, axial T2, and post gadolinium sagittal T1 MPRAGE (with reconstructions) images. The standard MR parameters are listed on the PBTC NIC web page located at http://www.childrenshospital.org/research/centers-departmental-programs/pediatric-brain-tumor-consortium-neuroimaging-center under Neuroimaging Studies/ Specific MR Imaging Sequences- Open PBTC Protocols.

  - Subjects with spinal DMG will undergo MRI with and without gadolinium and/or CT or PET/CT

Monitoring of CRP, ferritin, and LDH (only if LDH is elevated at baseline) levels may assist with the diagnosis and define the clinical course in regards to CRS/neurotoxicity. It is therefore,

recommended that CRP and LDH (if elevated at baseline) be monitored when lab specimens are drawn starting at Day 0 and continuing through hospitalization. In addition, lactate can be monitored as clinically indicated. The above schedule may be modified as needed based on the subject's clinical condition and the investigator's medical judgement.

5.5.4 Evaluation on Day 28 (± 4 days)

Day 28 (± 4 days) will be the first disease evaluation post cell infusion. Evaluations will include general laboratory tests, full physical exam, neurologic exam, performance status, weight, vital signs, adverse event collection, concomitant medication monitoring, disease evaluation, and correlative sample collection as per section 9.

5.5.4.1 Evaluations of subjects who Do NOT receive GD2CART

The following procedures/assessments will be completed for subjects who are enrolled but do not receive GD2CART, at the time points outlined in the Section 9 Study Calendar until disease progression and/or toxicities attributable to the study participation resolve to ≤ Grade 1 or stabilize:

- Disease assessment per standard of care
- Adverse/Serious Adverse Event reporting and concomitant medication documentation until 30 days after last procedure (e.g., leukapheresis, conditioning chemotherapy).

Should the subject fail to return to the clinic for a scheduled protocol specific visit, sites will need to make 2 attempts by a combination of telephone and mail to contact the subject. Sites must document both attempts to contact the subject. If a subject does not respond within 1 month after the second contact the subject will be considered lost to follow-up and no additional contact will be required.

5.5.5 Schedule and Procedures For Post Treatment Assessment Period (after Day 28) after GD2CART

After Day 28, subjects who received GD2CART will return to the clinic at the following intervals, <u>unless</u> there is disease progression and/or alternative disease therapy is started, at which time the subject will proceed to long term follow up (see section 5.5.6):

- Month 2 (± 2 weeks)
- Month 3 (± 2 weeks)
- Month 6 (± 4 weeks)
- Month 9 (± 4 weeks)
- Month 12 (± 4 weeks)

Evaluations of disease status in subjects who have not progressed after GD2CART will be conducted at Stanford for the first 24 months. Subsequent disease evaluations may be performed by the outside medical facility/care provider as per standard of care. If subject's disease has not progressed by Month 24, disease assessments will continue to be performed per standard of care.

Should a subject fail to return to the clinic for a scheduled protocol specific visit, sites will need to make 2 attempts by a combination of telephone and mail to contact the subject. Sites must document both attempts to contact the subject. If a subject does not respond within 1 month after

the second contact the subject will be considered lost to follow-up and no additional contact will be required.

The procedures to be completed in subjects who have received GD2CART cells is outlined in the Section **9** Study Calendar and will include general laboratory tests, physical exam, neurologic exam, performance status, weight, vital signs, adverse event collection, concomitant medication monitoring, disease evaluation, and correlative sample collection as per section **13.7**.

5.5.6 Long Term Follow-up Period

Subject who complete month 24 visit without developing disease progression, will be followed as per section **5.5.6.1**.

Subjects who:

- did not respond to treatment (i.e., did not achieve a CR or PR), or
- progress following a response and is either not eligible for re-treatment or chooses not to pursue re-treatment, or
- proceed to alternative disease therapy,

will undergo follow up as per section **5.5.6.2**.

All subjects who received CAR T cells, regardless of disease status or alternative therapy options, will undergo long term gene therapy follow up as per section **5.5.6.3**.

Should a subject fail to return to the clinic or respond to contact for a scheduled protocol specific visit/contact, sites will need to make 2 attempts by a combination of telephone and mail to contact the subject. Sites must document both attempts to contact the subject. If a subject does not respond within 1 month after the second contact the subject will be considered lost to follow-up and no additional contact will be required.

5.5.6.1 Long Term Follow-up Schedule and Evaluations for subjects who have **not** developed progressive disease (have an ongoing response)

5.5.6.1.1 Schedule

Subjects who received GD2CART and have NOT developed progressive disease will begin the long term follow-up period after they have completed Month 24 visit:

- Every 6 – 12 months (± 2 months) for 5 years
- Beginning with year 6 (± 3 months), subjects will return to the clinic or be contacted by phone, e-mail or mail with questionnaire, one time annually for up to 15 years as per section **5.5.6.3**.

5.5.6.1.2 Evaluations

The procedures in section **9** Study Calendar will be completed for all subjects who received GD2CART, who have NOT developed progressive disease, at the time points outlined above and will include general laboratory tests, physical exam, neurologic exam, performance status, and disease evaluation.

Evaluations of disease status in subjects who have not progressed after GD2CART will be conducted at Stanford for the first 24 months. Subsequent disease evaluations may be performed by the outside medical facility/care provider as per standard of care. Once a subject develops disease progression, no further disease evaluations are required on this study. All subjects will be followed for long term gene therapy follow up as well as survival and subsequent therapies.

5.5.6.2 Long Term Follow-up Schedule for subjects who develop progressive disease, are not eligible or decline retreatment, or proceed to alternative disease-directed therapy

5.5.6.2.1 Schedule

Subjects who received GD2CART who develop progressive disease, are not eligible or decline retreatment, or proceed to alternative disease-directed therapy will begin the long term follow-up period as outlined below:

- Month 3, 6 and 12 for the first year.
- Every 12 months (± 2 months) for 5 years
- Beginning with year 6 (± 3 months), subjects will return to the clinic or be contacted by phone, e-mail or mail with questionnaire, one time annually for up to 15 years after the date of cell infusion as per section **5.5.6.3**.

5.5.6.2.2 Evaluations

The following procedures will be completed at the time points outlined above and section **9** Study Calendar:

- Physical exam, vital signs and performance status for year 1 through 5 (may be performed by the local physician)
- Survival and subsequent therapies
- **Annual** evaluations in the first 5 years should specifically document any new malignancies, new incidence or exacerbation of a pre-existing neurologic disorder, new incidence or exacerbation of a prior rheumatologic or other autoimmune disorder, new incidence of a hematologic disorder; and other factors that may be relevant to the feasibility and scientific value of the long-term follow up observations (i.e. exposure to other cancer causing agents).

5.5.6.3 Safety Assessment Testing for Replication Competent Retrovirus in Retroviral Vector Based Gene Therapy Products during Subject Follow up

In compliance with OSP and FDA's ***Guidance for Industry: Gene Therapy Clinical Trials – Observing Participants for Delayed Adverse Events (2006)***, subjects who have received at least one dose of a genetically engineered cellular therapy will be evaluated for long term safety and occurrence of adverse events according to the requirements established by FDA guidance and the NIH Guidelines for Research Involving Recombinant or Synthetic Nucleic Acid Molecules (NIH Guidelines) (2016).

- Long term follow up (**year 1 - 5**) for gene therapy related toxicities
    - Targeted Physical Exam and History –

Gene Therapy annual evaluations should specifically document any new malignancies, new incidence or exacerbation of a pre-existing neurologic disorder, new incidence or exacerbation of a prior rheumatologic or other autoimmune disorder, new incidence of a hematologic disorder; and other factors that may be relevant to the feasibility and scientific value of the long-term follow up observations (i.e. exposure to other cancer causing agents).

- Targeted Adverse/Serious Adverse Event reporting including neurological, hematological, infections, autoimmune disorders, and secondary malignancies
- Targeted concomitant medication documentation including immunosuppressive drugs, anti-infectives, and vaccinations
- Blood Samples (archived) for RCR

  RCR blood samples must be collected at Month 3 (±1 month), Month 6 (± 1 month) and Month 12 (± 2 months) and sent to Indiana University Gene Therapy Testing Laboratory.

  After month 12, RCR blood samples will be collected annually for the next 4 years (for a total of 5 years) and banked if all RCR tests in first year were negative, with a brief clinical history conducted annually. If any post-treatment samples are positive, further analysis of the RCR and more extensive subject follow-up will be undertaken, in consultation with the FDA.

  If a subject dies or develops neoplasms during this follow up, efforts will be made to assay a biopsy sample for RCR.

  If any subject has more than 5% persistence of gene-transduced cells at Month 6 using 1A7, an anti-idiotype antibody that recognized the GD2CART, samples will be studied for clonality of persisting gene transduced cells. Such techniques may include analysis of BV chain expression, T cell cloning or LAM-PCR. If a predominant or monoclonal T cell clone derived from gene transduced cells is identified during the follow-up, the integration site and sequence will be identified and subsequently analyzed against human genome database to determine whether the sequences are associated with any known human cancers. If a predominant integration site is observed, the T cell cloning or LAM-PCR test will be used at an interval of no more than 3 months after the first observation to see if the clone persists or is transient. In all instances where monoclonality is persistent and particularly in instances where there is expansion of the clone, regardless of whether or not the sequence is known to be associated with a known human cancer, the subject should be monitored closely for signs of malignancy, so that treatment, if available, may be initiated early.

- Long term follow up (**year 6-15**) for gene therapy related toxicities

  Once a subject has completed 5 years of follow up without evidence of delayed adverse events secondary to genetically engineered cellular therapy product, the following evaluation will be conducted annually for the subsequent 10 years (± 2 months):

The subject will be contacted (in clinic or via phone or written questionnaire) to evaluate for development of delayed adverse events (See questionnaire in section **13.5**, Appendix E).

- If vector modified cells were detected in the blood during the previous visit, then blood for persistence of vector modified cells will be collected and tested until negative.
- Subjects are requested to inform the study research team of any changes to e-mail (if consented to use e-mail), phone, and address. E-mails will be sent to subjects via the Secure Email to ensure securely and confidentially over an SSL/encrypted connection.
- Distribution and collection of questionnaires

A draft letter to subject's primary doctor is provided in section **13.6,** Appendix F. This letter should be sent to the local health care provider via mail, fax or e-mail at the time the subject is referred back to the care of the local physician or with any change in primary doctors. E-mails will be sent to subjects via the Secure Email to ensure securely and confidentially over an SSL/encrypted connection.

- Subjects will be sent a request for information (**section 13.5**, Appendix E) and questionnaire annually.
  - If there is no response within 1 month, the study coordinator will follow up with a telephone call to request the information. The questionnaire may be completed with the subject responses over the phone at this time.
  - If there is no response to the telephone call, the letter and questionnaire (section **13.5,** Appendix E) should be sent to the subject via FedEx, signature required.
  - Each attempt to contact the subject and outcome must be documented in the medical record.

5.6 OPTION FOR ADDITIONAL DOSE(S) OF GD2CART

On the day the cell product is harvested, remaining cells that have been produced above and beyond the number of cells needed for a subject's dose level, research aliquot and QA retention vials will be cryopreserved using standard techniques. If an adequate number of additional cells are available to meet the numbers for an additional dose (with corresponding QA retention vials), the subject may be offered the option of retreatment (additional infusion of GD2CART including preparative chemotherapy regimen, toxicity assessment and research blood sampling) if the following criteria are met:

5.6.1 Eligibility Criteria for subsequent cell infusions:

✓ Cell infusion of GD2CART has been deemed safe at the targeted dose and has been completed without 2 subjects in a dose cohort experiencing DLTs.

✓ Response to previous infusion. Subjects who had a PR or SD with clinical benefit may elect to receive another infusion of cells. Subjects that initially had a CR may only

receive a second dose if evaluable disease recurs. Clinical benefit is indicated by an improvement in the subject's health status.

✓ At least 30 days have passed since the previous cell infusion.

✓ Circulating levels of GD2CART must be < 5% by flow cytometry.

✓ Any toxicity (regardless of causality) after the previous GD2CART cell infusion must resolve such that subjects meet all the initial eligibility criteria for major organ function and therapy washout as outlined in **3.1.**

✓ An adequate number of cryopreserved GD2CART and QA retention vials or an adequate number of apheresis product sufficient to generate an additional dose of GD2CART must be available.

The cell dose (based on CAR transduced cells) for the second infusion shall not be greater than the current dose level completed or the safe dose if this has been determined.

As a rule, subjects who incurred DLT after receiving the first cell infusion will not be eligible to receive additional cell infusions unless IRB, APB and FDA approval is granted on a case by case basis.

5.6.2 Procedures for Additional Doses

5.6.2.1 Subjects may receive additional antineoplastic and lymphodepleting chemotherapy prior to the second infusion of GD2CART. At the investigator's discretion, an intensified lymphodepleting chemotherapy regimen (fludarabine 30 mg/m^2 per day IV for 4 days and cyclophosphamide 600 mg/m^2 per day IV for 2 days) may be administered. This dosing has been used successfully at the NCI Pediatric Oncology Branch without increased toxicity (Personal communication, N. Shah, Pediatric Oncology Branch, National Cancer Institute, NIH). Colleagues' experience at Memorial Sloan Kettering (using CD19-28z CAR) using single-agent Cyclophosphamide (up to 3 Grams over 2 days) have reported significantly better responses with high dose (3G Cyclophosphamide - responses of 94%) as compared to low dose (1.5G Cyclophosphamide - responses of 38%) (Personal communication).

5.6.2.2 After QA retention vial samples have been tested for cell viability and cell number, meeting the original release criteria for cell viability, the cellular product will be thawed and administered IV over approximately 10-30 min or as tolerated based on volume status and/or DMSO toxicity.

5.6.2.3 Any subject who receives subsequent doses of GD2CART will NOT be evaluable for toxicity purposes of this study as they will be beyond the 28 day observation period for DLTs after their first cell infusion. However post-infusion monitoring will be the same as for the 1st infusion and all toxicities, including secondary reactions, will be recorded and reported. If two or more subjects develop Grade 4 toxicity at any time following the second GD2CART infusion that is felt to be possibly, probably or likely related to the CAR T cells, then second infusions will be paused pending discussion with the

FDA, IRB and APB regarding continuing second infusions as part of the experimental regimen.

5.6.2.4 Gene Therapy Follow Up

If a subject receives more than one cellular product, the timing of gene therapy long term evaluations is restarted with each subsequent gene therapy administration. For example, a subject receives gene therapy and has undergone long term follow up for 1.5 years, develops progressive disease and is treated with a second gene therapy product. That subject will begin the blood sample collection at 3, 6 and 12 months (± 1 month) post cell administration and continue for a total of 15 years of follow up from the LAST gene therapy administration.

6 INVESTIGATIONAL AGENT AND COMMERCIAL DRUG INFORMATION

6.1 GD2.BB.Z.ICASP9-CHIMERIC ANTIGEN RECEPTOR (GD2-CAR) RETROVIRAL TRANSDUCED AUTOLOGOUS PERIPHERAL BLOOD LYMPHOCYTES (GD2CART); FOLLOWING FLUDARABINE AND CYCLOPHOSPHAMIDE

6.1.1 Description

Cell therapy production will be conducted according to the SOPs in the manufacturing facility and must meet the requirements for a cGMP facility. The GD2.BB.z.iCasp9 replication incompetent retroviral vector has been manufactured by Bellicum Pharmaceuticals. All procedures will take place using good manufacturing process guidelines.

6.1.2 Stability:

Stability testing is ongoing. In the event a subject elects to receive retreatment, and adequate numbers of cells exist, stability will be evaluated prior to lymphodepleting chemotherapy and product thaw by cell viability analysis of a QA retention vial.

6.1.3 Administration

Guidance on administration of GD2CART is specified in section 5.4.2.

6.1.4 Toxicities

In addition to standard transfusion types of reactions (chills, fever, rigors), the toxicities specific to CAR T cell therapy are described in detail in section 2.5. Every effort will be made to mitigate the risk of these toxicities with the intensive monitoring plans outlined in section 5 and the supportive care measures outlined in section 4.2. With growing experience with CAR therapy world-wide, the risk of severe neurotoxicity is increasingly better recognized—most notable with the recent occurrences of fatal neurotoxicity. With the incorporation of mandatory anti-seizure prophylaxis and prospective monitoring of neurologic function, we plan to rigorously monitor and evaluate for any neurotoxicity, with a plan to treat neurotoxicity aggressively.

Therapeutic interventions will generally include hypertonic saline, CSF removal via the Ommaya reservoir, and mannitol and dexamethasone. If these measures fail to control the increased ICP,

consideration will be given to administering dasatinib to inhibit CAR function[50] and/or administration of AP1903 to permanently ablate the CAR T cells. The AP1903 drug will be administered as a suicide switch to deplete the genetically engineered cells. Avastin may also be utilized as it has shown some efficacy in the setting of tumor associated edema. An algorithm that provides an systematic approach to neurotoxicity management for patients enrolled on this trial is shown in in section 13.2.3.5. The algorithm will serve as a guideline but is not meant to supercede clinical judgment in the management of neurotoxicity on this trial, and deviations from the algorithm will not be considered protocol deviations.

6.2 FLUDARABINE

6.2.1 Description:

(Please refer to package insert for complete product Information) Fludarabine phosphate is a synthetic purine nucleoside that differs from physiologic nucleosides in that the sugar moiety is arabinose instead of ribose or deoxyribose. Fludarabine is a purine antagonist antimetabolite.

6.2.2 How Supplied:

It will be purchased by the institution's Pharmacy Department from commercial sources. Fludarabine is supplied in a 50 mg vial as a fludarabine phosphate powder in the form of a white, lyophilized solid cake.

6.2.3 Stability:

Following reconstitution with 2 mL of sterile water for injection to a concentration of 25 mg/ml, the solution has a pH of 7.7. The fludarabine powder is stable for at least 18 months at 2-8^{0}C; when reconstituted, fludarabine is stable for at least 16 days at room temperature. Because no preservative is present, reconstituted fludarabine will typically be administered within 8 hours. Specialized references should be consulted for specific compatibility information. Fludarabine is dephosphorylated in serum, transported intracellularly and converted to the nucleotide fludarabine triphosphate; this 2-fluoro-ara-ATP molecule is thought to be required for the drug's cytotoxic effects. Fludarabine inhibits DNA polymerase, ribonucleotide reductase, DNA primase, and may interfere with chain elongation, and RNA and protein synthesis.

6.2.4 Storage:

Intact vials should be stored refrigerated (2-8°C).

6.2.5 Administration:

Fludarabine is administered as an IV infusion in an appropriate solution over 30 minutes as described in Section 5.3.

6.2.6 Toxicities:

The primary side effect is myelosuppression; however, thrombocytopenia is responsible for most cases of severe and life-threatening hematologic toxicity. Serious opportunistic infections have occurred in subjects treated with fludarabine. Hemolytic anemia has been reported after one or more courses of fludarabine with or without a prior history of a positive Coomb's test; fatal

hemolytic anemia has been reported. In addition, bone marrow fibrosis has been observed after fludarabine therapy. Other common adverse effects include malaise, fever, chills, fatigue, anorexia, nausea and vomiting, and weakness. Irreversible and potentially fatal central nervous system toxicity in the form of progressive encephalopathy, blindness, and coma is only rarely observed at the currently administered doses of fludarabine. More common neurologic side effects at the current doses of fludarabine include weakness, pain, malaise, fatigue, paresthesia, visual or hearing disturbances, and sleep disorders. Adverse respiratory effects of fludarabine include cough, dyspnea, allergic or idiopathic interstitial pneumonitis. Tumor lysis syndrome has been rarely observed in fludarabine treatment of CLL.

6.3 CYCLOPHOSPHAMIDE

(Refer to FDA-approved package insert for complete product information)

6.3.1 Description:

Cyclophosphamide is a nitrogen mustard-derivative alkylating agent. Following conversion to active metabolites in the liver, cyclophosphamide functions as an alkyating agent; the drug also possesses potent immunosuppressive activity. The serum half-life after IV administration ranges from 3-12 hours; the drug and/or its metabolites can be detected in the serum for up to 72 hours after administration.

6.3.2 How Supplied:

Cyclophosphamide will be obtained from commercially available sources by the institution's Pharmacy Department.

6.3.3 Stability:

Following reconstitution as directed with sterile water for injection, cyclophosphamide is stable for 24 hours at room temperature or 6 days when kept at 2-8^{0}C.

6.3.4 Administration:

It will be diluted in an appropriate solution and infused as described in Section **5.3.**

6.3.5 Toxicities:

Hematologic toxicity occurring with cyclophosphamide usually includes leukopenia and thrombocytopenia. Anorexia, nausea and vomiting, rash and alopecia occur, especially after high-dose cyclophosphamide; diarrhea, hemorrhagic colitis, infertility, and mucosal and oral ulceration have been reported. Sterile hemorrhagic cystitis occurs in about 20% of subjects; severity can range from microscopic hematuria to extensive cystitis with bladder fibrosis. Although the incidence of hemorrhagic cystitis associated with cyclophosphamide appears to be lower than that associated with ifosfamide, mesna (sodium 2-mercaptoethanesulfonate) has been used prophylactically as a uroprotective agent in subjects receiving cyclophosphamide. Prophylactic mesna is not effective in preventing hemorrhagic cystitis in all subjects. Subjects who receive high dose cyclophosphamide may develop interstitial pulmonary fibrosis, which can be fatal. Hyperuricemia due to rapid cellular destruction may occur, particularly in subjects with hematologic malignancy. Hyperuricemia may be minimized by adequate hydration, alkalinization

of the urine, and/or administration of allopurinol. If allopurinol is administered, subjects should be watched closely for cyclophosphamide toxicity (due to allopurinal induction of hepatic microsomal enzymes). At high doses, cyclophosphamide can result in a syndrome of inappropriate antidiuretic hormone secretion; hyponatremia with progressive weight gain without edema occurs. At high doses, cyclophosphamide can result in cardiotoxicity. Deaths have occurred from diffuse hemorrhagic myocardial necrosis and from a syndrome of acute myopericarditis; in such cases, congestive heart failure may occur within a few days of the first dose. Other consequences of cyclophosphamide cardiotoxicity include arrhythmias, potentially irreversible cardiomyopathy, and pericarditis. Other reported adverse effects of cyclophosphamide include headache, dizziness, and myxedema; faintness, facial flushing, and diaphoresis have occurred following IV administration. Mesna (sodium 2-mercaptoethanesulphonate; given by IV injection) is a synthetic sulfhydryl compound that can chemically interact with urotoxic metabolites of cyclophosphamide (acrolein and 4-hydroxycyclophosphamide) to decrease the incidence and severity of hemorrhagic cystitis, but is not a required premedication for this study.

6.4 ACETAMINOPHEN (TYLENOL):

Will be given as a pre-medication. This agent will be provided by the institution’s pharmacy. Please refer to the package insert for complete pharmaceutical information on this product.

6.5 DIPHENHYDRAMINE (BENADRYL):

Will be given as a pre-medication IV or orally. This agent will be provided by the institution’s Pharmacy Department. Please refer to the package insert for complete pharmaceutical information on this product.

6.6 ANTIMICROBIAL PROPHYLAXIS

Subjects will receive appropriate antimicrobial prophylaxis (e.g., Bactrim for PCP and acyclovir for HSV and VZV prophylaxis) during fludarabine/cyclophosphamide treatment and for a minimum of 6 months following treatment. This agent will be provided by the institution’s Pharmacy Department. Please refer to the package insert for complete pharmaceutical information on this product.

6.7 LEVETIRACETAM (KEPPRA):

Subjects will receive levetiracetam 20 mg/kg/dose orally twice a day (maximum dose 1000 mg/dose) beginning the day before cell infusion (Day -1). This dose may be adjusted based on symptoms of neurotoxicity and/or with recommendations of neurologic consult. Levetiracetam should continue through day 28 once neurotoxicity develops. This agent will be provided by the institution’s Pharmacy Department. Please refer to the package insert for complete pharmaceutical information on this product.

6.8 MESNA

Pediatric subjects may be given mesna prophylactically as an uroprotective agent during and after the cyclophosphamide component of the conditioning lymphodepletion chemotherapy as described in Section **5.3.** This agent will be provided by the institution’s pharmacy. Please refer to the package insert for complete pharmaceutical information on this product.

6.9 AP1903 DIMERIZING AGENT

6.9.1 Description

AP1903, also known as Rimiducid, is a lipid-permeable tacrolimus analogue with homodimerizing activity. AP1903 homodimerizes an analogue of human protein FKBP12 (Fv) which contains a single acid substitution (Phe36Val) so that AP1903 binds to wild-type FKBP12 with 1000-fold lower affinity. This agent is used to homodimerize the Fv-containing drug-binding domains of genetically engineered receptors. Upon binding of AP1903 to the engineered FKBP12, caspase 9 activation ensues leading to endogenous caspase 3 activation and cells undergo apoptosis, beginning 30 minutes after infusion and peaking at 3 hours.

GD2CART contain the 'suicide' gene iCasp9; in the presence of AP1903, the iCasp9 promolecule dimerizes and activates the intrinsic apoptotic pathway, leading to cell death.

6.9.2 How Supplied:

AP1903 is packaged in 10 ml type I glass vials. The contents of each vial is composed of the labeled content (40 mg) of AP1903 drug substance dissolved in a sterile, endotoxin free, 24% Solutol HS 15/Water for Injection solution at an AP1903 concentration of 5 mg/ml and at pH of 5.0-7.5. Each vial is stoppered with a Teflon coated serum stopper and a yellow flip-off seal. The vial contains no preservatives.

AP1903 will be supplied by Bellicum Pharmaceuticals, who have provided authorization to cross reference to their IND.

6.9.3 Stability

Stable for at least 24 months when stored at 2-8°C.

6.9.4 Preparation and Administration

AP1903 should be warmed to room temperature prior to dilution. AP1903 is incompatible with materials containing plasticizer or DEHP and materials sterilized with ethylene oxide. The appropriate dose of AP1903 should be added to a 100 ml DEHP-free infusion bag of 0.9% Sodium Chloride Injection. Children 30 kg and below may have the dose diluted in 50 ml of 0.9% Sodium Chloride Injection if less fluid is desired. The final preparation should be stored at room temperature until administered. Since the final product contains no preservatives, the infusion should be completed within 8 hours of preparation. While prolonged direct exposure to light of the AP1903 drug is not encouraged, there is no need to protect from light after preparation prior to administration.

Premedicate with acetaminophen and diphenhydramine using standard dosing 15-30 minutes prior to AP1903. The diluted infusion should be administered at a dose of 0.4 mg/kg over 2 hours using a DEHP-free solution set that has been provided by Bellicum Pharmaceuticals. At the end of the infusion the line should be flushed with 0.9% Sodium Chloride Inj or 5% Dextrose Injection or any standard combination of these two fluids (e.g., 5% Dexrose in 0.45% Sodium Chloride Injection).

6.9.5 Toxicity:

None expected, the no observed effect level in dogs was 1000mg/kg, which is much beyond the prescribed 0.4 mg/kg dose. Urticaria and flushing observed in one patient, which did not occur with subsequent AP1903 administration after premedication. In the same trial, one patient experienced a cytokine release reaction after receiving AP1903 following dendritic cell infusions.

7 ADVERSE EVENTS AND REPORTING PROCEDURES

7.1 Potential Adverse Events

7.1.1 GD2.BB.z.iCasp9-CAR T cells

Because these cells have been previously administered in only a small number of humans, there may be unanticipated adverse events.

Section **2.4** and section **4.2.** discuss the potential risks of this investigational therapy based on previous studies with this or similar preparative regimens or cell products, including the risks of chemotherapy, risk of autoimmunity, risk of neurotoxicity, risk of the gene therapy component, and risk of cytokine storm. Transient, reversible mild effects have been observed with the administration of fresh cells, including chills, fever, rigors, diaphoresis, anorexia, nausea, diarrhea, headache and myalgias. General guidance for treatment of the most common toxicities are included in section **4.2** and section **13.2.2**.

7.1.2 Risk of Apheresis:

Apheresis is a safe procedure that is routinely performed in healthy adults. Participants will be closely monitored and procedures to minimize risks and prevent side effects are incorporated into all aspects of the protocol. The institutions have broad expertise to adequately manage side effects. The potential risks of apheresis in this trial are as follows:

a. The most common side effects of apheresis are pain and bruising at IV sites. A central venous catheter may be required. Possible side effects include pain, bleeding, bruising, infection, thrombosis, vascular perforation.

b. During apheresis, mild side effects from citrate anticoagulant are common and include chills, numbness and tingling ("pins and needles"), anxiety, muscle cramps, and nausea. More serious side effects due to citrate-induced hypocalcemia are uncommon and include low blood pressure, seizures, weakness, and tetany. Citrate reactions rapidly resolve when the collection is slowed down or stopped. Prophylactic IV $CaCl_2$ and $MgSO_4$ infusions may be administered to subjects deemed to be at high risk of citrate toxicity. Risks of parenteral calcium and magnesium include extravasation necrosis and cardiovascular effects including bradycardia and blood pressure changes. However, side effects are unlikely given the low rate of infusion and use of large bore catheters for apheresis.

c. Transient mild thrombocytopenia is common after apheresis, but bleeding is unlikely.

d. Dilutional anemia occurs during apheresis, but this is unlikely to be clinically significant.

e. Side effects of blood draws include pain and bruising, lightheadedness, and rarely, fainting.

7.2 ADVERSE EVENT DEFINITIONS

7.2.1 Adverse Event

An adverse event is defined as any reaction, side effect, or untoward event that occurs during the course of the clinical trial associated with the use of a drug in humans, whether or not the event is considered related to the treatment or clinically significant. For this study, AEs will include events reported by the subject, as well as clinically significant abnormal findings on physical examination or laboratory evaluation. A new illness, symptom, sign or clinically significant laboratory abnormality or worsening of a pre-existing condition or abnormality is considered an AE.

Adverse events will be collected and documented from the start of lymphodepletion chemotherapy and conclude 30 days after the last dose of study treatment. Prior to start of conditioning lymphodepletion chemotherapy, only unexpected serious adverse events considered related or possibly related to study procedures (i.e. leukapheresis) will be recorded and reported. Additional disease directed therapy after completing apheresis but before the start of lymphodepletion chemotherapy, will not be allowed. Toxicities present at the initiation of lymphodepletion chemotherapy will be considered baseline conditions.

Serious adverse events that occur more than 30 days after the last administration of investigational agent/intervention and have an attribution of at least possibly related to the agent/intervention should be recorded and reported as per this Section.

An abnormal laboratory value will be considered a reportable, recordable AE if the laboratory abnormality is characterized by any of the following:

- Results in discontinuation from the study therapy
- Is associated with clinical signs or symptoms
- Requires treatment or any other therapeutic intervention
- Is associated with death or another serious adverse event, including hospitalization.
- Is judged by the Investigator to be of significant clinical impact
- Is a hematologic abnormality, including WBCs, hemoglobin, neutrophils, lymphocytes, and platelets that constitutes a change in grade from baseline.

If any abnormal laboratory result is considered clinically significant, the investigator will provide details about the action taken with respect to the test drug and about the subject's outcome.

7.2.2 Suspected adverse reaction

Suspected adverse reaction means any adverse event for which there is a reasonable possibility that the investigational therapy caused the adverse event. For the purposes of IND safety reporting, 'reasonable possibility' means there is evidence to suggest a causal relationship between the investigational therapy and the adverse event. A suspected adverse reaction implies a lesser degree of certainty about causality than adverse reaction, which means any adverse event caused by a drug.

7.2.3 Unexpected adverse reaction

An adverse event or suspected adverse reaction is considered “unexpected” if it is not listed in the protocol or informed consent documents or is not listed at the specificity or severity that has been observed; or is not consistent with the risk information described in the general investigational plan or elsewhere in the current application. "Unexpected” also refers to adverse events or suspected adverse reactions that are mentioned in the protocol or informed consent documents as occurring with a class of drugs or as anticipated from the pharmacological properties of the drug, but are not specifically mentioned as occurring with the particular drug under investigation.

7.2.4 Serious

An Unanticipated Problem or Protocol Deviation is serious if it meets the definition of a Serious Adverse Event or if it compromises the safety, welfare or rights of subjects or others.

7.2.5 Serious Adverse Event

An adverse event or suspected adverse reaction is considered serious if in the view of the investigator or the sponsor, it results in any of the following:

- Death,
- A life-threatening adverse drug experience
- In-patient hospitalization or prolongation of existing hospitalization
- Persistent or significant incapacity or substantial disruption of the ability to conduct normal life functions
- A congenital anomaly/birth defect.
- Important medical events that may not result in death, be life-threatening, or require hospitalization may be considered a serious adverse drug experience when, based upon appropriate medical judgment, they may jeopardize the subject or subject and may require medical or surgical intervention to prevent one of the outcomes listed in this definition.

7.2.6 Disability

A substantial disruption of a person’s ability to conduct normal life functions.

7.2.7 Life-threatening adverse drug experience

Any adverse event or suspected adverse reaction that places the subject or subject, in the view of the investigator or sponsor, at immediate risk of death from the reaction as it occurred, i.e., it does not include a reaction that had it occurred in a more severe form, might have caused death.

7.2.8 Protocol Deviation

Any change, divergence, or departure from the IRB-approved research protocol.

7.2.9 Non-compliance

The failure to comply with applicable IRB requirements, FDA or other regulatory requirements for the protection of human research subjects.

7.2.10 Unanticipated Problem

Any incident, experience, or outcome that:

- Is unexpected in terms of nature, severity, or frequency in relation to

  (a) the research risks that are described in the IRB-approved research protocol and informed consent document, or other study documents, and

  (b) the characteristics of the subject population being studied; **AND**

- Is related or possibly related to participation in the research; **AND**
- Suggests that the research places subjects or others at a *greater risk of harm* (including physical, psychological, economic, or social harm) than was previously known or recognized.

7.3 Adverse Event Reporting

Both Serious and Non-Serious Adverse Events will be clearly noted in source documentation and listed on study specific Case Report Forms (CRFs). The Principal Investigator or designee will assess each Adverse Event (AE) to determine whether it is unexpected according to the Informed Consent, Protocol Document, or related to the investigation. All Serious Adverse Events (SAEs) will be tracked until resolution and until 30 days after the last dose of the study treatment, whichever is later.

Adverse events will be recorded and reported as per section **7.2.1**.

In addition, any suspected serious adverse events that occur after 30 days, during the initial 5 years of follow up, will also be recorded and reported (section **5.5.7**). Suspected serious adverse events occurring in subjects who received cell therapy that are potentially related to the gene therapy nature of this study will be reported at the time of their occurrence during 15 year follow up (section **5.5.7**).

7.3.1 Stanford Reporting

Serious Adverse Events (SAEs) (as defined in section **7.2.4**) graded according to CTCAE v5.0 and Appendix B, section **13.2,** Grade 3 and above, and all subsequent follow-up reports will be reported to the Stanford Cancer Institute Data and Safety Monitoring Committee (DSMC) regardless of the event's relatedness to the investigation for institutional reporting purposes.

Events meeting the IRB definition of 'Unanticipated Problem' will be reported to the IRB using eProtocol within 10 working days of DSMC review, or within 5 working days for deaths or life-threatening experiences.

All SAEs attributed possibly, probably or definitely related to GD2CART, regardless of expectedness, will be reported to the APB using eProtocol within 10 working days of determination, or within 5 working days for deaths or life-threatening experiences.

APB and IRB will be notified via an expedited safety report if any stopping rules are triggered, as described in section **12.6**.

Non-serious AEs will be reported to IRB and APB at annual continuing review.

7.4 IND SPONSOR REPORTING CRITERIA

The Principal Investigator must **immediately** (within 24 hours of knowledge of event) report to the sponsor, using the Form FDA MedWatch 3500a or institutional SAE report form, any serious adverse event, whether or not considered drug related or expected, including those listed in the protocol or informed consent documents and must include an assessment of whether there is a reasonable possibility that the CAR T cell therapy caused the event. The Principal Investigator is responsible for promptly providing the Sponsor with any information needed to determine whether the SAE requires submission of an IND Safety Report.

Study endpoints that are serious adverse events (e.g. all-cause mortality) must be reported in accordance with the protocol unless there is evidence suggesting a causal relationship between the study therapy and the event (e.g. death from anaphylaxis). In that case, the investigator must immediately report the death to the sponsor.

Non-serious adverse events must be recorded in a timely manner and reported to Sponsor at IRB continuing review and in IND Annual Reports.

Events will be submitted to Sponsor Dr. Crystal Mackall, at:

Crystal Mackall, M.D.

Professor Pediatrics & Medicine

Associate Director, Stanford Cancer Institute

265 Campus Dr G3141A, MC5456

Stanford, CA 94305

650-725-9670

7.4.1 Reporting Pregnancy

- ✓ Maternal exposure

If a subject becomes pregnant during the course of the study (for 4 months after the last cell infusion and for as long as CAR cells are detectable in the blood), the study treatment should be discontinued immediately and the pregnancy reported to the Sponsor and the Stanford regulatory authorities. The potential risk of exposure of the fetus to the investigational agent(s) or chemotherapy agents (s) should be documented in box B5 of the MedWatch form "Describe Event or Problem".

Pregnancy itself is not regarded as an AE unless there is a suspicion that the study treatment under study may have interfered with the effectiveness of a contraceptive medication. However, as subjects who become pregnant on study risk intrauterine exposure of the fetus to agents which may be teratogenic, the Sponsor is requesting that pregnancy should be reported in an expedited manner as **Grade 3** "***Pregnancy, puerperium and perinatal conditions - Other (pregnancy)***" under the ***Pregnancy, puerperium and perinatal conditions*** SOC.

Congenital abnormalities or birth defects and spontaneous miscarriages should be reported and handled as SAEs. Elective abortions without complications should not be handled as AEs. The

outcome of all pregnancies (spontaneous miscarriage, elective termination, ectopic pregnancy, normal birth, or congenital abnormality) should be followed up and documented.

If any pregnancy occurs in the course of the study, then the investigator should inform the Sponsor within 1 day, i.e., immediately, but **no later than 24 hours** of when he or she becomes aware of it.

The designated Sponsor representative will work with the investigator to ensure that all relevant information is provided to the Sponsor within 1 to 5 calendar days for SAEs and within 30 days for all other pregnancies.

The same timelines apply when outcome information is available.

- ✓ Paternal exposure

Male subjects should refrain from fathering a child or donating sperm during the study and for 4 months after the last dose of study treatment and for as long as CAR cells are detectable in the blood.

Pregnancy of the subject's partner is not considered to be an AE. However, the outcome of all pregnancies (spontaneous miscarriage, elective termination, ectopic pregnancy, normal birth, or congenital abnormality) occurring from the date of the first dose until 4 months after the last dose should, if possible, be followed up and documented.

7.5 FDA Reporting Criteria

7.5.1 IND Safety Reports to the FDA (Refer to 21 CFR 312.32)

The Sponsor will be responsible for reporting to the FDA any unexpected fatal or life-threatening suspected adverse reactions as soon as possible but no later than 7 calendar days of initial receipt of the information using appropriate reporting format.

Additionally, any new Grade 3 neurotoxicity (not present at baseline and excluding cranial neuropathies and ataxia which are common in this disease) lasting longer than 96 hours will be submitted to FDA in an expedited fashion.

7.5.2 FDA Annual Reports (Refer to 21 CFR 312.33)

The Sponsor will submit the Annual Report to the FDA according to 21CFR§312.33, and any associated FDA correspondences regarding the IND annual report.

7.5.3 Serious Adverse Event Reporting on Cell Therapy Products to the FDA

A sample from all products that are non-conforming or do not meet release specifications will be used to conduct an out of specification investigation and the remainder either disposed of according to our facility biohazardous material disposal SOP or the FDA will be contacted by the manufacturing team to determine whether the product is suitable for infusion. The manufacturing facility will report all products manufactured including those that did not meet release criteria or were otherwise not infused in the annual IND report to the FDA.

All HCT/P deviations involving 351 cell products will be reported using MedWatch From FDA3500A according to FDA publication "Guidance for Industry: MedWatch Form FDA 3500A: Mandatory Reporting of Adverse Reactions Related to Human Cells, Tissues, and Cellular and Tissue-Based Products (HCT/Ps) available at: http://www.fda.gov/BiologicsBloodVaccines/GuidanceComplianceRegulatoryInformation/Guidances/Tissue/ucm074000.htm.

7.5.4 Action Plan for Positive Results on Cell Product Safety Testing:

In the unlikely event that a positive sterility test or mycoplasma test result is obtained after distribution of a cell product or after administration of the product to the subject, the following steps will be initiated IMMEDIATELY:

a. Stanford Director of Manufacturing and Process Development (or designee) will notify the IND Sponsor (Sponsor's Medical Representative) and principal investigator at 650-721-5750. Both will be updated with any substantive changes, including the final report on the identification and sensitivity from the positive sterility test. The Miltenyi QS personnel will determine the need for quality improvement based on the nature and extent of the incident.
b. If Stanford Center for Cancer Cell Therapy Director of Manufacturing and Process Development (or designee) is unable to reach Principal Investigator within 15 minutes, contact inpatient attending physician caring for the subject on the hospital service via hospital page with direct communication.. NOTE: The Sponsor (Sponsor's Medical Representative), Principal Investigator, and/or designee will contact the attending physician, who will determine the extent of the work-up of a positive culture in consultation with appropriate infectious disease consultants, as well as determine an appropriate action treatment plan.
c. The Principal Investigator/attending physician will discuss the positive results with the subject, and specify the clinical therapy, antibiotic regimen and/or monitoring plan.
d. A contaminated sample of a product that has been administered to a subject will be handled in the same fashion as a Grade 4/5 toxicity. The Principal Investigator will be responsible for notifying the IRB and APB via an Unanticipated Problem (UP) report within 5 working days, and the Sponsor will notify the FDA via an expedited 7-day IND Safety Report.

In addition to the above, appropriate Safety reporting will be done as per SOP. A sample of each product is retained by Quality Systems and will be sent to the Microbiology Laboratory for repeat testing and speciation. An Out-of-Specification (OOS) Investigation will be conducted by the Quality Systems staff of the manufacturing laboratory including root cause analysis, review of viable environmental monitoring results collected at the time of manufacturing on personnel, equipment and reagents. Whether or not attribution is established, a formal Corrective and Preventive Action plan will be issued by the manufacturing staff and appropriate remediation will be performed including retraining of manufacturing personnel, elimination of any contaminated reagents and re-cleaning of the production facility followed by viable microbiological monitoring to establish effectiveness of cleaning.

8 CORRELATIVE/SPECIAL STUDIES

CAR T cell therapy targeting a single tumor associated antigen has mediated striking remissions in B cell leukemia and lymphoma/DLBCL. The clinical experience to date has demonstrated variable patterns of tumor remission and CAR T cell efficacy and persistence. In subjects treated with singular CD19 specific or CD22 specific CAR T cells, relapse patterns have included both respective CD19 or CD22 negative disease or relapsed disease with persistent, variable surface expression of the targeted antigen. The complex interplay of tumor, T cell and intrinsic CAR properties that influence these outcomes are not well understood. We aim to utilize this study as an opportunity to collect correlative data that will permit extensive study of cell compartments prior to and following CAR T cell therapy. We aim to integrate multi-dimensional technologies to permit complex analyses of the apheresis product, the CAR T cell product pre-infusion and *in vivo* expanded CAR T cells following antigen encounter. We additionally aim to investigate properties of DIPG tumors that render them resistant or susceptible to CAR T cell cytotoxicity.

Overall goal of study correlatives:

- Conduct analyses of the manufactured T cell product and blood and CSF post-infusion to identify biomarkers associated with enhanced CAR T cell expansion, persistence, and/or phenotype.
- Assess whether changes in the level of ctDNA in the cerebrospinal fluid can provide prognostic information and/or information regarding clonal evolution of DIPG over time.
- Evaluate whether antigen expression or tumor microenvironment are correlated with response to CAR T cell

Specific Aims:

1) CAR T cell correlatives:

- Measure expansion, persistence, and phenotype of adoptively transferred GD2CART in the CSF and blood and correlate with antitumor effects.
- Characterize CAR T cell functionality using cytokine analysis of patient samples during the period of highest likelihood of CAR expansion. Compare CSF to peripheral blood cytokine production for evaluation of functional effects of CAR in the CNS space.
- Where possible, use TCR sequencing to fate map cells contained in the manufactured CAR product to evaluate persistent GD2CART and identify subsets with a greater likelihood of T cell persistence in the setting of adoptive cell therapy.
- Evaluate chromatin structure and epigenomic modifications of CAR T cells using ATAC-seq and couple with RNA-seq for transcriptomic evaluation of CAR T cells.
- Assess the impact of T cell subset composition as delineated using flow cytometry, mass cytometry and ATAC-Seq on CAR T cell expansion, persistence, and phenotype.
- Compare persistence of GD2CART administered in this trial to that observed in a simultaneous trial that will administer GD2CART for pediatric solid tumors at similar doses and with the same lymphodepleting regimen.

2) Tumor correlatives:

- Assess whether changes in the level of ctDNA in the cerebrospinal fluid or peripheral blood can provide prognostic information and/or information regarding clonal evolution of DIPG before, during, and after CAR T cell therapy.
- Characterize antigen expression and evaluate tumor microenvironment on relapsed tumor tissue samples prior to and/or following CAR-mediated targeting

8.1 Sample Collection Schedule

8.1.1 Study Correlatives

The samples to be collected and schedule for sample collection is detailed in section **13.7**, Appendix G.

8.1.2 Persistence of genetically engineered transduced cells

The procedures and methodologies for testing persistence of genetically engineered transduced cells are specified in section **13.7**.

If any subject has more than 5% persistence of gene transduced cells at Month 6 using 1A7, an anti-idiotype antibody that recognized the anti-GD2 CAR T cells, samples will be studied for clonality of persisting gene transduced cells. Such techniques may include analysis of BV chain expression, T cell cloning or LAM-PCR. If a predominant or monoclonal T cell clone derived from gene transduced cells is identified during the follow-up, the integration site and sequence will be identified and subsequently analyzed against human genome database to determine whether the sequences are associated with any known human cancers. If a predominant integration site is observed, the T cell cloning or LAM-PCR test will be used at an interval of no more than 3 months after the first observation to see if the clone persists or is transient. In all instances where monoclonality is persistent and particularly in instances where there is expansion of the clone, regardless of whether or not the sequence is known to be associated with a known human cancer, the subject should be monitored closely for signs of malignancy, so that treatment, if available, may be initiated early.

8.2 Blood Drawing Limits for Research Purposes

8.2.1 Adults

The volume to be drawn per day for research purposes will be limited based on institutional policy. Subjects may undergo a small volume apheresis (approx. one to two blood volumes) in lieu of standard blood draw to obtain peripheral blood lymphocytes for correlative studies including Day 28 (± 1 week), and 3 (± 1 month), 6 (± 1 month), 9 (± 1 month), and 12 (± 2 months) months.

8.2.2 Pediatric Subjects:

The volume to be drawn per day for research purposes will be limited based on institutional policy. Subjects may undergo a small volume apheresis (approx. one to two blood volumes) in lieu of standard blood draw to obtain peripheral blood lymphocytes for correlative studies including Day 28, and 3 (± 14 days), 6 (± 14 days), 9 (± 14 days), and 12 (± 30 days) months. If the volume of blood exceeds that limit, the correlative studies will be prioritized as follows:

- CAR T correlatives/monitoring:
- PCR for RCR
- PCR for CAR
- Flow for CAR
- Cytof (T and B cell panels)
- TCR sequencing/single cell analysis
- Epigenomics/ATAC-Seq
- Circulating tumor DNA

9 STUDY CALENDAR

			Preparative Regimen and Cell Infusion			Post Treatment Assessment				Long Term Follow-Up			
**Procedure**	**Screening[a]**	**Apheresis** (up to 48 hours prior, unless noted)	**Day -4, Day -3, Day -2**	**Prior to cell infusion** (< 24 hours, unless noted)	**Day 0**	**Daily, Day 1-Day 14 (± 2 d)**	**Twice weekly, Day 15-Day 27 (± 4 d)**	**Day 28 (± 4 d)**	**Monthly, Month 2-3 (±2 weeks)**	**Month 6 (±1 mo.) 9, (±1 mo) 12 (±2 mo) q6-12 months to 5 years**	**Annually, Year 6 to 15 (± 3 mos)**	**At Disease Progression**	**Long Term Gene Therapy Follow Up (until Yr 15)**
History (at screening) and Physical Exam	X			X		X	X	X	X	X			
Vital signs	X	X	X	X	X[k]	X	X	X	X	X			
Neurologic exam	X			X		X[n]	X[n]	X	X	X			
Performance Status	X							X	X	X			
Height	X		X[c]										
Weight	X	X	X[c]	X				X					
ICP measure (Ommaya)				X[i]	X[i]	X[i]	X[i]	X[i]					
Labs													
• CBC with diff	X	X[b]	X	X		X[m]	X[m]	X	X	X			
• PT/PTT	X												
• Chemistries[d]	X	X[b]	X	X		X[m]	X[m]	X	X				
• Phosphorus	X	X[b]	X[c]			X[m]	X[m]						
• Magnesium	X	X[b]	X[c]	X		X[m]	X[m]						
• CRP	X	X[b]	X[c]	X		X[m]	X[m]	X					
• Ferritin	X		X[c]					X					
• Uric acid and LDH			X[c]			X[m]	X[m]						
• HIV, HBsAg, HBsAb HBcAb, anti-HCV Ab		X[e]											
• Urinalysis	X			X									
• β-HCG pregnancy test on females of child-bearing potential	X	X[b]	X[b]										
• RCR				X[r]					X[r]	X[r]			
ECG	X												
ECHO, MUGA or cardiac MRI	X[f]												
Correlative Research Studies	Correlative samples outlined in Section 13.7, Appendix G												

Agent: GD2.BB.z.iCasp9-chimeric antigen receptor (GD2 CAR) retroviral transduced autologous peripheral blood lymphocytes; following fludarabine and cyclophosphamide

			Preparative Regimen and Cell Infusion			Post Treatment Assessment				Long Term Follow-Up			
**Procedure**	**Screening[a]**	**Apheresis (up to 48 hours prior, unless noted)**	**Day -4, Day -3, Day -2**	**Prior to cell infusion (< 24 hours, unless noted)**	**Day 0**	**Daily, Day 1-Day 14 (± 2 d)**	**Twice weekly, Day 15-Day 27 (± 4 d)**	**Day 28 (± 4 d)**	**Monthly, Month 2-3 (±2 weeks)**	**Month 6 (±1 mo.) 9, (±1 mo) 12 (±2 mo) q6-12 months to 5 years**	**Annually, Year 6 to 15 (± 3 mos)**	**At Disease Progression**	**Long Term Gene Therapy Follow Up (until Yr 15)**
• Leukapheresis		X[g]						X[o]	X[o]	X[l]			
Disease Evaluation													
• H3K27M mutation documentation	X[q]												
• Imaging: Brain MRI or CT, MRI with and w/o gadolinium,	X[h]			X[h]		X[j]	X[j]	X[h,s]	X[h,s]	X[h,s]		X[h,]	
Treatment Regimen													
• Fludarabine			X[l]										
• Cyclophosphamide			X[l]										
• GD2CART infusion					X[p]								
Response Evaluation								X[h,s]	X[h,s]	X[h,s]		X[h,s]	
Adverse Events		X	X[t]	X[t]	X[t]	X[t]	X[t]	X[t]	X[t]	X[t]	X[t]	X[t]	X[t]
Concomitant Medications	X	X	X	X	X	X	X	X	X	X[u]	X[u]		X[u]
Long term follow up questionnaires										X[u]	X[u]		X[u]

a: Within 28 days, unless otherwise specified

b: Once within 7 days of leukapheresis or start of lymphodepleting chemotherapy

c: Day -5 or -4 only

d: Laboratory evaluation to include; Chemistries: Sodium (Na), Potassium (K), Chloride (Cl), Total CO2 (bicarbonate), Creatinine, Glucose, Urea nitrogen (BUN), Albumin, Calcium total, Alkaline Phosphatase, ALT/GPT and/or AST/GOT, Bilirubin, Total Protein); creatinine clearance may be performed if the serum creatinine is elevated.

e: If collected on this study, within 28 days prior to leukapheresis. If cryopreserved from previous collection, within institutional standard time frame for autologous collection.

f: Testing performed within 180 days may be used for confirmation of eligibility

g: For cell acquisition for product development. This may be skipped if subject has cryopreserved cells that meet IND criteria.

h: Disease evaluations will be specific to the subject's location of disease: (brain MRI or MRI with and w/o gadolinium): Screening, once within 7 days prior to cell infusion (at investigator's discretion), Day 28 (±7 days) and Month 3 (± 1 month), 6 (± 1 month), 9 (± 1 month), 12 (± 2 months), then q6-12 (± 2 months) months. If subject's disease has not progressed by Month 24, disease assessments will continue at standard of care frequency

i: In subjects with DIPG, measurement of ICP via Ommaya Catheter will be performed at baseline (Day 0 prior to infusion), Day 4, Day 7, Day 11, Day 14, Day 21 and Day 28, and with evidence of increased ICP or clinical deterioration.

j: Subjects with DIPG will undergo brain MRI on D7, D14, and D21. If clinical condition post-infusion prevents MRI, a bedside CT will be obtained on those days.

k: Vital signs (blood pressure, heart rate, respiratory rate, pulse oximetry, temperature) prior to infusion, within 15 minutes after start of infusion (±10 min), and then 30 (±10 min), 60 (±10 min) and 120 (±10 min) minutes after infusion.

l: Fluid support and supportive medications as per section **5.3** and institutional procedures.

m: Daily until Day 7 (± 2 days) and then Day 14 (± 2 days) and then Day 21 (± 4 days).

n: Neurologic exam daily with documentation of ICANS Consensus Grading; ICE score / CAPD evaluated every shift (± 2 hours) and with any change in neurologic functioning until day 7, then daily until day 27 (± 4 days); neurologic exam with each visit after discharge.

o: Subjects may undergo a small volume apheresis (approx. one to two blood volumes) in lieu of standard blood draw to obtain peripheral blood lymphocytes for correlative studies including Day 28 (±7 days), and 3 (± 1 month) , 6 (± 1 month) , 9 (± 1 month) , and 12 (± 2 months) months.

p: Premedications will be provided as described in protocol. Subject may be offered additional CAR T cell treatments if enough cells were manufactured from the initial preparation, the subject experienced at least partial response to the first infusion, and the subject meets all eligibility criteria. Subsequent infusions will follow the same procedures as the first.

q: H3K27M mutation documentation requirement can be met anytime since diagnosis.

r: RCR obtained prior to cell infusion (may be obtained at any time prior to infusion), and at 3 (±1 month), 6 (± 1 month), and 12 months (± 2 months) post cell administration. Subsequent RCR blood samples will be stored annually x 5 years if RCR in first year negative.

s: If no response (i.e., progressive disease) by Day 28, then no further close clinical follow-up required and subject will be followed as per section **5.5.4.1**. If subject has SD, PR or CR monitor by physical exams, disease evaluations, vitals, CBC with diff, Chemistries until PD or subject proceeds to other therapies at investigator's discretion; at which time long-term follow-up will proceed as per section **5.5.4.1**

t: Adverse events will be collected and documented from the start of lymphodepletion chemotherapy and conclude 30 days after the last dose of study treatment. Prior to start of conditioning lymphodepletion chemotherapy, only unexpected serious adverse events considered related or possibly related to study procedures (i.e. leukapheresis) will be recorded and reported. Serious adverse events that occur more than 30 days after the last administration of investigational agent/intervention and have an attribution of at least possibly related to the agent/intervention should be recorded and reported

u: Gene Therapy Follow Up: Annual Physical exam, vital signs and performance status (may be performed by local physician) X 5 years (specifically document any new malignancies, new incidence or exacerbation of a pre-existing neurologic disorder, new incidence or exacerbation of a prior rheumatologic or other autoimmune disorder, new incidence of a hematologic disorder, targeted AEs and targeted con meds). After 5 years, health status data will be obtained from surviving subjects via telephone contact or mailed/emailed questionnaires. The long term follow up period for retroviral vectors is 15 years.

.

10 MEASUREMENTS

10.1 OUTCOME MEASURES

10.1.1 Primary Objectives:

10.1.1.1 Determine the feasibility of manufacturing GD2CART for administration in children and young adults with H3K27M DIPG or spinal H3K27M mutated DMG using retroviral vector and dasatinib in the Miltenyi CliniMACS Prodigy® system.

Feasibility is defined by the rate of successful manufacture of the GD2CART produced with the Miltenyi CliniMACS Prodigy® system to satisfy the targeted dose level and meet the required release specifications.

10.1.1.2 Assess the safety and identify the maximum tolerated dose (MTD)/recommended phase 2 dose (RP2D) of GD2CART in subjects with H3K27M DIPG administered after cyclophosphamide/fludarabine based lymphodepletion regimen using the following dose escalation schedule: DL1: 1e6 transduced T cells/kg; DL2: 3e6 transduced T cells/kg; DL3: 10e6 transduced T cells/kg. The MTD is defined as the dose level below that in which 2/6 subjects with experience DLTs during dose escalation.

10.1.1.3 Safety of GD2CART as evidenced by the incidence and severity of dose limiting toxicities (DLT), adverse events, serious adverse events, laboratory abnormalities, changes in vital signs, and changes in physical examination following infusion of GD2CART graded according to the Common Terminology Criteria for Adverse Events (CTCAE) Version 5.0 and Appendix B, section 13.2 in subjects with H3K27M DIPG and subjects with spinal H3K27M mutated DMG[7].

10.1.2 Secondary Objectives:

10.1.2.1 In a preliminary manner, assess clinical benefit of GD2CART at the RP2D in children and young adults with H3K27M DIPG and spinal H3K27M mutated DMG.

Clinical benefit will be measured by overall survival (OS) in subjects with DIPG and in subjects with DMG. In addition, post progression survival (PPS), progression free survival (PFS), time to progression (TTP), and radiographic and clinical response will be evaluated.

10.1.2.1.1 Clinical Benefit Definitions

- Overall Survival (OS)

OS is defined as the time from the start of the lymphodepleting chemotherapy preparative regimen to the date of death from any cause.

- Post Progression Survival (PPS)

PPS is measured for each subject with DIPG as OS minus PFS, and for each patient with recorded progression as OS minus TTP[7].

- Progression Free Survival (PFS)

PFS is defined as the time from the start of the lymphodepleting chemotherapy preparative regimen to the date of radiographic progression or death from any cause.

- Time to Progression (TTP)

TTP is the time from the start of the lymphodepleting chemotherapy preparative regimen to the date of radiographic progression (death is censored).

10.1.2.1.2 Tumor Response Criteria

Complete Response (CR)

Complete disappearance on MR of all evaluable tumor and mass effect, on a stable or decreasing dose of corticosteroids (or receiving only adrenal replacement doses), accompanied by a stable or improving neurologic examination. If CSF was positive, it must be negative.

Partial Response (PR)

Greater than or equal to 50% reduction in tumor size by bi-dimensional measurement, as compared with the baseline measurements, on a stable or decreasing dose of corticosteroids, accompanied by a stable or improving neurologic examination. *Axial FLAIR images will be used for tumor measurements.*

Stable Disease (SD)

Neurologic exam is at least stable and maintenance corticosteroid dose not increased, and MR/CT imaging meets neither the criteria for PR nor the criteria for Progressive Disease

Progressive Disease (PD)

Progressive neurologic abnormalities or worsening neurologic status not explained by causes unrelated to tumor progression (e.g., anticonvulsant or corticosteroid toxicity wean, electrolyte disturbances, sepsis, hyperglycemia, etc.), OR a greater than 25% increase in the bi-dimensional measurement, taking as a reference the smallest disease measurement recorded since the start of protocol therapy, OR the appearance of a new tumor lesion.

Increasing doses of corticosteroids required to maintain stable neurological status should be strongly considered as a sign of clinical progression unless in the context of recent wean or transient neurologic change due e.g. to radiation effects.

10.1.2.2 If unacceptable toxicity occurs that is possibly, probably or likely related to GD2CART, assess the capacity for AP1903, a dimerizing agent, to mediate clearance of the genetically engineered cells and resolve toxicity.

In the event of unacceptable toxicity (defined as grade 4 life threatening toxicity believed by the investigators to cause substantial risk to the subject) possibly, probably or definitely related to GD2CART, administration of AP1903 will eliminate the persistence of genetically engineered cells, and allow resolution of toxicity.

10.2 Exploratory Objectives

1. Measure expansion/persistence/phenotype of adoptively transferred GD2CART in the CSF and blood and correlate this with antitumor effects.

2. Conduct analyses of the manufactured T cell product and blood and CSF post-infusion to identify biomarkers associated with enhanced CAR T cell expansion, persistence and/or phenotype.
3. Assess whether changes in the level of ctDNA in the cerebrospinal fluid can provide prognostic information and/or information regarding clonal evolution of DIPG over time.
4. Evaluate whether antigen expression or tumor microenvironment are correlated with response to CAR T cell.

10.3 Institutional Review of Protocol

The protocol, the proposed informed consent and all forms of participant information related to the study (e.g. advertisements used to recruit participants) will be reviewed and approved by the Stanford IRB and Stanford Cancer Institute Scientific Review Committee (SRC). Any changes made to the protocol will be submitted as a modification and will be approved by the IRB prior to implementation. The Protocol Director will disseminate the protocol amendment information to all participating investigators.

10.4 Data and Safety Monitoring Plan

10.4.1 Clinical Team

The clinical research team will meet on a regular basis during dose escalation and when subjects are being actively treated on the trial to discuss cell manufacturing, toxicities, eligibility questions, trial accrual, and treatment needs. Decisions about dose level enrollment and dose de-escalation if applicable will be made based on the toxicity data from prior subjects in each disease group. Members from cell processing facilities will join as needed.

All data will be collected in a timely manner and reviewed by the principal investigator or a lead associate investigator. Adverse events will be reported as required above. Any safety concerns, new information that might affect either the ethical and or scientific conduct of the trial, or protocol deviations will be immediately reported to the IRB, DSMC, APB and to the Sponsor.

The principal investigator will review adverse event and response data on each subject to ensure safety and data accuracy. The principal investigator will personally conduct or supervise the investigation and provide appropriate delegation of responsibilities to other members of the research staff.

10.4.2 Data Safety Monitoring Committee (DSMC)

The Stanford Cancer Center Data and Safety Monitoring Committee (DSMC) will be the monitoring entity for this study. The DSMC will audit study-related activities to determine whether the study has been conducted in accordance with the protocol, local standard operating procedures, FDA regulations, and Good Clinical Practice (GCP). In addition, the DSMC will regularly review serious adverse events, adverse events, and protocol deviations associated with the research to ensure the protection of human subjects. Results of the DSMC audit will be communicated to the IRB and APB and the appropriate regulatory authorities at the time of continuing review, or in an expedited fashion, as needed.

10.4.3 Safety Monitoring Committee (SMC)

The Center for Cancer Cell Therapy (CCT) has assembled an independent SMC consisting of Stanford and external academic investigators who are independent of the clinical trial under review and who are knowledgeable in cellular therapies to review all serious adverse events (SAEs), subject deviations, and internal and external audit/monitoring reports. The SMC will confirm dose escalation decisions based on available AEs and dose limiting toxicity (DLT) determinations for each dose cohort, and determine when the study needs to be halted based on protocol specified safety rules.

10.5 DATA MANAGEMENT PLAN

Case Report Forms (CRFs) are printed or electronic documents designed to record all protocol-related information on each trial participant. CRFs should summarize the clinical findings and observations necessary to ensure safety of participants on the study, and to document the study outcomes. Data will be entered into the Stanford database.

All data will be kept secure. Personal identifiers will not be used when collecting and storing data. Paper study files will be kept in a locked, secure location. Electronic study data will be stored in encrypted, backed-up, password protected computers. An enrollment log will be maintained in the regulatory binder/file which is the only location of personal identifiers with unique subject identification number.

11 COLLABORATIVE AGREEMENTS

Collaborative research agreement exists for conduct of correlative studies with Adaptive Biosciences, Inc. This collaborative agreement is disclosed in the informed consent document. A contract for manufacturing the cell product exists with Miltenyi Biotec Inc., which is disclosed in the informed consent document.

12 STATISTICAL CONSIDERATIONS

12.1 STATISTICAL DESIGN

A formal statistical analysis plan (SAP) will be prepared and finalized before database lock for the final analysis for the study report. The SAP will provide details regarding the definition of analysis subjects (populations), analysis variables, and analysis methodology to meet all study objectives.

The principle and key elements of the SAP are provided as follows:

- In general, safety and efficacy data will be summarized with descriptive statistics, including means, standard deviations, medians, minimums and maximums for continuous variables, the number of subjects and percent in each category for categorical variables.
- Data from each individual will be tabulated as appropriate. Efficacy and safety endpoints will be tabulated by dose cohort and time point.

The aims of this clinical trial are three-fold:

1. Determine the feasibility of manufacturing GD2CART for administration in subjects with H3K27M DIPG and subjects with spinal H3 K27M-mutant DMG using a retroviral vector

and dasatinib in the the Miltenyi CliniMACS Prodigy® system. The CliniMACS Prodigy® represents the next generation in automated cell processing, combining and streamlining cell processing workflows into one closed system.

2. Assess the safety and identify the maximum tolerated dose (MTD)/recommended phase 2 dose (RP2D) of GD2CART in subjects with H3K27M DIPG administered after cyclophosphamide/fludarabine based lymphodepletion regimen using the following dose escalation schedule: DL1: 1 X 10^6 transduced T cells/kg; DL2: 3 X 10^6 transduced T cells/kg; DL3: 10 X 10^6 transduced T cells/kg. In addition, the safety of the MTD/RP2D will be assessed in children and adults with spinal H3 K27M-mutant DMG.
3. In a preliminary manner, assess clinical activity of GD2CART at the RP2D in children and young adults with H3K27M DIPG or spinal H3 K27M-mutant DMG.

The precedent for conducting clinical trials of this scope was established by the ongoing clinical trials CCT5001/IRB-41382 and CCT5007/IRB-41383 conducted by Stanford Center for Cancer Cell Therapy.

12.2 PRIMARY OBJECTIVES:

✓ Determine the feasibility of manufacturing GD2CART for administration in subjects with H3K27M DIPG and subjects with spinal H3 K27M-mutant DMG using a retroviral vector in the Miltenyi CliniMACS Prodigy® system.

Feasibility is defined by the rate of successful manufacture of the GD2CART produced with the Miltenyi Prodigy to satisfy the targeted dose level and meet the required release specifications.

✓ Assess the safety and identify the maximum tolerated dose (MTD)/recommended phase 2 dose (RP2D) of GD2CART in subjects with H3K27M DIPG administered after cyclophosphamide/fludarabine based lymphodepletion regimen using the following dose escalation schedule: DL1: 1 X 10^6 transduced T cells/kg; DL2: 3 X 10^6 transduced T cells/kg; DL3: 10 X 10^6 transduced T cells/kg.

✓ Assess safety in children and young adults with spinal H3 K27M-mutant DMG administered GD2CART at MTD/RP2D after cyclophosphamide/fludarabine based lymphodepletion regimen.

The MTD is a dose level immediately below the level at which the enrollment is stopped due to DLT(s), as explained specifically below:

- If **more than one subject** in the first three subjects included in a dose level experience DLT as defined above, MTD will have been exceeded.
- If DLT develops in **one** of the 3 subjects included in a cohort, the cohort will be then expanded up to six:
- If 2 or more of these 6 included subjects develop DLT, the MTD will have been exceeded.

Safety of GD2CART as evidenced by the incidence and severity of dose limiting toxicities (DLT), adverse events, serious adverse events, laboratory abnormalities, changes in vital signs, and changes in physical examination following infusion of GD2CART at the recommended dose, recorded and graded according to the Common Terminology Criteria for Adverse Events

(CTCAE) Version 5.0 and Appendix B: Guidelines Toxicity Assessment and Management 13.2.

12.2.1 Feasibility

Feasibility will be defined as the successful manufacturing of GD2CART that meet established release criteria to satisfy the targeted dose level. Although we anticipate reaching the targeted cell dose during manufacture, feasibility of manufacturing cells remains a primary objective in this subject population. Subjects will be enrolled at the targeted dose level (until adequate subjects are enrolled to produce the correct number of cells for safety evaluation at that dose level) as long as 3 or more of the first 3 to 6 subjects in the targeted dose level are able to produce adequate cells for evaluation.

For example, this might mean that 6 to 9 subjects will need to be enrolled at a dose level to result in 6 for the safety evaluation. However, if less than 3 of 6 subjects at a given dose level are able to have adequate cells produced, evaluation of that level and beyond for safety and feasibility will not take place. If less than 3 of 6 subjects at dose level 1 are able to have adequate cells produced, enrollment will proceed to dose level -1. If less than 3 of 6 subjects are able to have adequate cells produced at dose level -1, enrollment to this study will stop. If cell growth limitations preclude administration of the targeted cohort cell dose, the subject will receive as many cells as possible, and be considered part of the lower dose cohort. If a minimum of 1.0 x 10^6 GD2CART per kg cannot be obtained for infusion, the subject may be treated but will not be evaluable for toxicity or response, but will be considered a feasibility failure.

If after the first 6 subjects have been enrolled at a given dose level, more than 3 are unable to have adequate GD2CART (that meet COA for infusion), accrual to that dose level will stop and the dose escalation phase of the study will also end, since the upper 90% one-sided confidence interval about 3/6 is 79.9%; thus, it would be unlikely that the true feasibility rate is 80% or greater for a given, which would be desirable. The evaluation of subjects in the expansion cohort will take place using the highest dose level at which feasibility, as well as safety, was identified. In the expansion cohorts, the fraction which are able to manufacture the targeted dose level will also be monitored, and beginning with the 6th subject in an expansion cohort, if at any point fewer than half of the enrolled subjects are able to manufacture an acceptable level of cells, the accrual to the expansion cohort will end.

12.2.2 Determination of MTD/RP2D

Assess the safety of administering escalating doses of autologous GD2CART that can be feasibly produced to meet established release specifications in subjects with H3K27M DIPG following a cyclophosphamide/fludarabine conditioning regimen. Dose escalation will proceed as outlined in **Table 1**.

The endpoint for determination of MTD/RP2D in subjects is evidenced by the incidence and severity of dose limiting toxicities (DLTs) (i.e. laboratory abnormalities, changes in vital signs, and changes in physical examination) following chemotherapy preparative regimen and infusion of GD2CART, recorded and graded according to the Common Terminology Criteria for Adverse Events (CTCAE) Version 5 (and Appendix B: Guidelines Toxicity Assessment and Management, section **13.2**) at three dose levels until the maximum tolerated dose (MTD) is determined. If Dose level 3 can be feasibly manufactured and is administered in up to 6 subjects without evidence of

DLT, this dose will be considered RP2D, or if no efficacy is observed, consideration will be given to amending the clinical trial to include additional dose levels.

The dose escalation procedure follows a 3+3 design beginning with dose level 1, based on the DLT count in each cohort, with escalation also constrained by the feasibility of producing the doses called for. The purpose of the design is not to invert the dose-toxicity curve at a target DLT rate, but rather to proceed with appropriate caution to dose level 3, which we expect will be reached without observing any DLT. Safety monitoring will continue throughout the study in the expanded cohorts of up to 20 subjects with pontine DMG and 10 subjects with spinal DMG at the final RP2D.

12.2.3 Safety assessment in Subjects with DIPG (expansion cohort) and Subjects with Spinal DMG treated at MTD/RP2D

As noted above, up to a total of 20 subjects with DIPG and 10 subjects with spinal DMG will receive the investigational regimen at MTD/RP2D.

In a safety ‘lead-in’ assessment, initially 3 subjects with spinal DMG will receive GD2CART at RP2D in which subjects will be staggered by 14 days between each subject infusion. Should 1/3 subjects experience a DLT, the safety ‘lead in’ will be expanded to 6. If an additional subject in the safety lead in experiences a DLT the dose will be de-escalated and a safety assessment will be conducted at the next lower dose of GD2CART.

The expansion cohort of subjects with DIPG treated at MTD/RP2D, including those evaluable during dose escalation, and the cohort of subjects with spinal DMG, the GD2CART dose will be decreased by one dose level if the tabulated threshold of adverse events that meet the criteria for Dose Limiting Toxicity (DLT) is met in either one of the expansion cohorts of subjects treated at RP2D.

- Specifications

  The maximum acceptable rate for DLT is 30%. We will perform safety monitoring to evaluate this DLT rate after 10 cell infusions in subjects with DIPG at the RP2D, and additionally after every 5 thereafter until we reach a total enrollment of 20 subjects with DIPG. The safety analyses are designed to reduce the dose if the observed rate at any evaluation is such that the lower one-sided 80% confidence limit (LCL80) exceeds the acceptable rate. We use the Wilson score method (Brown, Cai, and DasGupta 2001; Brown, Cai, and DasGupta 2002) to compute the LCL80.

  Based on these specifications, we will reduce the dose if the observed rate for DLT meets the criteria tabulated below for each level of enrollment. The actual LCL80 estimates show that the performance of this rule closely matches the maximum acceptable rate, and is not overly conservative.

  Dose De-escalation occurs if DLT meets these criteria

Number of DLTs	Current Enrollment	LCL 80
5	10	0.312
7	15	0.313
9	20	0.317

In the event that dose de-escalation occurs, the new cohort treated at the lower dose will be monitored under the same rule, but starting again at 10 subjects treated at the lower dose for the first look. If the rule is triggered in the de-escalated cohort, enrollment will stop. In operation, the rule is implemented with the convention that if the number of DLTs reaches the critical level before the monitoring cohort is completely ascertained (so that the result is completely foreseeable), the de-escalation will be triggered in subjects with spinal DMG. So, for example, if there are 5 DLTs out of the first 8 subjects treated, the de-escalation will be triggered. The same rule applies to stopping in the de-escalated cohort, if appropriate.

12.3 Safety endpoints

All subjects who receive experimental treatment (GD2CART infusion) will be considered evaluable and will be analyzed for safety and efficacy.

Subjects not treated for any reason will be included in the disposition tabulation but will be considered inevaluable and excluded from the safety and efficacy analysis.

The safety and tolerability of GD2CART regimen will be assessed by:

- Suspected adverse events, and
- Suspected serious adverse events

As evidenced by:

  - Changes in clinical laboratory tests (clinical chemistry, hematology, etc).
  - Changes in vital signs (blood pressure, pulse, respiratory rate and body temperature).
  - Changes in physical exams. Signs and symptoms assessed may require additional testing as clinically indicated such as ECG, PFT, radiographic studies, etc.
  - Subject reported signs and symptoms

Safety data will be analyzed per standard methods and interpreted descriptively. Safety data will be summarized for each dose level separately and for the dose cohorts as a whole. Adverse events will be assessed using the CTCAE version 5.0 and Appendix B, **Section 13.2** for type and severity of event. Serious Adverse Events will be summarized for the targeted dose level. Reasons for discontinuation of study therapy will be tabulated.

Laboratory testing includes hematology, serology, serum chemistry, and urinalysis. Baseline laboratory testing will be those results obtained prior to initiating the conditioning lymphodepletion chemotherapy regimen. The study will utilize local lab for all clinical laboratory testing. Laboratory data will be tabulated based on the following result class.

- Normal: result is within the local lab normal range
- Abnormal: result is either higher or lower than the normal range

All abnormal values will be assessed for clinical significance; only clinically significant laboratory values will be captured in the case report form.

Vital signs collected immediately prior to receiving study drug will be the baseline vital signs. Observed vital sign values and change from baseline in vital signs at each visit will be summarized without formal statistical testing.

Vital sign result may also be tabulated based on the following result class.

- Normal: result is within the normal range
- Abnormal: result is either higher or lower than the normal range

All abnormal values will be assessed for clinical significance; clinical significance will be captured in the case report form. Number and percent of subjects within each result class will be tabulated by time point for each vital sign.

Findings of physical examinations will be tabulated by dose cohorts without formal statistical analysis.

12.4 Efficacy Endpoint

- ✓ In a preliminary manner, assess efficacy of GD2CART at the RP2D in children and young adults with H3K27M DIPG and children and young adults with spinal H3 K27M-mutant DMG.

The main goal for the dose expansion portion of the trial is to collect data about safety and tolerability from a larger group of patients receiving therapy at RP2D. A secondary goal is to collect data about efficacy such as overall survival of children and young adults with H3K27M DIPG, in order to compute point, interval and quantile estimates. Any patients from the dose escalation cohort whose disease is amenable for response evaluation and who were treated at the MTD/RP2D will be included in the expansion cohort numbers. Assuming 20% censoring and 10% loss-to-followup, enrollment of 20 evaluable patients with H3K27M DIPG would provide our study 80% power to detect a three-fold increase in median overall survival time (from 11 months to 33 months) with a 5% type I error rate. This is within both the total study time of 36 months (3 years) and the long-term follow-up time. It is possible that a lesser increase in median overall survival time would be adequate for future study of this regimen in this patient population, depending upon safety and feasibility. Thus the efficacy parameter will be regarded as a secondary objective for the trial.

The number of subjects with spinal DMG is not anticipated to be significant, hence the clinical activity in up to 10 subjects with spinal DMG receiving cell therapy will be reported separately and descriptively, including progression free survival (PFS) and overall survival (OS).

In addition, clinical response will be evaluated at Day 28, 3 months, and every 3 months up to 1 year, as per section **9**, in all subjects until disease progression at which time, subjects will be followed as per section **5.5.6** until off-study criteria are met.

12.5 Capacity for AP1903 to mediate clearance of genetically engineered cells and resolve toxicity

Subjects who have unacceptable toxicity, defined as life-threatening/grade 4 toxicity believed by the investigators to cause substantial risk to the subject, which is possibly, probably or definitely related to the cellular therapy, that triggers administration of AP1903 will have the levels of CAR+ T cells in the blood measured at the time of AP1903 administration, then 1 hour, 2 hours, 4 hours, 8 hours, 24 hours and 48 hours after AP1903 dosing. The changes in CAR+ T cells will be reported descriptively. Patients will also be monitored clinically for changes in

symptomatology using standard approaches and the changes will be documented and reported descriptively.

12.6 PROTOCOL STOPPING RULES

The study will be halted pending discussions with the FDA, IRB and APB if any of the following conditions are met:

a. Two DLTs occurs in dose -1 cohort in either subject group (subjects with DIPG or spinal DMG).
b. Development of EBV lymphoma or polyclonal lymphoproliferative disease (PLPD).
c. Any Grade 5 event at least possibly related to the research regimen.
d. If the targeted number of cells cannot be produced in 3 of the first 6 subjects or 5 of the first 10 subjects on this trial, further enrollment will be paused pending evaluation of the manufacturing process regardless of the safety evaluation.

12.7 EXPLORATORY ANALYSIS

- ✓ Measure expansion/persistence of adoptively transferred GD2CART in the CSF and blood and correlate this with antitumor effects.
- ✓ Conduct analyses of the manufactured T cell product and blood and CSF post-infusion to identify biomarkers associated with enhanced CAR T cell expansion, persistence and/or phenotype.
- ✓ Assess whether changes in the level of ctDNA in the cerebrospinal fluid can provide prognostic information and/or information regarding clonal evolution of DIPG over time.
- ✓ Evaluate whether antigen expression or tumor microenvironment are correlated with response to CAR T cell.

12.7.1 Persistence of GD2CART Analyses

- ✓ Measure expansion/persistence of adoptively transferred GD2CART in the CSF and blood and correlate this with antitumor effects.

Peripheral blood and CSF will be collected when available and separately analyzed for the presence of GD2CART. The percentage of all CD3+ cells in a sample that are positive by flow cytometry for GD2-CAR containing T cells will be analyzed and reported as time from T cell infusion. Correlation analyses will be performed to determine relationship to responders vs. non-responders.

12.7.2 Conduct analyses of the manufactured T cell product and blood and CSF post-infusion to identify biomarkers associated with enhanced CAR T cell expansion, persistence and/or phenotype.

Measurements of expansion and persistence in subjects with DIPG and DMG who have received GD2CART will be analyzed to identify biomarkers associated with expansion, persistence and/or phenotype. It will be difficult to draw conclusions from this analysis, but rather this analysis will be hypothesis generating.

12.7.3 Assess whether changes in the level of ctDNA in the cerebrospinal fluid can provide prognostic information and/or information regarding clonal evolution of DIPG over time.

12.7.4 Evaluate whether antigen expression or tumor microenvironment are correlated with response to CAR T cell.

This testing will provide an assessment of the tumor immune environment and will contribute to understanding mechanisms of tumor evasion through modulation of antigen density and/or a suppressive tumor environment.

12.8 SAMPLE SIZE

12.8.1 Accrual estimates

We anticipate enrollment of 1-2 subjects per month during the dose escalation portion of this study, but expect to enroll 2-3 subjects per month during enrollment to the expansion cohorts, given the number of subjects with DIPG and DMG treated in the local/regional area. The recruitment period for this study is expected to be 1.5-2 years. Subjects will be followed for 1 year post treatment. The total duration of this study to meet primary objectives is expected to be 2.5-3 years of active treatment and short term follow up, and a total of 17 years of long term follow up after the last subject completes study therapy.

12.8.2 Sample size justification

The primary objectives of this study are safety and feasibility. Initially 3-6 evaluable subjects with H3K27M DIPG may be enrolled sequentially in 3 dose levels of GD2CART to establish MTD/RP2D, for a minimum of 4 subjects and a maximum of 18.

Once RP2D is established, up to a total of 20 evaluable subjects with DIPG and 10 evaluable subjects with spinal H3 K27M-mutant DMG will be treated at the RP2D dose (including any from the dose escalation phase) to further assess safety and perform a preliminary analysis of clinical activity. In addition, we will allow for replacement of 6 total inevaluable subjects (subjects enrolled but who cannot receive cells, either due to physical deterioration or withdrawn consent during cell growth).

Thus, a maximum of 54 (18 in the dose escalation + 24 in the expansion cohorts + 6 replaced for manufacturing feasibility and 6 replaced for inevaluability following enrollment) subjects may be enrolled to determine the objectives of this study.

13 APPENDICES

13.1 APPENDIX A: APPENDIX A: PERFORMANCE STATUS CRITERIA

**PERFORMANCE STATUS CRITERIA** *Karnofsky and Lansky performance scores are intended to be multiples of 10.*					
**ECOG (Zubrod)**		**Karnofsky**		**Lansky**	
**Score**	**Description**	**Score**	**Description**	**Score**	**Description**
0	Fully active, able to carry on all pre-disease performance without restriction.	100%	Normal, no complaints, no evidence of disease.	100%	Fully active, normal.
		90%	Able to carry on normal activity; minor sighs of symptoms of disease.	90%	Minor restrictions in physically strenuous activity.
1	Restricted in physically strenuous activity but ambulatory, able to carry out light or sedentary work, e.g., light housework, office work.	80%	Able to carry on normal activity with effort; some signs or symptoms of disease.	80%	Active, but tires more quickly.
		70%	Cares for self, unable to carry on normal activity or do active work.	70%	Both greater restriction of, and less time spent in, play activities.
2	Ambulatory and capable of all self-care but unable to carry out any work activities. Up and about more than 50% of waking hours.	60%	Requires occasional assistance but is able to care for most of own needs.	60%	Up and around, but minimal active play; keeps busy with quieter activities.
		50%	Requires considerable assistance and frequent medical care.	50%	Gets dressed, but lies around much of the day; no active play; able to participate in quiet play and activities.
3	Capable of only limited self-care, confined to bed or chair more than 50% of waking hours	40%	Disabled; requires special care and assistance.	40%	Mostly in bed; participates in quiet activities.
		30%	Severely disabled; hospitalization indicated, although death not imminent.	30%	In bed; needs assistance even for quiet play.
4	Completely disabled. Cannot carry on any self-care. Totally confined to a bed or chair	20%	Very ill; hospitalization necessary; active supportive treatment required.	20%	Often sleeping; play entirely limited to very passive activities.
		10%	Moribund, fatal process progressing rapidly	10%	No play; does not get out of bed
5	Dead	0%	Patient expired	0%	Unresponsive; Dead

13.2 APPENDIX B: GUIDELINES TOXICITY ASSESSMENT AND MANAGEMENT

13.2.1 Guidelines for Grading Suspected Cytokine Release Syndrome according to ASTCT CRS Consensus Grading[52]#

CRS Parameter	Grade 1	Grade 2	Grade 3	Grade 4
**Fever**[†]	Temperature ≥ 38° C	Temperature ≥ 38° C	Temperature ≥ 38° C	Temperature ≥ 38° C

With either:

**Hypotension**	None	Not requiring vasopressors	Requiring one vasopressor with or without vasopressin	Requiring multiple vasopressors (excluding vasopressin)

And/or[§]:

**Hypoxia**	None	Requiring low-flow nasal cannula^ or blow-by	Requiring high-flow nasal cannula, facemask, non-rebreather mask, or Venturi mask	Requiring positive pressure (e.g.: CPAP, BiPAP, intubation and mechanical ventilation

CPAP: Continuous positive airway pressure; BiPAP: Bilevel positive airway pressure

Organ toxicities associated with CRS may be graded according to CTCAE v5.0 but they do not influence CRS grading.

[†] Fever is defined as temperature ≥ 38° C not attributable fo any other cause. In patients who have CRS then receive anti-yretics or anti-cytokine therapy such as tocilizumab or steroids, fever is no long required to grade subsequent CRS severity. In this case, CRS grading is driven by hypotension and/or hypoxia.

[§] CRS grade is determined by the more severe event: hypotension or hypoxia not attributable to any other cause. For example, a patient with a temperature of 39.5 ° C, hypotension requiring one vasopressor and hypoxia requiring low-flow nasal cannula is classified as having Grade 3 CRS.

^ Low-flow nasal cannula is defined as oxygen delivered at ≤ 6 liters/minute. Low flow also includes blow-by oxygen delivery, sometimes used in pediatrics. High-flow nasal cannula is defined as oxygen delivered at > 6 liters/minute.

13.2.2 Guidelines for Treating Suspected Cytokine Release Syndrome

Grade 1	*Symptoms are not life threatening and require symptomatic treatment only*<br>Treatment:<br>✓ Assess for infection<br>✓ Treat fever and neutropenia if present, monitor fluid balance, antipyretics, analgesics as needed.
Grade 2	*Symptoms require and respond to moderate intervention*<br>Treatment:<br>✓ As above for Grade 1<br>✓ Monitor organ function closely (incl. cardiac, respiratory, renal, neurologic, liver)
Grade 3<br>or Grade 2 with co-morbidity	*Symptoms require and respond to aggressive intervention*<br>Treatment:<br>✓ As above for Grade 2<br>✓ Consider tocilizumab with or without corticosteroids<br>✓ Consider vasopressors, oxygen supplementation
Grade 4	*Life-threatening symptoms*<br>Treatment:<br>✓ As above<br>✓ Positive pressure airway assistance or Mechanical ventilation<br>✓ Multiple vasopressors or high dose vasopressors

[1]high dose vasopressor doses shown in Table 2.

Tocilizumab is administered at a dose of 4-8 mg/kg infused over 1 hour (not to exceed 800 mg).

Table 2. High-Dose Vasopressors (all doses are required for ≥ 3 hours)

Norepinephrine monotherapy	≥ 0.2 mcg/kg/min
Dopamine monotherapy	≥ 10 mcg/kg/min
Phenylephrine monotherapy	≥ 200 mcg/min
Epinephrine monotherapy	≥ 0.1 mcg/kg/min
If on vasopressin	[1]Vasopressin + NE equivalent of ≥ 0.1mcg/kg/min
If on combination vasopressors (not vasopressin)	[1]Norepinephrine equivalent of ≥ 20 mcg/kg/min

[1]*VASST Trial Vasopressor Equivalent Equation:*

Norepinephrine equivalent dose = [norepinephrine (mcg/min)] + [dopamine (mcg/kg/min) ÷ 2] + [epinephrine (mcg/min)] + [phenylephrine (mcg/min) ÷10]

13.2.3 Encephalopathy assessment tools for grading Immune effector Cell-Associated Neurotoxicity Syndrome (ICANS)

13.2.3.1 Immune effector Cell-associated Encephalopathy (ICE) Assessment

Directions: Answer whether each task was performed correctly (Not Done/Yes/No). If you answer YES put 1 in the Score column; If you answer NO (or Not Done) put 0 in the Score column.

Tasks	Performed correctly?	Score
**Orientation**		
1. What is the current year?		**0**
2. What is the current month?		**0**
3. What is the current city?		**0**
4. What hospital are you in?		**0**
**Naming**		
5. Name this object (*point to an object in the room)*		**0**
6. Name this object (*point to an object in the room)*		**0**
7. Name this object (*point to an object in the room)*		**0**
**Following commands**		
8. Show me (insert object, e.g. 2 fingers) or Close your eyes and stick out your tongue.		**0**
**Writing**		
9. Write a simple sentence (*provide paper and pencil)*		**0**
**Attention**		
10. Count backwards from 100 in 10's.		**0**
**Total Score**		**0**

Scoring ICE

No impairment: Score 10

Grade 1 ICANS: Score 7-9

Grade 2 ICANS: Score 3-6

Grade 3 ICANS: Score 0-2

Grade 4 ICANS: Score 0 due to patient unarousable and unable to perform ICE assessment

13.2.3.2 ASBMT Immune effector Cell-Associated Neurotoxicity Syndrome (ICANS) Consensus Grading for Adults

Neurotoxicity Domain	Grade 1	Grade 2	Grade 3	Grade 4
**ICE Score^**	7-9	3-6	0-2	0 (patient is unarousable and unable to perform ICE)
**Depressed level of consciousness⁑**	Awakens spontaneously	Awakens to voice	Awakens only to tactile stimulus	Patient is unarousable or requires vigorous or repetitive tactile stimuli to arouse. Stupor or coma
**Seizure**	N/A	N/A	Any clinical seizure focal or generalized that resolves rapidly; or<br>Non-convulsive seizures on EEG that resolve with intervention	Life-threatening prolonged seizure (> 5 min); or<br>Repetitive clinical or electrical seizures without return to baseline in between.
**Motor findings§**	N/A	N/A	N/A	Deep focal motor weakness such as hemiparesis or paraparesis
**Raised ICP / Cerebral edema**	N/A	N/A	Focal/local edema on neuroimaging#	Diffuse cerebral edema on neuroimaging;<br>Decerebrate or decorticate posturing; or<br>Cranial nerve VI palsy; or<br>Papilledema; or<br>Cushing's triad

ICANS grade is determined by the most severe event (ICE score, level of consciousness, seizure, motor findings, raised ICP/cerebral edema) not attributable to any other cause. For example, a patient with and ICE score of 3 who has a generalized seizure is classified as having a Grade 3 ICANS.

^A patient with an ICE score of 0 may be classified as having Grade 3 ICANS if the patient is awake with global aphasia. But a patient with an ICE score of 0 may be classified as having a Grade 4 ICANS if the patient is unarousable.

⁑Depressed level of consciousness should be attributable to no other cause (e.g. no sedating medication).

§Tremors and myoclonus associated with immune effector cell therapies may be graded according to CTCAE v5.0 but they do not influence ICANS grading.

#Intracranial hemorrhage with or without associated edema is not considered a neurotoxicity feature and is excluded from ICANS grading. It may be graded according to CTCAE v5.0.

ICE: Immune effector Cell-associated Encephalopathy; **ICP:** Intracranial pressure; **EEG:** electroencephalogram

13.2.3.3 Encephalopathy Assessment for Children < 12 years using Cornell Assessment of Pediatric Delirium **(CAPD)**[65],[66]

Adapted from Taube et al.[65] and reproduced in [52] with permission from Wolters Kluwer.

**Answer the following based on interactions with the child over the course of the shift**	**Never**<br>**4**	**Rarely**<br>**3**	**Sometimes**<br>**2**	**Often**<br>**1**	**Always**<br>**0**
1. Does the child make eye contact with the caregiver?					
2. Are the child's actions purposeful?					
3. Is the child aware of his/her surroundings?					
4. Does the child communicate needs and wants?					
	**Never**<br>**0**	**Rarely**<br>**1**	**Sometimes**<br>**2**	**Often**<br>**3**	**Always**<br>**4**
5. Is the child restless?					
6. Is the child consolable?					
7. Is the child underactive-very little movement while awake?					
8. Does it take the child a long time to respond to interactions?					

For patients age 1-2 year, the following serve as guidelines to the corresponding questions:

1. Holds gaze. Prefers primary parent. Looks at speaker.
2. Reaches and manipulates objects, tries to change position, if mobile may try to get up.
3. Prefers primary parent, upset when separated from preferred caregivers. Comforted by familiar objects (i.e., blanket or stuffed animal)
4. Uses single words or signs
5. No sustained calm state
6. Not soothed by usual comforting actions, for example, singing, holding, talking, and reading
7. Little if any play, efforts to sit up, pull up, and if mobile crawl or walk around
8. Not following simple directions. If verbal, not engaging in simple dialogue with words or jargon.

13.2.3.4 ASTCT Immune effector Cell-Associated Neurotoxicity Syndrome (ICANS) Consensus Grading for Children

Neurotoxicity Domain	Grade 1	Grade 2	Grade 3	Grade 4
**ICE Score for children > 12 years^**	7-9	3-6	02	0 (patient is unarousable and unable to perform ICE)
**CAPD score for children ≤ 12 years**	<9	<9	≥9	Unable to perform CAPD
**Depressed level of consciousness⁑**	Awakens spontaneously	Awakens to voice	Awakens only to tactile stimulus	Patient is unarousable or requires vigorous or repetitive tactile stimuli to arouse. Stupor or coma
**Seizure (any age)**	N/A	N/A	Any clinical seizure focal or generalized that resolves rapidly; or<br>Non-convulsive seizures on EEG that resolve with intervention	Life-threatening prolonged seizure (> 5 min); or<br>Repetitive clinical or electrical seizures without return to baseline in between.
**Motor weakness (any age)§**	N/A	N/A	N/A	Deep focal motor weakness such as hemiparesis or paraparesis
**Raised ICP / Cerebral edema (any age)**	N/A	N/A	Focal/local edema on neuroimaging#	Decerebrate or decorticate posturing; or<br>Cranial nerve VI palsy; or<br>Papilledema; or<br>Cushing's triad; or<br>Signs of diffuse cerebral edema on neuroimaging

ICANS grade is determined by the most severe event (ICE or CAPD score, level of consciousness, seizure, motor findings, raised ICP/cerebral edema) not attributable to any other cause.

^A patient with an ICE score of 0 may be classified as having Grade 3 ICANS if the patient is awake with global aphasia. But a patient with an ICE score of 0 may be classified as having a Grade 4 ICANS if the patient is unarousable.

⁑Depressed level of consciousness should be attributable to no other cause (e.g. no sedating medication).

§Tremors and myoclonus associated with immune effector cell therapies may be graded according to CTCAE v5.0 but they do not influence ICANS grading.

#Intracranial hemorrhage with or without associated edema is not considered a neurotoxicity feature and is excluded from ICANS grading. It may be graded according to CTCAE v5.0.

ICE: Immune effector Cell-associated Encephalopathy; **CAPD:** Cornell Assessment of Pediatric Delirium; **ICP:** Intracranial pressure; **EEG:** electroencephalogram

13.2.3.5 Protocol for managing neurologic symptoms in patients receiving GD2CART

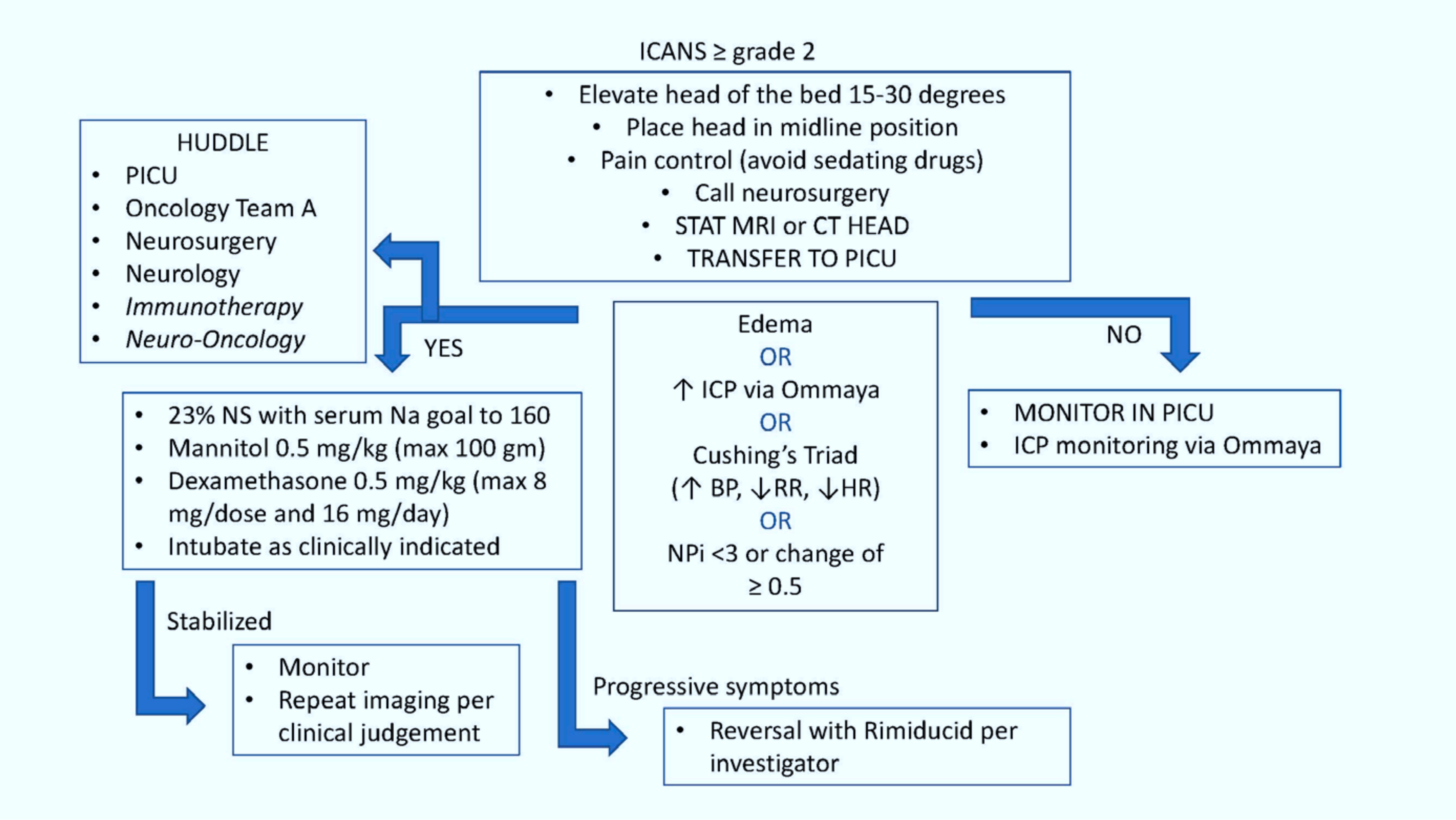

13.3 Appendix C: Calculation of Weight for Cell Dose Calculation in Morbidly Obese Candidates

Formulation for deriving the weight to be used in targeting cell doses in morbidly obese cell candidates.

1. **Definition**

Obesity is defined as a BMI > 30.

BMI = wgt (kg) / [hgt (M)]2

2. **Calculation of ideal body weight** is performed using the standard, published formula:

Male: 50 + 2.3(Hgt - 60) where Hgt is in inches, and the result is expressed in kg.

ex. The ideal weight of a 5'10" male = 50 + 2.3(10) = 73 kg.

Female: 45.5 + 2.3(Hgt - 60), where height is in inches, and the result is in kg.

3. **Calculation of the “practical weight.”**

Calculate the midway point, halfway between the actual and ideal body weights (ie the average of the two numbers). This is the "practical weight" to be used in calculating the targeted cell dose.

4. **Example**:

Subject’s actual weight = 143 kg.

Subject’ s actual height 173 cm = 69 in

BMI = 48

IBW formula = 50 + 2.3(9) = 70.7 kg

Midway point between 70.0 and 143 = 107 kg.

The weight we would use in targeting cell dose is 107 kg.

1 dose by weight with adjustment:

- IBW + 50%(Weight-IBW)
- Practical body weight = (IDW + actual BW)/2

2 Formula

IBW (men)

- 52 kg + 1.9 kg/inch above 5 feet
- 50 kg + 2.39 (height in inches – 60)

IBW (women)

- 49 kg + 1.7 kg/inch above 5 feet
- 45.5 kg + 2.39 (height in inches – 60)

13.4 Appendix D: Monitoring Gene Therapy Trials: Replication Competent Retrovirus (RCR)

Subject Testing for RCR PCR

Subject blood samples will be obtained at the following time points:

1. Prior to cell infusion
2. 3 months(± 2 weeks)
3. 6 months (± 1 month)
4. 1 year (±2 months)
5. Archive samples annually thereafter for 5 years (+/- 3 months), if previous samples have been negative.

All Samples are scheduled from the most recent cell infusion.

Procedures:

Samples will be collected from subjects returning to clinic or collected by a local physician and shipped and sent to Indiana University according to the Laboratory Manual.

Document Retention

1. RCR Reports from Indiana University will be sent to the Sponsor Investigator; the study coordinator will obtain the report from the study PI.
2. Scan the report and save results of RCR PCR (or S+L-) testing in a password protected subject file associated with the gene therapy protocol. Hard copies will be retained in the subject's research record in a locked file cabinet.
3. Record the sample result on the Master S+L-/RCR PCR Spreadsheet saved in the study files.

13.5 APPENDIX E: DRAFT LETTER AND QUESTIONNAIRE TO SUBJECTS FOR LONG TERM FOLLOW-UP FOR DELAYED ADVERSE EVENTS

[date]

[name and address]

Dear [subject name],

You have participated in a clinical research study that requires that the study doctors and nurses monitor your health for 15 years. In addition to the annual visits you will be attending, **we would like for you to report certain events listed below to your study doctor or nurse if they occur**:

1. Your doctor tells you that you have been diagnosed with any new type of cancer, including blood disorders such as leukemia or lymphoma (this would be separate from your cancer diagnosis).
2. You develop loss of feeling in any part of your body, especially hands and feet; you develop a loss of control of any body part (arms, legs…); you have a seizure; you experience memory loss. In addition, if you experience a worsening of any of the symptoms listed, please contact your study nurse or doctor. These types of symptoms are called neurological disorders. If your primary doctor or specialist tells you that you have developed neurological symptoms, contact your study doctor or nurse.
3. You develop arthritis or autoimmune disease, or worsening of any previously experienced arthritis or autoimmune disease which you were experiencing prior to participation in the study. If you are experiencing symptoms of arthritis or have been told by your doctor that you have an autoimmune disease, contact your study doctor or nurse.

Please complete the attached questionnaire and return it in the Fed-Ex envelope to the study coordinator.

If you experience any of the events listed above during the upcoming year, please contact your study physician or the study nurse listed below as soon as you can. They may ask you questions about your health and will record your symptoms/disease and then monitor your health if they decide that it is necessary. When you call, please mention that you participated in a gene therapy clinical trial at the < put your institution here> . Your subject identification number under this protocol is (#XXX).

Study Coordinator:

Name
Address
Phone
Email

If you have any questions about this letter or the follow up procedures for the study itself, please do not hesitate to contact the above study nurse.

Thank you for your continued participation in our clinical research study. Best regards,

[study coordinator]

Questionnaire to Subjects for Long Term Follow-up for Delayed Adverse Events

Subject Identification [put subject study number here]

Within the past year, have you:

1. **Had any problems with your health?** ☐ YES ☐ NO

If Yes, please explain: ____________________

____

2. **Required any hospitalizations?** ☐ YES ☐ NO

If Yes, please describe when and the reason: ____________________

____

____

3. **Seen any healthcare provider?** ☐ YES ☐ NO

If Yes, please describe when and the reason: ____________________

____

____

4. **Started on any new medications?** ☐ YES ☐ NO

If Yes, please list: ____________________

____

5. **Developed any new conditions or illnesses?** ☐ YES ☐ NO

If Yes, please describe: ____________________

____

Please share any other new health concerns or problems:

____

When you have completed this questionnaire, please return it to:

Study Coordinator:

Name
Address
Phone
Fax
Email

A pre-addressed stamped envelope has been enclosed for your convenience, if you choose to mail this questionnaire. We will also accept faxed or e-mailed completed questionnaires as well.

Thank you very much for your participation.

13.6 APPENDIX F: PHYSICIAN (LOCAL MEDICAL PROVIDER) LETTER

[date]

[name and address]

Dear [physician name],

Your subject [subject name] has participated in a clinical research study that requires 15 year monitoring for adverse events. To aid in reporting adverse events that are possible related to the clinical research study, we are asking the subjects on our research study to designate a primary care or infectious disease physician that may help in the monitoring and reporting of adverse events. Your subject has designated you. **If upon any of your visits with your subject, any of the following events are reported or discovered, please contact the study nurse or physician as soon as possible**:

1. New malignancies
2. New incidence of exacerbation of a pre-existing neurologic disorder
3. New incidence or exacerbation of a prior rheumatologic or other autoimmune disorder
4. New incidence of a hematologic disorder.

If your subject experiences any of these events, please contact the study coordinator below as soon as you can so that they can record the event and then monitor your subject's health if necessary. When you call, please mention that the subject has participated in a gene therapy clinical trial in the < designate location, institution and sponsor investigator> .

Study Coordinator

Name

Address

Phone

Email

If you have any questions about this letter or the study itself, please do not hesitate to contact the above study nurse.

Thank you for your support in helping us to monitor for delayed adverse events. Best regards,

13.7 Appendix G: Correlative Sample Schedule

GD2 CAR Trial Correlative Sample Collection Matrix

Priority	mL	type	purpose	Apheresis	Product	Baseline (prior to LD	D0	D7 (± 2 d)	D14 (± 2 d)	D21 (± 4 d)	D28 (± 7 d)	M2 (± 2 w)	M3 (± 1 m)	M4 (± 2 w)	M5 (± 2 w)	M6 (± 1 m)	M9 (± 1 m)	M12 (± 2 m)	At Progression	Minimum Cell Number[5]
		**Apheresis Product**																		
		collected by manufacturer		X																
		collected by Stanford									X[1]		X[1]			X[1]	X[1]	X[1]		
		**CAR T cell Product**																		
		collected by manufacturer	baseline measurements for correlatives		X															
		**PBMCs**																		
1	4	Green top (Heparin)	CAR-FACS flow panel		X		X	X	X	X	X	X	X	X	X	X	X	X	X	2 x10^6
2	5	Lavender top (EDTA)	CAR qRT-PCR		X		X	X	X	X	X	X	X	X	X	X	X	X	X	
3	5	Green top (Heparin)	CyTOF	X	X	X		X	X[2]	X	X[2]	X	X[2]	X	X	X[2]	X	X	X[2]	4-5 x10^6
4	5	Lavender top (EDTA)	TCR sequencing/ CAR-T cell fat	X	X				X		X		X			X	X	X	X	
5	5	Lavender top (EDTA)	ATAC-Seq/RNA-Seq	X	X				X		X		X			X	X	X	X	
6	5	Green top (Heparin)	Sample banking			X	X	X	X	X	X	X	X	X	X	X	X	X	X	
7	5	Lavender top (EDTA)	Sample banking			X	X	X	X	X	X	X	X	X	X	X	X	X	X	
		**Plasma**																		
	5	Streck Cell-Free DNA Tubes	Cell-free DNA[3]			X			X		X		X			X	X	X	X	
		Lavender top (EDTA)	Cytokines[3,4]				X	X	X	X	X									
		Lavender top (EDTA)	Sample banking[3]			X	X	X	X	X	X	X	X	X	X	X	X	X	X	
		**CSF**																		
1	1	Falcon tube	CAR-FACS flow panel[4,6]			X		X	X	X	X	X[6]	X[6]	X[6]	X[6]	X[6]			X[6]	2 x10^6
2	1	Falcon tube	Cytokines[3,4,6]			X		X	X	X	X									
3	1	Falcon tube	CyTOF[6]			X		X	X	X	X	X[6]	X[6]	X[6]	X[6]	X[6]			X[6]	4-5 x10^6
4	1	CFD tube	Cell-free DNA[3,6]			X			X		X	X[6]	X[6]	X[6]	X[6]	X[6]			X[6]	
5	1	Falcon tube	Sample banking[4,6]			X		X	X	X	X	X[6]	X[6]	X[6]	X[6]	X[6]			X[6]	
		**Tumor**																		
1		FFPE	Sample banking			X[6]													X[6]	
2		Frozen Tissue	Sample banking			X[6]													X[6]	
Total Blood Volume per visit (mL)																				
						24	14	19	39	19	39	19	39	19	19	39	34	34	39	

Notes
1. A small volume apheresis (1-2 blood volumes) may be collected in lieu of peripheral blood tubes listed below
2. If possible, collect 10 mL
3. Plasma separated from Lavendar top (EDTA) collection tube
4. Collect at any time of neurotoxicity and at Day 28 (for subjects with CSF involvement), at discretion of investigator
5. If patient sample volume limits are reached, collection tubes may be volume reduced and/or pooled and aliquoted based on minimum cell number requirements and the correlative priority number
6. When available, plan to collect these samples

Target sample volumes are ideal, and may be adjusted for age, size and condition of patient. If blood volumes are limited, testing completion will be prioritized according to **Section 8.2.2.**

14 REFERENCES

1. Louis, D.N., et al., *The 2016 World Health Organization Classification of Tumors of the Central Nervous System: a summary.* Acta Neuropathol, 2016. **131**(6): p. 803-20.
2. Khuong-Quang, D.A., et al., *K27M mutation in histone H3.3 defines clinically and biologically distinct subgroups of pediatric diffuse intrinsic pontine gliomas.* Acta Neuropathol, 2012. **124**(3): p. 439-47.
3. Wu, G., et al., *Somatic histone H3 alterations in pediatric diffuse intrinsic pontine gliomas and non-brainstem glioblastomas.* Nat Genet, 2012. **44**(3): p. 251-3.
4. Castel, D., et al., *Histone H3F3A and HIST1H3B K27M mutations define two subgroups of diffuse intrinsic pontine gliomas with different prognosis and phenotypes.* Acta Neuropathol, 2015. **130**(6): p. 815-27.
5. Warren, K.E., *Diffuse intrinsic pontine glioma: poised for progress.* Front Oncol, 2012. **2**: p. 205.
6. Ostrom, Q.T., et al., *CBTRUS Statistical Report: Primary brain and other central nervous system tumors diagnosed in the United States in 2010-2014.* Neuro Oncol, 2017. **19**(suppl_5): p. v1-v88.
7. Cooney, T., et al., *Contemporary survival endpoints: an International Diffuse Intrinsic Pontine Glioma Registry study.* Neuro Oncol, 2017. **19**(9): p. 1279-1280.
8. Fisher, P.G., et al., *A clinicopathologic reappraisal of brain stem tumor classification. Identification of pilocystic astrocytoma and fibrillary astrocytoma as distinct entities.* Cancer, 2000. **89**(7): p. 1569-76.
9. Bouffet, E., et al., *Radiotherapy followed by high dose busulfan and thiotepa: a prospective assessment of high dose chemotherapy in children with diffuse pontine gliomas.* Cancer, 2000. **88**(3): p. 685-92.
10. Cohen, K.J., et al., *Temozolomide in the treatment of children with newly diagnosed diffuse intrinsic pontine gliomas: a report from the Children's Oncology Group.* Neuro Oncol, 2011. **13**(4): p. 410-6.
11. Dunkel, I.J., B. O'Malley, and J.L. Finlay, *Is there a role for high-dose chemotherapy with stem cell rescue for brain stem tumors of childhood?* Pediatr Neurosurg, 1996. **24**(5): p. 263-6.
12. Finlay, J.L., et al., *High-dose multi-agent chemotherapy followed by bone marrow 'rescue' for malignant astrocytomas of childhood and adolescence.* J Neurooncol, 1990. **9**(3): p. 239-48.
13. Hargrave, D., U. Bartels, and E. Bouffet, *Diffuse brainstem glioma in children: critical review of clinical trials.* Lancet Oncol, 2006. **7**(3): p. 241-8.

14. Jalali, R., et al., *Prospective evaluation of radiotherapy with concurrent and adjuvant temozolomide in children with newly diagnosed diffuse intrinsic pontine glioma.* Int J Radiat Oncol Biol Phys, 2010. **77**(1): p. 113-8.

15. Jansen, M.H., et al., *Diffuse intrinsic pontine gliomas: a systematic update on clinical trials and biology.* Cancer Treat Rev, 2012. **38**(1): p. 27-35.

16. Jennings, M.T., et al., *Preradiation chemotherapy in primary high-risk brainstem tumors: phase II study CCG-9941 of the Children's Cancer Group.* J Clin Oncol, 2002. **20**(16): p. 3431-7.

17. Maude, S.L., *Tisagenlecleucel in pediatric patients with acute lymphoblastic leukemia.* Clin Adv Hematol Oncol, 2018. **16**(10): p. 664-666.

18. Neelapu, S.S., Frederick L. Locke, Nancy L. Bartlett, Lazaros Lekakis, David Miklos, Caron A. Jacobson, Ira Braunschweig, Olalekan Oluwole, Tanya Siddiqi, Yi Lin, John Timmerman, Patrick J. Stiff, Jonathan Friedberg, Ian Flinn, Andre Goy, Mitchell Smith, Abhinav Deol, Umar Farooq, Peter McSweeney, Javier Munoz, Irit Avivi, Januario E. Castro, Jason R. Westin, Julio C. Chavez, Armin Ghobadi, Krishna V. Komanduri, Ronald Levy, Eric D. Jacobsen, Patrick Reagan, Adrian Bot, John M. Rossi, Lynn Navale, Yizhou Jiang, Jeff S. Aycock, Meg Elias, Jeff Wiezorek and William Y. Go, *Kte-C19 (anti-CD19 CAR T Cells) Induces Complete Remissions in Patients with Refractory Diffuse Large B-Cell Lymphoma (DLBCL): Results from the Pivotal Phase 2 Zuma-1*, in *Blood*. 2016, American Society of Hematology: San Diego, CA.

19. Yu, A.L., et al., *Anti-GD2 antibody with GM-CSF, interleukin-2, and isotretinoin for neuroblastoma.* N Engl J Med, 2010. **363**(14): p. 1324-34.

20. Mody, R., et al., *Irinotecan-temozolomide with temsirolimus or dinutuximab in children with refractory or relapsed neuroblastoma (COG ANBL1221): an open-label, randomised, phase 2 trial.* Lancet Oncol, 2017. **18**(7): p. 946-957.

21. Mount, C.W., et al., *Potent antitumor efficacy of anti-GD2 CAR T cells in H3-K27M(+) diffuse midline gliomas.* Nat Med, 2018. **24**(5): p. 572-579.

22. Gubin, M.M., et al., *Tumor neoantigens: building a framework for personalized cancer immunotherapy.* J Clin Invest, 2015. **125**(9): p. 3413-21.

23. Rizvi, N.A., et al., *Cancer immunology. Mutational landscape determines sensitivity to PD-1 blockade in non-small cell lung cancer.* Science, 2015. **348**(6230): p. 124-8.

24. Merchant, M.S., et al., *Phase I Clinical Trial of Ipilimumab in Pediatric Patients with Advanced Solid Tumors.* Clin Cancer Res, 2016. **22**(6): p. 1364-70.

25. Davis, R.J., et al., *Anti-PD-L1 Efficacy Can Be Enhanced by Inhibition of Myeloid-Derived Suppressor Cells with a Selective Inhibitor of PI3Kdelta/gamma.* Cancer Res, 2017. **77**(10): p. 2607-2619.

26. Pugh, T.J., et al., *The genetic landscape of high-risk neuroblastoma.* Nat Genet, 2013. **45**(3): p. 279-84.

27. Grasso, C.S., et al., *Functionally defined therapeutic targets in diffuse intrinsic pontine glioma.* Nat Med, 2015. **21**(7): p. 827.

28. Mackay, A., et al., *Integrated Molecular Meta-Analysis of 1,000 Pediatric High-Grade and Diffuse Intrinsic Pontine Glioma.* Cancer Cell, 2017. **32**(4): p. 520-537 e5.

29. Lee, D.W., et al., *T cells expressing CD19 chimeric antigen receptors for acute lymphoblastic leukaemia in children and young adults: a phase 1 dose-escalation trial.* Lancet, 2015. **385**(9967): p. 517-28.

30. Long, A.H., et al., *4-1BB costimulation ameliorates T cell exhaustion induced by tonic signaling of chimeric antigen receptors.* Nat Med, 2015. **21**(6): p. 581-90.

31. Monje, M. and P.G. Fisher, *Neurological complications following treatment of children with brain tumors.* J Pediatr Rehabil Med, 2011. **4**(1): p. 31-6.

32. Weng, J., et al., *IL-15 enhances the antitumor effect of human antigen-specific CD8+ T cells by cellular senescence delay.* Oncoimmunology, 2016. **5**(12): p. e1237327.

33. Pule, M.A., et al., *Virus-specific T cells engineered to coexpress tumor-specific receptors: persistence and antitumor activity in individuals with neuroblastoma.* Nat Med, 2008. **14**(11): p. 1264-70.

34. Louis, C.U., et al., *Antitumor activity and long-term fate of chimeric antigen receptor-positive T cells in patients with neuroblastoma.* Blood, 2011. **118**(23): p. 6050-6.

35. Locatelli, F., *Phase 1 trial with GD2-directed CAR T cell therapy in neuroblastoma patients*, in *EHA-EBMT 2nd European CAR T Cell Meeting*. 2020: Barcelona, Spain.

36. Panditharatna, E., et al., *Clinically Relevant and Minimally Invasive Tumor Surveillance of Pediatric Diffuse Midline Gliomas Using Patient-Derived Liquid Biopsy.* Clin Cancer Res, 2018. **24**(23): p. 5850-5859.

37. Freeman, C.R. and J.P. Farmer, *Pediatric brain stem gliomas: a review.* Int J Radiat Oncol Biol Phys, 1998. **40**(2): p. 265-71.

38. Ostrom, Q.T., et al., *Epidemiology of Intracranial Gliomas.* Prog Neurol Surg, 2018. **30**: p. 1-11.

39. Cohen, K.J., A. Broniscer, and J. Glod, *Pediatric glial tumors.* Curr Treat Options Oncol, 2001. **2**(6): p. 529-36.

40. Langmoen, I.A., et al., *Management of pediatric pontine gliomas.* Childs Nerv Syst, 1991. **7**(1): p. 13-5.

41. Schwartzentruber, J., et al., *Driver mutations in histone H3.3 and chromatin remodelling genes in paediatric glioblastoma.* Nature, 2012. **482**(7384): p. 226-31.

42. Qin, E.Y., et al., *Neural Precursor-Derived Pleiotrophin Mediates Subventricular Zone Invasion by Glioma.* Cell, 2017. **170**(5): p. 845-859 e19.

43. Ali, N., et al., *Xenogeneic graft-versus-host-disease in NOD-scid IL-2Rgammanull mice display a T-effector memory phenotype.* PLoS One, 2012. **7**(8): p. e44219.

44. Nagaraja, S., et al., *Transcriptional Dependencies in Diffuse Intrinsic Pontine Glioma.* Cancer Cell, 2017. **31**(5): p. 635-652 e6.

45. Louveau, A., et al., *Structural and functional features of central nervous system lymphatic vessels.* Nature, 2015. **523**(7560): p. 337-41.

46. Till, B.G., et al., *Adoptive immunotherapy for indolent non-Hodgkin lymphoma and mantle cell lymphoma using genetically modified autologous CD20-specific T cells.* Blood, 2008. **112**(6): p. 2261-71.

47. Di Stasi, A., et al., *Inducible apoptosis as a safety switch for adoptive cell therapy.* N Engl J Med, 2011. **365**(18): p. 1673-83.

48. Heczey, A., et al., *CAR T Cells Administered in Combination with Lymphodepletion and PD-1 Inhibition to Patients with Neuroblastoma.* Mol Ther, 2017. **25**(9): p. 2214-2224.

49. D'Angelo, S.P., et al., *Antitumor Activity Associated with Prolonged Persistence of Adoptively Transferred NY-ESO-1 (c259)T Cells in Synovial Sarcoma.* Cancer Discov, 2018. **8**(8): p. 944-957.

50. Weber, E.W., et al., *Pharmacologic control of CAR-T cell function using dasatinib.* Blood Adv, 2019. **3**(5): p. 711-717.

51. Schade, A.E., et al., *Dasatinib, a small-molecule protein tyrosine kinase inhibitor, inhibits T-cell activation and proliferation.* Blood, 2008. **111**(3): p. 1366-77.

52. Lee, D.W., et al., *ASTCT Consensus Grading for Cytokine Release Syndrome and Neurologic Toxicity Associated with Immune Effector Cells.* Biol Blood Marrow Transplant, 2019. **25**(4): p. 625-638.

53. Davila, M.L., et al., *Efficacy and toxicity management of 19-28z CAR T cell therapy in B cell acute lymphoblastic leukemia.* Sci Transl Med, 2014. **6**(224): p. 224ra25.

54. Lee, D.W., et al., *Current concepts in the diagnosis and management of cytokine release syndrome.* Blood, 2014. **124**(2): p. 188-95.

55. Maude, S.L., et al., *Chimeric antigen receptor T cells for sustained remissions in leukemia.* N Engl J Med, 2014. **371**(16): p. 1507-17.

56. Weber, J., et al., *A phase I trial of intravenous interleukin-6 in patients with advanced cancer.* J Immunother Emphasis Tumor Immunol, 1994. **15**(4): p. 292-302.

57. Taraseviciute, A., et al., *Chimeric Antigen Receptor T Cell-Mediated Neurotoxicity in Nonhuman Primates.* Cancer Discov, 2018. **8**(6): p. 750-763.

58. Saleh, M.N., et al., *Phase I trial of the chimeric anti-GD2 monoclonal antibody ch14.18 in patients with malignant melanoma.* Hum Antibodies Hybridomas, 1992. **3**(1): p. 19-24.

59. Murray, J.L., et al., *Phase I trial of murine monoclonal antibody 14G2a administered by prolonged intravenous infusion in patients with neuroectodermal tumors.* J Clin Oncol, 1994. **12**(1): p. 184-93.

60. Richman, S.A., et al., *High-Affinity GD2-Specific CAR T Cells Induce Fatal Encephalitis in a Preclinical Neuroblastoma Model.* Cancer Immunol Res, 2018. **6**(1): p. 36-46.

61. Majzner, R.G., et al., *Neurotoxicity Associated with a High-Affinity GD2 CAR-Letter.* Cancer Immunol Res, 2018. **6**(4): p. 494-495.

62. Lynn, R.C., et al., *c-Jun overexpression in CAR T cells induces exhaustion resistance.* Nature, 2019. **576**(7786): p. 293-300.

63. Hoseini, S.S., et al., *Bispecific antibody does not induce T-cell death mediated by chimeric antigen receptor against disialoganglioside GD2.* Oncoimmunology, 2017. **6**(6): p. e1320625.

64. Richards, R.M., E. Sotillo, and R.G. Majzner, *CAR T Cell Therapy for Neuroblastoma.* Frontiers in Immunology, 2018. **9**(2380).

65. Traube, C., et al., *Cornell Assessment of Pediatric Delirium: a valid, rapid, observational tool for screening delirium in the PICU*.* Crit Care Med, 2014. **42**(3): p. 656-63.

66. Silver, G., et al., *Delirium screening anchored in child development: The Cornell Assessment for Pediatric Delirium.* Palliat Support Care, 2015. **13**(4): p. 1005-11.

Phase 1 Clinical Trial of Autologous GD2 Chimeric Antigen Receptor (CAR) T cells (GD2CART) for Diffuse Intrinsic Pontine Gliomas (DIPG) and Spinal Diffuse Midline Glioma (DMG)

DEPARTMENT OF PEDIATRICS- HEMATOLOGY AND ONCOLOGY,

and DEPARTMENT OF NEUROLOGY - NEUROONCOLOGY

STANFORD CANCER INSTITUTE

Principal Investigator:	**Michelle Monje, M.D., Ph.D.**
Lead Associate Investigator:	**Robbie Majzner, M.D.**
IND-holder:	**Crystal L Mackall, MD**

Study Agent:	**Autologous T-Cells transduced with retroviral vector (14g2a-CD8.BB.z.iCasp9) expressing GD2 chimeric antigen receptor ; and chemotherapy**
Protocol Version:	**29 December 2020**

Confidentiality Statement

This document contains confidential information of Stanford University School of Medicine. Do not copy or distribute without written permission of the sponsor.

Document History	Notes
Amd 0 Version Date: 23 March 2020	Initial IRB and FDA submission
Amd 0 Version Date: 29 April 2020	Amendment based on FDA reviewer comments. Significant changes include:<br>• Update IND number and name of investigational agent<br>• Add Section 4.2.5 'Unacceptable Toxicity'<br>• Add to DLT definition any new Grade 3 neurotoxicity lasting longer than 28 days [Section 5.4.5]<br>• Change expedited safety reporting to FDA [Section 7.5.1] to include any new Grade 3 neurotoxicity lasting longer than 96 hours.<br>• Correct typo in Table 3, Cohort Level -1<br>• Edit/formatting changes to eligibility criteria for clarity. Update correlative sample collection calendar.
Amd 1 Version Date: 06 July 2020	Significant changes include:<br>• Removed investigator: Meena Kadapakkam, M.D.<br>• Amendment to allow parallel dose escalation of subjects with spinal DMG, with the addition of a spinal cord assessment tool and treatment algorithm for potential spinal cord toxicity.<br>• Research sample collection via lumbar puncture has been added.<br>• Updated correlative sample collection.
Amd 2 Version Date: 03 December 2020	Significant changes include:<br>• Makes the ICP measurement, LPs and MRI schedules more flexible based on patient need and balancing risk/benefit for patient.<br>• Option to administer 2nd dose of GD2DART via intraventricular route with higher dose of LD chemotherapy.<br>• Provides a systematic quantitative measure of clinical benefit.<br>• Refines performance status for wheelchair bound patients; removes supratentorial spread as an exclusion criterion.<br>• Allows for follow up evaluations with local physicians given geographical locations of patients with these diseases and pandemic restrictions<br>• Modifies CRS supportive care to better meet the needs of this patient population.<br>• Minor modification to correlative sampling for sending samples from Local Health Care Provider.
Amd 2-1 Version Date 29 December 2020	Change ICV dose to 30 x10^6 GD2CART cells, with option for no lymphodepleting chemotherapy.

PRINCIPAL INVESTIGATOR'S SIGNATURE PAGE

Michelle Monje, MD, PhD

Title: Associate Professor of Neurology and Neurological Sciences

265 Campus Drive, G3077, MC5461
Stanford, CA 94305-5461
T: 650-721-5750
F: 650-724-5824
mmonje@stanford.edu

Signature _______________________ **Date** _______________

Protocol: GD2CART in DIPG and Spinal DMG

Agent: GD2.BB.z.iCasp9-chimeric antigen receptor (GD2 CAR) retroviral transduced autologous peripheral blood lymphocytes; following fludarabine and cyclophosphamide

SPONSOR DETAILS

**Name of IND Sponsor:**	Crystal L. Mackall, M.D.
Address:	265 Campus Dr. G3141A, MC5456<br>Stanford, California, 94305
Telephone Number:	1-650-725-9670

STANFORD CO-INVESTIGATORS

Liora Schultz, M.D.<br>Clinical Assistant Professor of Pediatrics<br>725 Welch Road<br>Palo Alto, CA 94304<br>T: 650-497-8953<br>F: 650-736-8092<br>lioras@stanford.edu	Kara Davis, D.O.<br>Assistant Professor of Pediatrics<br>265 Campus Dr, G2078<br>Stanford, California, 94305<br>T: 650-724-8073<br>F: 650-736-8092<br>kardavis@stanford.edu
Robbie Majzner, M.D.<br>Instructor of Pediatrics<br>1000 Welch Rd, Suite 300 Palo Alto, CA 94304<br>T: 650-723-5535<br>F: 650-723-5231<br>rmajzner@stanford.edu	Crystal Mackall, M.D.<br>Professor of Pediatrics and Medicine<br>265 Campus Dr, G3141A<br>Stanford, California, 94305<br>T: 650-725-9670<br>F: 650-736-8092<br>cmackall@stanford.edu
Sneha Ramakrishna MD<br>Instructor of Pediatrics<br>1000 Welch Rd.,<br>Suite 300 Palo Alto, CA 94304<br>T: 650-497-8953<br>ramakrs@stanford.edu	Gerald Grant, M.D., FACS<br>Endowed Professor in Pediatric Neurosurgery and Professor of Neurology<br>300 Pasteur Drive, Room R211, MC 5327<br>Stanford, CA 94304<br>T: 650-497-8775<br>Ggrant2@Stanford.edu
Sonia Partap M.D.<br>Clinical Associate Professor, Neurology & Neurological Sciences<br>750 Welch Road, Suite 317<br>Palo Alto, CA 94304<br>T: 650-723-0993<br>spartap@Stanford.edu	Cynthia Campen, M.D.<br>Clinical Associate Professor, Neurology & Neurological Sciences<br>750 Welch Road, Suite 317<br>Palo Alto, CA 94304<br>T: 650-723-0993<br>ccampen@Stanford.edu
Paul Fisher, M.D.<br>Professor of Pediatric Neuro-Oncology, Professor of Pediatrics<br>750 Welch Road, Suite 317<br>Palo Alto, CA 94304<br>T. 650-736-0885<br>pfisher@Stanford.edu	Lindsey Rasmussen, M.D.<br>Clinical Assistant Professor, Pediatrics-Critical Care<br>770 Welch Rd, Ste 435, MC 5876<br>Palo Alto, CA 94304<br>T:920-915-1013<br>lkrasmus@stanford.edu
Timothy Cornell M.D.<br>Professor of Pediatric Critical Care Medicine<br>770 Welch Rd, Ste 435, MC 5876<br>Palo Alto, CA 94304<br>T: 734-904-5467<br>tcornell@stanford.edu	Susan Hinicker M.D.<br>Assistant Professor of Radiation Oncology<br>300 Pasteur Dr. Rm A099, MC 5302<br>Stanford, CA 94305<br>T. 650-725-2209<br>shiniker@stanford.edu

Agent: GD2.BB.z.iCasp9-chimeric antigen receptor (GD2 CAR) retroviral transduced autologous peripheral blood lymphocytes; following fludarabine and cyclophosphamide

Kristen Yeom, M.D.<br>Associate Professor of Radiology<br>725 Welch Rd, MC 5654<br>Palo Alto, CA 94304<br>T. 721-2388<br>kyeom@Stanford.edu	**STUDY COORDINATORs**<br>Anne Cunniffe Marcy, MSBH, CCRP<br>800 Welch Road, FC329<br>Palo Alto, CA 94304<br>T: 650-721-9994<br>acmarcy@stanford.edu<br><br>Christina Baggott, RN, PhD, PPCNP-BC, CPON<br>800 Welch Road, FC319<br>Palo Alto, CA 94304<br>T: 650-497-7659<br>baggott@stanford.edu
	**BIOSTATISTICIAN**<br>John Tamaresis<br>Room T101F, Redwood Bldg M/C 5405<br>150 Governor's Lane<br>Stanford, CA 94305<br>T: 650-498-6425<br>jtamares@stanford.edu
**Coordinating Center/Participating Sites:**	Single Site

Agent: GD2.BB.z.iCasp9-chimeric antigen receptor (GD2 CAR) retroviral transduced autologous peripheral blood lymphocytes; following fludarabine and cyclophosphamide

SYNOPSIS

**Protocol Number**	IND# 19801/ e-Protocol # IRB-52934 / Oncore: PEDSCCT6005
**Protocol Title**	Phase 1 Clinical Trial of Autologous GD2 Chimeric Antigen Receptor (CAR) T cells (GD2CART) for Diffuse Intrinsic Pontine Gliomas (DIPG) and Spinal Diffuse Midline Glioma (DMG)
**IND Sponsor**	Crystal L. Mackall, M.D.
**Principal Investigator**	Michelle Monje, M.D., Ph.D.
**Name of Investigational Product**	**GD2CART**: Autologous T-Cells transduced with retroviral vector (14g2a-CD8. BB.z.iCasp9) expressing GD2 chimeric antigen receptor; and chemotherapy
**Clinical Phase**	Phase 1
**Background and Rationale**	**<u>Diffuse Midline Glioma (DMG) and Diffuse Intrinsic Pontine Gliomas (DIPG)</u>**<br><br>Diffuse Midline Gliomas (DMG) harboring the H3 K27M mutation, including diffuse intrinsic pontine glioma (DIPG) are lethal, high-grade pediatric brain tumors that are inoperable and pose significant challenges for treatment. Pediatric DMGs were categorized separately in the 2016 WHO classification of CNS tumors from adult gliomas based on the distinct underlying genetic abnormalities[1]. Pediatric DMG is characterized by K27 M mutations in the histone H3 gene *H3F3A,* or less commonly in the related *HIST1H3B* gene, a diffuse growth pattern, and a midline location (e.g. thalamus, brain stem and spinal cord) [2], [3]. The majority of pediatric DMGs arise in the brainstem (> 90% DIPG)[4].<br><br>Diffuse Intrinsic Pontine Glioma (DIPG) is a devastating, aggressive brain tumor arising in the ventral pons and occurring chiefly during childhood. Though brainstem tumors are infrequent among adults, diffuse gliomas of the brainstem comprise approximately 10-15% of pediatric brain tumors, with half of all pediatric malignant gliomas occurring in the brainstem[5]. With an estimated 200-400 children affected by DIPG annually in the United States, it is the second most common malignant brain tumor of childhood[6]. The prognosis is bleak: in the absence of effective therapies, DIPG is uniformly fatal and is the leading cause of childhood brain tumor-related death. Median age at diagnosis is 6.3 years, with median overall survival of 11.2 months[7]; 90% of children will die from the disease within 2 years of initial diagnosis, with less than 1% surviving after 5 years[8]. Many clinical trials over the past three decades have explored the use of various therapeutic agents for DIPG, employing conventional and high-dose chemotherapies as well as targeted agents. Chemotherapy has been attempted at time points before, during and after radiation therapy. Despite all efforts, no improvement in overall survival has been demonstrated to date[9],[10],[11],[12],[13],[14],[15],[16].<br><br>**<u>CAR Therapies</u>**<br><br>The field of cancer immunotherapy has exploded in recent years in part due to successes created by the application of chimeric antigen receptor (CAR) T cell therapy in B cell malignancies. CARs are non-native receptors that link an antigen-binding domain to cell signaling domain(s). When expressed in T cells, CARs endow MHC-unrestricted antigen specificity. Dramatic clinical responses in acute lymphoblastic leukemia (ALL) and lymphoma have led to U.S. Food and Drug Administration (FDA) approval of Kymriah for ALL and lymphoma [17] and YESCARTA™ for lymphoma[18]. A growing number of clinical trials have recently focused on solid tumors, targeting a variety of surface antigens,

including EGFR806, EGFRt, and EGFRvIII, carcinoembryonic antigen (CEA), human epidermal growth factor receptor 2 (HER2), fibroblast activation protein (FAP), and the disialoganglioside GD2.

GD2 has already been credentialed as an immune target for neuroblastoma. Dinutuximab, an anti-GD2 monoclonal antibody (mAb), improves overall survival among high-risk neuroblastoma patients when administered as part of a multimodal regimen for upfront disease[19]. More recently, the combination of dinutuximab with irinotecan/temozolomide was demonstrated to mediate objective responses in 53% of patients with recurrent neuroblastoma, compared to a 6% response rate in patients receiving irinotecan/temozolomide plus a targeted, non-immune agent (temsirolimus)[20]. Our group recently discovered that GD2 is also highly overexpressed in DIPG, at levels on early passage DIPG cell lines that exceed expression in neuroblastoma[21].

CAR Therapies Targeting GD2+ Pediatric Tumors

For cancers with high mutational burdens, such as melanoma, non-small cell lung cancer and colorectal cancers with microsatellite instability, blockade of PD-1, a T cell inhibitory signal, is often sufficient to unleash naturally acquired antitumor immunity[22];[23];[22]. Current concepts hold that mutated proteins create neoantigens that drive antitumor immune responses after PD-1 blockade, since cancers with low mutational burdens do not typically respond to checkpoint blockade [24-26]. Diffuse intrinsic pontine glioma demonstrates a low mutation burden[27], [28], low PDL1 expression and low numbers of tumor-infiltrating lymphocytes and therefore immunotherapy focused on checkpoint blockade is unlikely to demonstrate significant benefit. CAR T cells, in contrast, can effectively clear tumors with low mutational burden. Furthermore, unlike monoclonal antibodies, CAR T cells readily traffic across the blood:brain barrier. For these reasons, our efforts to develop immunotherapies for DIPG have focused on developing CAR T cells.

To identify potential targets for CAR T-cell immunotherapy in DIPG, we screened cell surface antigens using an antibody array in patient-derived DIPG cultures. Significant overlap between independent patient-derived cultures suggests conservation of a core group of surface markers across DIPG patients. From these common targets, we observed that the disialoganglioside GD2 was expressed at high levels on nearly all cells in each of the patient-derived DIPG cultures screened[21]. Unlike monoclonal antibodies, which do not efficiently cross the blood-brain barrier, activated T-cells can infiltrate the CNS following adoptive transfer[29]. We generated human GD2-targeting CAR T-cells incorporating a 4-1BBz costimulatory domain (GD2-CAR)[30] and observed significant GD2-dependent killing and cytokine generation upon exposure to patient-derived DIPG cultures relative to control CD19-CAR T-cells incorporating 4-1BBz (CD19-CAR)[21].

To evaluate *in vivo* efficacy of GD2CART against DIPG, we prepared orthotopic mouse xenografts of DIPG cultures derived from post-mortem patient tissue. DIPG cultures were transduced with a luciferase-expressing construct to enable longitudinal monitoring of tumor burden. These xenograft models faithfully recapitulate the diffusely infiltrating histology of DIPG[31], [32]. Mice were distributed by tumor burden into equivalent treatment and control groups before receiving 1e7 GD2-CAR or CD19-CAR T-cells by a single intravenous injection 7-8 weeks after establishment of pontine xenografts. Within 40 days post-treatment (DPT), marked reductions in tumor burden were observed across two independent GD2-CAR T-cell treated cohorts of mice bearing SU-DIPG6 xenografts[21]. All GD2-CAR treated animals demonstrated complete tumor clearance by bioluminescence imaging. By contrast, no mice in the CD19-CAR T-cell control groups exhibited significant tumor regression[21]. This striking tumor clearance replicated in a second patient-derived model of H3K27M+ DIPG (SU-DIPG-13FL) and in a third, particularly aggressive patient-derived H3K27M+ DIPG model (SU-DIPG-13P*). A dramatic survival advantage was also observed in these xenografted mice[21].

	Given these data we propose a single institution, Phase I dose escalation trial of GD2.BB.z-iCasp9-CAR T cells (GD2CART) following cyclophosphamide/fludarabine lymphodepleting therapy in children with H3K27M+ DIPG and spinal H3 K27M DMG following completion of standard, up front radiotherapy. We propose to conduct the safety assessment in subjects with DIPG of any age, rather than initially targeting adolescents or adults, because pontine disease is rare in adolescents and adults and evaluation of safety in spinal DMGs, which occur more commonly in adolescents and young adults, will not inform safety for pontine DMG, since much of the risk of toxicity relates to the location of the tumor. Furthermore, enrollment of adolescents or adults prior to children is not necessary since first, second and third generation CARs targeting GD2 and incorporating the same scFv have already been tested in several clinical trials and have demonstrated safety and significant clinical activity[33],[34, 35].<br><br>Patients are eligible for enrollment when they are at least 6 weeks from completion of standard upfront radiotherapy, regardless of documented evidence of progression, and if all other eligibility criteria are met. Given that manufacturing and completion of release testing takes approximately 14 days, patients will receive the treatment approximately 2-3 months following completion of standard upfront radiotherapy. This eligibility criterion was chosen based of the following considerations: 1) A requirement for documented progression prior to enrollment is not feasible, since radiographic progression often cannot be reliably distinguished from radionecrosis in patients. 2) Post-progression survival is very short (median 2.3 months) and may not be long enough for patients to benefit from the effects of the GD2CART. 3) Median progression-free survival following radiotherapy is 7.0 months (80.8% demonstrating progression within 12 months), therefore the risk of progression beyond 3 months is sufficiently high to justify the risks and morbidity associated with the investigational treatment regimen 4) Preclinical models demonstrate that bulky disease is a risk factor for treatment-related morbidity and mortality due to hydrocephalus[21], therefore enrolling patients prior to documented clinical or radiographic progression will increase the likelihood that the therapy can be rendered safely. The study will evaluate safety of administration, feasibility of manufacturing, identify the recommended phase 2 dose (RP2D) and conduct a preliminary assessment of anti-tumor activity. The CAR vector will incorporate an inducible Caspase 9 that can lead to efficient T cell apoptosis following exposure to AP1903 should toxicity require inactivation of the cell product.
**Objectives**	Primary Objectives:<br>✓ Determine the feasibility of manufacturing autologous T cells transduced with 14g2a-CD8-BBz-iCasp9 retroviral vector expressing GD2 Chimeric Antigen Receptor (GD2CART) for intravenous administration in children and young adults with H3K27M DIPG and spinal H3 K27M DMG using a retroviral vector and dasatinib in the Miltenyi CliniMACS Prodigy® system.<br>✓ Assess the safety and identify the MTD and/or recommended phase 2 dose (RP2D) of GD2CART in subjects with H3K27M DIPG and H3K27M spinal DMG administered after cyclophosphamide/fludarabine-based lymphodepletion regimen using the following dose escalation schedule: DL1: 1e6 transduced T cells/kg; DL2: 3e6 transduced T cells/kg; DL3: 10e6 transduced T cells/kg.<br>✓ Assess the safety of the MTD/RP2D of GD2CART in children and young adults with H3K27M DIPG and spinal H3K27M DMG.<br>Secondary Objectives<br>✓ In a preliminary manner, assess clinical benefit of GD2CART at the RP2D in children and young adults with H3K27M DIPG and in children and young adults with spinal H3 K27M DMG.<br>✓ If unacceptable toxicity (as defined in Section 12.5), occurs that is possibly, probably or likely related to GD2CART, assess the capacity for AP1903, a dimerizing agent, to

	mediate clearance of the genetically engineered cells and resolve toxicity.<br>Exploratory Analyses:<br>✓ Measure expansion/persistence of adoptively transferred GD2CART in the CSF and blood and correlate this with antitumor effects.<br>✓ Conduct analyses of the manufactured T cell product and blood and CSF post-infusion to identify biomarkers associated with enhanced CAR T cell expansion and/or persistence.<br>✓ Assess whether changes in the level of ctDNA in the cerebrospinal fluid can provide prognostic information and/or information regarding clonal evolution of DIPG or DMG over time.<br>✓ Evaluate whether antigen expression or tumor microenvironment are correlated with response to CAR T cell infusion.
**Primary Endpoint(s)**	The primary endpoints for this study are:<br>- Feasibility defined by the rate of successful manufacture of the GD2CART produced with retroviral vector and dasatinib in the Miltenyi CliniMACS Prodigy® system to satisfy the targeted dose level and meet the required release specifications.<br>- Identify the MTD and/or RP2D in subjects with H3K27M DIPG and in subjects with spinal H3 K27M DMG as evidenced by the number of DLTs in 3 dose cohorts (DL1: 1e6 transduced T cells/kg; DL2: 3e6 transduced T cells/kg; DL3: 10e6 transduced T cells/kg). MTD is defined as the dose below that in which 2/6 subjects experienced DLTs.<br>-Assess safety of GD2CART as evidenced by the incidence and severity of dose limiting toxicities (DLT), adverse events, serious adverse events, laboratory abnormalities, changes in vital signs, and changes in physical examination following infusion of GD2CART graded according to the Common Terminology Criteria for Adverse Events (CTCAE) Version 5.0 in children and young adults with H3K27M DIPG and children and young adults with spinal DMG.
**Secondary and Exploratory Endpoints**	1. Measure clinical outcomes following administration of a lymphodepleting preparative regimen and GD2CART in two groups of subjects: H3K27M DIPG and spinal H3K27M DMG, to include:<br>a. Overall Survival (OS), also including Post Progression Survival (PPS), Progression Free Survival (PFS), Time to Progression (TTP), and<br>b. Radiographic response to include complete response (CR), partical response (PR), stable disease (SD), and progressive disease (PD), and<br>c. Clinical evaluation of neurologic status compared to baseline.<br>2. In the event of unacceptable toxicity possibly, probably or definitely related to GD2CART, assess the efficacy of AP1903 in eliminating the genetically engineered cells and in resolving the toxicity.
**Study Centers**	This is a single site, Stanford University, investigator initiated protocol conducted at Lucile Packard Children's Hospital (LPCH) Stanford.
**Sample Size**	This study has a 3 part analysis to establish sample size:<br>1. Feasibility of manufacture;<br>2. Safety and MTD/RP2D in subjects with H3K27M+ DIPG and in subjects with spinal H3K27M DMG after administration of GD2CART following a lymphodepletion chemotherapy regimen ; and<br>3. Preliminary assessment of efficacy of GD2CART in children and young adults with H3K27M DIPG or with spinal H3K27M DMG following standard radiotherapy.

	If feasibility of cell production is not met (i.e. 3 of the first 6 subjects' cells at each dose level cannot be produced to meet the established release criteria) further enrollment will be paused pending evaluation of the manufacturing process, and modifications made appropriate to improving feasibility prior to continuing enrollment or a decision will be made to establish the RP2D as the dose level wherein manufacturing was feasible. The study will allow for up to 6 subjects to be replaced due to inability to achieve the target cell product.<br><br>Safety will include a determination of MTD/RP2D in treated subjects with DIPG and subjects with spinal DMG using a standard 3 + 3 dose escalation design at 3 dose levels (DL1: 1e6 transduced T cells/kg; DL2: 3e6 transduced T cells/kg; DL3: 10e6 transduced T cells/kg). A determination of MTD/RP2D will also be evaluated in children and young adults with spinal H3 K27M DMG.Given the risk assessment of the two patient populations, dose escalation safety of the DIPG cohort will inform the safety of dosing of subjects with spinal DMG in an effort to reduce unnecessary exposure to subtherapeutic doses while preserving safety with a rapid escalation to therapeutic doses in this vulnerable population. A minimum of 4 and a maximum of 18 evaluable subjects may be needed to determine RP2D in each group.<br><br>Once RP2D is established, up to a total of 20 evaluable subjects with DIPG (including any evaluable subjects from the dose escalation phase) and 10 evaluable subjects with spinal H3K27M DMG will be treated at the RP2D dose to further assess safety and perform a preliminary analysis of clinical activity. In addition, we will allow for 6 inevaluable subjects (subjects enrolled but who cannot receive cells, either due to physical deterioration or withdrawn consent during cell growth).<br><br>Thus, a maximum of 66 (36 in 2 dose escalation cohorts + 14 in expansion cohort of DIPG and 4 in expansion cohort of spinal DMG + 6 replaced for manufacturing feasibility + 6 replaced for inevaluability following enrollment) subjects may be enrolled to determine safety, feasibility, and preliminary efficacy.
**Overall Duration of the Study**	During the dose escalation phase accrual rate will be limited by observational periods dictated by the study, and thus a maximum of 1-2 subjects accrued/month is expected. During the expansion phase, at least 2 subjects will be accrued per month. Therefore, this study may require up to 3 years to complete accrual. The study primary and secondary objectives will be completed in approximately 4 years.
**Duration of Study per Subject**	Subject's active participation in this study is expected to be 5 years, with a total of 15 years of long term follow up from the time of cell infusion on this study or on a long term follow up study, according to the U.S. Food and Drug Administration (FDA) Guidance for Industry: Gene Therapy Clinical Trials – Observing Participants for Delayed Adverse Events.
**Subject Population**	Subjects ≥ 2 year of age and ≤ 30 years of age, with biopsy documented H3K27M DIPG or spinal H3K27M DMG enrolled following standard upfront radiation therapy, who meet the eligibility criteria.
**Eligibility criteria**	**Inclusion Criteria**<br>✓ Disease Status<br>o <u>Dose escalation phase and DIPG expansion cohort</u>: Tissue diagnosis of H3K27M mutant Diffuse Intrinsic Pontine Glioma (DIPG) with radiographically evident tumor restricted to the brainstem, OR<br>o <u>Dose escalation phase and Spinal DMG expansion cohort</u>: Tissue diagnosis of H3K27M mutant Diffuse Midline Glioma (DMG) of the spinal cord

- ✓ Age: Greater than or equal to 2 year of age and less than or equal to 30 years of age
- ✓ Prior Therapy:
  - o At least 6 weeks following completion of front line radiation therapy.
  - o At least 3 weeks post chemotherapy or 5 half-lives, whichever is shorter must have elapsed since any prior systemic therapy, except for systemic inhibitory/simulatory immune checkpoint therapy, which requires 5 half-lives..
- ✓ Performance Status: Subjects > 16 years of age: Karnofsky ≥ 60% OR Eastern Cooperative Oncology Group (ECOG) performance status of 0 or 1; Subjects ≤ 16 years of age: Lansky scale ≥ 60% (See section **13.1**, Appendix A). Subjects who are unable to walk because of paralysis, but who are up in a wheelchair, will be considered ambulatory for the purpose of assessing the performance score.
- ✓ Normal Organ and Marrow Function (supportive care is allowed per institutional standards, i.e. filgrastim, transfusion)
  - a) ANC ≥ 1,000/uL
  - b) Platelet count ≥ 100,000/uL
  - c) Absolute lymphocyte count ≥ 150/uL
  - d) Hemoglobin ≥ 8 g/dL
  - e) Adequate renal, hepatic, pulmonary and cardiac function defined as:
    - o Creatinine within institutional norms for age (i.e. ≤ 2 mg/dL in adults or according to table below in children <18 years) OR creatinine clearance (as estimated by Cockcroft Gault Equation) ≥ 60 mL/min

Age<br>(Years)	Maximum Serum<br>Creatinine (mg/dL)
≤5	0.8
5 < age ≤ 10	1.0
>10-18	1.2
>18	2.0

    - o Serum ALT/AST ≤ 3.0 ULN (grade 1)
    - o Total bilirubin ≤ 1.5 mg/dl, except in subjects with Gilbert's syndrome.
    - o Cardiac ejection fraction ≥ 45%, no evidence of physiologically significant pericardial effusion as determined by an ECHO, and no clinically significant ECG findings
    - o Baseline oxygen saturation > 92% on room air
- ✓ Pregnancy Test

  Females of childbearing potential must have a negative serum or urine pregnancy test (females who have undergone surgical sterilization are not considered to be of childbearing potential).
- ✓ Contraception

  Subjects of child-bearing or child-fathering potential must be willing to practice birth control from the time of enrollment on this study and for four (4) months after receiving the preparative lymphodepletion regimen or for as long as GD2CART are detectable in peripheral blood or CSF.
- ✓ Ability to give informed consent. Pediatric subjects will be included in age appropriate discussion and written assent will be obtained for those ≥ 7 years of age, when appropriate.

	**Exclusion Criteria**<br>✓ Tumor involvement of cerebellar vermis or hemispheres (pontocerebellar peduncle involvement is allowed), thalamic lesions.<br>✓ Clinically significant swallowing dysfunction as judged by clinical assessment.<br>✓ Current systemic corticosteroid therapy<br>✓ Prior CAR therapy.<br>✓ Uncontrolled fungal, bacterial, viral, or other infection. Previously diagnosed infection for which the patient continues to receive antimicrobial therapy is permitted if responding to treatment and clinically stable.<br>✓ Ongoing infection with HIV or hepatitis B (HBsAg positive) or hepatitis C virus (anti-HCV positive). A history of hepatitis B or hepatitis C is permitted if the viral load is undetectable per quantitative PCR and/or nucleic acid testing.<br>✓ Clinically significant systemic illness or medical condition (e.g. significant cardiac, pulmonary, hepatic or other organ dysfunction), that in the judgement of the principal investigator is likely to interfere with assessment of safety or efficacy of the investigational regimen and its requirements.<br>✓ In the investigator's judgment, the subject is unlikely to complete all protocol-required study visits or procedures, including follow-up visits, or comply with the study requirements for participation.<br>✓ Known sensitivity or allergy to any agents/reagents used in this study.<br>✓ Primary immunodeficiency or history of autoimmune disease (e.g. Crohns, rheumatoid arthritis, systemic lupus) requiring systemic immunosuppression/systemic disease modifying agents within the last 2 years
**Investigational Product, Dose, and Mode of Administration**	Autologous peripheral blood mononuclear cells (PBMC) will be obtained by leukapheresis and cryopreserved. Cryopreserved PBMC stored from participation in other institutional cell therapy or cell collection studies may be used to generate the cellular product on this study as long as they meet the criteria established in this IND. PBMC will be transported to the manufacturing facility where they will undergo selection, activation, transduction with the retroviral vector, expansion, supplemented with dasatinib and formulated for the manufacture of GD2CART. The product will be cryopreserved and returned to Stanford's Cell Therapy Facility (CTF), from which the product will be distributed to the patient care unit for infusion. A conditioning lymphodepletion chemotherapy regimen with cyclophosphamide and fludarabine will be administered prior to cell infusion.<br>Lymphodepletion prior to GD2CART infusion will occur as follows:<br>• Fludarabine 25 mg/m^2 per day IV for days -4, -3, -2<br>• Cyclophosphamide 500 mg/m^2 per day IV for days -4, -3, -2<br>Autologous GD2CART will be administered intravenously in 3 escalating doses (starting at Dose level 1) on Day 0 in hospitalized subjects with either DIPG or spinal DMG.<br>▪ Dose Level -1: 3e5 transduced T cells/kg (± 20%)<br>▪ Dose Level 1: 1e6 transduced T cells/kg (± 20%)<br>▪ Dose Level 2: 3e6 transduced T cells/kg (± 20%)<br>▪ Dose Level 3: 10e6 transduced T cells/kg (± 20%)<br>Subjects who in the investigator's opinion would benefit or subjects who had stable disease with clinical benefit or clinical response (CR or PR) to the initial infusion and who later develop recurrence or progression of disease may be offered an additional dose of GD2CART

	administered intravenously at the same dose or intraventricularly at a set dose as described in **Section 5.6.2**.
**Study Design and Methodology**	The dose escalation phase utilizes a 3 + 3 study design and will test safety in subjects with H3K27M DIPG and spinal H3K27M DMG enrolled at least 6 weeks following standard first line radiation therapy. With substantial data supporting safety of GD2 CAR T cells in children and adults and the age of the vast majority of subjects with DIPG, enrollment in this dose escalation portion will allow subjects of any age.<br><br>Three planned dose levels of GD2CART will be administered in escalating cohorts starting at dose level 1: 1e6 transduced T cells/kg (± 20%). Infusions will be staggered between subjects to evaluate safety; 28 days must elapse after infusion of GD2CART in the first subject in each dose cohort; 14 days must elapse after cell infusion in the second and subsequent subjects in each dose cohort; and 28 days must elapse after the last subject in each dose cohort to fully access DLTs. If 2/6 subjects experience DLT at dose level 1, dose -1 will be explored (3e5 transduced T cells/kg (± 20%)). MTD will be defined as the dose level in which <30% (2 out of 6) subjects experience DLTs. The MTD, or highest cell dose studied if MTD is not reached, will be considered the RP2D. To avoid unnecessary exposure of subjects to subtherapeutic doses while preserving the safety during rapid dose escalation, the safety data from subjects being treated with H3K27M DIPG will inform the safety of that dose level in subjects with spinal H3K27M DMG. For example, if three subjects with DIPG complete dose level 1 without DLT, subsequent subjects with spinal DMG may enroll to receive dose level 2. The safety data of a dose in subjects with spinal DMG though will NOT inform the cohort of subjects with DIPG given the risks associated with tumor location of DIPG in the pons.<br><br>Once the RP2D is established in the two disease cohorts, enrollment will be expanded to a total of 20 subjects with DIPG and a total of 10 subjects with DMG to further investigate safety and to obtain preliminary data regarding efficacy. During conduct of the two expansion cohorts (DIPG and DMG), safety boundaries will be used to monitor a DLT rate of 30%; however, given the expected toxicity rate we do not anticipate crossing these boundaries. If the number of DLTs in either expansion cohort exceeds 30%, the MTD/RP2D dose level will be de-escalated to the next lower dose and that dose expansion cohort will complete enrollment with the updated RP2D dose level to further assess safety at this dose level and gather preliminary evidence for efficacy.<br><br>Subjects with DIPG will have an intraventricular catheter (Ommaya catheter) placed following enrollment and prior to T cell infusion to allow monitoring, and treatment if necessary, of increased intracranial pressure (ICP).<br><br>Subjects will receive a conditioning lymphodepletion chemotherapy regimen of fludarabine and cyclophosphamide, followed by intravenous infusion of GD2CART (Day 0). Subjects will be monitored closely as an inpatient or outpatient with close proximity to Stanford Clinic, at the investigators' discretion for at least 28 days post-T cell infusion (or longer if the principal investigator or her designee deems necessary) for safety assessments, according to the following schedule:<br><br>Neurological exam: Daily while hospitalized, or at least 5 times per week as outpatient (with no more than 48 hours between exams) until Day 14, then twice weekly as outpatient until Day 28, with increased frequency as clinically indicated.<br><br>Measurement of ICP in subjects with Ommaya Reservoir: baseline D0 (prior to infusion), and may include Day 3, Day 7, Day 10, Day 14, Day 18, Day 21, Day 24, Day 28, or as clinically indicated. Lumbar puncture (LP) pressure may be measured in consenting subjects with spinal DMG on Day 7, 10, and 14, and possibly Day 0, 21 and 28, or as clinically indicated. Should toxicities arise in either patient population that suggest increasing pressure, this scheduled may be altered to best provide clinical decisions with less frequent or

	additional ICP or LP as needed. If evidence of neurotoxicity, including ICANS≥2, increased intracranial pressure or clinical deterioration suspected due to neurologic compromise, the patient will be treated according to the neurotoxicity management algorithm (section **13.2.3.5**) which includes emergent imaging and transfer to the ICU for intensive monitoring and intervention.<br><br>Radiographic imaging in subjects with DIPG: MRI at baseline (within 28 days prior to D-4), with optional scans at D7, D14, D21 and mandatory scan at D28, or as clinically indicated. If clinical condition post-infusion prevents MRI, a CT may be obtained on those days. Sequences detailed in section **5.1.k.**<br><br>Disease evaluations of subjects with spinal H3K27M DMG will be specific to the subject's location of disease and will include MRIs with and without gadolinium.<br><br>Clinical evaluation of neurologic status will be performed at baseline, at least weekly post cell infusion to Day 27, Day 28, and with each follow up evaluation (described in **Section 5.5.5** until PD. Evaluations may be performed in person or via telemedicine conference.<br><br>Neurologists, neuro-oncologists, neurosurgeons and neurointensivists will be consulted and involved in every case and neurointensive care provided for management of increased intracranial pressure or neurologic compromise. The period of DLT assessment will be 28 days after infusion of GD2CART (Day 0). Subjects will be evaluated closely for all toxicities, antitumor effects and for persistence of CAR expressing T cells in blood and CSF, when feasible.<br><br>Retreatment may be offered to subjects as described in **Section 5.6.2.**

SCHEMA

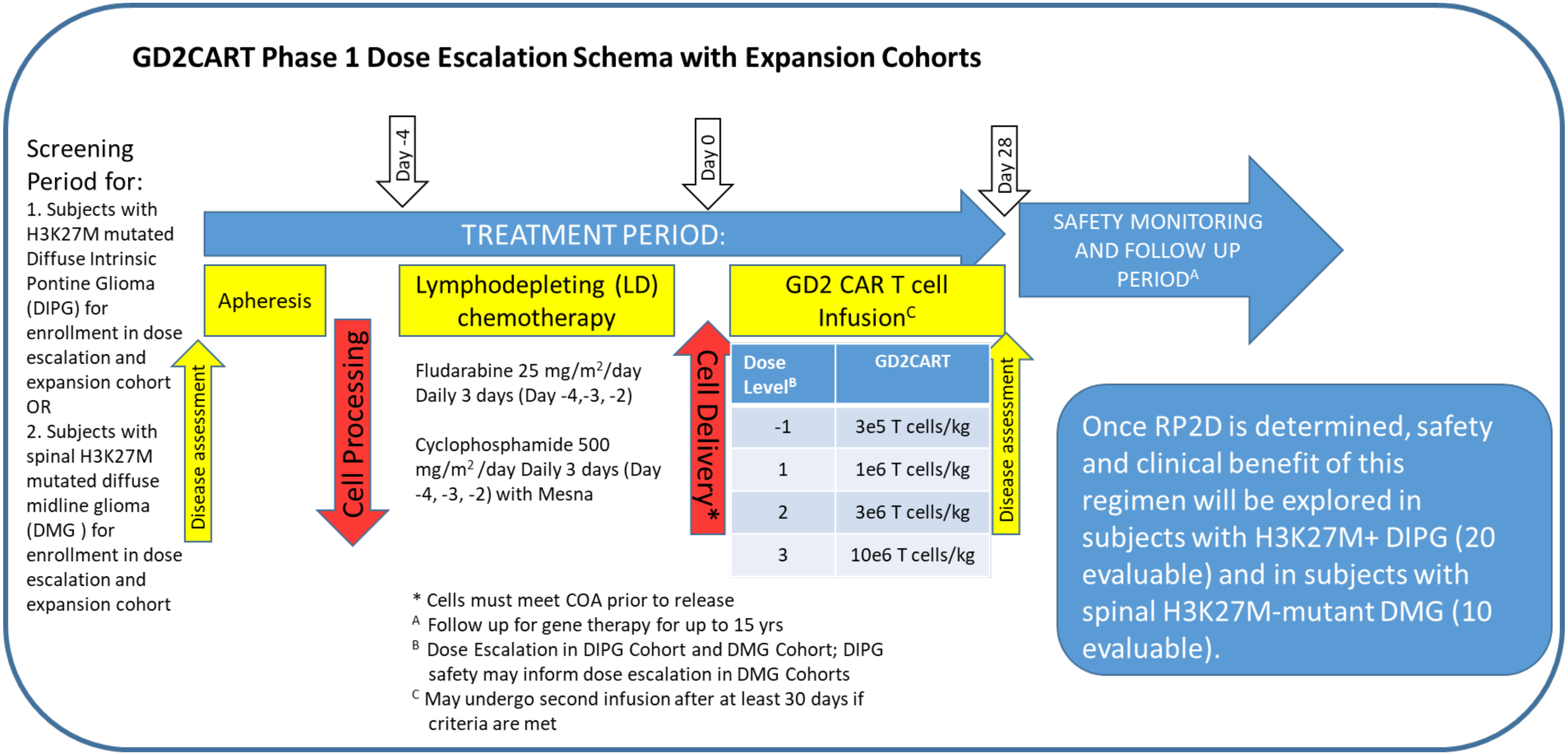
GD2CART Phase 1 Dose Escalation Schema with Expansion Cohorts
Screening Period for:
1. Subjects with H3K27M mutated Diffuse Intrinsic Pontine Glioma (DIPG) for enrollment in dose escalation and expansion cohort
OR
2. Subjects with spinal H3K27M mutated diffuse midline glioma (DMG) for enrollment in dose escalation and expansion cohort
Day -4
Day 0
Day 28
TREATMENT PERIOD:
SAFETY MONITORING AND FOLLOW UP PERIOD^A
Apheresis
Lymphodepleting (LD) chemotherapy
GD2 CAR T cell Infusion^C
Disease assessment
Cell Processing
Cell Delivery*
Disease assessment
Fludarabine 25 mg/m²/day Daily 3 days (Day -4,-3, -2)
Cyclophosphamide 500 mg/m² /day Daily 3 days (Day -4, -3, -2) with Mesna
Dose Level^B | GD2CART
-1 | 3e5 T cells/kg
1 | 1e6 T cells/kg
2 | 3e6 T cells/kg
3 | 10e6 T cells/kg
Once RP2D is determined, safety and clinical benefit of this regimen will be explored in subjects with H3K27M+ DIPG (20 evaluable) and in subjects with spinal H3K27M-mutant DMG (10 evaluable).
* Cells must meet COA prior to release
A Follow up for gene therapy for up to 15 yrs
B Dose Escalation in DIPG Cohort and DMG Cohort; DIPG safety may inform dose escalation in DMG Cohorts
C May undergo second infusion after at least 30 days if criteria are met

GD2CART Activity Schema

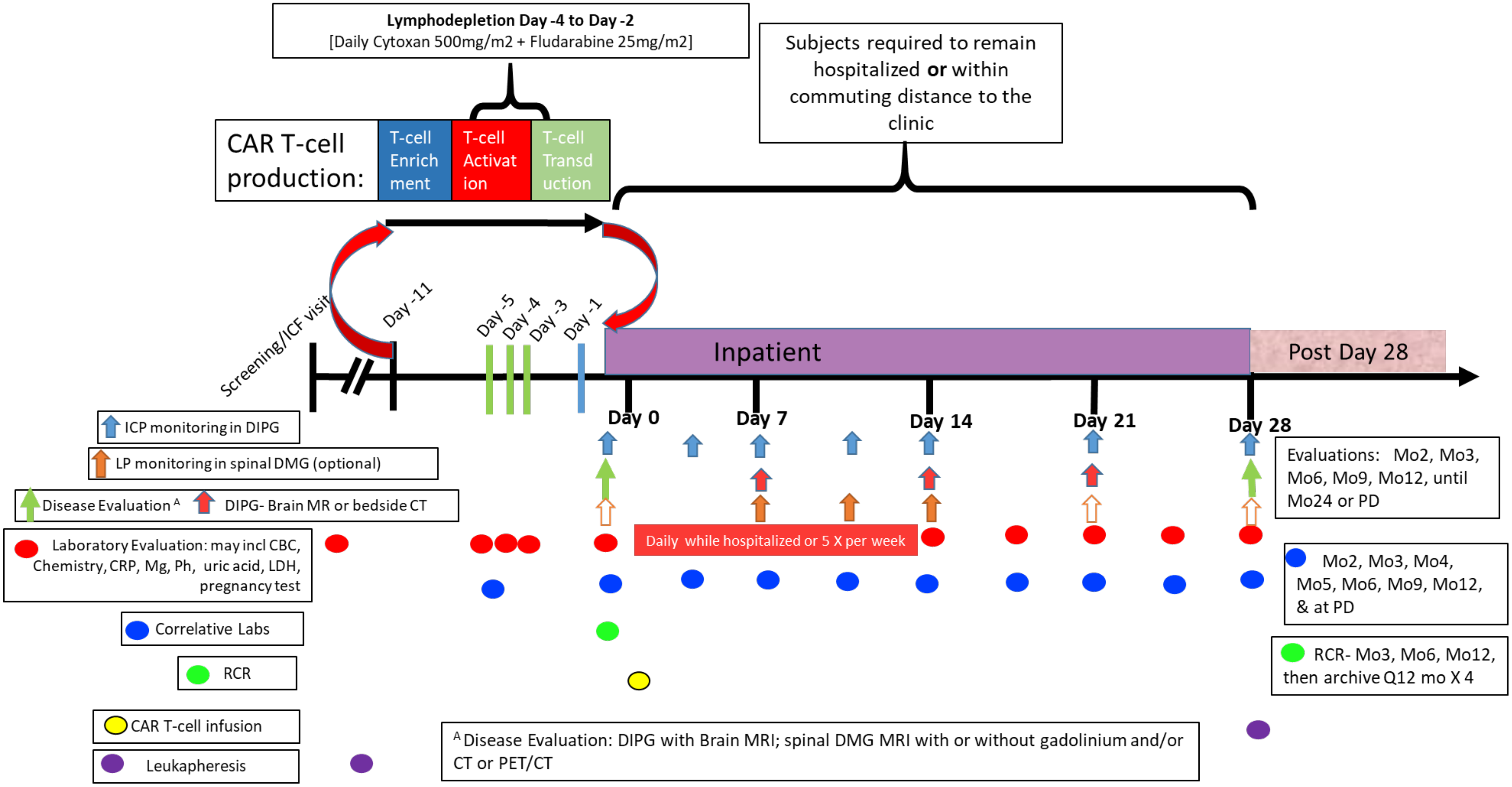

TABLE OF CONTENTS

PRINCIPAL INVESTIGATOR'S SIGNATURE PAGE 3
SPONSOR DETAILS 4
STANFORD CO-INVESTIGATORS 4
SYNOPSIS 6
SCHEMA 15
TABLE OF CONTENTS 17
TABLE OF FIGURES 20
TABLE OF TABLES 20
LIST OF ABBREVIATIONS AND DEFINITION OF TERMS 21
1 OBJECTIVES 23
1.1 PRIMARY OBJECTIVE 23
1.2 SECONDARY OBJECTIVES 23
1.3 EXPLORATORY ANALYSES: 23
2 BACKGROUND AND RATIONALE 23
2.1 DIFFUSE MIDLINE GLIOMAS (DMG) 23
2.2 DIFFUSE INTRINSIC PONTINE GLIOMAS (DIPG) 24
2.3 GD2 ANTIGEN 24
2.4 CAR THERAPIES 25
2.5 PREVIOUS HUMAN EXPERIENCE WITH GD2-CAR T CELL THERAPIES 31
2.6 SAFETY CONSIDERATIONS FOR GD2 CAR THERAPY 36
2.7 RISK FOR ON-TARGET TOXICITY AND RATIONALE FOR THE INTEGRATED SUICIDE DOMAIN (ICASP9) 40
2.8 CORRELATIVE STUDIES BACKGROUND 42
2.9 STUDY DESIGN 42
2.10 PROTOCOL RATIONALE AND SUMMARY 42
3 PARTICIPANT SELECTION AND ENROLLMENT PROCEDURES 47
3.1 SCREENING PARTICIPANT ELIGIBILITY CHECKLIST 48
3.2 INFORMED CONSENT PROCESS 52
3.3 SUBJECT SCREENING ASSESSMENTS AND REGISTRATION 52

4 TREATMENT PLAN 53

4.1 OVERVIEW 53

4.2 : GENERAL CONCOMITANT MEDICATION AND SUPPORTIVE CARE GUIDELINES 56

4.3 CRITERIA FOR REMOVAL FROM PROTOCOL THERAPY AND OFF STUDY CRITERIA 58

5 SUBJECT STUDY PROCEDURES 59

5.1 SUBJECT SCREENING 59

5.2 LEUKAPHERESIS FOR CELL ACQUISITION: 61

5.3 CONDITIONING LYMPHODEPLETION CHEMOTHERAPY REGIMEN 62

5.4 INVESTIGATIONAL AGENT ADMINISTRATION AND RESEARCH PROCEDURES 64

5.5 EVALUATIONS AND FOLLOW UP 72

5.6 OPTION FOR ADDITIONAL DOSE(S) OF GD2CART 78

6 INVESTIGATIONAL AGENT AND COMMERCIAL DRUG INFORMATION 80

6.1 GD2.BB.Z.ICASP9-CHIMERIC ANTIGEN RECEPTOR (GD2-CAR) RETROVIRAL TRANSDUCED AUTOLOGOUS PERIPHERAL BLOOD LYMPHOCYTES (GD2CART); FOLLOWING FLUDARABINE AND CYCLOPHOSPHAMIDE 80

6.2 FLUDARABINE 81

6.3 CYCLOPHOSPHAMIDE 82

6.4 ACETAMINOPHEN (TYLENOL): 83

6.5 DIPHENHYDRAMINE (BENADRYL): 83

6.6 ANTIMICROBIAL PROPHYLAXIS 84

6.7 LEVETIRACETAM (KEPPRA): 84

6.8 MESNA 84

6.9 AP1903 DIMERIZING AGENT 84

7 ADVERSE EVENTS AND REPORTING PROCEDURES 85

7.1 POTENTIAL ADVERSE EVENTS 85

7.2 ADVERSE EVENT DEFINITIONS 86

7.3 ADVERSE EVENT REPORTING 88

7.4 IND SPONSOR REPORTING CRITERIA 89

7.5 FDA REPORTING CRITERIA 91

8 CORRELATIVE/SPECIAL STUDIES 92

8.1 SAMPLE COLLECTION SCHEDULE 93

8.2 BLOOD DRAWING LIMITS FOR RESEARCH PURPOSES 94

9 STUDY CALENDAR 95

10 MEASUREMENTS 98

10.1 OUTCOME MEASURES 98

10.2 EXPLORATORY OBJECTIVES 100

10.3 INSTITUTIONAL REVIEW OF PROTOCOL 100

10.4 DATA AND SAFETY MONITORING PLAN 100

10.5 DATA MANAGEMENT PLAN 101

11 COLLABORATIVE AGREEMENTS 101

12 STATISTICAL CONSIDERATIONS 102

12.1 STATISTICAL DESIGN 102

12.2 PRIMARY OBJECTIVES: 102

12.3 SAFETY ENDPOINTS 105

12.4 EFFICACY ENDPOINT 106

12.5 CAPACITY FOR AP1903 TO MEDIATE CLEARANCE OF GENETICALLY ENGINEERED CELLS AND RESOLVE TOXICITY 107

12.6 PROTOCOL STOPPING RULES 107

12.7 EXPLORATORY ANALYSIS 107

12.8 SAMPLE SIZE 108

13 APPENDICES 110

13.1 APPENDIX A: APPENDIX A: PERFORMANCE STATUS CRITERIA 110

13.2 APPENDIX B: GUIDELINES TOXICITY ASSESSMENT AND MANAGEMENT 111

13.3 APPENDIX C: CALCULATION OF WEIGHT FOR CELL DOSE CALCULATION IN MORBIDLY OBESE CANDIDATES 120

13.4 APPENDIX D: MONITORING GENE THERAPY TRIALS: REPLICATION COMPETENT RETROVIRUS (RCR) 121

13.5 APPENDIX E: DRAFT LETTER AND QUESTIONNAIRE TO SUBJECTS FOR LONG TERM FOLLOW-UP FOR DELAYED ADVERSE EVENTS 122

13.6 APPENDIX F: PHYSICIAN (LOCAL MEDICAL PROVIDER) LETTER 124

13.7 APPENDIX G: CORRELATIVE SAMPLE SCHEDULE 125

13.8 APPENDIX H: CLINICAL EVALUATION OF NEUROLOGIC STATUS 126

13.9 APPENDIX I: INTRACEREBROVENTRICULAR CATHETER (ICV) ADMINISTRATION VIA OMMAYA RESERVOIR (ADULT/PEDS) 129

14 REFERENCES 131

TABLE OF FIGURES

Figure 1: GD2 is an immunotherapy target in DIPG 27

Figure 2: GD2-CAR T cells mediate potent and lasting antitumor response in DIPG orthotopic xenografts 29

Figure 3: GD2-CAR T cell therapy improves survival in DIPG orthotopic xenografts 30

Figure 4: GD2-CAR (OX40.28.z) 31

Figure 5: Effects of Dasatinib on GD2CART 34

Figure 6: GD2 CAR T Culture Expansion on the Prodigy 35

Figure 7: Improved GD2 CAR T Function with addition of dasatinib. 35

TABLE OF TABLES

Table 1: Conditioning Lymphodepletion Chemotherapy Regimen 63

Table 2: GD2CART Product Rapid Release Criteria 64

Table 3: GD2CART Dose Levels 67

Table 4: Dose Escalation Rules 68

LIST OF ABBREVIATIONS AND DEFINITION OF TERMS

ADL	Activities of daily living
AE	Adverse event
BID	Twice daily
BMT-CTF	Stanford Bone Marrow Transplant – Cellular Therapeutics Facility
BSA	Body surface area
CAPD	Cornell Assessment of Pediatric Delirium
CAR	Chimeric Antigen Receptor
CBC	Complete blood count
CI	Confidence interval
CLS	Capillary Leak Syndrome
CMAX	Maximum concentration of drug
CMV	Cytomegalovirus
CNS	Central nervous system
CRF	Case report/Record form
CR	Complete response
CRS	Cytokine release syndrome
CSF	cerebral spinal fluid
CTCAE	Common Terminology Criteria for Adverse Events
DIPG	Diffuse Intrinsic Pontine Glioma
DLCO	Diffusing capacity of the lungs for carbon monoxide
DLT	Dose Limiting Toxicity
DMG	Diffuse Midline Glioma
DMSO	Dimethyl Sulfoxide
DPT	Days post treatment
DSMB	Data Safety Monitoring Board
EBV	Epstein-Barr virus
ECG	Electrocardiogram
EFS	Event free survival
ELISA	enzyme-linked immunosorbent assay
GI	Gastrointestinal
GMP	Good Manufacturing Practices
GvHD	Graft versus Host Disease
HbsAG	Hepatitis B surface antigen
Hgb	Hemoglobin
HIV	Human Immunodeficiency Virus
HLH	Hemophagocytic lymphohistiocytosis
HPF	High-power field
HSV	herpes simplex virus
HSCT	Hematopoietic Stem Cell Transplant
HTN	Hypertensions
HUS	Hemolytic Uremic Syndrome
ICANS	Immune effector Cell-Associated Neurotoxicity Syndrome

ICE	Immune effector Cell-associated Encephalopathy
ICP	Intracranial pressure
ICV	intracerebroventricular
IDB	ideal body weight
IRB	Institutional Review Board
IV	Intravenous
LCGM	Stanford’s Laboratory for Cell and Gene Medicine
LLN	Lower limit of normal
MAS	macrophage activation syndrome
MRD	Minimal residual disease
MRI	Magnetic resonance imaging
MTD	Maximum tolerated dose
MUGA scan	multigated acquisition scan
NHL	Non-Hodgkin lymphoma
OR	Overall response
OS	Overall survival
PBMC	Peripheral blood mononuclear cells
PCR	polymerase chain reaction
PD	Progressive disease
PFS	Progression free survival
PFTs	pulmonary function tests
PLT	Platelet
PPS	Post progression survival
RP2D	Recommended phase 2 dose
PR	Partial response
QD	Once daily
RCR	Replication Competent Retrovirus
RECIST	Response evaluation criteria in solid tumors
RR	Response rate
R/R	Relapsed/refractory
SAE	Serious adverse event
SCT	stem cell transplant
SD	Stable disease
TCR	T-cell Receptor
TLS	Tumor lysis syndrome
TTP	Time to progression
ULN	Upper limit of normal
UNK	Unknown
VZV	varicella zoster virus
WBC	White blood cell
WHO	World Health Organization

1 OBJECTIVES

1.1 PRIMARY OBJECTIVE

1. Determine the feasibility of manufacturing autologous T cells transduced with 14g2a-CD8-BBz-iCasp9 retroviral vector expressing GD2 Chimeric Antigen Receptor (GD2CART) for administration in subjects with H3K27M+ diffuse intrinsic pontine glioma (DIPG) or subjects with spinal H3K27M diffuse midline glioma (DMG) using a retroviral vector and dasatinib in the Miltenyi CliniMACS Prodigy® system.
2. Assess the safety and identify the maximum tolerated dose (MTD) and/or recommended phase 2 dose (RP2D) of GD2CART in subjects with H3K27M+ DIPG and in subject with spinal H3K27M DMG administered after cyclophosphamide/fludarabine-based lymphodepletion regimen using the following dose escalation schedule: DL1: 1e6 transduced T cells/kg; DL2: 3e6 transduced T cells/kg; DL3: 10e6 transduced T cells/kg.
3. Assess the safety of the MTD/RP2D of GD2CART in subjects with H3K27M+ DIPG and subjects with spinal H3K27M DMG.

1.2 SECONDARY OBJECTIVES

1. In a preliminary manner, assess clinical benefit of GD2CART at the RP2D in subjects with H3K27M DIPG or spinal H3K27M DMG.
2. If unacceptable toxicity (as defined in section 12.5) occurs that is possibly, probably or likely related to GD2CART, assess the capacity for AP1903, a dimerizing agent, to mediate clearance of the genetically engineered cells and resolve toxicity.

1.3 EXPLORATORY ANALYSES:

1. Measure expansion/persistence/phenotype of adoptively transferred GD2CART in the CSF and blood and correlate this with antitumor effects.
2. Conduct analyses of the manufactured T cell product and blood and CSF post-infusion to identify biomarkers associated with enhanced CAR T cell expansion, persistence and/or phenotype.
3. Assess whether changes in the level of ctDNA in the cerebrospinal fluid can provide prognostic information and/or information regarding clonal evolution of DIPG over time.
4. Evaluate whether antigen expression or tumor microenvironment are correlated with response to CAR T cell.

2 BACKGROUND AND RATIONALE

2.1 DIFFUSE MIDLINE GLIOMAS (DMG)

The 2016 World Health Organization Classification of Tumors of the Central Nervous System combined histopathological and molecular features to standardize the diagnosis and nomenclature of CNS tumors[1]. Previously pediatric diffuse gliomas were grouped with their adult counterparts despite known differences. Understanding the distinct underlying genetic abnormalities has aided this separation of entities. One defined group of tumors primarily occurring in children, is characterized by K27M mutations, a diffuse growth pattern and a midline location, such as the

thalamus, brain stem and spinal cord[1]. Over 70% of patients with DMG harbor the H3K27M mutation, which correlates with a poorer outcome[36]. Two types of DMG with the H3K27M mutation will be the focus of this phase 1 clinical trial, spinal DMG and DIPG.

2.2 DIFFUSE INTRINSIC PONTINE GLIOMAS (DIPG)

Diffuse Intrinsic Pontine Glioma (DIPG) is a devastating, aggressive brain tumor of childhood arising in the ventral pons. Though brainstem tumors are rare among adults, they comprise approximately 10-15% of pediatric brain tumors, with half of all pediatric malignant gliomas occurring in the brainstem[5]. DIPG is the most common tumor subtype in this anatomical region, constituting 80% of brainstem gliomas[37]. With an estimated 200-400 children affected by DIPG annually in the United States, it is the second most common malignant brain tumor of childhood[6, 38]. The prognosis is bleak: in the absence of effective therapies, DIPG is uniformly fatal and is the leading cause of childhood brain tumor death. Median age at diagnosis is 6.3 years, with median overall survival of 11.2 months[7]; 90% of children will die from the disease within 2 years of initial diagnosis, with less than 1% surviving after 5 years[8].

Because DIPG grows diffusely and infiltrates critical brainstem structures, surgical resection is not possible. Radiation therapy has remained the mainstay of treatment for the past three decades. At most treatment centers, the standard recommendation is conventionally fractionated local field radiotherapy with dose range of 54-60 Gy for a period of 6 weeks[39]. Radiotherapy provides temporary improvement or stabilization of symptoms and extends overall survival by an average of 3 months; median survival is less than 5 months without radiation[40]. Though both clinical and radiographic responses are initially observed, local recurrence invariably occurs.

Many clinical trials of the past three decades have explored the use of various therapeutic agents for DIPG, employing conventional and high-dose chemotherapies as well as targeted agents. Chemotherapy has been attempted at time points before, during and after radiation therapy. Despite all efforts, no improvement in overall survival has been demonstrated to date[9],[10],[11],[12],[13],[14],[15],[16].

2.3 GD2 ANTIGEN

Tumor targets sought in the development of novel immune therapies require that the antigen is not widely expressed on normal human cells, or differential levels of surface expression on tumors versus normal tissue, thus enabling a therapeutic window. Surface disialoganglioside GD2 is one such target, and is overexpressed on the surface of a variety of cancer tumors including neuroectodermal or epithelial cells, including melanoma, neuroblastoma and osteosarcoma compared to normal tissue. Data generated in Dr. Monje's laboratory at Stanford shows very high overexpression of GD2 in virtually all samples of H3 K27M mutant gliomas[21].

GD2 has already been credentialed as an immune target for neuroblastoma. Dinutuximab, an anti-GD2 mAb improves overall survival among high risk patients as part of multimodal regimen for therapy of upfront disease[19]. More recently, the combination of dinutuximab with irinotecan/temozolomide was demonstrated to mediate objective responses in 53% of patients with recurrent neuroblastoma, compared to a 6% response rate in patients receiving irinotecan/temozolomide plus a target, non-immune agent (temsirolimus)[20]. GD2 targeted therapy has not yet been tested in DIPG. Anti-GD2 antibodies are not good candidates however, due to limited trafficking across the blood:brain barrier. In contrast, because CAR T cells

demonstrate robust trafficking across the blood:brain barrier, we have focused our efforts in developing a CAR targeting GD2 for treatment of DIPG.

2.3.1 GD2 as a target in DIPG and *in vitro* data

To identify potential targets for CAR T-cell immunotherapy in DIPG, we screened cell surface antigens using an antibody array in patient-derived DIPG cultures (**Figure 1a**). Significant overlap between independent patient-derived cultures (**Figure 1b**), suggests conservation of a core group of surface markers across DIPG patients. From these common targets, we observed that the disialoganglioside GD2 was expressed at high levels on each of twelve patient-derived DIPG cultures screened (**Figure 1a**)[21], but not on two samples of H3 wild type glioma. Hit validation by flow cytometry in six H3K27M+ DIPG cultures confirmed uniform, high GD2 expression in all H3K27M+ DIPG cultures examined, including those with the H3F3A K27M mutation (SU-DIPG-6, 13, 17, 25, 29) and the less-common HIST1H3B K27M mutation (SU-DIPG-21)[3],[2],[41] (**Figure 1c**). GD2 expression was far lower in two histone-3 WT pediatric high-grade gliomas (pHGG), including a case of H3WT DIPG (**Figure 1c**). Double immunostaining of primary human DIPG tissue for H3K27M to identify infiltrating malignant cells and GD2 confirmed local expression of GD2 in the native tumor context (**Figure 1d**). Importantly, GD2 expression on DIPG cells was found to be higher than any previous malignancy screened, including neuroblastoma for which anti-GD2 antibodies are part of the standard of care (data not shown) Double immunostaining of primary human DIPG tissue for H3K27M to identify infiltrating malignant cells and GD2 confirmed significant overexpression of GD2 in the native tumor context compared to non-malignant neural tissue (**Figure 1d**).

2.4 CAR THERAPIES

The field of cancer immunotherapy has exploded in recent years with the successes created by the application of chimeric antigen receptor (CAR) T cell therapy in cancers, primarily hematologic malignancies. CARs are non-native receptors that link an antigen-binding domain to cell signaling domain(s). When expressed in T cells, CARs endow MHC-unrestricted antigen specificity. Dramatic clinical responses observed in acute lymphoblastic leukemia (ALL) and lymphoma have led to U.S. Food and Drug Administration (FDA) approval of Kymriah™[17] for ALL and lymphoma and YESCARTA™[18] for lymphoma. A growing number of clinical trials have recently focused on solid tumors, targeting a variety of surface antigens, including EGFR806, EGFRt, and EGFRvIII, carcinoembryonic antigen (CEA), human epidermal growth factor receptor 2 (HER2), fibroblast activation protein (FAP), and the diganglioside GD2.

2.4.1 GD2-41BBζ CAR T cells are active against H3K27M DIPG

2.4.1.1 GD2 CAR T cells mediate potent anti-tumor activity *in vitro*

We generated human GD2-targeting CAR T-cells incorporating a 4-1BBz costimulatory domain (GD2-CAR)[30] (**Figure 1e**) and observed significant GD2-dependent killing (**Figure 1f**) and cytokine generation (**Figure 1g**) upon exposure to patient-derived DIPG cultures relative to control CD19-CAR T-cells incorporating 4-1BBz (CD19-CAR)[21]. Notably, GD2-CAR T-cells do not produce significant cytokines or induce cell killing when exposed to the H3WT, GD2-negative VUMC-DIPG10 patient-derived DIPG culture, providing evidence of therapeutic specificity of GD2-CAR T-cells toward H3K27M DIPG. To further confirm the targeting specificity of GD2-CAR T-cells, we used CRISPR-Cas9-mediated deletion of GD2 synthase (B4GALNT1) in patient-

derived DIPG cells to generate GD2 knockout DIPG cells. Loss of GD2 antigen expression eliminated cytokine production by the GD2-CAR T-cells in comparison to untreated or DIPG cells electroporated with a control guide sequence targeting the AAVS1 locus (**Figure 1h**).

This CAR is delivered via a retroviral vector that was generated by cloning the 14g2a scFv into a CAR with a CD8a transmembrane and hinge region, 4-1BB costimultory domain, and CD3ζ.

Figure 1: GD2 is an immunotherapy target in DIPG

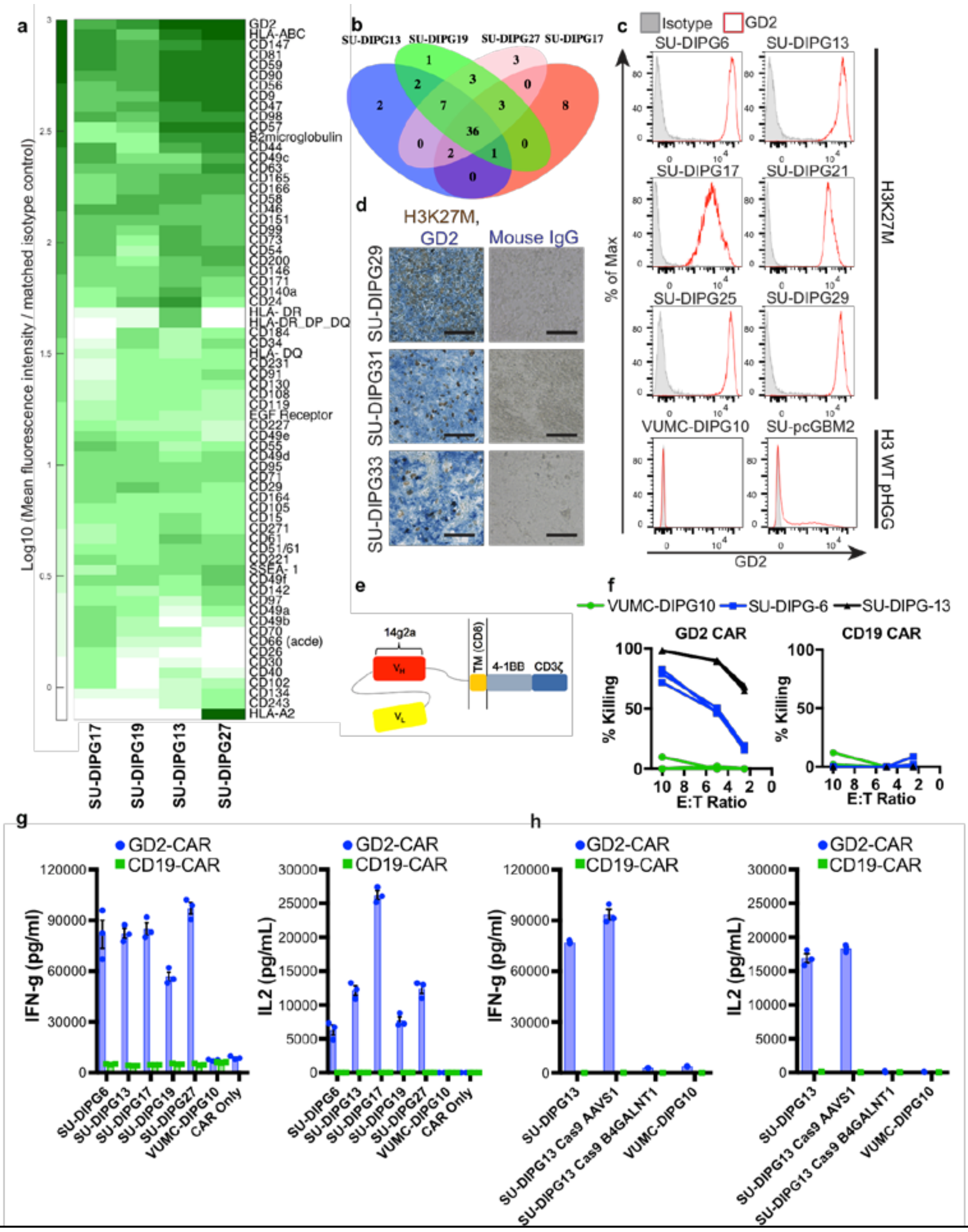

Figure 1: (**a**) Top 68 cell surface antigens expressed on DIPG as determined using flow cytometry screening of a monoclonal antibody panel in patient-derived DIPG cell cultures. (**b**) Assessment of hit overlap between screened cultures identified a total of 36 hits present at an median fluorescence intensity (MFI) of at least 10 times isotype control in all screened cultures. (**c**) Flow cytometry staining of histone 3 K27M DIPGs reveals high, generally homogeneous GD2 expression in contrast to histone 3 WT pediatric high-grade glioma cultures VUMC-DIPG10, diagnosed as a DIPG, and SU-pcGBM2, which arose in cortex. (**d**) Double immunohistochemistry of primary DIPG tumor specimens utilizing an antibody against mutant H3K27M (brown) to identify tumor cells and the anti-GD2 mAb 14g2a (blue) reveals extensive local GD2 expression in primary DIPG (scale bar = 100 microns). (**e**) Schematic of the GD2.4-1BB.z-CAR utilized in functional experiments. (**f/g**) GD2-CAR, but not CD19-CAR T cells, mediate potent lysis (**f**) and produce high levels of IFNγ and IL-2 (**g**) following co-culture with $GD2^{hi}$ H3K27M DIPG cells, but not $GD2^{lo/neg}$ H3WT tumor cells. (**h**) GD2-CAR T cells do not produce substantial levels of IFNγ or IL-2 following co-culture with H3K27M $GD2^{neg}$ line generated using CRISPR/Cas9 to knockout GD2 synthase compared with unmodified control cells or Cas9 targeting the control AAVS1 locus. Data as shown are mean±SEM.

2.4.1.2 GD2 CAR T cells mediate potent anti-tumor activity *in vivo*

To evaluate *in vivo* efficacy of GD2-CAR T-cells against DIPG, we prepared orthotopic mouse xenografts of DIPG cultures derived from post-mortem patient tissue. DIPG cultures were transduced with a luciferase-expressing construct to enable longitudinal monitoring of tumor burden. These xenograft models faithfully recapitulate the diffusely infiltrating histology of DIPG[31], [42]. Mice were distributed by tumor burden into equivalent treatment and control groups before receiving $1x10^7$ GD2-CAR or CD19-CAR T-cells by a single intravenous injection 7-8 weeks after establishment of pontine xenografts. Within 40 days post-treatment (DPT), marked reductions in tumor burden were observed across two independent GD2-CAR T-cell treated cohorts of mice bearing SU-DIPG6 xenografts[27],[21] (**Figure 2a)**. Similar results were observed in a second patient-derived xenograft model, SU-DIPG13FL[42] (**Figure 2e**). All GD2-CAR treated animals demonstrated complete tumor clearance by bioluminescence imaging. By contrast, no mice in the CD19-CAR T-cell control groups exhibited significant tumor regression[21]. At 50 DPT brains were harvested, and immunostaining for the mutant histone H3K27M – present in all engrafted tumor cells – revealed that GD2-CAR treated tumors had been largely eradicated (**Figure 2c,d,g,h,i**).

Most patient-derived orthotopic DIPG xenograft models require many months for lethality, limiting the ability to monitor survival benefit due to development of xenogeneic graft versus host disease (GVHD) after treatment with human T-cells[43]. We therefore used SU-DIPG-13P*, a model that exhibits a dense pattern of growth histologically[44], and is consistently lethal within one month. Substantial improvement in survival was seen in GD2-CAR treated animals compared with CD19-CAR treated controls (**Figure 3a**). GD2-CAR treated animals that survived the initial phase of glioma clearance returned to a visibly healthy state indistinguishable from untreated immunodeficient mice until the onset of GVHD symptoms 4+ weeks after CAR administration that invariably triggered endpoint criteria. Histologic analysis of the brains of endpoint GD2-CAR treated animals reveals clearance of this high-burden tumor while surrounding neural tissues appear grossly normal (**Figure 3b**).

Figure 2: GD2-CAR T cells mediate potent and lasting antitumor response in DIPG orthotopic xenografts

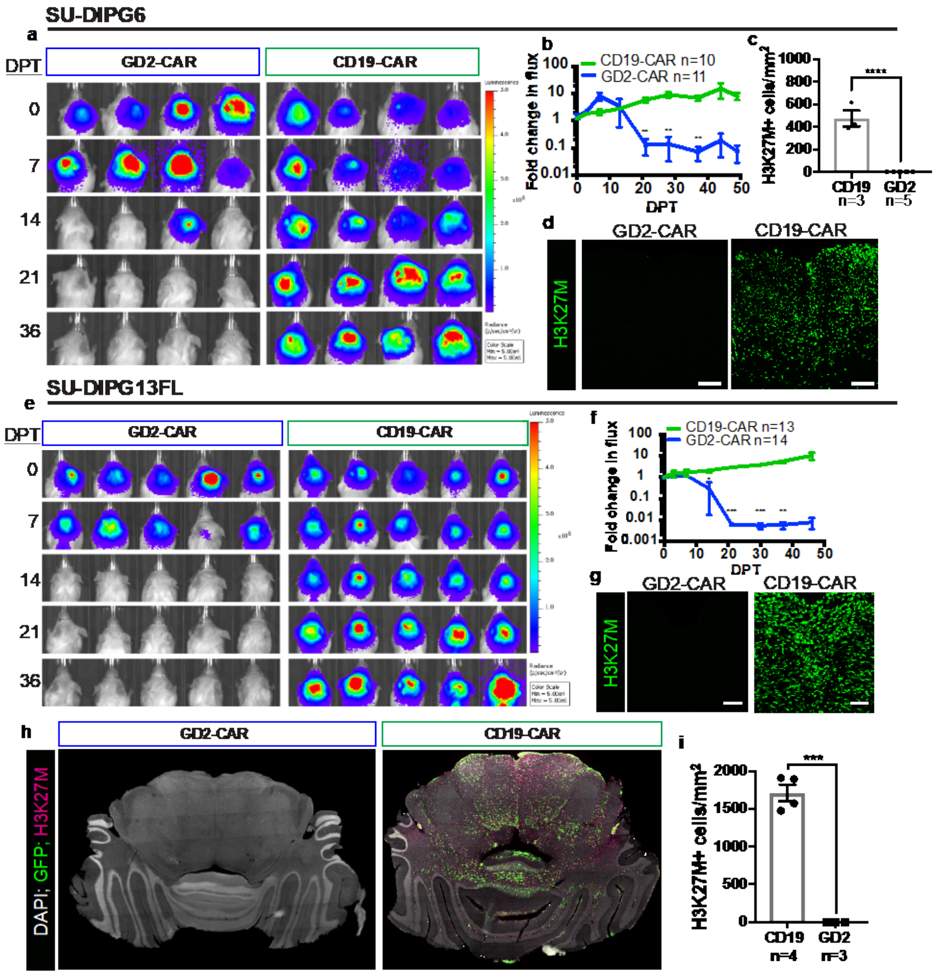

Figure 2: (**a**) Bioluminescence imaging of NSG mice xenografted with luciferase-expressing SU-DIPG 6 into the pons (color map for all images: radiance, min = 5E4, max = 5E6) and infused intravenously with 1E7 GD2-CAR or CD19-CAR T cells as designated. Between 14 and 28 days post treatment (DPT), a dramatic and universal antitumor response is observed in GD2-CAR T cell treated mice, while regression was not observed in those treated with CD19-CAR T cells. (**b**) Substantial reduction in luminescence flux at the tumor site occurred within 28 DPT. (**c**) Quantification of H3K27M+ tumor cell density within infiltrated brainstem regions of SU-DIPG6 GD2-CAR (n=5) vs. CD19-CAR (n=3) T cell treated mice. No region with even marginal tumor density at the injection site or elsewhere was found within GD2-CAR T cell treated animals. (**d**) Representative immunofluorescence confocal microscopy of CD19-CAR and GD2-CAR treated SU-DIPG6 tumors staining for the mutant histone H3K27M demonstrates clearance of tumor in GD2-CAR T cell treated animals. (**e,f**) In an additional patient-derived orthotopic xenograft model of DIPG, SU-DIPG13FL, tumor burden is substantially reduced within 21 DPT. (**g**) Representative immunofluorescent confocal microscopy of SU-DIPG13FL xenografts treated with CD19- or GD2-CAR T cells reveals clearance of H3K27M+, GFP+ tumor cells. (**h**) Tiled immunofluorescence images across infiltrating tumor regions identify no localized residual tumor burden. (**i**) Quantification of H3K27M+ tumor cell density within infiltrated brainstem regions of SU-DIPG13FL. Data as shown are mean±SEM. ****p<0.0001, ***p<0.001, **p<0.01, *p<0.05 by unpaired 2-tailed Student's t-test with Holm-Sidak correction for multiple comparisons applied for bioluminescence imaging data. Scale bars = 100 microns.

To better understand the etiology of treatment-related toxicity in these DIPG xenograft models, we examined the brains of treated SU-DIPG6 xenograft-bearing mice acutely at DPT14 (**Figure**

3c). GD2-CAR treatment was accompanied by a widespread inflammatory infiltrate involving brain parenchyma, meninges and ventricles that was most prominent in the brainstem. Ventriculomegaly was observed, consistent with hydrocephalus. We observed histologically normal-appearing neurons present throughout the pons, hippocampus, and cortex of GD2-CAR T-cell-treated animals with no evidence of neuronal cell killing nor other tissue destruction in this model (**Figure 3c**). Thus, neuropathological evaluation indicates that the toxicity described above results from brainstem inflammation and hydrocephalus due to fourth ventricular compression during the tumor-clearing interval and not on-target, off-tumor toxicity of GD2-CAR T-cells.

Figure 3: GD2-CAR T cell therapy improves survival in DIPG orthotopic xenografts

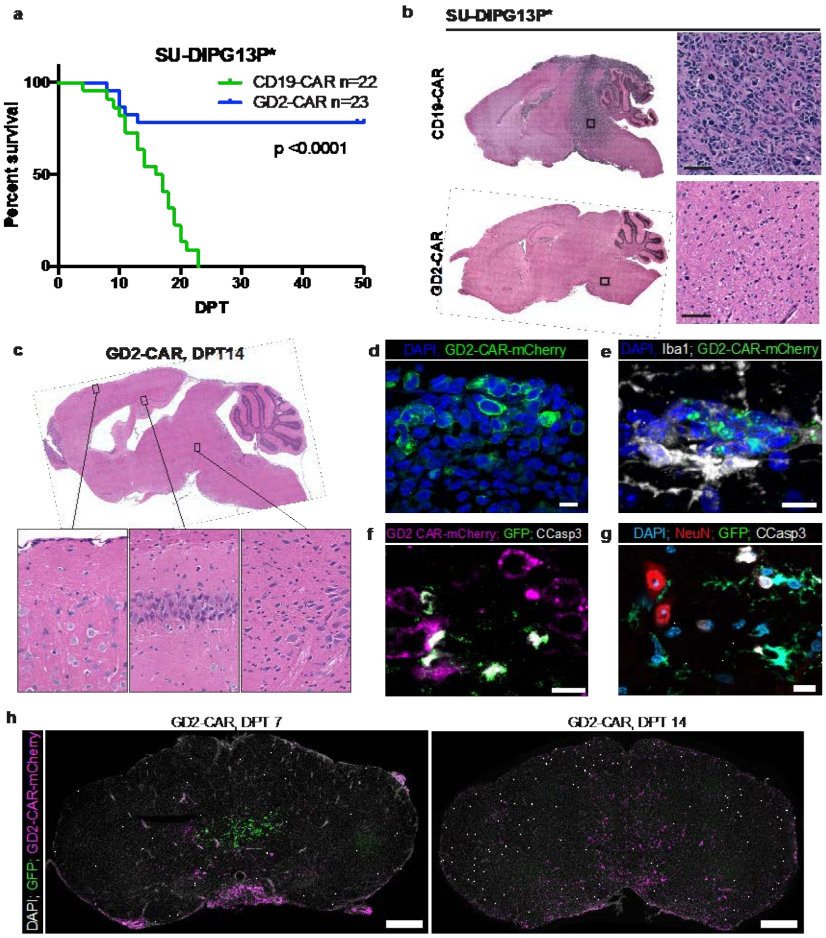

Figure 3: (**a**) Survival analysis of GD2-CAR T cell treated orthotopic xenografts in SU-DIPG-13P*, a particularly aggressive patient-derived xenograft model of DIPG that is lethal within one month of engraftment, reveals a robust survival improvement in GD2-CAR T cell treated animals (p<0.0001 Log-rank (Mantel-Cox) test,n=22 CD19-CAR and 23 GD2 CAR across 3 independent cohorts). While CD19-CAR T cell treated xenografts were universally lethal by study endpoint, all GD2-CAR T cell-treated animals that survived the acute toxicity of therapy survived to study endpoint at which time they manifested GVHD-like symptoms. (**b**) Hematoxylin-eosin staining of SU-DIPG13P* xenografts at DPT50 demonstrate clearance by GD2-CAR T cells of highly-infiltrative parenchymal tumor observed throughout the brain in CD19-CAR T cell-treated controls and normal gross tissue architecture. **(c)** Hematoxylin-eosin staining of SU-DIPG6 GD2-CAR T cell-treated xenograft analyzed at DPT14 demonstrates ventriculomegaly but histologically normal-appearing neurons in cortex, hippocampus, and brainstem (inset images). (**d**) Fluorescence microscopy of DPT7 SU-DIPG13FL xenografts reveals intravenously-administered GD2-CAR-mCherry T cells infiltrating the engrafted tumor.

Figure 3: (**e**) Representative image of infiltrating GD2-CAR-mCherry T cells at DPT14 in a SU-DIPG13FL xenografted medulla demonstrates spatial association with Iba1+ macrophages. (**f**) Representative image of GD2-CAR-mCherry T cell-mediated tumor cell killing with apoptosis of GFP+ tumor cells as evidenced by co-localization with cleaved caspase 3+ occurs in proximity to non-apoptotic NeuN+ neurons (**g**) in the xenografted pons, shown here at DPT7. (**h**) Representative images of GD2-CAR-mCherry T cells infiltrating the parenchyma of SU-DIPG13FL xenografts during the period of acute antitumor activity.

To visualize CAR T-cell infiltration into the parenchyma and tumor, we generated GD2-4-1BBz-mCherry and CD19-4-1BBz-mCherry fusion constructs (**Figure 3d**). By DPT7, GD2-CAR T-cells are extensively distributed throughout the leptomeninges of treated animals, leptomeningeal tumor has been largely eradicated, and few mCherry+ cells are present within the brain parenchyma

(**Figure 3h**). By DPT14, mCherry+ GD2-CAR T-cells had widely infiltrated throughout the parenchyma and numerous foci of Iba1+ macrophages (**Figure 3e**) are present in the xenografted site, along with extensive apoptotic cleaved caspase 3+ cells (**Figure 3f**). Notably, very few cleaved caspase 3+ apoptotic cells are neurons as identified by NeuN double immunostaining (10 total apoptotic neurons identified across 4 mice; **Figure 3g**. This supports a model in which intravenously administered GD2-CAR T-cells enter through the meningeal lymphatic system[45], then subsequently infiltrate brain parenchyma, although the mechanism of CAR T-cell trafficking to the tumor remains to be defined. Given that resolution of tumor clearance and ventriculomegaly temporally coincide in treated animals, it is likely that antigen-specific antitumor activity, rather than on-target, off-tumor cell killing, precipitates neuroinflammation and edema during active tumoricidal activity that results in hydrocephalus.

2.5 Previous Human Experience with GD2-CAR T cell Therapies

Several previous and ongoing clinical trials have tested CARs targeting the disialoganglioside GD2. The first trial at Baylor College of Medicine utilized the scFv derived from dinutuximab in a CAR comprised of a TCR-ζ signaling domain but no embedded costimulatory endodomain[33]. This 1st generation GD2-CAR showed limited expansion *in vivo*, consistent with results using CARs targeting other antigens that did not integrate a costimulatory endodomain into the CAR[46]. Nonetheless, the 1st generation GD2 CAR was effective in inducing tumor regression and long-term disease control in a subset of patients. Low level persistence of these engineered T cell CARs were found up to 192 weeks after infusion and were associated with longer survival[33],[34]. These findings suggest that both the target GD2 and the approach have strong merit and that modifying the product based on scientific insights could improve outcome for more patients.

Figure 4: GD2-CAR (OX40.28.z)

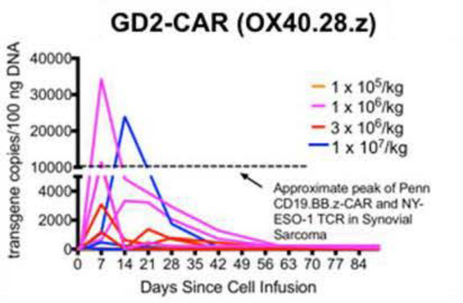

Figure 4: GD2-CAR.OX40.28.z undergo significant dose dependent expansion but show poor persistence *in vivo*. Shown is quantitative PCR based measurement of CAR expressing T cells in the peripheral blood of patients following infusion of CAR engineered T cells for patients enrolled on NCI 14-C-0059.

The Pediatric Oncology Branch NCI and others sought to develop a more potent GD2CART by incorporating costimulatory endodomains into the receptor and two clinical trials have tested GD2-CAR.28.OX40.z.iCasp9 for patients with osteosarcoma and neuroblastoma. Due to the risk for the GD2CART to mediate neurotoxicity due to low level GD2 expression on peripheral nerves and the central nervous system, and the significant peripheral neurotoxicity observed following treatment with dinutuximab for neuroblastoma[19], an inducible caspase-9 gene to was integrated into the vector allowing use of the small molecule dimerizing agent, AP1903, as a safety switch in the event of untoward toxicity[47]. It is important to note, however, that significant toxicity was not observed in this trial and therefore utilization of this safety switch was not necessary.

Results from the Baylor experience using the GD2-CAR.OX40.28.z.iCasp9 CAR after cyclophosphamide/fludarabine conditioning demonstrated robust CAR T cell expansion without toxicity but no objective responses were observed and GD2-CAR persistence and CAR expansion

was not improved by co-treatment with PD-1 blockers[48]. Additionally, they found expansion of myeloid cell populations in patients that may contribute to the limited efficacy and persistence of this CAR[48].

Fifteen patients were treated by investigators on the NCI trial (NCT#02107963) with T cells engineered to express GD2-CAR.OX40.28.z.iCasp9 (osteosarcoma n=12, neuroblastoma, n=3). The preparative regimen employed cyclophosphamide as a single agent and a very cautious dose escalation scheme starting at 1 x 10^5 GD2CART/kg. No dose limiting toxicity and no evidence for neurotoxicity, or other on-target, off-tumor toxicity was observed. AP1903 was never administered. Grade 1 cytokine release syndrome was observed consistent with expansion of GD2CART *in vivo*. Expansion/persistence of GD2CART in patients enrolled on this trial is shown in **Figure 4**. For the purpose of comparison, we include the maximal expansion observed using NY-ESO-1 engineered TCRs in synovial sarcoma[49] and CD19.BB.z CAR for leukemia at Penn (maximal expansion using CD19.28z CAR at NCI is approximately 100,000 copies/100mcgDNA). We conclude from these results that the GD2-CAR.OX40.28.z.iCasp9 T cells undergo substantial expansion *in vivo* but that they do not persist beyond 60 days. This data provides important evidence for the safety of GD2-CAR therapy since no significant toxicity was observed despite expansion of the engineered T cells *in vivo* to levels associated with clinically meaningful anti-cancer effects in other diseases. The data also illustrates the limited tumor exposure to the GD2CART accomplished using this platform due to their short persistence. Given the slow pace of responses seen with immunotherapy in solid tumors, we hypothesize that engineering the GD2-CAR platform to enhance persistence and/or functionality is essential if we are to increase the likelihood that meaningful antitumor effects will be induced against osteosarcoma and neuroblastoma.

2.5.1 Overcoming T Cell Exhaustion

Preclinical data suggests that the basis for the limited persistence/functionality of 14g2a GD2-CARs tested thus far is the development of T cell exhaustion as a result of tonic signaling of the GD2-CAR incorporating the CD28 costimulation domain[30]. This phenomena is substantially reduced, although not eliminated, by incorporation of the 4-1BB costimulatory domain, which appears to protect T cells from the detrimental effects of low level, chronic stimulation. We have demonstrated that the development of early exhaustion due to tonic signaling is a fundamental feature limiting the efficacy of a GD2-CAR incorporating the 14g2a scFv and a CD28 costimulatory domain[21]. We further demonstrated in this manuscript that functionality of the 14g2a CAR is substantially enhanced by switching the costimulatory domain from CD28 to 4-1BB, due to the ability for 4-1BB to provide "anti-exhaustion" effects while CD28 induces "pro-exhaustion" effects. Interestingly, in a follow-up manuscript, when both CD28 and 4-1BB are present, we demonstrated that the "pro-exhaustion" effect of CD28 is dominant [30]. Thus, our preclinical data provides strong rationale for eliminating CD28 costimulation in CARs incorporating 14g2a and for including 4-1BB as the preferred costimulatory domain. Note that we have performed extensive efforts in an attempt to eliminate tonic signaling by mutations in the 14g2a scFv but these have been unsuccessful at creating a scFv that retains good antigen binding properties but lacks tonic signaling properties. Clinical experience with CD28-based versus 4-1BB-based CD19-CARs confirms the propensity for exhaustion since CD19.28.z-CAR show very limited persistence whilst CD19.BB.z-CARs often persist.

We have also attempted to change the linker between heavy and light chain (from a Whitlow linker as in our CD19 CAR to a GS4 x3 linker) but this did not significantly alter cytokine production by the CAR *in vitro*. We also found that adding a long spacer (CH2CH3 domain) in the GD2-4-1BB CAR abrogated its *in vitro* activity (for unknown reasons). In summary, smaller alterations in CAR architecture have not resulted in a convincingly superior product, but altering the costimulatory domain has.

Several trials have previously utilized the [30]14g2a binder and have demonstrated safety and efficacy. The first trials conducted at Baylor utilized the 14g2a binder with no costimulatory domain[33, 34]; in this trial significant antitumor effects were observed with long-term follow-up and no evidence for acute or chronic toxicity. The follow-up trial conducted at Baylor and NCI utilized the 14g2a binder with CD28 plus OX40 costimulatory domains and an Italian trial, conducted under the director of Professor Franco Locatelli, utilizes the 14g2A binder with CD28 and 4-1BB costimulatory endodomains. Fifteen patients have been treated with stage IV neuroblastoma (ages 2 – 18 years of age; 3 at DL1:1e6 cells/kg; 3 at DL2: 2e6 cells/kg; 3 at DL3: 3e6 cells/kg; 7 at the MTD/RD of 10e6 cells/kg) with no DLTs reported during dose escalation. The most common toxicities were grade 1/2 CRS, grade 3/4 neutropenia, thrombocytopenia and anemia[35]. AP1903 was administered in one patient for grade 4 CRS and was associated with clearance of > 90% of CAR-T cells within 48 hours (*personal communication, Locatelli*). Clinical responses (PR or CR) were observed in 10/15 patients infused (3 PD, 1 SD and 1 patient was NED at infusion)[35].

There is an ongoing University College London (UCL) trial which uses the KM666 scFv and a CD28 costimulatory domain. A Chinese trial is underway but the binder utilized is not known. This trial uses a so-called fourth generation CAR (CD28, 4-1BB, and CD27 costimulation). Both the ongoing UCL trial, the Italian trial and the Chinese trial have seen transient responses, but the UCL trial has been marked by limited T cell persistence, consistent with the development of early T cell exhaustion. We are in close contact with Dr. John Anderson, the principal investigator (PI) of the trial ongoing at UCL (NCT#02761915). His trial has climbed to high dose levels without toxicity and they are continuing to accrue (DL1: 1e7 1RG-CART/m^2 on Day 0; DL2: cyclophosphamide (300 mg/ m^2/day X 4 days followed by 1e7 1RG-CART/m^2 on Day 0; DL3: cyclophosphamide (300 mg/ m^2/day X 4 days) and fludarabine (25 mg/m^2/day X 5 days) followed by 1e7 1RG-CART/m^2 on Day 0; DL4: cyclophosphamide (300 mg/ m^2/day X 4 days) and fludarabine (25 mg/m^2/day X 5 days) followed by 1e8 1RG-CART/m^2 on Day 0; DL5: cyclophosphamide (300 mg/ m^2/day X 4 days) and fludarabine (25 mg/m^2/day X 5 days) followed by 5-10e8 1RG-CART/m^2 on Day 0. As more data becomes available from his trial, i.e. the safety and possible efficacy of higher dose levels, we will consider adding additional levels to our trial as necessary.

The trial proposed here will be the only clinical study using the the 14g2a scFv and 4-1BB costimulation exclusively, which based upon the studies presented in Long et al.[30], will substantially enhance persistence compared to those that also incorporate a CD28 signaling endodomain. Given our significant findings surrounding T cell exhaustion resulting in loss of potency and persistence, the strong activity of the GD2-BBz CAR we have demonstrated in multiple papers and disease types[30],[1],[21], we feel it is warranted to use only 4-1BB costimulation.

2.5.2 GD2-CAR Viral Construct

This CAR proposed in this clinical trial is delivered via a retroviral vector that was generated by cloning the 14g2a scFv into a CAR with a CD8a transmembrane and hinge region, 4-1BB costimulatory domain, and CD3ζ as well as Bellicum's iCasp9 safety switch, separated by a ribosomal skip sequence (T2A); manufactured by Bellicum Pharmaceuticals. The viral construct is included in the IND submitted to the FDA.

2.5.3 GD2CART Cells Cultured in Dasatinib

While the 4-1BB costimulatory domain reduces the level of GD2 CAR exhaustion, it does not completely abrogate the effect. The Mackall laboratory has discovered that dasatinib, which is a known inhibitor of lymphocyte-specific tyrosine kinase (LCK), prevents CAR T cell signaling and inhibits CAR signaling[50] through a known mechanism of inhibition of LCK and other SRC tyrosine kinases[51]. In order to limit the exhaustive effects of CAR T cell tonic signaling, we have cultured GD2CART in the presence of dasatinib and found that at the end of the culture period, these cells express lower levels of exhaustion markers (TIM3 and LAG-3) and higher levels of CD62L and CCR7, markers associated with a healthy stem cell memory T cell subset (**Figure 5**a-b). In a xenograft model of osteosarcoma, GD2CART that were cultured in dasatinib until they were administered to mice significantly enhanced anti-tumor efficacy compared to those cultured in media without dasatinib (**Figure 5**).

Figure 5: Effects of Dasatinib on GD2CART

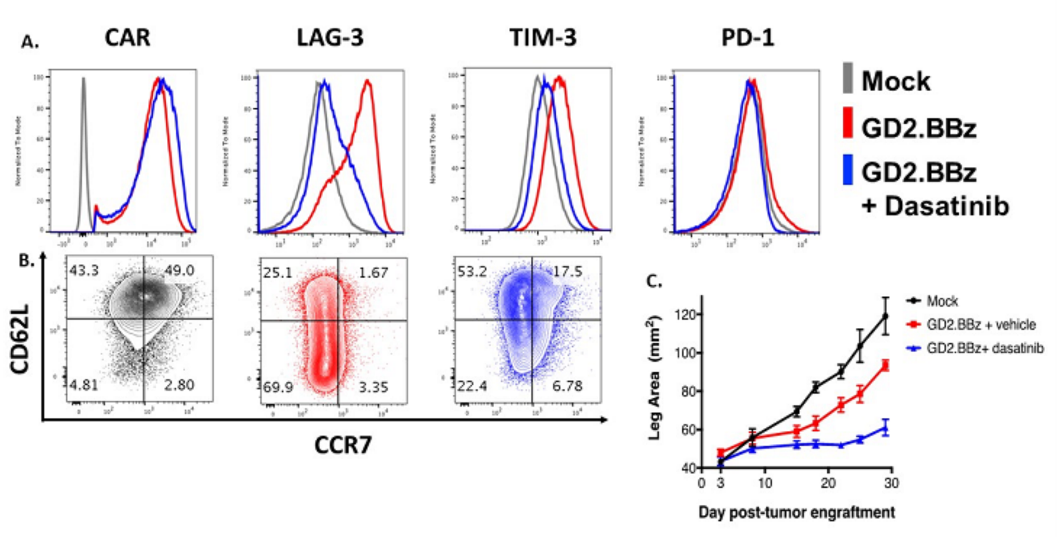

Figure 5: Effects of Dasatinib on GD2.BB.z CAR T Cells. (A) GD2.BB.z CAR T cells cultured in the presence of dasatinib express (A) lower levels of exhaustion markers and (B) higher levels of markers of T stem cell memory on day ten of culture than those cultured in the absence of the drug. (C) Mice were orthotopically injected with osteosarcoma cell line 143b and then treated with GD2.BB.z CAR T cells that were cultured in media with or without dasatinib. (C) Tumor growth was significantly delayed in mice treated with CAR T cells that had been cultured in dasatinib containing media compared to those that had not.

GD2CART cultured in dasatinib are protected from the detrimental effects of tonic signaling, are less exhausted, expand better (**Figure 6**), and we propose, are more effective upon adoptive transfer (**Figure 7**). GD2CART that are cultured in dasatinib have a memory like phenotype while

those cultured without the drug are more slanted towards effector cells. GD2CART will therefore be manufactured with IL-7 and IL-15 in the presence of dasatinib (added on Day 3 and 5) with the goal of infusing a highly potent, non-exhausted CAR.

Figure 6: GD2 CAR T Culture Expansion on the Prodigy

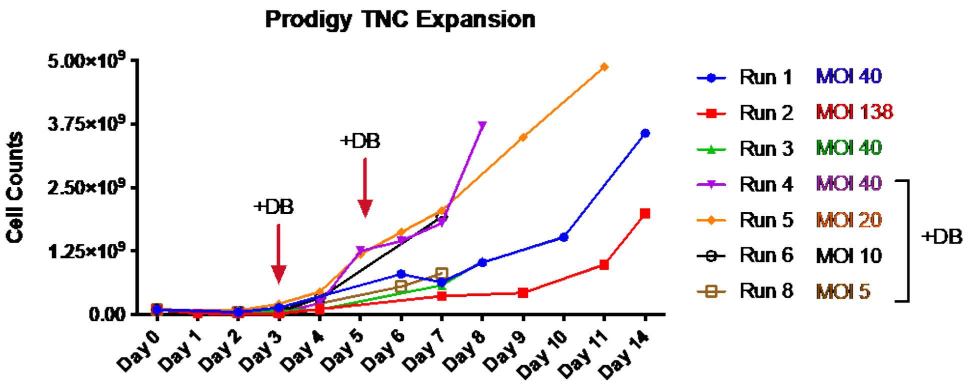

**Figure 6: GD2 CAR T Culture Expansion on the Prodigy.** Culture treatment with dasatinib improves expansion of GD2 CAR T cultures on the Prodigy, compared to control untreated GD2 CAR T Prodigy runs, which demonstrated less expansion by Day 7 harvest, the target for clinical manufacturing. Runs 1-3 were conducted without the addition of Dasatinib. Runs 4 -- 8 were conducted with the addition of Dasatinib (DB) (1 uM).

Figure 7: Improved GD2 CAR T Function with addition of dasatinib.

A)

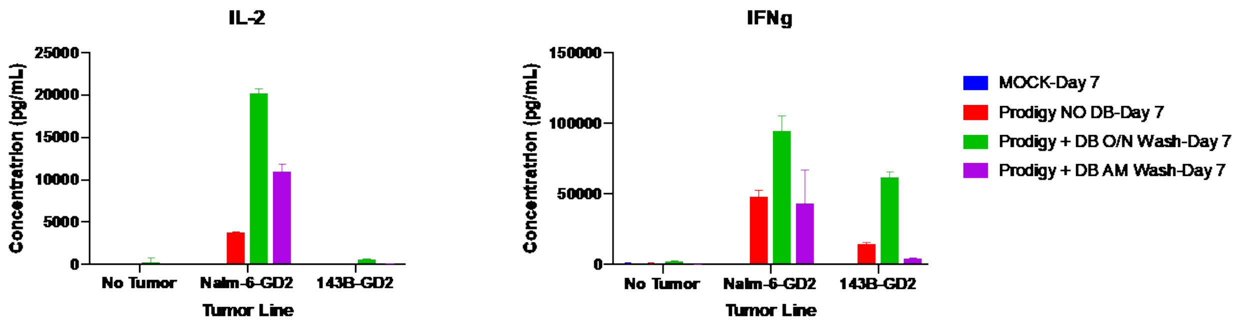

B)

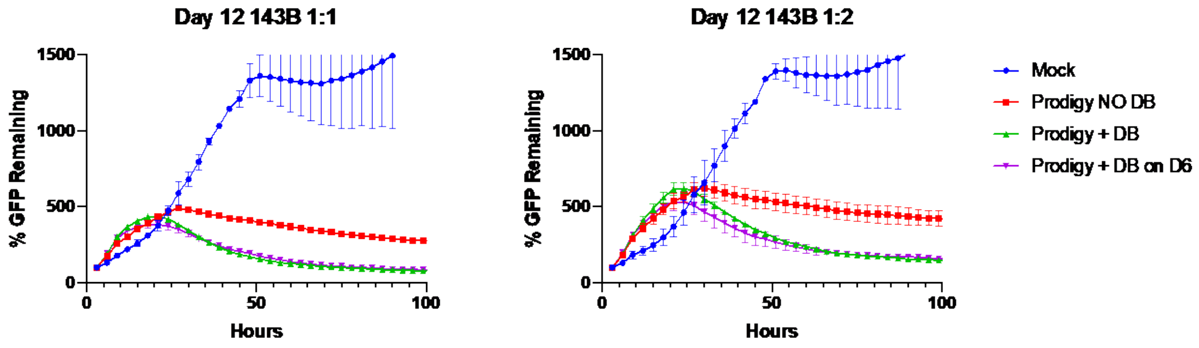

Figure 7: Improved GD2 CAR T Function with addition of dasatinib. A) The addition of dasatinib to the GD2 CAR T cells grown on the Prodigy (green and purple), improved the IL-2 and IFN-gamma cytokine secretion of GD2 CAR T cells against Nalm6-GD2 and 143B-GD2 tumor lines, compared to untreated GD2 CAR T cells (red). Green indicates GD2 CAR T cells grown on the Prodigy were washed out of dasatinib overnight, while purple indicates the cells were washed out the morning of the co-culture. All conditions were tested on Day 7, the target harvest day for clinical manufacturing. **B)** The addition of dasatinib to GD2 CAR T cells grown on the Prodigy (green and purple), improves the killing ability compared to untreated GD2 CAR T cells (red) against a 143B-GD2-GFP$^+$ osteosarcoma line. The Prodigy + DB (green) condition received dasatinib treatment at Day 3 and Day 6, while the Prodigy + DB on D6 (purple) only received dasatinib treatment at Day 6 (1 uM dasatinib). 1:1 and 1:2 represent effector-to-target (E:T) ratios of GD2 CAR T:143B. Mock T cells (blue) exhibited no tumor killing. %GFP remaining represents % Tumor cells remaining from initial co-culture.

2.6 SAFETY CONSIDERATIONS FOR GD2 CAR THERAPY

2.6.1 Risk of chemotherapy:

Toxicities resulting from fludarabine and cyclophosphamide in the doses proposed in the current study are well known and are what have been used in the prior CAR T cell therapy protocols. The preparative regimen is designed to decrease the number of endogenous T cells, including T regulatory cells that may suppress CAR T cell mediated activity, and to induce increased availability of homeostatic cytokines thereby allowing for better engraftment of the transferred CAR T cells. The dose limiting toxicity for both fludarabine and cyclophosphamide is myelosuppression, however myelosuppressive effects are expected to be transient using the doses proposed. Other toxicities including fever, nausea, vomiting, stomatitis, diarrhea, anorexia, edema, skin rashes, myalgias, headache, agitation, and fatigue should be easily managed with appropriate supportive care. Hemorrhagic cystitis can occur in subjects who receive cyclophosphamide, but is unlikely given the relatively low dose administered in this trial and given that continuous intravenous infusion of normal saline will be used prophylactically as a uroprotective agent. Tumor lysis syndrome (TLS) following fludarabine and cyclophosphamide administration can occur in subjects with advanced bulky disease but this has not been described in the setting of brain tumors. Finally, opportunistic infections (protozoan, viral, fungal, and bacterial) have been observed post-fludarabine and cyclophosphamide, especially in heavily pre-treated individuals. Subjects will receive appropriate antimicrobial prophylaxis (e.g., Bactrim for PCP and acyclovir for HSV and VZV prophylaxis) during and after treatment as per institutional standards.

2.6.2 Risk of Autoimmunity:

Autoimmune toxicity is a theoretical risk of adoptive cell therapy trials for cancer and could occur if the transferred populations recognize the target antigen on normal tissues. Thus far, on-target, off-tumor toxicity of both CD19-CAR, CD19/CD22-CAR and CD22-CAR T cells has been restricted to B cell aplasia, which can be managed with immunoglobulin replacement therapy. There were no on-target, off-tumor toxicities noted in the previous GD2-CAR T cell therapy trials. Given that the GD2-CAR T cells tested in this study incorporates scFvs that have already been tested in clinical trials, it is unlikely that unexpected autoimmune toxicity will occur, but subjects will be monitored closely for the occurrence of unexpected toxicity and if it is observed, appropriate supportive care, or if severe, administration of AP1903 to ablate the CAR T cells.

2.6.3 Risks of Gene Therapy:

Risks of gene therapy include insertional mutagenesis or emergence of replication-competent retrovirus (RCR). While insertional mutagenesis is theoretically possible using retroviral vectors, this has only been observed in the setting of infants treated for X-SCID using retroviral vector-mediated gene transfer into CD34+ bone marrow stem cells. In the case of retroviral or lentiviral vector-mediated gene transfer into mature T-cells, there has been no evidence of long-term toxicities associated with these procedures since the first NCI sponsored gene transfer study in 1989.

The proposed protocol will test the cellular product for RCR, and patient follow up will comply with all current FDA guidelines. As the viral vectors used have been engineered to minimize the risk of emergence of replication competent retrovirus, subjects will be tested for RCR with long term follow-up as per FDA Guidance (July 2018). All participants will be followed for several years following receipt of this therapy and in the case of the development of a second malignancy, all efforts will be made to determine whether replication competent retrovirus has emerged.

2.6.4 Risk of Cytokine Release Syndrome:

Cytokine release syndrome has recently been defined as "a supraphysiologic response following any immune therapy that results in the activation or engagement of endogenous or infused T cell and/or immune effector cells"[52]. Symptoms are varied and can be progressive, must include fever at the onset and may include hypotension, capillary leak (hypoxia) and end organ dysfunction"[52], including arrhythmia, cardiomyopathy, heart block, renal failure, pleural effusions, transaminitis and coagulopathy[52]. Cytokine release syndrome (CRS) has been the topic of several reports and reviews[53],[54],[52]. In brief, CRS comprises a febrile, sepsis-like picture that results from hemodynamic and organ effects of supraphysiologic levels of inflammatory cytokines produced directly or indirectly by the activated T cells. Among the most important of these cytokines are IL-6 and IFN-gamma[29],[55],[53]. Laboratory alterations that commonly occur with CRS include C-reactive protein (CRP) and ferritin. One rare toxicity observed in CAR T cell patients, which is thought to overlap with CRS is hemophagocytic lymphohistiocytosis or macrophage activation syndrome (HLH/MAS), as it shares many features with CRS.

CRS can be safely managed with supportive care and in some cases, immunosuppression using anti-IL6R mAbs therapy and corticosteroids[54]. CRS is limited in subjects with low tumor burdens, as CRS severity correlates with the degree of CAR T cell expansion. CRS has been limited in the setting of CAR T cells for solid tumors, likely due to the relatively lower rate and degree of CAR T cell expansion that occurs in this setting. Recently forty-nine experts from experts in immune effector cell therapies, including CAR T therapy met at a meeting supported by the American Society for Transplant and Cell Therapy (ASTCT) and reached consensus on the grading definitions for immune effector cell-associated CRS[52] contained in section **13.2**, Appendix B.

2.6.5 Risk of Neurotoxicity

2.6.5.1 Immune Effector Cell-Associated Neurotoxicity Syndrome [ICANS]

Neurotoxicity is observed in a significant fraction of patients treated with CD19-CAR therapies [29],[55],[53] with toxicity ranging from mild to severe. Symptoms tend to be more diverse than observed with CRS[52]. The pathobiology of CD19-CAR associated neurotoxicity is not fully

understood, but the clinical syndrome is associated with increased expansion of CAR T cells and identification of CAR T cells in the cerebrospinal fluid. Neurotoxicity may be seen more frequently in subjects with CNS leukemia[29], however subjects without documented CNS leukemia or lymphoma can also develop symptoms of neurotoxicity that range from mild to severe. Hence, no direct association between CNS disease burden and neurotoxicity severity has been observed in the context of CD19-CAR therapies for leukemia[29]. The syndrome typically manifests clinically as tremors, dysgraphia, impaired attention, confusion, aphasia and/or dysmetria, and occasionally seizures. Expressive aphasia appears to be a characteristic feature. Radiographic changes are variable and the syndrome typically resolves in 1-2 weeks and appears fully reversible in the vast majority of patients. The prevailing hypothesis regarding the pathophysiology of this syndrome is that it reflects non-specific neurotoxic effects of cytokines and/or activated T cells, rather than a direct on-target effect of CAR T cells. Indeed, the dose limiting toxicity of IL-6 when administered in a Phase 1 trial was neurotoxicity and transient aphasia was observed[56]. In further support of this hypothesis, the Jensen laboratory at Seattle Children's Hospital has recently developed a rhesus model of neurotoxicity that utilizes a CD20-CAR rather than the CD19-CAR platform and the model appears to model the human syndrome well[57]. This provides further evidence against a direct, on-target effect involving CD19 in brain tissue. Lethal neurotoxicity has occurred in <1% of patients treated with CD19-CAR T cells and is associated with cerebral edema and herniation in patients with severe cytokine release syndrome. The occurrence of lethal neurotoxicity is reduced at lower doses of CD19-CAR T cells.

Based upon this experience, the ASTCT consensus group convened in 2017 defined ICANS as "a disorder characterized by a pathologic process involving the central nervous system following any immune therapy that results in the activation or engagement of endogenous or infused T cell and/or other immune effector cells. Symptoms or signs can be progressive and may include aphasia, altered level of consciousness, impairment of cognitive skills, motor weakness, seizure and cerebral edema"[52]. As CARs targeting non-CD19 antigens have not been associated with severe neurotoxicity in clinical trials, we are optimistic that cerebral edema associated with cytokine release syndrome will not occur in this trial, but patients will be observed carefully as discussed below.

2.6.5.2 Neurotoxicities due to tumor location

The neurotoxicities associated with GD2CART in subjects with DIPG could manifest differently than prior cell therapies due to the nature of the GD2 disialoganglioside on cell surfaces of tumors of neuroectodermal origin, as well was the nature and location of the tumor itself. To better understand the etiology of treatment-related neurotoxicity in DIPG xenograft models, we examined the brains of treated SU-DIPG6 xenograft-bearing mice acutely at DPT14 (**Figure 3**c). GD2-CAR treatment was accompanied by a widespread lymphocytic infiltrate involving brain parenchyma, meninges and ventricles that was most prominent in the brainstem. Ventriculomegaly was observed, consistent with hydrocephalus. Despite T cell infiltration, we observed histologically normal-appearing neurons present throughout the pons, hippocampus, and cortex of GD2-CAR T-cell-treated animals with no evidence of neuronal cell killing nor other tissue destruction in this model (**Figure 3**c). Thus, neuropathological evaluation indicates that the toxicity described above results from brainstem inflammation and hydrocephalus due to fourth ventricular compression during the tumor-clearing interval and not on-target, off-tumor toxicity of GD2-CAR T-cells. The uniqueness of the tumor location in this patient population requires the

phase 1 dose escalation design be implemented in this patient population, separate and apart from patients with spinal DMG.

To mitigate the neurotoxicity risks in the DIPG patient population we plan to insert an intraventricular catheter (Ommaya catheter) prior to cell infusion for monitoring and if necessary, rapid and efficient treatment of increased intracranial pressure (ICP). Increased intracranial pressure will be treated by neurologists, intensivists and neurosurgeons who are familiar with the trial using the guidelines shown in section **13.2.4**. Briefly, upon onset of symptoms of increased ICP or evidence for increased ICP upon routine Ommaya monitoring, patients with DIPG will be appropriately positioned, imaged using MRI if possible or CT scan if MRI is not possible. Therapeutic interventions will generally include hypertonic saline, CSF removal via the Ommaya reservoir, and mannitol and dexamethasone. If these measures fail to control the increased ICP, consideration will be given to administering dasatinib to inhibit CAR function[50] and/or administration of AP1903 to permanently ablate the CAR T cells. Avastin may also be utilized as it has shown some efficacy in the setting of tumor associated edema. An algorithm that provides an systematic approach to neurotoxicity management for patients enrolled on this trial is shown in in section **13.2.4**. The algorithm will serve as a guideline but is not meant to supercede clinical judgment in the management of neurotoxicity on this trial, and deviations from the algorithm will not be considered protocol deviations.

The potentially life-threatening intracranial complications of DIPG are not anticipated in the patient population with H3K27M-mutated spinal DMG, but spinal cord inflammation could cause spinal cord dysfunction and in severe cases could cause spinal cord vascular compromise. We will monitor for potential spinal cord toxicities due to inflammation very closely and will also use a systematic approach for neurotoxicity management as outlined in section **13.2.5.** While intended as a measure of clinical benefit, we will monitor neurologic status via a standardized tool in both patient populations which can provide additional assessment data when evaluating for tunor related neurologic toxicity (see **Section 13.8**).

All subjects will receive a conditioning lymphodepletion chemotherapy regimen of fludarabine and cyclophosphamide (which may occur as an outpatient as determined by clinical judgment), followed by hospitalization for intravenous infusion of GD2CART on Day 0 and inpatient or outpatient monitoring with close proximity to the Stanford clinic until at least Day 28 according to the following guidelines.

1. Subjects with DIPG and, at investigator discretion, subjects with DMG will have an intraventricular catheter (Ommaya catheter) placed following enrollment and prior to T cell infusion to allow monitoring, and treatment if necessary, of increased intracranial pressure (ICP).

2. All subjects will receive a conditioning lymphodepletion chemotherapy regimen of fludarabine and cyclophosphamide, followed by infusion of (Day 0). Subjects will be monitored closely as an inpatient for at least 28 days post-T cell infusion or as an outpatient in close proximity to the clinic if all toxicities are resolving/have resolved, at investigator’s discretion, according to the following schedule:

3. Neurological exam: Daily from D0 to Day 28 or 5 times per week if outpatient (with no more than 48 hours between evaluations) with Clinical Evaluation of Neurologic Status (**Section 13.8**) at least weekly post cell infusion to Day 27, on Day 28, and with each follow up evaluation. Increase as clinically indicated.

4. Measurement of ICP via Ommaya Reservoir (with research sample collection): baseline D0 (prior to infusion), Day 3, Day 7, Day 10, Day 14, Day 18, Day 21, Day 24, Day 28, or as clinically indicated. If any evidence of increased intracranial pressure or clinical deterioration suspected due to neurologic compromise, patient will be transferred to the ICU for more intensive ICP monitoring. Modification of this schedule is at the investigator's discretion and will not be considered a protocol deviation.

5. Measurement of pressures via lumbar puncture (LP) in subjects with spinal DMG (with research sample collection) [Optional]: Day 7, Day 10 and Day 14 or as clinically indicated, additional meaurements may be taken at Day 0, Day 21 and Day 28. If any evidence of toxicities that would benefit from a clear determination of pressure, additional LP measurements may be undertaken. Modification of this schedule is at the investigator's discretion and will not be considered a protocol deviation.

6. Radiographic imaging of subjects with DIPG: MRI at baseline (within 7 days of Day -4), D7, D14, D21 and D28. If clinical condition post-infusion prevents MRI, a CT will be obtained on those days. The standard MR parameters are listed on the PBTC NIC web page located at http://www.childrenshospital.org/research/centers-departmental-programs/pediatric-brain-tumor-consortium-neuroimaging-center under Neuroimaging Studies/ Specific MR Imaging Sequences- Open PBTC Protocols.

7. Neurooncologists, neurosurgeons and neurointensivists will be consulted and involved in every case and neurointensive care provided for management of neurologic deterioration and/or subclinical increase in intracranial pressure or spinal pressure, guided by the algorithm shown in section **13.2.4** and section **13.2.5**. The algorithms will serve as guidelines but are not meant to supercede clinical judgment in the management of neurotoxicity on this trial, and deviations from the algorithms will not be considered protocol deviations.

8. Consideration may also be given to administering dasatinib to inhibit CAR function[50] and/or administration of AP1903 to permanently ablate the CAR T cells if the patient develops uncontrolled, life threatening toxicity.

2.7 Risk for on-Target Toxicity and Rationale for The integrated suicide domain (iCasp9)

Anti-GD2 monoclonal antibodies cause pain requiring continuous infusion of narcotics for analgesia due to their interaction with peripheral nerves and possibly engagement of the complement system[58],[59],[19]. Notably however, clinical trials of CAR T cells targeting GD2 have not resulted in significant toxicity despite significant CAR T cell expansion and signs of on-tumor efficacy. Neither CNS toxicity nor peripheral neuropathy has been observed following GD2-CAR therapy in clinical trials. Furthermore, our murine model, which is relevant for assessing toxicity since GD2 is identical in mice and humans, demonstrated no evidence for on-target toxicity on normal neural tissue.

Richman, et al.[60] reported poorly characterized toxicity following administration of a CAR that incorporated the E101K scFv which is a mutated form of 14g2a. We published a response to this manuscript detailing major concerns regarding the conclusions published in this work[61]. To summarize, the relevance of toxicity seen with the E101K binder to that expected with 14g2a is questionable since the affinity is higher and it remains possible that this mutated binder could also acquire cross-reactivity with other gangliosides. To add to the concerns, our group also made a

CAR consisting of the same E101K mutated "high-affinity" binder and demonstrated substantial antitumor activity in mice with no evidence for CNS toxicity[62]. This is across experiments in more than 200 mice (including those in our Letter to the Editor[61]. The authors of the cited manuscript also attempted to implicate a 3F8 (alternative scFv) based CAR as causing the same symptoms in mice, but the authors of a different manuscript on a 3F8 based CAR saw no such neurotoxicity[63]. It is unclear what is causing the toxicity in the models at this one center, but we offer some possible explanations in our letter (CRS/CRES, cross reactivity of the mutated binder with a different ganglioside, etc.)[61].

As described above, several clinical trials of 14g2a based CAR T cells, including those resulting in robust T cell expansion and clinically significant remissions and no patients have developed neurotoxicity. No patients in the ongoing UCL trial have developed neurotoxicity either and none were reported in the Chinese trial. Nonetheless, given that the GD2.BB.z-CAR has been designed to be more potent than those incorporating the CD28 costimulatory domain previously tested in clinical trials due to diminished T cell exhaustion, it is possible that we could observe on-target neurotoxicity related to low levels of GD2 on normal neural tissues. Furthermore, as noted above, even in the absence of on-target toxicity involving normal neural tissues, we could also observe toxicity related to tumor swelling and subsequent hydrocephalus or swelling of the brainstem itself, which could adversely impact vital functions. Due to the potential risk for toxicity therefore, we have incorporated a "suicide switch" within the CAR T cell receptor to allow for rapid ablation in the event of unacceptable toxicity. The iCasp9 system has been vetted in clinical trials for allogeneic transplant and results in efficient and rapid ablation of T cells and is the [60, 61, 63, 64] most effective suicide domain available to date, which provides an important measure of safety. We have also recently demonstrated that dasatinib can inhibit CAR signaling and could be utilized in the context of untoward toxicity[56], which may be utilized in this trial if untoward toxicity is observed. This trial will enroll patients with H3K27M diffuse pontine glioma (DIPG) and patients with spinal H3K27M DMG who lack any curative options, thus justifying the administration of a therapeutic with potential risk.

Diffuse midline gliomas (DMG) of the pons (also called diffuse intrinsic glioma, DIPG) and the spinal cord (spinal DMG) each confer a distinct set of possible toxicities associated with CAR T cell therapy. In the pons, on-target, on-tumor effects of therapy could result in brainstem swelling that results in brainstem dysfunction, hydrocephalus, and increased intracerebral pressure with possible herniation syndromes. With spinal cord glioma, these potentially life threatening intracranial complications are not anticipated but spinal cord inflammation could cause spinal cord dysfunction and in severe cases, could cause spinal cord vascular compromise. We therefore propose that pontine and spinal cord H3K27M+ DMGs require dedicated dose-finding arms of the trial. However, as the potential toxicity for pontine DIPG immunotherapy is more severe, establishing safety in the pontine DIPG arm can inform the spinal cord arm. Spinal cord DMG is rarer than pontine DIPG, so the spinal cord arm is expected to accrue more slowly and we anticipate fewer participants and the trial would be unlikely to accrue sufficient number of patients to complete a full dose escalation in patients with spinal DMG. Therefore to avoid unnecessary exposure of patients with spinal DMG to subtherapeutic doses while preserving safety and maintaining rapid escalation to therapeutic dose ranges, we propose two separate dose finding efforts for spinal and pontine DMG in which spinal DMG patients can be advanced to the dose level shown to be safe for patients with pontine DIPG (but not vice versa).

2.8 CORRELATIVE STUDIES BACKGROUND

1. Measure expansion/persistence/phenotype of adoptively transferred GD2CART in the CSF and blood and correlate this with antitumor effects.
2. Conduct analyses of the manufactured T cell product and blood and CSF post-infusion to identify biomarkers associated with clinical benefit and/or enhanced CAR T cell expansion and/or persistence.
3. Assess whether changes in the level of ctDNA in the cerebrospinal can provide prognostic information and/or information regarding clonal evolution of DIPG over time.
4. Evaluate whether antigen expression or tumor microenvironment are correlated with response to CAR T cell.

2.9 STUDY DESIGN

2.9.1 Short Title for Study

GD2CART in DIPG or spinal DMG

2.9.2 Interventional model

One arm, open label single treatment

2.10 PROTOCOL RATIONALE AND SUMMARY

Diffuse Midline Gliomas (DMG) harboring the H3 K27M mutation, including diffuse intrinsic pontine glioma (DIPG) are lethal, high-grade pediatric brain tumors that are inoperable and pose significant challenges for treatment. Diffuse Intrinsic Pontine Glioma (DIPG) is a devastating, aggressive brain tumor of childhood arising in the ventral pons and comprises approximately 10-15% of pediatric brain tumors, with half of all pediatric malignant gliomas occurring in the brainstem[5]. While radiation therapy constitutes the mainstay of treatment, it only provides temporary improvement or stabilization of symptoms, extending overall survival by only 3 months. Prognosis is bleak with 90% of children expiring due to disease within 2 years of diagnosis. Hence there is an urgent need for novel effective therapies in this disease.

Chimeric Antigen Receptor (CAR) expressing T-cells is a new therapy wherein a subject's own T-cells are harvested and subsequently genetically modified in order to target cell surface antigens on specific cancer cells. In addition to their specificity, these CAR T-cells can be modified to be highly proliferative and possess the ability to negate immunosuppressive mechanisms making them ideal agents against highly aggressive cancers. We screened cell surface antigens in DIPG cultures in an attempt to identify potential targets for CAR T cell immunotherapy as a potential effective therapy. We observed that the disialoganglioside GD2 was expressed at high levels on each of twelve patient-derived DIPG cultures screened.

The primary goal of this trial is to evaluate the feasibility of generating GD2CART to meet the manufacturing specifications in children and young adults with H3K27M DIPG or spinal H3K27M DMG, to establish the safety and RP2D of GD2CART in subjects with H3K27M DIPG and spinal H3K27M DMG. In a preliminary fashion, this study will assess clinical benefit in an expanded cohort of subjects with DIPG and with DMG treated at RP2D.

Because GD2CART have not been previously administered to individuals with DMG (pontine or spinal), a dose finding 3 + 3 dose escalation design will initially be used in the two disease cohorts separately. Given the dismal survival prognosis of this disease with a median age at diagnosis of 6.3 years and the fact that children usually die from the disease within 2 years of initial diagnosis, children will be eligible for this first-in-DMG cell therapy clinical trial. A conservative staggering protocol between patients will allow investigators to mitigate risk to the numbers of patients treated. Doses of GD2-CAR T cells of 1 x 10^7/kg have been administered in other clinical trials without toxicity, but to mitigate risk we have chosen a conservative starting cell dose of 1e6/kg, with a careful dose escalation (dose level 2: 3e6 transduced T cells/kg; dose level 3; 10e6 transduced T cells/kg). Spinal cord DMG is rarer than pontine DIPG, so the spinal cord arm is expected to accrue more slowly and we anticipate fewer participants, and it is quite likely that we will not have sufficient numbers to complete a separate dose escalation in spinal cord DMG. Therefore to avoid unnecessary exposure of subjects with spinal DMG to subtherapeutic doses that have already been demonstrated to be safe in DIPG, while preserving rapid escalation to thera[peutic dose ranges, we propose to allow the safety data during dose escalation of subjects with pontine DIPG to inform the safety of the spinal DMG (but not vice versa.

Once the MTD/RP2D is determined for each DMG category, an expansion cohort of subjects with H3K27M DIPG and 10 subjects with spinal DMG will be treated to further explore safety and conduct a preliminary evaluation of efficacy.

To maximize safety of administration in the DIPG patient population, subjects will be carefully selected to minimize the risk of tumor swelling induced herniation or cord compression. Specifically, patients with thalamic lesions will not be eligible to reduce the risk of herniation as a result of tumor swelling. Subjects with biopsy documented H3K27M DIPG or with spinal H3K27M DMG will be eligible if at least 6 weeks have elapsed since completion of first line radiation therapy. In addition, close monitoring during participation in this trial will be tailored to the tumor location and access to immediate intervention should toxicities emerge is clearly outlined for each group (pontine DIPG and spinal DMG) See sections **13.2.4** and **13.2.5** respectively.

A secondary objective will assess the capacity of AP9013 to mediate clearance of the genetically engineered cells to resolve toxicity, if unacceptable toxicity occurs (see section **12.5**) that is possibly, probably or likely related to the GD2CART.

Correlative analyses on this protocol or a companion study are defined above.

The precedent for conducting clinical trials of this scope was established by the ongoing clinical trials CCT5001/IRB-41382 and CCT5007/IRB-41383 conducted by Stanford Center for Cancer Cell Therapy.

This study will be conducted at Stanford by the principal investigator Michelle Monje, M.D., Ph.D. The Sponsor of this study will be Crystal Mackall, MD, Professor Pediatrics & Medicine, Associate Director, Stanford Cancer Institute.

2.10.1 Primary Outcome Type

Safety and Feasibility and RP2D

2.10.2 Investigational Agent

GD2CART: Autologous T cells transduced with retroviral vector (GD2.BB.z.iCasp9) Chimeric Antigen Receptor (GD2-CAR) cultured with dasatinib; following Fludarabine and Cyclophosphamide

2.10.3 IND number:

IND # 19801, Sponsor: Crystal L. Mackall, M.D.

2.10.4 Primary outcome measures:

This study will be registered on ClinicalTrials.gov but is not subject to the results reporting requirement.

1. Title: Rate of successful manufacture of GD2CART using a retroviral vector in the Miltenyi CliniMACS Prodigy ® system
    - Outcome Measure1: The percentage of apheresis samples (fresh or frozen) that are successfully processed and expanded to manufacture GD2CART that satisfy the target dose level and meet release specifications will be determined for each dose cohort.
    - Outcome Timeframe1: 14 days after apheresis or thawing of cryopreserved peripheral blood mononuclear cells (PBMCs).
2. Title: MTD/RP2D of GD2CART in subjects with H3K27M DIPG and subjects with spinal H3K27M DMG
    - Outcome Measure2: Incidence and severity of dose limiting toxicities (DLTs) following chemotherapy preparative regimen and infusion of GD2.BB.z.iCasp9-CAR T cells (GD2CART), as recorded and graded according to Common Terminology Criteria for Adverse Events (CTCAE) version 5.0 and Appendix B, section **13.2**, at each dose level tested in subjects with H3K27M DIPG and spinal H3K27M DMG following standard upfront radiation therapy.
    - Outcome Timeframe2: 28 days after infusion of GD2CART
3. Safety of GD2CART in subjects with H3K27M DIPG and spinal H3K27M DMG treated at the RP2D
    - Outcome Measure3: Suspected adverse events and serious adverse events following chemotherapy preparative regimen and infusion of GD2CART, as recorded and graded according to Common Terminology Criteria for Adverse Events (CTCAE) version 5.0 and Appendix B, section **13.2**.
    - Outcome Timeframe3: 28 days after infusion of GD2CART

2.10.5 Secondary Outcome Measure

1. Title: Clinical benefit of GD2CART at RP2D in children and young adults with H3K27M DIPG and with spinal H3K27M DMG

- Outcome Measure4: Primary determinant of clinical activity in subjects with DIPG and subjects with spinal H3K27M DMG will be overall survival (OS) at 12 months post-diagnosis compared to historical controls.

In addition, progression free survival (PFS), post-progression survival (PPS), and radiographic and clinical response will be assessed as best response (i.e. complete response [CR], partial response [PR], stable disease [SD], or progressive disease [PR]) at Day 28, 3 months and 6 months post-infusion.

- Timeframe4: Day 28, 3 months, 6 months, 9 months and 12 months and 24 months post GD2CART.

2. Title: Ability of AP1903 to eliminate persistence of genetically engineered cells, and allow resolution of toxicity in the event unacceptable toxicity considered possibly, probably or definitely related to GD2CART.

- Outcome Measure5: Resolution of toxicity to ≤ grade 2 within 72 hours of administration of AP1903
- Timeframe5: 72 hours after administration of AP1903

2.10.6 General Study Design

This is a phase 1, open label, single site dose escalation trial of GD2CART following cyclophosphamide/fludarabine lymphodepleting chemotherapy in subjects with H3K27M DIPG and spinal H3K27M DMG following at a minimum, completion of standard, up front radiotherapy. Given the universally fatal outcome in these patients, the very short post-progression survival and the increased risk with bulky disease identified in murine models[7], patients will be eligible for enrollment when they are at least 6 weeks from completion of standard upfront radiotherapy, regardless of documented evidence of progression, if all other eligibility criteria are met. In addition, given the established human safety data in administration of GD2-CARs and the median age of presentation, the dose escalation cohort will not be restricted by patient age. The eligibility criteria for dose escalation in DIPG was chosen based of the following considerations:

1) A requirement for documented progression prior to enrollment is not feasible, since radiographic progression cannot be reliably distinguished from radionecrosis in all patients.
2) Post-progression survival is very short (median 2.3 months) and may not be long enough for patients to benefit from the effects of the GD2CART.
3) Median progression free survival following radiotherapy is 7.0 months (80.8% demonstrating progression within 12 months), therefore the risk of progression beyond 3 months is sufficiently high to justify the risks and morbidity associated with the investigational treatment regimen.
4) Preclinical models of DIPG demonstrate that bulky disease is a risk factor for treatment related morbidity and mortality due to hydrocephalus, therefore enrolling patients prior to documented clinical or radiographic progression will increase the likelihood that the therapy can be rendered safely.

In the pons, on-target, on-tumor effects of therapy could result in brainstem swelling that results in brainstem dysfunction, hydrocephalus, and increased intracerebral pressure with possible herniation syndromes. With spinal cord glioma, these potentially life threatening intracranial complications are not anticipated but spinal cord inflammation could cause spinal cord dysfunction and in severe cases, could cause spinal cord vascular compromise. We therefore propose that

pontine and spinal cord H3K27M+ DMGs require dedicated dose-finding arms of the trial. However, as the potential toxicity for pontine DIPG immunotherapy is more severe, establishing safety in the pontine DIPG arm can inform the spinal cord arm and allow for rapid dose escalation while minimizing the number of subjects exposed to subtherapeutic dose ranges.

Once the MTD/RP2D is established in both disease categories, the study will continue to evaluate safety of administration, feasibility of manufacturing, but will also conduct a preliminary assessment of clinical benefit in children and young adults with H3K27M DIPG and spinal H3K27M DMG. The CAR vector will incorporate an inducible Caspase 9 that can lead to efficient T cell apoptosis following exposure to AP1903 should toxicity require inactivation of the cell product.

Eligible subjects will undergo leukapheresis to obtain starting material for the CAR T cells. Cryopreserved PBMC stored from participation in other institutional cell therapy or cell collection studies or performed as standard collections may be used to generate the cellular product on this study as long as they meet the criteria established in this IND. In brief, cryopreserved PBMC will undergo selection, activation, transduction with the retroviral vector, expansion, and formulation in a GMP Facility using the Miltenyi CliniMACS Prodigy® system for the manufacture of GD2.BB.z.iCasp9-CAR T cells. The product will be cryopreserved and transferred to Stanford's Cell Therapy Facility (CTF), from which the product will be distributed to the patient care unit for infusion.

Prior to infusion, patients will receive a lymphodepleting chemotherapy preparative regimen of fludarabine and cyclophosphamide (fludarabine 25 mg/m^2/d x 3 days and cyclophosphamide 500 mg/m^2/d x 3 days) on Days -4, -3, -2, followed by infusion of GD2CART on Day 0. Subjects will be closely monitored for cell therapy toxicities, including monitoring of intracranial pressure in subjects with DIPG (see secton 13.2.3.5) and monitoring for spinal cord dysfunction in subjects with spinal DMG as outlined in section 4.2.4. Subjects will be evaluated after cell infusion for toxicity, antitumor effects and for persistence of CAR in blood samples and functionality of transduced T cells. Additional blood and CSF (if feasible) will be collected to complete correlative study analysis. Although GD2-CAR T cells have previously been administered in children, the product proposed here uses a unique costimulatory endodomain, and undergoes a slightly different manufacturing process and will be administered for the first time in subjects with pontine and spinal DMG. Hence a standard 3 + 3 dose escalation design will be used, enrolling 3 to 6 subjects sequentially using 3 dose levels of GD2CART (1e6 transduced T cells/kg (± 20%), 3e6 transduced T cells/kg (± 20%), and 10e6 transduced T cells/kg (± 20%)) to establish MTD/RP2D in children with DIPG and spinal DMG, although the safety of the arm escalating with DIPG can inform the dose escalation levels of the arm escalating with spinal DMG. Once the RP2D is established, additional subjects will be enrolled to two groups: children with H3K27M DIPG (up to 20 including subjects treated at MTD in dose escalation) or children and young adults with spinal H3K27M DMG (up to 10 including subjects treated at MTD in dose escalation), to further evaluate the safety and clinical activity of this regimen as outlined in Section 12.4.

Subjects who in the investigator's opinion would benefit and subjects who show improvement (clinically or radiographically) after infusion of the first intravenous dose of GD2CART may be eligible to receive a second infusion (see **Section 5.6**). The second dose may include intracerebroventricular administration or may be preceded by an intensified dose of lymphodepleting chemotherapy if given intravenouslyas outlined in **Section 5.6.**

2.10.7 Number of Subjects

Initially 3-6 evaluable subjects with H3K27M DIPG and spinal H3K27M DMG may be enrolled sequentially in 3 dose levels of GD2CART to establish MTD/RP2D, for a minimum of 4 subjects and a maximum of 18 per DMG disease type. Once RP2D is established, up to 20 evaluable subjects with H3K27M DIPG (including those from the dose escalation phase) and up to 10 subjects with spinal H3K27M DMG (including those from the dose escalation phase) will be treated at the RP2D dose to further assess safety and perform a preliminary analysis of clinical activity. Up to 6 subjects may be enrolled to replace subjects with feasibility issues, and up to 6 subjects may replace inevaluable subjects. Thus, 36 + 14 + 4 + 6 +6 yields a maximum of 66 subjects with H3K27M DIPG or spinal H3K27M DMG who may be enrolled to determine safety, feasibility, and preliminary efficacy of GD2CART therapy.

2.10.8 Study Duration

2.10.8.1 Primary Completion:

Up to 1-2 subjects will be accrued per month, and therefore this study may require up to 3 years to complete accrual. The study primary and secondary objectives will be completed in approximately 4 years.

2.10.8.2 Study Completion:

Subjects will be followed after treatment to evaluate toxicities, track disease progression, and to monitor for gene therapy effects. Short-term follow-up includes evaluations up until disease progression or subsequent alternative disease-directed therapy is initiated. In addition, long term follow up for gene therapy according to the U.S. Food and Drug Administration (FDA) ***Guidance for Industry: Gene Therapy Clinical Trials – Observing Participants for Delayed Adverse Events*** will be conducted on every infused subject for the required 15 years post infusion of gene-edited cells on this study or an alternative long-term follow up protocol.

3 PARTICIPANT SELECTION AND ENROLLMENT PROCEDURES

All subjects must sign and date the Institutional Review Board (IRB) and Administrative Panel on Biosafety (APB) approved consent form before initiating any study specific procedures or activities that are not part of a subject's routine care.

The Screening Participant Eligibility Checklist on the following page must be completed in its entirety for each subject prior to registration. The completed, signed, and dated checklist must be retained in the subject's study file and the study's Regulatory Binder or an electronic version completed within the subject's medical record.

The study coordinator, treating physician, and an independent reviewer must verify that the participant's eligibility is accurate, complete, and legible in source records, as required by the CCTO SOP 'Confirmation of Participant Eligibility in Clinical Trials'. A description of the eligibility verification process should be included in the EPIC or other Electronic Medical Record progress note.

The protocol-specific checklist is **required** by the SRC and must be approved by the IRB.

3.1 SCREENING PARTICIPANT ELIGIBILITY CHECKLIST

Protocol Title:	**Phase 1 Clinical Trial of Autologous GD2 Chimeric Antigen Receptor (CAR) T cells (GD2CART) for Diffuse Intrinsic Pontine Glioma (DIPG) and Spinal Diffuse Midline Glioma (DMG)**
Protocol Number:	**CCT6005 / IRB-52934**
Sponsor Investigator:	**Michelle Monje, M.D., Ph.D.**

II. Subject Information:

Subject Name/ID:
Gender: ☐ Male ☐ Female

III. Study Information:

SRC Approved ☐ IRB Approved ☐ Contract signed ☐

IV. Inclusion/Exclusion Criteria- STAGE 1

**Inclusion Criteria**<br>(From IRB approved protocol)	**Yes**	**No**	**Supporting Documentation***
1. Disease Status<br>• Diagnosis of H3K27M mutated Diffuse Intrinsic Pontine Glioma (DIPG), OR<br>• Diagnosis of spinal H3K27M mutated diffuse midline glioma (DMG)	☐	☐	or NA ☐
2. Age:<br>Greater than or equal to 2 year of age and less than or equal to 30 years of age.	☐	☐	
3. Prior Therapy:<br>• At least 6 weeks following completion of standard upfront radiation therapy.	☐	☐	
• At least 3 weeks post chemotherapy or 5 half-lives, whichever is shorter, must have elapsed since any prior systemic therapy, except for systemic inhibitory/stimulatory immune checkpoint therapy that requires 5 half-lives.	☐	☐	
4. Performance Status:<br>Subjects > 16 years of age: Karnofsky ≥ 60% OR Eastern Cooperative Oncology Group (ECOG) performance status of 0 or 1; Subjects ≤ 16 years of age: Lansky scale ≥ 60% (See section 13.1, Appendix A). Subjects who are unable to walk because of paralysis, but who are up in a wheelchair, will be considered ambulatory for the purpose of assessing the performance score.	☐	☐	

5. Normal Organ and Marrow Function (supportive care is allowed per institutional standards, i.e. filgrastim, transfusion)<br>i. ANC ≥ 1000/uL<br>ii. Platelet count ≥ 100,000/uL<br>iii. Absolute lymphocyte count ≥ 150/uL<br>iv. Hemoglobin ≥ 8 g/dL<br>v. Adequate renal, hepatic, pulmonary and cardiac function defined as:	ANC<br>Platelet<br>ALC<br>Hgb		
• Creatinine within institutional norms for age (i.e. ≤ 2 mg/dL in adults or according to table below in children <18 years) OR creatinine clearance (as estimated by Cockcroft Gault Equation) ≥ 60 mL/min	Creatinine	☐	

Age (Years)	Maximum Serum Creatinine (mg/dL)
≤5	0.8
5 < age ≤ 10	1.0
>10-18	1.2
>18	2.0

• Serum ALT/AST ≤ 3.0 ULN (grade 1)	AST/ALT	☐	
• Total bilirubin ≤ 1.5 mg/dl, except in subjects with Gilbert's syndrome.	Bilirubin	☐	
• Cardiac ejection fraction ≥ 45%, no evidence of physiologically significant pericardial effusion as determined by an ECHO, and no clinically significant ECG findings	LVEF	☐	
• Baseline oxygen saturation > 92% on room air	O2 Sat	☐	
6. Pregnancy Test<br>Females of childbearing potential must have a negative serum or urine pregnancy test (females who have undergone surgical sterilization are not considered to be of childbearing potential)	☐	☐	or NA ☐
7. Contraception<br>Subjects of child-bearing or child-fathering potential must be willing to practice birth control from the time of enrollment on this study and for four (4) months after receiving the preparative regimen or for as long as GD2CART cells are detectable in peripheral blood or CSF.	☐	☐	or NA ☐
8. Ability to give informed consent.<br>All subjects ≥ 18 years of age must be able to give informed consent. For subjects <18 years old their legal authorized representative (LAR) (i.e. parent or guardian) must give informed consent. Pediatric subjects will be included in age appropriate discussion and written assent will be obtained for those > 7 years of age, when appropriate. If a minor becomes of age	☐	☐	

during participation of this study, he/she will be asked to reconsent as an adult.			

**Exclusion Criteria**	**Yes**	**No**	**Supporting Documentation***
1) Tumor involvement of cerebellar vermis or hemispheres (pontocerebellar peduncles involvement is acceptable), or thalamic lesions.	☐	☐	
2) Clinically significant swallowing dysfunction or prominent dysphagia, as determined by the clinical investigator.	☐	☐	
3) Current systemic corticosteroid therapy.	☐	☐	
4) Prior CAR therapy.	☐	☐	
5) Uncontrolled fungal, bacterial, viral, or other infection. Previously diagnosed infection for which the patient continues to receive antimicrobial therapy is permitted if responding to treatment and clinically stable.	☐	☐	
6) Ongoing infection with:<br>• HIV,<br>• Hepatitis B (HBsAg positive) or<br>• Hepatitis C virus (anti-HCV positive).<br>A history of hepatitis B or hepatitis C is permitted if the viral load is undetectable per quantitative PCR and/or nucleic acid testing.	☐	☐	
7) Clinically significant systemic illness or medical condition (e.g. significant cardiac, pulmonary, hepatic or other organ dysfunction), that in the judgement of the principal investigator is likely to interfere with assessment of safety or efficacy of the investigational regimen and its requirements.	☐	☐	
8) Women who are pregnant or breastfeeding.	☐	☐	or NA ☐
9) In the investigator's judgment, the subject is unlikely to complete all protocol-required study visits or procedures, including follow-up visits, or comply with the study requirements for participation.	☐	☐	
10) Known sensitivity or allergy to any agents/reagents used in this study.	☐	☐	
11) Primary immunodeficiency or history of autoimmune disease (e.g. Crohns, rheumatoid arthritis, systemic lupus) requiring systemic immunosuppression/systemic disease modifying agents within the last 2 years	☐	☐	

*All subject files must include supporting documentation to confirm subject eligibility. The method of confirmation can include, but is not limited to, laboratory test results, radiology test results, subject self-report, and medical record review.

IV. Statement of Eligibility

By signing this eligibility form, I verify that this subject is [☐ **eligible** / ☐ **ineligible**] for participation in this clinical trial. This study is approved by the Stanford Cancer Institute Scientific Review Committee, the Stanford IRB, and has finalized financial and contractual agreements as required by Stanford School of Medicine's Research Management Group.

Treating Physician Signature:	Date:
Printed Name:	
Secondary Reviewer Signature:	Date:
Printed Name:	
Study Coordinator Signature:	Date:
Printed Name:	

3.2 INFORMED CONSENT PROCESS

All participants must be provided a consent form describing the study with sufficient information for participants to make an informed decision regarding their participation. The investigational nature and research objectives of this trial, the procedures and treatments involved and their attendant risks and discomforts and potential benefits, and alternative therapies will be carefully explained to the subject, and asked to review it and to ask questions prior to agreeing to participate in this protocol. The subject is reassured that participation on trial is entirely voluntary and that he/she can withdraw or decide against treatment at any time without adverse consequences.

Pediatric subjects will be included in age appropriate discussion. Written assent will be obtained for those ≥ 7 years of age when deemed appropriate by the clinician and the child's parents or legally authorized representative(s). Should a minor subject reach the age of majority during participation in this trial (active therapy or follow up) they will be asked to complete the informed consent as an adult.

The original signed copy of the consent document must be retained in the research file and a copy placed in the medical record.

3.3 SUBJECT SCREENING ASSESSMENTS AND REGISTRATION

3.3.1 General considerations for Subject Screening

The screening period begins on the date the subject and/or the subject's LAR signs the IRB/IEC approved ICF and continues through confirmation of enrollment (the date triple review eligibility sign off is completed to verify that the participant's eligibility is accurate, complete, and legible in source records). Informed consent must be obtained before completion of any non-standard of care study specific procedures. Procedures that are part of standard of care are not considered study specific procedures and may be performed prior to obtaining consent and used to confirm eligibility.

After written informed consent has been obtained, subjects will be screened to confirm study eligibility and participation. Only subjects who meet the eligibility criteria listed in sections **3.1** will be enrolled in the study. If at any time prior to enrollment the subject fails to meet the eligibility criteria, the subject should be designated as a screen failure on the subject screening log with the reasons for failing screening.

All subjects will undergo the screening procedures, which includes a comprehensive history and physical exam performed by a study physician or nurse practitioner. Imaging and organ specific studies will be performed as per institutional guidelines. Confirmation of this data must occur within 28 days of enrollment, unless specified otherwise.

3.3.2 Study Enrollment

Before enrollment of a subject into the study, the responsible physician must ensure the subject meets all eligibility criteria using the Study Screening Procedures outlined in section **5.1**. Eligibility criteria will be reviewed and confirmed by the Principal Investigator or designee prior to any subject being enrolled into the study (section **3.1**).

Enrollment will be defined as the date triple review eligibility sign off is completed to verify that the participant's eligibility is accurate, complete, and legible in source records. At time of enrollment, each subject will receive a unique subject identification number. This number will be used to identify the subject throughout the study and must be used on all study documentation related to the subject. Furthermore, the subject identification number must remain constant throughout the entire clinical study, it must not be changed after enrollment or if the subject is rescreened or retreated.

4 TREATMENT PLAN

4.1 OVERVIEW

This is a single site, open label Phase I study in subjects with H3K27M pontine diffuse midline glioma DIPG and spinal H3K27M DMG who have undergone front-line radiation therapy. In light of the previous clinical experience in which GD2-CAR T cells were administered safely in children and adult subjects with osteosarcoma and neuroblastoma, and because DMG occurs essentially exclusively in children <12 years, we plan enrollment of pediatric subjects during the dose escalation phase. Because GD2CART have not been previously administered in subjects with DMG and because preclinical models demonstrate that a significant toxicity risk related to disease within the pons in patients with pontine DMG which may swell during effective therapy, we will conduct a standard 3 + 3 dose escalation design to establish MTD/RP2D using 3 dose levels (1e6 transduced T cells/kg (± 20%), 3e6 transduced T cells/kg (± 20%), and 10e6 transduced T cells/kg (± 20%)) in the cohort of subjects with DIPG. While spinal cord DMG confers a distinct set of possible toxicities associated with CART cell therapy, such as spinal inflammation causing spinal cord dysfunction, we do not anticipate the potentially life-threatening intracranial complications that could occur with DIPG. Hence a separate dose escalation cohort will be performed in subjects with spinal H3K27M DMG but does escalation decisions in this cohort will be informed by demonstration of safety for a given dose in the DIPG arm. In order to avoid unnecessary exposure of subjects to subtherapeutic doses while preserving safety and maintaining rapid escalation to therapeutic dose ranges, we proposed to allow the safety data collected during dose escalation of subjects in the pontine DIPG arm to inform the dose escalation of subjects in the spinal DMG arm.

Once the RP2D is defined in each arm, we will expand the cohorts to assess efficacy in a total of 20 evaluable subjectsin the DIPG arm and a total of 10 evaluable subjects in the spinal DMG arm treated at RP2D, and further determine safety in these two DMG arms.

Subjects are eligible for enrollment when they are at least 6 weeks from completion of standard upfront radiotherapy, regardless of documented evidence of progression, and if all other eligibility criteria are met. This eligibility criterion was chosen based of the following considerations:

1) A requirement for documented progression prior to enrollment is not feasible, since radiographic progression cannot be reliably distinguished from radionecrosis in all patients.

2) Post-progression survival is very short (median 2.3 months) and may not be long enough for patients to benefit from the effects of the GD2CART.

3) Median progression free survival following radiotherapy is 7.0 months (80.8% demonstrating progression within 12 months), therefore the risk of progression beyond 3 months is sufficiently high to justify the risks and morbidity associated with the investigational treatment regimen.

4) Preclinical models of DIPG demonstrate that bulky disease is a risk factor for treatment related morbidity and mortality due to hydrocephalus, therefore enrolling patients prior to documented clinical or radiographic progression will increase the likelihood that the therapy can be rendered safely.

An Ommaya catheter will be inserted after enrollment and prior to GD2CART infusion in subjects with DIPG. Non-mobilized autologous PBMC will be obtained by leukapheresis in all subjects and transduced with GD2.BB.z.iCasp9 retroviral vector. Cryopreserved PBMC stored from participation in other institutional cell therapy or cell collection studies may be used to generate the cellular product on this study as long as they meet the criteria established in this IND. Subjects will receive a lymphodepleting chemotherapy preparative regimen with fludarabine and cyclophosphamide, followed by infusion of GD2CART at Stanford Lucile Packard Children's Hospital (LPCH). The study will evaluate safety of administration, feasibility of manufacturing, identify the recommended phase 2 dose (RP2D) and conduct a preliminary assessment of clinical activity in subjects with DIPG by evaluating overall survival (OS) compared to historical controls and in subjects with DMG by describing overall survival (OS). In addition, radiographic response, progression free survival (PFS) will be evaluated in all subjects . The CAR vector will incorporate an inducible Caspase 9 that can lead to efficient T cell apoptosis following exposure to AP1903 should toxicity require inactivation of the cell product.

The feasibility of generating GD2CART using a retroviral vector in the Miltenyi CliniMACS Prodigy® 'all-in-one' cell processing system will be evaluated as a primary objective. If feasibility of cell production is not met (i.e. 3 of the first 6 subjects' cells cannot be produced to meet the established release criteria) further enrollment will be paused pending evaluation of the manufacturing process, and modifications made appropriate to improving feasibility prior to continuing enrollment. This may require a protocol or IND amendment. In any event, if 3 of the first 6 cell products manufactured do not meet established criteria, it will be reported to the IRB.

While this CAR has been administered to subjects with neuroblastoma and osteosarcoma, it has not been previously administered to subjects with DMG, which offers new challenges to management of potential CAR therapy-related toxicities. To mitigate risk in this patient population, subjects will be carefully selected to avoid large disease burden, and subjects with DIPG will have an intraventricular catheter (Ommaya catheter) placed prior to cell infusion for monitoring and if necessary, rapid and efficient treatment of increased intracranial pressure. Increased intracranial pressure will be treated by neurologists, intensivists and neurosurgeons who are familiar with the trial using the guidelines shown in section **13.2.4**. Subjects with spinal DMG will be closely monitored using a standardized spinal cord assessment tool (section **13.2.5**) and will also be treated by neurologists, intensivists and neurosurgeons using guidelines (section **13.2.5**).

4.1.1 Determination of Maximum Tolerated Dose (MTD)/Recommended Phase 2 Dose (RP2D)

Initially the MTD/RP2D will be determined in the pediatric population of subjects with pontine DIPG and spinal DMG by treating 3 to 6 subjects sequentially in 3 dose finding cohorts of GD2CART (1e6 transduced T cells/kg (± 20%), 3e6 transduced T cells/kg (± 20%), and 10e6 transduced T cells/kg (± 20%)). The DLT assessment period is defined as 28 days. A 28 day safety assessment will follow infusion of GD2CART in the first subject in each dose cohort prior to cell infusion of the second subject. Fourteen (14) day safety assessment will follow infusion of GD2CART of subsequent subjects in each dose cohort. Four weeks (28 days) must elapse after completion of cell infusion in the final subject in each dose cohort to allow for safety assessment before treating subjects at the next higher dose cohort. Therefore, infusion at the next higher dose will not proceed until the last subject infused on the completed dose cohort has been observed for at least 28-days after infusion of GD2CART. Because we do not anticipate the potentially life threatening intracranial complications of pontine DIPG in the subjects with spinal DMG, we propose to allow the safety of GD2CART in the pontine DIPG arm to inform the safety of dose escalation in spinal cord DMG arm. Spinal cord DMG is rarer than pontine DIPG, so the spinal cord arm is expected to accrue more slowly and we anticipate fewer participants and insufficient accrual to efficiently complete accrual to each of three dose levels during dose escalation of the spinal DMG arm. Hence, if the first subject with pontine DIPG receives dose level 1 of GD2CART and 28 days elapses without DLT, subsequent subjects with spinal DMG may receive GD2CART if 14 days elapse between subject infusions without DLT. Similarly, if 3 subjects with pontine DIPG complete 28 days of monitoring without DLT at dose level 1, the next subject with spinal DMG may enroll to dose level 2. The reverse of this rule will not apply to subjects with pontine DIPG given the unique nature of their potential risk for on-target, on-tumor effects in the pons.

If more than one (1) DLT occurs in the first dose level, (2 out of 6) the dose will be de-escalated to Dose level -1 (3e5 transduced T cells/kg (± 20%)) for safety evaluation. If ≥ 2 DLTs occur at dose level -1, the study will be suspended pending discussion with the FDA, IRB, APB to improve safety. Otherwise, dose escalation will proceed as outlined in section **5.5.4.**

4.1.2 Dose Expansion Cohorts

Once the MTD/RP2D is established (MTD is defined as the dose level below that in which 2/6 subjects experience DLTs), additional subjects will be enrolled to further evaluate the safety and conduct a preliminary evaluation of clinical benefit of this regimen in two cohorts:

1. Subjects with H3K27M DIPG (n=20 total)
2. Subjects with spinal H3K27M DMG (n=10 total).

The trial will continue to evaluate safety in the dose-expansion cohort. Safety boundaries will be used to monitor a DLT rate of 30%. A dose-expansion cohort of subjects with H3K27M DIPG will enroll up to 20 evaluable subjects to monitor safety and indicate efficacy, while the spinal H3K27M DMG will enroll up to 10 subjects at RP2D. The trial will evaluate safety profile after the first 4 and 9 subjects are evaluable in the dose-expansion cohort, corresponding to a total of 10 and 15 evaluable subjects treated at the MTD/RP2D with 6 subjects from the dose escalation cohort.

4.2 : General Concomitant Medication and Supportive Care Guidelines

4.2.1 Infection Prophylaxis

Any temperature of >38° C will require initiation of the fever work-up and treatment according to institutional standards.

4.2.1.1 Viral Prophylaxis

All subjects will be treated as high risk and will receive viral prophylaxis according to institutional standards and investigator clinical judgement.

4.2.1.2 Fungal Prophylaxis

All subjects will be treated as high risk and will receive fungal prophylaxis according to institutional standards and investigator clinical judgement.

4.2.2 Blood Product Support for Anemia and Thrombocytopenia

Using CBC's as a guide, the subject will receive platelets and packed red blood cells (PRBC's) as needed. Attempts will be made to keep Hb > 8.0 gm/dl, and plts > 50,000/mm3. All blood products with the exception of the lymphocyte product will be irradiated. Leukocyte filters will be utilized for all blood and platelet transfusions to decrease sensitization to transfused WBC's and decrease the risk of CMV infection.

In subjects with coagulopathy, attempts will be made to keep fibrinogen ≥ the lower limit of normal.

4.2.3 Cytokine Release Syndrome

Cytokine release syndrome (CRS), a toxicity associated with infusion of CAR T cell therapy, has been described in section 2.6.4. Grading and management of CRS in this protocol will follow the guidelines in section 13.2, Appendix B[29],[52] which includes diligent supportive care and evaluations for infection, with immunosuppression using anti-IL6R mAbs and/or corticosteroids reserved for more severe cases. Use of anakinra, an IL-1 receptor antagonist, currently approved by the US Food and Drug Administration for the treatment of patients with rheumatoid arthritis and neonatal-onset multisystem inflammatory disease, has been used off label for the treatment of secondary hemophagocytic lymphohistiocytosis (HLH), a condition in the spectrum of CRS potentially associated with chimeric antigen receptor (CAR) T-cell therapy[65],[66]. Anakinra may be considered for management of CRS related toxicities, at the investigator's discretion. Because understanding of the constellation of symptoms defining CRS is evolving, AE CRFs will capture both the syndrome and the individual symptomatology of CRS. Investigators should follow the protocol's CRS management guidelines whenever possible, but deviation from the guidance will not be considered a protocol deviation as all toxicity management should be at the treating physician's discretion.

4.2.4 Neurotoxicity

Neurotoxicity (e.g., encephalopathy, somnolence, aphasia) has been observed with CAR T cell therapies (immune effector cell therapies) and will be scored using the tools available in section

13.2, Appendix B, using a combination of the ASTCT Immune effector Cell-Associated Neurotoxicity Syndrome (ICANS) Consensus Grading for Adults with the Immune effector Cell-associated Encephalopathy (ICE) assessment tool; or ASTCT Immune effector Cell-Associated Neurotoxicity Syndrome (ICANS) Consensus Grading for Children with either the ICE assessment tool or the Cornell Assessment of Pediatric Delirium (CAPD)[52].

Administration of GD2CART will occur after placement of an Ommaya catheter in subjects with DIPG to monitor intracranial pressure. Subjects with DMG at higher risk of increased intracranial pressure may also have an Ommaya catheter, at investigator discretion. Neurooncologists, neurosurgeons and neurointensivists will be consulted and involved in every case for management of neurologic deterioration and/or subclinical increase in intracranial pressure. If patients develop uncontrolled toxicities, they will be given AP1903 to ablate the CAR T cell product.

All subjects will receive levetiracetam (Keppra) beginning the day before cell infusion. For good clinical practice, clinicians and staff will evaluate the ICE or CAPD score or standard neuro checks every 8 hours (± 2 hours) or as clinically indicated. Neurologic evaluations will be assessed with the scheduled clinical evaluation daily.

Evaluation of any new onset of neurotoxicity should consider recommended interventions in Appendix B section 13.2.3. These recommendations should serve as guidance for toxicity management, but deviation from the guidance will not be considered a protocol deviation, as all toxicity therapy should be at the treating physician’s discretion. If increased ICP is suspected, recommendations for management consideration are provided in section 13.2.4. Spinal cord function in patients with spinal DMG should be conducted with neurologic exams according to American Spinal Injury Association (ASIA) International standards for neurological classification of spinal cord injury ASIA Impairment Scale (https://asia-spinalinjury.org/wp-content/uploads/2016/02/International_Stds_Diagram_Worksheet.pdf) and will follow the recommended interventions outlined in section 13.2.5.

Medications with sedative properties should be avoided if possible unless required to manage seizures, i.e. benzodiazepines. Subjects and their families/caregivers should be warned of the risk of late neurotoxicity thru day 28 and told to seek immediate medical attention for any new symptoms of neurotoxicity.

4.2.5 Unacceptable Toxicity

GD2CART incorporates an inducible Caspase 9 gene (iCasp9) that can act as a suicide safety switch in the event of unacceptable toxicity, defined as life-threatening/grade 4 toxicity believed by the investigators to cause substantial risk to the subject, which is possibly, probably or definitely related to the cellular therapy. In the presence of the small molecule AP1903, the iCasp9 promolecule dimerizes and activates the intrinsic apoptotic pathway, leading to cell death. Should subjects encounter unacceptable toxicity that cannot be safely managed with supportive care as described within this protocol, AP1903 may be administered to rapidly deplete GD2CART levels (see section 6.9 for more information on AP1903).

4.3 Criteria for Removal from Protocol Therapy and Off Study Criteria

4.3.1 Criteria for removal prior to GD2CART cell infusion

Subjects will be taken off treatment and followed until effects of leukapheresis or chemotherapy have reversed and all toxicities are resolved to Grade 1 or baseline for any of the following:

- ✓ General or specific changes in the subject's condition render the subject unacceptable for cell infusion on this study in the judgment of the investigator.
- ✓ Pregnancy in a female of child-bearing potential.
- ✓ Cells do not meet infusion release criteria (criteria other than targeted dose).

Once toxicities and effects of leukapheresis or chemotherapy resolve, subjects who are unable to undergo cell infusion will be removed from this study.

4.3.2 Criteria for removal from the option for a 2nd cell infusion:

Subjects will not be eligible for further therapy (2nd cell infusion as outlined in section 5.6) (and will be followed until off-study criteria are met) for the following:

- ✓ Dose limiting toxicity (DLT) after first infusion. The definition of DLT is in section 5.4.5, unless approved by the FDA and IRB in advance of the second infusion.
- ✓ Pregnancy in a female of child-bearing potential.
- ✓ No cells available for re-treatment.

4.3.3 Off-Study Criteria

- ✓ Subject withdrawal of consent (in which case the reason will be documented, if possible). Subjects who withdraw consent for additional procedures will be requested to participate in long-term follow up.
- ✓ Subject who meet any of the withdrawal criteria listed in Section 4.3.1 and who have recovered from all study-induced toxicity.
- ✓ Subject lost to follow-up. Should a subject fail to return to the clinic for a scheduled protocol specific visit, site will need to make 2 attempts by a combination of telephone and mail to contact the subject. Site must document both attempts to contact the subject. If a subject does not respond within 1 month after the second contact the subject will be considered lost to follow-up and no additional contact will be required.
- ✓ Death
- ✓ Conclusion of the 15 years of follow up, or subject enrolls in a separate long-term follow up protocol for subjects receiving gene transfer.

4.3.4 Off-Study Procedure

Off study date and reason should be documented in the study CRFs. For subjects withdrawing consent, the investigator should inquire whether the subject agrees to allow chart review of normal medical care procedures and/or long term follow-up of gene therapy research participants. Cell products and biological samples from off-study subjects may continue to be used for process development and correlative studies, as outlined in the consent form.

5 SUBJECT STUDY PROCEDURES

All subjects will undergo the screening procedure, which includes a comprehensive history and physical exam performed by a study physician as outlined below. Imaging studies will be performed as per institutional guidelines. The following screening tests must be performed within 28 days prior to enrollment unless specified otherwise.

5.1 SUBJECT SCREENING

The screening period begins on the date the subject/LAR signs the IRB and APB approved consent form and continues through confirmation of enrollment. Procedures that are to be performed as part of the practice of medicine and which would be done whether or not study entry was contemplated, such as for diagnosis or treatment of a disease or medical condition, may be performed and the results subsequently used for determining study eligibility without first obtaining consent. Informed consent must be obtained prior to initiation of any clinical screening procedures that are performed solely for the purpose of determining eligibility for research, i.e. withdrawal from medication (wash-out period). Only subjects who meet the eligibility criteria listed in section **3** will be enrolled in the study.

Screening will proceed as outlined in section 3.3; the following screening evaluations must be completed **within 28 days of enrollment unless otherwise specified**.

(a) Medical history

The subject's complete history through review of medical records and by interview will be collected and recorded. Concurrent medical signs and symptoms must be documented to establish baseline severities. A disease history, including the date of initial diagnosis, verification of diagnosis of H3K27M DIPG or spinal DMG (H3K27M mutation may be determined at any point since diagnosis), and prior radiotherapy, or other disease directed therapies and the disease response, and duration of response to the radiotherapy or prior treatment also will be recorded.

(b) Physical examination

A complete physical examination will be performed. The exam will include general appearance of the subject, height and weight, examination of the skin, eyes and ears, nose, throat, lungs, heart, abdomen, extremities, musculoskeletal system, and a thorough nervous system evaluation.

(c) Vital signs, including blood pressure, heart rate, oxygen saturation and temperature will be recorded.

(d) Performance status (ECOG or Lansky or Karnofsky) see Appendix A, Section **13.1.**

(e) Electrocardiogram (ECG)

(f) Evaluation for HIV seropositivity to consist of ELISA and, if positive, confirmation by Western blot within the time requirements for autologous apheresis donation for standard of care collections, or within 28 days prior to the leukapheresis procedure for procedures performed on this study. The investigator, in the event of a positive finding, will make appropriate counseling available.

(g) Evaluation for Hepatitis B core antibody (HBcAb), Hepatitis B surface antibody (HBsAb), Hepatitis B surface antigen (HBsAg) and Hepatitis C Virus (HCV) (anti-HCV Antibody) within the time requirements for autologous apheresis donation for standard of care collections, or within 28 days prior to leukapheresis procedure for procedures performed on this study.

(h) β-HCG pregnancy test on all women of child-bearing potential (within 28 days of enrollment)

(i) General Laboratory Tests: The following will be obtained during the screening process:

- ✓ Chemistries: (sodium, potassium, chloride, bicarbonate, BUN, creatinine, glucose, calcium, AST/ ALT, alkaline phosphatase, bilirubin, albumin, total protein) **(**A measured 24 hour urine creatinine clearance test may be performed if the serum creatinine is elevated, and the measured value will be recorded in the CRF and may be used to qualify the subject for study participation)
- ✓ Phosphorus and magnesium
- ✓ CBC with differential
- ✓ C-reactive protein (CRP), ferritin
- ✓ Urinalysis

(j) ECHO, MUGA or Cardiac MRI for LVEF and pericardial effusion assessment

Testing for cardiac performance and ejection fraction to confirm eligibility may be performed anytime within 180 days prior to enrollment.

(k) Disease Evaluation: Disease evaluations will be specific to the subject's location of disease and may include imaging studies: Brain MRI, MRI with and without gadolinium, CT or PET/CT. Disease evaluation of subjects with DIPG will include Standard MR imaging with Sagittal T1 MPRAGE, axial DWI, axial T2 FLAIR, axial T2, and post gadolinium sagittal T1 MPRAGE (with reconstructions) images. The standard MR parameters are listed on the

PBTC NIC web page located at http://www.childrenshospital.org/research/centers-departmental-programs/pediatric-brain-tumor-consortium-neuroimaging-center under Neuroimaging Studies/ Specific MR Imaging Sequences- Open PBTC Protocols.

In addition to radiologic evaluation, subjects will undergo Clinical Evaluation of Neurologic Status to serve as a baseline for determination of clinical benefit (see **Section 13.8, Appendix H: Clinical Evaluation of Neurologic Status**).

(l) Concomitant Medication

A list of concomitant medications will be captured at the end of screening at the time of confirmation of eligibility. Medications that are stopped during the screening period prior to eligibility sign off do not need to be recorded. The concomitant medication list will be updated from data collected at each clinic visit.

(m) Additional Tests:

The PI may order additional tests in some subjects if needed to fully assess clinical status and obtain baseline results.

5.2 Leukapheresis for Cell Acquisition:

For subjects who do not have a leukapheresis product stored from a previous procedure that meets requirements for utilization on this study, following enrollment, they will undergo leukapheresis for cell collection. Leukapheresis (apheresis) will be performed according to institutional standards with the goal of obtaining adequate cells to generate the planned cell doses of transduced T cells/kg. Prophylactic intravenous $CaCl_2$ and $MgSO_4$ infusions may be administered by the apheresis clinical team per standard operating procedures. Institutional guidelines will be followed for venous access and apheresis procedures. If PBMCs have been cryopreserved for a different cell therapy or cell collection study, or standard of care, and meet the requirements for this study, they may be used to generate GD2CART on this study if they meet criteria outlined in the IND.

The patient may have a second apheresis collection to obtain the CD3+ cell target and the required minimum number of cells, at the investigator's discretion. If the patient cannot be scheduled for a subsequent apheresis collection and is unable to meet the required number of cells, the patient will be discontinued from the study. If, after enrollment and leukapheresis, the decision is made to not proceed with GD2CART cell manufacturing, the collected apheresis product will be made available to the subject for alternative CAR-T cell production, if requested.

The criteria for initiating leukapheresis is as follows:

- ✓ Subjects must have no evidence of a clinically significant uncontrolled infection prior to leukapheresis.
- ✓ No systemic cortico steroid therapy within 2 weeks prior to leukapheresis.
- ✓ No new signs or symptoms of kidney or liver dysfunction outside eligibility criteria within last 7 days
- ✓ Must not be pregnant

Any changes to eligibility criteria after enrollment will not affect eligibility/enrollment on this study, unless in the physician's estimation they affect the safety of the subject moving forward with the investigational regimen.

The following procedures/requirements will occur on the leukapheresis collection day (+2 days) (unless otherwise specified) and as outlined in the section **9** Study Calendar:

- Vital signs, including blood pressure, heart rate, respiratory rate, oxygen saturation, and temperature
- Weight (day of leukapheresis or day before) including body mass index (BMI) calculation. If BMI > 30, manufactured cell dose will be based on practical weight rather than actual weight (see section **13.3**, Appendix C).
- Labs (to be drawn within 7 days prior to leukapheresis)
    - Chemistry panel (sodium, potassium, chloride, bicarbonate, BUN, creatinine, glucose, calcium, AST and/or ALT, alkaline phosphatase, bilirubin, albumin, total protein)
    - Phosphorus and magnesium
    - CBC with differential
    - C-reactive protein (CRP)
    - Pregnancy test (in child-bearing females, if screening pregnancy test was performed > 7 days prior to leukapheresis)
- Leukapheresis
- Adverse/Serious Adverse Event reporting related to the leukapheresis procedure
- Concomitant medications documentation
- Correlative studies sample collection, as detailed in Appendix G, section **13.7**

5.3 Conditioning Lymphodepletion Chemotherapy Regimen

Subjects will receive a conditioning lymphodepletion chemotherapy regimen consisting of cyclophosphamide and fludarabine in order to induce lymphocyte depletion and create an optimal environment for expansion of GD2CART *in vivo*. Subjects will initiate conditioning chemotherapy with cyclophosphamide and fludarabine beginning on Day -4 through Day -2. The 3-day conditioning chemotherapy regimen may be administered in an outpatient setting per investigator's discretion. The dose calculation for the chemotherapy administration (on Days -4 through -2) will be based on the height and weight measured on day -4 to -6 (± 5 days). At the investigator's discretion, the timing of the 3-day regimen of conditioning lymphodepletion chemotherapy may be adjusted based on the best interest of the patient.

In order to initiate the conditioning lymphodepletion regimen, there must be 14 days or more than five half-lives from the last dose of standard chemotherapy and at least 2 weeks since the last dose of corticosteroids. The subject must meet the criteria established in Section **5.3.1.**

5.3.1 Eligibility criteria for initiating conditioning lymphodepletion chemotherapy regimen

Routine anti-emetic prophylaxis and treatment should be employed. The criteria for initiating the conditioning regimen is as follows:

Subjects must have:

- no evidence of uncontrolled infection,
- no clinically significant cardiac dysfunction,
- serum creatinine must be < 2 x ULN,
- no change in neurologic status that in the investigator's assessment puts the patient at unacceptable risk of toxicity or is a significant indication of disease progression,
- negative pregnancy test in child-bearing females.
- no systemic corticosteroids for 14 days prior to chemotherapy

Should an event exceed these criteria immediately prior to conditioning chemotherapy, conditioning chemotherapy must be delayed until the event resolves to ≤ Grade 1 or baseline.

5.3.2 Conditioning Lymphodepletion Chemotherapy Regimen Procedures

The procedures for Day -5 to Day -2 are noted in the Study Calendar (see section **9**). Females of childbearing potential must have a negative pregnancy test within 7 days prior to starting chemotherapy.

5.3.2.1 Fludarabine and Cyclosphosphamide Administration

Each subject will receive the lymphodepleting regimen as follows:

Table 1: Conditioning Lymphodepletion Chemotherapy Regimen

**Drug**	**Dose**	**Days**
Cyclophosphamide	500 mg/m^2 per day IV infusion over approximately 60 minutes, daily for 3 days.	-4, -3, -2
Fludarabine	After conclusion of cyclophosphamide infusion, 25 mg/m^2 per day IV infusion in 50 mL of 0.9% sodium chloride over approximately 30 minutes, daily for 3 days.	-4, -3, -2

There will be no dose adjustment of chemotherapy agents for weight considerations or abnormal lab values. If subjects are eligible for the trial, then the full dose of lymphodepletion agents will be administered.

5.3.2.1.1 Supportive Care and Premedications for Lymphodepletion Chemotherapy

- Fluid and electrolyte balance will be maintained as per institutional guidelines and may include as guidance (modifications from these guidelines will be based on investigator's best clinical judgement and will not constitute a protocol deviation): Hydration may be initiated 2 hours prior to cyclophosphamide using NS (5% Dextrose and 0.9% Sodium Chloride Injection, USP solution) or another isotonic fluid. Oral and intravenous fluids should meet at a minimum 2/3 of If able to tolerate oral fluids, subject may be discharged after 2 hours of hydration. When discharged to home, include patient instructions: patient to drink 4 ounces/m^2/2hr = xx ounces) every 2 hours and void every 2 hours until bedtime. If unable to tolerate oral fluids, continue IV hydration until 4 hours post-cyclophosphamide.

- Mesna may be administered at a dose of 300 mg/m^2 per day by continuous IV infusion at the investigator's discretion. If administered, the dose of mesna will be divided each day, with half the dose mixed with cyclophosphamide in an appropriate amount of fluid based on the hydration rate, and half the dose infused afterwards, as per institutional standard practice.
- Furosemide: During conditioning lymphodepletion chemotherapy regimen, IV furosemide (0.5 - 1 mg/kg/dose to a maximum of 20 mg per dose) may be administered as needed to maintain normal urine output and fluid balance. Hydration will be adjusted to maintain 2/3 fluid maintenance, urine output and urine specific gravity. In addition, serum electrolyte levels will be monitored routinely and IV fluid content including potassium chloride supplementation will be adjusted to maintain normal serum electrolyte levels.
- Anti-emetics

  Routine anti-emetic prophylaxis and treatment should be employed. Corticosteroids may not be used.

  5.3.2.1.2 Correlative Samples

  Collection of blood, tissue, or cerebrospinal fluid samples for correlative research will occur as outlined in section 13.7 Appendix G: Correlative Sample Schedule. Samples may be omitted if limited by institutional research blood draw volume limits or in the best interest of the subject.

5.4 Investigational Agent Administration and Research Procedures

5.4.1 Cell Processing:

Cellular Product: GD2.BB.z.iCasp9-chimeric antigen receptor (GD2-CAR) retroviral transduced autologous peripheral blood lymphocytes (called GD2CART)

Autologous GD2CART will be generated from fresh or cryopreserved PBMCs under GMP conditions using Miltenyi CliniMACS Prodigy® system. Apheresis products from participating subjects will be sent to the manufacturing site through Stanford Bone Marrow Transplant – Cellular Therapeutics Facility (BMT-CTF); final cell products will be received through and final formulated product distributed by the BMT-CTF.

Any prepared cells not required for the first infusion or for research or regulatory purposes (including sufficient QA retention vials for testing cryopreserved product stability at time of additional infusions) will be cryopreserved by standard techniques and will be made available should the subject be eligible for a second infusion as outlined in section **5.6**. Additional product in excess of that needed for infusion may be utilized for research.

Fresh or cryopreserved peripheral blood mononuclear cells (PBMC) (depending on the timing of apheresis relative to cell culture, subject condition and scheduling availability) will be used for cell manufacturing. Final product will be required to meet standard release criteria as detailed below. All procedures will take place using good manufacturing process (GCP) guidelines.

The release criteria will be based upon analyses of GD2CART and will include:

Table 2: GD2CART Product Rapid Release Criteria

Test	Criteria
Cell viability	≥ 70%
Cell number	within 20% of planned dose level
% CAR+ cells	≥ 10%
Endotoxin	≤ 5 EU/kg
Mycoplasma	≤ 10 CFU/mL
RCR	Negative
Sterility testing	Negative

In the event that a product does not meet one or more of the release criteria but infusion is deemed to be in the best interest of the subject, the investigator, with approval of FDA, IRB and APB and consent of the subject/LAR, may elect to infuse the product, with the exception of dose which will be handled according to section **12.2.1** and does not require additional approvals.

5.4.1.1 Determination of product administration: fresh or cryopreserved

GD2CART will be administered on Day 0. Cells may be cryopreserved after harvest according to Standard Operating Procedures in the manufacturing facility in an appropriately-sized CryoMACS Freezing Bag, or administered fresh (depending on cell culture growth, patient condition, etc). If cells are cryopreserved, infusion will be scheduled when final sterility culture results are available unless the subject's clinical condition requires immediate intervention. In addition, unavoidable delays to the infusion date may include any of the following:

- Subject develops a health condition, significant disease progression or infection prior to cell administration, such that the infusion criteria in Section **5.4.2.1** cannot be met; or
- Schedule conflict arises (patient or patient's family, intervening holidays, within manufacturing facility, etc.)

If it becomes necessary to infuse cells prior to the final sterility culture results, sterility release of the product will be completed based on preliminary reading of the culture results (Day 4-5) and negative gram stain. The Action Plan outlined in section **7.5.4**, will be followed in the unlikely event that a positive sterility or mycoplasma test is obtained after administration of the product.

5.4.2 GD2.BB.z.iCasp9-CAR T cell Infusion

Subjects will be hospitalized to receive treatment with GD2CART, if not previously hospitalized. An Ommaya intraventricular catheter will be inserted, if not already present, for close monitoring of intracranial pressure in subjects with DIPG. Subjects will undergo pre-cell infusion evaluation as per section **5.5.1** and section **9**. Subjects may remain hospitalized for 28 days or may be discharged if treatment-related AEs have resolved to Grade 1 or better at the discretion of the treating physician, but must stay in close proximity to Stanford, for at least 28 days post-infusion.

5.4.2.1 Cell Infusion Criteria

Subjects must meet the following criteria in order for cells to be infused (based on labs obtained within 24 hrs of cell infusion):

- GD2CART must have met release criteria (section **5.4.1**), except cell dose (see section **5.4.3**).
- Subject has no evidence of hemodynamic instability
- Subject has not developed a new requirement for supplemental oxygen therapy
- Subject has not developed symptoms concerning for new, systemic infection, significant disease progression, or any condition that in the opinion of the PI may pose an unacceptable risk to the subject
- There is no evidence of clinically significant cardiac dysfunction, serum creatinine > 2 x ULN, and no new onset acute neurological toxicity > Grade 1.
- No systemic corticosteroid therapy for at least 7 days prior to infusion.

If these criteria are not met, measures will be taken to resolve the underlying condition(s) and the cell infusion must be delayed until the event resolves. Fresh GD2CART cells may be infused within 72 hours of planned infusion or frozen for later administration. If the GD2CART infusion is delayed > 2 weeks, conditioning chemotherapy may be repeated**, at investigator discretion**.

5.4.2.2 Premedications

Subjects will receive levetiracetam 20 mg/kg/dose orally twice a day (maximum dose of 1000 mg/dose) beginning the day before cell infusion (Day -1) unless clinically contraindicated.

Subjects will receive the following medications 30-120 (±10) minutes prior to cell infusion:

- Diphenhydramine: 1 mg/kg/dose PO or IV
- Acetaminophen: 10-15 mg/kg/dose PO

5.4.2.3 Cell Infusion

Cell infusion will be scheduled when final sterility culture results are available unless the subject's disease status/clinical condition requires more urgent intervention. In addition, unavoidable delays to the infusion date may include any of the following:

- Subject develops a health condition or infection prior to cell administration, such that the infusion criteria in section **5.4.2.1** cannot be met; or
- Schedule conflict arises (patient or patient's family, intervening holidays, within the manufacturing facility, etc.)

Cells are delivered to the subject care unit by a staff member from BMT-CTF. Prior to infusion, the cell product identity label is double-checked by two authorized staff (MD or RN), an identification of the product and documentation of administration are entered in the subject's chart, as is done for blood banking protocols. Cell products should NOT be infused unless the product identification matches the subject's identification.

Cells are to be infused intravenously (IV) over 10-30 minutes (or as fast as tolerated based on volume and/or DMSO toxicity) via non-filtered tubing, gently agitating the bag during infusion to

prevent cell clumping. After infusing cells, rinse the infusion tubing to ensure complete cell infusion according to institutional procedures. Documentation in the medical record should include the volume of cell infusion, the thaw start/stop time (if cells are cryopreserved), and cell product infusion time start/stop times.

Second doses of GD2CART may be administered intracerebroventricularly as described in **Section 5.6.2** and **Section 13.9**.

5.4.3 Determination of Cell Dose

Cell dose will be body weight-based according to the following rules:

The dose of GD2CART for each dose cohort will be based on doses calculated using actual body weight, unless subject has BMI > 30, within 7 days of starting cell manufacturing. Subjects with BMI greater than 30 may have doses calculated using ideal body weight (IBW) plus 50% of the difference between actual weight and IBW (see section **13.3,** Appendix C for calculation).

If cell growth limitations preclude administration of the number of cells targeted for the assigned cohort level, the subject will receive as many cells as possible, as the efficacious dose is not yet known, and the subject will be evaluable for feasibility of manufacturing (as per section **12.2.1)** but will not be evaluable for safety in that dose cohort, and may be replaced in the numbers for that dose cohort.

5.4.4 Dose Escalation to Determine MTD/RP2D

There will be a Phase 1 dose-escalation design with three dose cohorts of subjects with DIPG or spinal DMG to determine the MTD/RP2D. The number of GD2CART for each dose level cohort described in **Table 3**:

Table 3: GD2CART Dose Levels

Dose Escalation Schedule	
**Dose Level**	**Dose of GD2-CART**
Cohort Level -1	3 x 10^5 transduced T cells/kg (± 20%)
Cohort Level 1	1 x 10^6 transduced T cells/kg (± 20%)
Cohort Level 2	3 x 10^6 transduced T cells/kg (± 20%)
Cohort Level 3	10 x 10^6 transduced T cells/kg (± 20%)

Each dose cohort will initially include a minimum of 3 subjects. We propose to conduct the safety assessment in subjects with DIPG or spinal DMG at any age, rather than initially targeting adolescents or adults for three specific reasons:

1. Pontine disease is rare in adolescents and adults
2. Evaluation of safety in spinal DMG, which occur more commonly in adolescents and young adults, will not inform safety for pontine DMG since much of the risk of toxicity relates to the location of the tumor. Because we do not anticipate the potentially life

threatening intracranial complications of pontine DIPG in the subjects with spinal DMG, we propose to allow the safety of GD2CART in the pontine DIPG arm to inform the safety of dose escalation in spinal cord arm.

3. Enrollment of adolescents or adults prior to children is not necessary since first, second and third generation CARs targeting GD2, incorporating the same scFv have already been tested in several clinical trials and have demonstrated safety and significant clinical activity[33],[34],[35].

A 28 day safety assessment period will follow infusion of GD2CART of the first subject in each dose cohort. Subsequent subjects in that cohort and subsequent cohorts may be treated after a two week (14 day) safety assessment period. Four weeks (28 days) must elapse after cell infusion in the final subject in each dose cohort to allow for safety assessment of DLTs (as defined in section **5.4.5**) before treating subjects on the next higher dose cohort level. Therefore, treatment in the next higher dose cohort will not proceed to a higher dose level until all subjects have been treated in that disease arm in the prior dose cohort and the last subject treated on the completed dose cohort has been observed for at least 28-days after infusion of GD2CART.

If more than one DLT occurs in the first dose level of either disease group, (2 out of 6) the dose will be de-escalated to Dose level -1 (3e5 transduced T cells/kg (± 20%)) in that disease group for safety evaluation. If cell growth limitations preclude administration of the number of cells targeted for the assigned cohort level, the subject will receive as many cells as possible, as the efficacious dose is not yet known, and the subject will be evaluable for feasibility of manufacturing but will not be evaluable for safety in that dose cohort, and may be replaced in the numbers for that dose cohort. If the 3rd dose level is completed in a disease group without DLT, an MTD may not be determined. This will be considered the 'highest cell dose' studied in that disease group. The RP2D may be the MTD or may be a lower dose chosen by the investigator, in collaboration with the sponsor, based upon feasibility, tolerability and clinical activity. If clinical activity is observed in a dose cohort, despite no observed DLTs, the investigator, in collaboration with the sponsor, may elect to expand that dose cohort to further explore the clinical activity while minimizing the risk of toxicities.

Dose escalation will follow the rules outlined in the **Table 4**.

Table 4: Dose Escalation Rules

Number of Subjects with DLT at a Given Dose Level	Escalation Decision Rule
0 out of 3	Enter up to 3 subjects at the next dose level. If 0 out of 3 occur, dose may escalate.
≥ 2	Dose escalation will be stopped. This dose level will be declared the maximally administered dose (highest dose administered). Up to three (3) additional subjects will be entered at the next lowest dose level if only 3 subjects were treated previously at that dose.

1 out of 3	If DLT develops in the first subject at Dose Level 1 in a disease group, the dose will be de-escalated to Dose Level -1 (3 x 10^5 transduced T cells/kg (± 20%)), and accrual to Dose Level -1 will proceed to evaluate safety in that disease group. If DLT develops in any subject at Dose Level -1, accrual will be temporarily stopped to that disease group while consultation with the IRB, APB and FDA occurs.<br><br>If DLT develops in one of the 3 subjects at Dose Levels 2 or 3, the cohort will be expanded to 6 subjects in that disease group. If no additional subjects develop DLT, MTD will not have been exceeded and the next dose level can be administered after the 28 day safety assessment period of the last subject at this dose level.<br><br>If 1 or more of this group suffer DLT, then dose escalation is stopped, and this dose is declared the maximally tolerated dose. Up to three (3) additional subjects will be entered at the next lowest dose level if only 3 subjects were treated previously at that dose.
≤1 out of 6 at highest dose level below the maximally administered dose	This is the MTD for a disease group and is generally the recommended phase 2 dose. At least 6 subjects must be entered at this dose level.

5.4.5 Dose Limiting Toxicity

Adverse events that are considered disease-related (not suspected of relationship to GD2CART) will not be considered dose-limiting toxicities. Only those AEs suspected to be related to the investigational agent, GD2CART will be used in the definition of DLT. Toxicities occurring after initiation of the conditioning lymphodepletion chemotherapy regimen but prior to GD2CART infusion, will primarily be attributable to the chemotherapy administration or disease, if not extraneous causes. After cell infusion, toxicities will be evaluated for temporal and causal relationship to conditioning lymphodepletion chemotherapy regimen versus cell infusion. Some symptoms may overlap and attribution will not be clearly definable, in which case, toxicities will be attributed as possibly related to both preparative regimen and cell infusion. Toxicities will be attributed to the T cells if: 1) they were NOT present before T cell infusion; OR 2) they increase in Grade in temporal association with the T cell infusion; AND 3) they are not clearly explained by other factors.

Subjects who are fully assessable for DLT are those that have completed the conditioning lymphodepletion chemotherapy regimen and received the GD2CART infusion. Chemotherapy-related toxicities experienced by subjects who are unable to receive GD2CART *will not* be considered evaluable in the definition of DLT.

See section **7.2** Causality for definitions of 'suspected'. The definition of DLT in these studies uses NCI's Common Terminology Criteria for Adverse Events (CTCAEv5.0), the grading system for CRS and neurotoxicity in Appendix B, section **13.2**.

5.4.5.1 Definition of DLT:

Adverse events that are at least possibly related to the investigational agent (GD2CART) with onset within the first 28 days following GD2CART infusion will be considered DLTs as follows:

Hematological Toxicity:

Hematologic toxicities are common after CAR T cell therapies therefore, the following criteria will be considered:

- Subjects with abnormal counts at baseline will not be evaluable for hematological toxicity
- Subjects evaluable for hematologic toxicity: Any grade 4 neutropenia or thrombocytopenia lasting > 14 days despite best supportive care is a DLT.

Non-hematologic Toxicity:

- Any grade 5 toxicity
- CRS toxicity Grade 4 in severity, or Grade 3 in severity for greater than 7 days (see Appendix B, section **13.2**.)
- Infusion reactions ≥ Grade 2 in severity lasting more than 24 hours despite standard supportive care.
- Grade 4 neurotoxicity, (as defined in CTCAEv5 as life threatening, urgent intervention needed) for greater than 96 hours (see Appendix B, section **13.2)**.
- Any new Grade 3 neurotoxicity (not present at baseline and excluding cranial neuropathies and ataxia, which are common in this disease) lasting longer than 28 days.
- Any other Grade 3 or greater, non-hematological toxicity which is possibly, probably, or definitely attributed to investigational product (GD2CART) with exclusion of transient, laboratory abnormalities without clinical consequence, and occurring within 28 days of investigational product administration will be considered a DLT with the following **exceptions**:
    - Grade 3 neurotoxicity
    - Grade 3 diarrhea that resolves to ≤ grade 2 within 4 days;
    - Grade 3 low electrolyte levels that are correctable and asymptomatic, Grade 3 hypoalbuminemia;
        - Hypocalcemia toxicity grade should be assigned based on the calcium level corrected for degree of hypoalbuminemia according to the following formula: For every albumin decrease of 1 gm/dL a total calcium increase of 0.2 mmol/L is to be made;
    - Abnormal coagulation parameters in subjects on anticoagulant therapy or with pre-existing coagulopathy;

- Grade 3 transaminase, alkaline phosphatase, bilirubin or other liver function test elevation, provided there is resolution to ≤ grade 2 or baseline within 14 days. Grade 4 transaminitis lasting < 72 hours will also not be considered DLT;
- Grade 3 or 4 fever lasting ≤ 14 days, as this is common and expected with CAR therapy and may be a biomarker for an effective immunotherapeutic regimen;
- Grade 3 or 4 infection or neutropenic fever unless infection is not considered likely related to cyclophosphamide/fludarabine and relationship to GD2CART is suspected (Note: Grade 4 infection uncontrolled for > 7 days will be considered DLT);
- Grade 3 nausea and/or anorexia.

As noted in section **2.5.4** CRS will be graded according to the ASTCT CRS Consensus Grading[52] (See Appendix B, section **13.2**).

5.4.5.2 Definition of Maximal Tolerated Dose (MTD)

The MTD is a dose level immediately below the level at which the enrollment is stopped in one disease group due to DLTs, as explained specifically below:

- If **<u>more than one subject</u>** in the first three subjects in a disease group in a dose level experience DLT as defined above, MTD will have been exceeded.
- If DLT develops in **<u>one</u>** of the 3 subjects in a disease group included in a cohort, the cohort will be then expanded up to six:
- If 2 or more of these 6 included subjects develop DLT, the MTD will have been exceeded.

If no additional subjects in that disease group develops a DLT, the MTD will not have been exceeded and the next dose level can be administered after the 28-day safety assessment period of the last subject at the dose level.

If MTD is exceeded at any dose level, three subjects will be added to the immediate lower dose level, unless it has been previously expanded to six. If less than 2 of 6 subjects develop DLT at that level, it will be defined as the MTD.

5.4.6 Dose Expansion Cohort

To gain further experience with the safety, feasibility and clinical activity of the GD2CART in subjects with DIPG and subjects with spinal H3K27M DMG, the RP2D cohort will expand in two groups to include a total of 20 subjects with DIPG and 10 subjects with DMG treated, including those subjects treated at the RP2D during the dose escalation phase as described in section **4.1.2**. The main goal for the dose expansion portion of the trial is to collect data about safety and tolerability from a larger group of patients receiving treatment at the RP2D. A secondary goal is to collect data about efficacy, such as overall survival, in order to compute point, interval, and quantile estimates in subjects with DIPG as per section **12.4**. The number of subjects with spinal H3K27M DMG is not anticipated to be significant, hence the clinical activity (OS) in up to 10 subjects treated at the RP2D will be reported separately and descriptively.

5.5 Evaluations and Follow Up

Subjects will be hospitalized prior to receipt of GD2CART, if not before administration of conditioning lymphodepletion chemotherapy regimen. Subjects will be monitored closely inpatient or in close proximity to Stanford for at least 28 days, at the investigator's discretion. For all patients, neurooncologists, neurosurgeons and neurointensivists will be consulted and involved in every case and neurointensive care provided for management of neurologic deterioration and/or increase in intracranial pressure.

During this post cell infusion period, procedures will be completed at the following time points as outlined in the Section **9** Study Calendar:

5.5.1 Evaluation Prior to Cell infusion

Prior to cell infusion subjects will undergo safety evaluations with general laboratory tests, physical exam, neurologic exam, vital signs, adverse event collection, concomitant medication monitoring, and correlative sample collection.

Once, any time between enrollment and start of initial cell infusion, a blood sample for replication competent lentivirus (RCL) testing will be collected and sent to Indiana Gene Therapy Testing Laboratory (IU GTTL), as described in section **13.4**.

Please refer to the section **9** Study Calendar for a list of all procedures, including the following:

- Measurement of ICP via Ommaya Reservoir (baseline) prior to cell infusion in patients with Ommaya catheter
- Disease evaluation with Brain MRI will be performed in subjects with DIPG within 28 days prior to starting lymphodepleting chemotherapy.

5.5.2 Required monitoring during cell infusion:

Monitoring will include vital signs (blood pressure, heart rate, respiratory rate, oxygen saturation, and temperature) prior to infusion, within 15 minutes after start of infusion (± 10 min), and then 30 (± 10 min), 60 (± 10 min), and 120 (± 10 min) minutes after infusion. ICP monitoring will be performed in the ICU and emergency measures for treatment of increased intracranial pressure will be available.

- ✓ Supplemental oxygen will be available at the bedside.
- ✓ If an allergic or other acute reaction occurs, studies appropriate for investigation of a transfusion reaction will be performed (urinalysis, CBC, Coomb's test).
- ✓ Acute reactions or increased intracranial pressure will be treated according to institutional standards of care, while avoiding steroid administration if possible.

5.5.3 Evaluations Post cell Infusion Day 1 to Day 27

After completing GD2CART infusion all subjects will be followed in the post treatment assessment period. Counting from Day 0 (GD2CART infusion), subjects will undergo the following evaluations:

- Day 1 to Day 14: Daily during hospitalization or 5 times per week if outpatient (with no more than 48 hours between evaluations)

- Day 15 to Day 27, Daily during hospitalization or twice per week if outpatient (± 4 days)
- Any time the subject's clinical condition changes, clinical best practices will be used to determine the frequency of evaluation

Please refer to the section **9** Study Calendar for a list of all procedures at each visit.

Procedures will include safety evaluations with general laboratory tests, targeted physical exams (as clinically indicated), neurologic exams with measurement of ICP via Ommaya Reservoir and spinal cord assessment including lumbar puncture opening pressure assessments (optional unless clinically indicated) in subjects with spinal DMG (See section **13.2.5.1)**, adverse event collection, concomitant medication monitoring, and correlative sample collection. Vital sign monitoring (including blood pressure, heart rate, oxygen saturation, respiratory rate, and temperature) should be performed every shift (± 2 hours) (at least every 8 hours) or as clinically indicated during hospitalization and with each visit after discharge. Neurotoxicity evaluations in the form of ICE score/ CAPD evaluations should be performed every shift (± 2 hours) (at least every 8 hours) and with any change in neurologic functioning during the initial hospitalization for cell infusion.

- If any evidence of increased intracranial pressure, changes in spinal cord function or clinical deterioration suspected due to neurologic compromise, patient will be transferred to the ICU (see section **13.2.4**and section **13.2.5**, Appendix B) or re-admitted to the hospital if previously discharged.
- Disease evaluations will be specific to the subject's location of disease and may include imaging studies as follows to be conducted as clinically indicated:
  - Subjects with DIPG will undergo brain MRI on D7 (optional), D14 (optional), and D21 (optional) at the investigator's discretion. If clinical condition post-infusion prevents MRI, and a scan is determined to be needed, a bedside CT will be obtained on those days. Standard MR imaging will include Sagittal T1 MPRAGE, axial DWI, axial T2 FLAIR, axial T2, and post gadolinium sagittal T1 MPRAGE (with reconstructions) images. The standard MR parameters are listed on the PBTC NIC web page located at http://www.childrenshospital.org/research/centers-departmental-programs/pediatric-brain-tumor-consortium-neuroimaging-center under Neuroimaging Studies/ Specific MR Imaging Sequences- Open PBTC Protocols.
  - Subjects with spinal DMG will undergo MRI with and without gadolinium and/or CT or PET/CT

Monitoring of CRP, ferritin, and LDH (only if LDH is elevated at baseline) levels may assist with the diagnosis and define the clinical course in regards to CRS/neurotoxicity. It is therefore, recommended that CRP and LDH (if elevated at baseline) be monitored when lab specimens are drawn starting at Day 0 and continuing through hospitalization. In addition, lactate can be monitored as clinically indicated. The above schedule may be modified as needed based on the subject's clinical condition and the investigator's medical judgement.

5.5.4 Evaluation on Day 28 (± 4 days)

Day 28 (± 4 days) will be the first disease evaluation post cell infusion. Evaluations will include general laboratory tests, full physical exam, neurologic exam, performance status, weight, vital

signs, adverse event collection, concomitant medication monitoring, disease evaluation, including radiologic evaluation (**Section 10.1.2.1**) and clinical evaluation of neurologic status (**Section 13.8**), and correlative sample collection as per section **9**.

5.5.4.1 Evaluations of subjects who Do NOT receive GD2CART

The following procedures/assessments will be completed for subjects who are enrolled but do not receive GD2CART, at the time points outlined in the Section **9** Study Calendar until disease progression and/or toxicities attributable to the study participation resolve to ≤ Grade 1 or stabilize:

- Disease assessment per standard of care
- Adverse/Serious Adverse Event reporting and concomitant medication documentation until 30 days after last procedure (e.g., leukapheresis, conditioning chemotherapy).

Should the subject fail to return to the clinic for a scheduled protocol specific visit, sites will need to make 2 attempts by a combination of telephone and mail to contact the subject. Sites must document both attempts to contact the subject. If a subject does not respond within 1 month after the second contact the subject will be considered lost to follow-up and no additional contact will be required.

5.5.5 Schedule and Procedures For Post Treatment Assessment Period (after Day 28) after GD2CART

After Day 28, subjects who received GD2CART will return to the clinic at the following intervals, or be seen by their local provider for evaluation, with telehealth visits at Stanford when feasible, unless there is disease progression and/or alternative disease therapy is started, at which time the subject will proceed to long term follow up (see section **5.5.6)**:

- Month 2 (± 2 weeks)
- Month 3 (± 2 weeks)
- Month 6 (± 4 weeks)
- Month 9 (± 4 weeks)
- Month 12 (± 4 weeks)

Evaluations of disease status in subjects who have not progressed after GD2CART will be conducted at Stanford (or be seen by their local provider for evaluation, with results and scans sent to Stanford for review and data collection; supplemented by telehealth visits with Stanford when feasible) for the first 24 months. Subsequent disease evaluations may be performed by the outside medical facility/care provider as per standard of care. If subject's disease has not progressed by Month 24, disease assessments will continue to be performed per standard of care.

Should a subject fail to return to the clinic for a scheduled protocol specific visit, sites will need to make 2 attempts by a combination of telephone and mail to contact the subject. Sites must document both attempts to contact the subject. If a subject does not respond within 1 month after the second contact the subject will be considered lost to follow-up and no additional contact will be required.

The procedures to be completed in subjects who have received GD2CART cells is outlined in the Section **9** Study Calendar and will include general laboratory tests, physical exam, neurologic

exam, performance status, weight, vital signs, adverse event collection, concomitant medication monitoring, disease evaluation, and correlative sample collection as per section **13.7**.

5.5.6 Long Term Follow-up Period

Subject who complete month 24 visit without developing disease progression, will be followed as per section **5.5.6.1**.

Subjects who:

- did not respond to treatment (i.e., did not achieve a CR, PR, or clinical benefit), or
- progress following a response and is either not eligible for re-treatment or chooses not to pursue re-treatment, or
- proceed to alternative disease therapy,

will undergo follow up as per section **5.5.6.2**.

All subjects who received CAR T cells, regardless of disease status or alternative therapy options, will undergo long term gene therapy follow up as per section **5.5.6.3**.

Should a subject fail to return to the clinic or respond to contact for a scheduled protocol specific visit/contact, sites will need to make 2 attempts by a combination of telephone and mail to contact the subject. Sites must document both attempts to contact the subject. If a subject does not respond within 1 month after the second contact the subject will be considered lost to follow-up and no additional contact will be required.

5.5.6.1 Long Term Follow-up Schedule and Evaluations for subjects who have **not** developed progressive disease (have an ongoing response)

5.5.6.1.1 Schedule

Subjects who received GD2CART and have NOT developed progressive disease will begin the long term follow-up period after they have completed Month 24 visit:

- Every 6 – 12 months (± 2 months) for 5 years
- Beginning with year 6 (± 3 months), subjects will return to the clinic or be contacted by phone, e-mail or mail with questionnaire, one time annually for up to 15 years as per section **5.5.6.3**.

5.5.6.1.2 Evaluations

The procedures in section **9** Study Calendar will be completed for all subjects who received GD2CART, who have NOT developed progressive disease, at the time points outlined above and will include general laboratory tests, physical exam, neurologic exam, performance status, and disease evaluation (radiologic and Clinical Evaluation of Neurologic Status [section **13.8**]).

Evaluations of disease status in subjects who have not progressed after GD2CART will be conducted for the first 24 months. Subsequent disease evaluations will be performed as per standard of care. Once a subject develops disease progression, no further disease evaluations are required on this study. All subjects will be followed for long term gene therapy follow up as well as survival and subsequent therapies.

5.5.6.2 Long Term Follow-up Schedule for subjects who develop progressive disease, are not eligible or decline retreatment, or proceed to alternative disease-directed therapy

5.5.6.2.1 Schedule

Subjects who received GD2CART who develop progressive disease, are not eligible or decline retreatment, or proceed to alternative disease-directed therapy will begin the long term follow-up period as outlined below:

- Month 3, 6 and 12 for the first year.
- Every 12 months (± 2 months) for 5 years
- Beginning with year 6 (± 3 months), subjects will return to the clinic or be contacted by phone, e-mail or mail with questionnaire, one time annually for up to 15 years after the date of cell infusion as per section **5.5.6.3**.

5.5.6.2.2 Evaluations

The following procedures will be completed at the time points outlined above and section **9** Study Calendar:

- Physical exam, vital signs and performance status for year 1 through 5 (may be performed by the local physician)
- Survival and subsequent therapies
- **Annual** evaluations in the first 5 years should specifically document any new malignancies, new incidence or exacerbation of a pre-existing neurologic disorder, new incidence or exacerbation of a prior rheumatologic or other autoimmune disorder, new incidence of a hematologic disorder; and other factors that may be relevant to the feasibility and scientific value of the long-term follow up observations (i.e. exposure to other cancer causing agents).

5.5.6.3 Safety Assessment Testing for Replication Competent Retrovirus in Retroviral Vector Based Gene Therapy Products during Subject Follow up

In compliance with OSP and FDA's ***Guidance for Industry: Gene Therapy Clinical Trials – Observing Participants for Delayed Adverse Events (2006)***, subjects who have received at least one dose of a genetically engineered cellular therapy will be evaluated for long term safety and occurrence of adverse events according to the requirements established by FDA guidance and the NIH Guidelines for Research Involving Recombinant or Synthetic Nucleic Acid Molecules (NIH Guidelines) (2016).

- Long term follow up (**year 1 - 5**) for gene therapy related toxicities
    - Targeted Physical Exam and History –

      Gene Therapy annual evaluations should specifically document any new malignancies, new incidence or exacerbation of a pre-existing neurologic disorder, new incidence or exacerbation of a prior rheumatologic or other autoimmune disorder, new incidence of a hematologic disorder; and other factors that may be relevant to the feasibility and scientific value of the long-term follow up observations (i.e. exposure to other cancer causing agents).

- Targeted Adverse/Serious Adverse Event reporting including neurological, hematological, infections, autoimmune disorders, and secondary malignancies
- Targeted concomitant medication documentation including immunosuppressive drugs, anti-infectives, and vaccinations
- Blood Samples (archived) for RCR

  RCR blood samples must be collected at Month 3 (±1 month), Month 6 (± 1 month) and Month 12 (± 2 months) and sent to Indiana University Gene Therapy Testing Laboratory.

  After month 12, RCR blood samples will be collected annually for the next 4 years (for a total of 5 years) and banked if all RCR tests in first year were negative, with a brief clinical history conducted annually. If any post-treatment samples are positive, further analysis of the RCR and more extensive subject follow-up will be undertaken, in consultation with the FDA.

  If a subject dies or develops neoplasms during this follow up, efforts will be made to assay a biopsy sample for RCR.

  If any subject has more than 5% persistence of gene-transduced cells at Month 6 using 1A7, an anti-idiotype antibody that recognized the GD2CART, samples will be studied for clonality of persisting gene transduced cells. Such techniques may include analysis of BV chain expression, T cell cloning or LAM-PCR. If a predominant or monoclonal T cell clone derived from gene transduced cells is identified during the follow-up, the integration site and sequence will be identified and subsequently analyzed against human genome database to determine whether the sequences are associated with any known human cancers. If a predominant integration site is observed, the T cell cloning or LAM-PCR test will be used at an interval of no more than 3 months after the first observation to see if the clone persists or is transient. In all instances where monoclonality is persistent and particularly in instances where there is expansion of the clone, regardless of whether or not the sequence is known to be associated with a known human cancer, the subject should be monitored closely for signs of malignancy, so that treatment, if available, may be initiated early.

- Long term follow up (**year 6-15**) for gene therapy related toxicities

  Once a subject has completed 5 years of follow up without evidence of delayed adverse events secondary to genetically engineered cellular therapy product, the following evaluation will be conducted annually for the subsequent 10 years (± 2 months):

  The subject will be contacted (in clinic or via phone or written questionnaire) to evaluate for development of delayed adverse events (See questionnaire in section **13.5,** Appendix E).

  - If vector modified cells were detected in the blood during the previous visit, then blood for persistence of vector modified cells will be collected and tested until negative.

- Subjects are requested to inform the study research team of any changes to e-mail (if consented to use e-mail), phone, and address. E-mails will be sent to subjects via the Secure Email to ensure securely and confidentially over an SSL/encrypted connection.
- Distribution and collection of questionnaires

A draft letter to subject's primary doctor is provided in section **13.6,** Appendix F. This letter should be sent to the local health care provider via mail, fax or e-mail at the time the subject is referred back to the care of the local physician or with any change in primary doctors. E-mails will be sent to subjects via the Secure Email to ensure securely and confidentially over an SSL/encrypted connection.

- Subjects will be sent a request for information (**section 13.5**, Appendix E) and questionnaire annually.
  - If there is no response within 1 month, the study coordinator will follow up with a telephone call to request the information. The questionnaire may be completed with the subject responses over the phone at this time.
  - If there is no response to the telephone call, the letter and questionnaire (section **13.5,** Appendix E) should be sent to the subject via FedEx, signature required.
  - Each attempt to contact the subject and outcome must be documented in the medical record.

5.6 Option for Additional Dose(s) of GD2CART

On the day the cell product is harvested, remaining cells that have been produced above and beyond the number of cells needed for a subject's dose level, research aliquot and QA retention vials will be cryopreserved using standard techniques. If an adequate number of additional cells are available to meet the numbers for an additional dose (with corresponding QA retention vials), the subject may be offered the option of retreatment (additional infusion of GD2CART (either intravenous or intraventricular route) with or without preparative chemotherapy regimen, toxicity assessment and research blood sampling) if the following criteria are met:

5.6.1 Eligibility Criteria for subsequent cell infusions:

- ✓ For IV dosing, cell infusion of GD2CART has been deemed safe in at least 2 subjects at that dose without experiencing DLTs; or a flat dose of GD2CART for ICV dosing.
- ✓ Potential for Benefit / Response to previous infusion. Subjects who in the judgement of the investigator may benefit and/or subjects who had at least a PR or SD with clinical benefit will be eligible for an additional cell infusion either intravenous or intraventricular. Subjects that initially had a CR may only receive a second dose if evaluable disease recurs. Clinical benefit is indicated by an improvement in the subject's health status or improvement in clinical evaluation of neurologic status compared to baseline.
- ✓ At least 30 days have passed since the previous cell infusion.
- ✓ Circulating levels of GD2CART must be < 5% by flow cytometry.

- ✓ Any toxicity (regardless of causality) after the previous GD2CART cell infusion must resolve such that subjects meet all the initial eligibility criteria for major organ function and therapy washout as outlined in **3.1.**
- ✓ An adequate number of cryopreserved GD2CART and QA retention vials or an adequate number of apheresis product sufficient to generate an additional dose of GD2CART must be available if administering cells via intravenous route. If intracerebroventricular catheter (ICV) route is determined to potentially be the most efficacious, 30 X 10^6 GD2CART cells ± 20%, will be administered, or if total dose is <30 X 10^6 GD2CART cells, all available cells manufactured will be administered.

The cell dose (based on CAR transduced cells) for the second infusion shall not be greater than the current dose level completed or the safe dose if this has been determined.

As a rule, subjects who incurred DLT after receiving the first cell infusion will not be eligible to receive additional cell infusions unless IRB, APB and FDA approval is granted on a case by case basis.

5.6.2 Procedures for Additional Doses

5.6.2.1 Lymphodepleting chemotherapy

Subjects may receive additional antineoplastic and lymphodepleting chemotherapy prior to the second infusion of GD2CART. At the investigator's discretion, an intensified lymphodepleting chemotherapy regimen (fludarabine 30 mg/m^2 per day IV for 4 days and cyclophosphamide 600 mg/m^2 per day IV for 4 days, resulting in 120 mg/m^2 of fludarabine and 2400 mg/m^2 of cyclophosphamide) may be administered for subjects receiving IV administration of GD2CART. A similar dosing has been used successfully at the NCI Pediatric Oncology Branch without increased toxicity (Personal communication, N. Shah, Pediatric Oncology Branch, National Cancer Institute, NIH). Colleagues' experience at Memorial Sloan Kettering (using CD19-28z CAR) using single-agent Cyclophosphamide (up to 3 Grams over 2 days) have reported significantly better responses with high dose (3G Cyclophosphamide - responses of 94%) as compared to low dose (1.5G Cyclophosphamide - responses of 38%) (Personal communication). For ICV administration, lymphodepleting chemotherapy may be omitted at the investigator's discretion.

5.6.2.2 Cell Product Preparation and Administration: Intravenous (IV) or intracerebroventricular (ICV) through Ommaya Catheter

5.6.2.2.1 IV route: If the cryopreserved product is outside the farthest GD2CART stability plan time point, a QA retention vial sample will be thawed and tested for cell viability, which must meet the original IND release criteria. After QA retention vial samples have been tested and met the original release criteria for cell viability, or the cryopreserved product confirmed to be within the known stability timeframe, the cellular product will be thawed and administered IV over approximately 10-30 min or as tolerated based on volume status and/or DMSO toxicity.

5.6.2.2.2 ICV route: The previously manufactured cryopreserved cell product will be thawed, washed according to standard operating procedure in BMT-CTF to remove

cryoprotectant, and resuspended in 0.9% Normal Saline, USP to a volume of 3 mL (± 2 mL) in a sterile syringe for intraventricular administration through the Ommaya Catheter (see **Section 13.9** and separate SOP). Samples will be obtained from the final product for sterility testing, including a rapid release test for gram stain with a sample sent to Stanford Microbiology lab for 14 day sterility and mycoplasma testing. The resuspended GD2CART product will be transported by BMT-CTF staff with the applicable Chain of Custody Form to the bedside for intraventricular administration using Stanford Standardized procedure for Intraventricular administration via Ommaya Reservoir (Adult, Peds) (see steps in **section 13.9 Appendix I: Intracerebroventricular Catheter (ICV) Administration via Ommaya Reservoir (Adult/Peds)**.

Any subject who receives subsequent doses of GD2CART will NOT be evaluable for toxicity purposes of this study as they will be beyond the 28 day observation period for DLTs after their first cell infusion. However post-infusion monitoring will be the same as for the 1st infusion and all toxicities, including secondary reactions, will be recorded and reported. If two or more subjects develop Grade 4 toxicity at any time following the second GD2CART infusion that is felt to be possibly, probably or likely related to the CAR T cells, then second infusions will be paused pending discussion with the FDA, IRB and APB regarding continuing second infusions as part of the experimental regimen.

5.6.2.3 Gene Therapy Follow Up

If a subject receives more than one cellular product, the timing of gene therapy long term evaluations is restarted with each subsequent gene therapy administration. For example, a subject receives gene therapy and has undergone long term follow up for 1.5 years, develops progressive disease and is treated with a second gene therapy product. That subject will begin the blood sample collection at 3, 6 and 12 months (± 1 month) post cell administration and continue for a total of 15 years of follow up from the LAST gene therapy administration.

6 INVESTIGATIONAL AGENT AND COMMERCIAL DRUG INFORMATION

6.1 GD2.BB.z.iCasp9-CHIMERIC ANTIGEN RECEPTOR (GD2-CAR) RETROVIRAL TRANSDUCED AUTOLOGOUS PERIPHERAL BLOOD LYMPHOCYTES (GD2CART); FOLLOWING FLUDARABINE AND CYCLOPHOSPHAMIDE

6.1.1 Description

Cell therapy production will be conducted according to the SOPs in the manufacturing facility and must meet the requirements for a cGMP facility. The GD2.BB.z.iCasp9 replication incompetent retroviral vector has been manufactured by Bellicum Pharmaceuticals. All procedures will take place using good manufacturing process guidelines.

6.1.2 Stability:

Stability testing is ongoing. In the event a subject elects to receive retreatment, and adequate numbers of cells exist, stability will be evaluated prior to lymphodepleting chemotherapy and product thaw by cell viability analysis of a QA retention vial.

6.1.3 Administration

Guidance on administration of intravenous GD2CART is specified in section **5.4.2.** Guidance on administration of intraventricular GD2CART is specified in **section 13.9 Appendix I: Intracerebroventricular Catheter (ICV) Administration via Ommaya Reservoir (Adult/Peds)**.

6.1.4 Toxicities

In addition to standard transfusion types of reactions (chills, fever, rigors), the toxicities specific to CAR T cell therapy are described in detail in section **2.5**. Every effort will be made to mitigate the risk of these toxicities with the intensive monitoring plans outlined in section **5** and the supportive care measures outlined in section **4.2.** With growing experience with CAR therapy world-wide, the risk of severe neurotoxicity is increasingly better recognized—most notable with the recent occurrences of fatal neurotoxicity. With the incorporation of mandatory anti-seizure prophylaxis and prospective monitoring of neurologic function, we plan to rigorously monitor and evaluate for any neurotoxicity, with a plan to treat neurotoxicity aggressively.

Therapeutic interventions will generally include hypertonic saline, CSF removal via the Ommaya reservoir, and mannitol and dexamethasone. If these measures fail to control the increased ICP, consideration will be given to administering dasatinib to inhibit CAR function[50] and/or administration of AP1903 to permanently ablate the CAR T cells. The AP1903 drug will be administered as a suicide switch to deplete the genetically engineered cells. Avastin may also be utilized as it has shown some efficacy in the setting of tumor associated edema. Algorithms that provide an systematic approach to neurotoxicity management for patients with DIPG and patients with spinal DMG enrolled on this trial are shown in section 13.2.4.and section 13.2.5 The algorithms will serve as a guideline but is not meant to supercede clinical judgment in the management of neurotoxicity on this trial, and deviations from the algorithms will not be considered protocol deviations.

6.2 Fludarabine

6.2.1 Description:

(Please refer to package insert for complete product Information) Fludarabine phosphate is a synthetic purine nucleoside that differs from physiologic nucleosides in that the sugar moiety is arabinose instead of ribose or deoxyribose. Fludarabine is a purine antagonist antimetabolite.

6.2.2 How Supplied:

It will be purchased by the institution's Pharmacy Department from commercial sources. Fludarabine is supplied in a 50 mg vial as a fludarabine phosphate powder in the form of a white, lyophilized solid cake.

6.2.3 Stability:

Following reconstitution with 2 mL of sterile water for injection to a concentration of 25 mg/ml, the solution has a pH of 7.7. The fludarabine powder is stable for at least 18 months at 2-8^{0}C; when reconstituted, fludarabine is stable for at least 16 days at room temperature. Because no preservative is present, reconstituted fludarabine will typically be administered within 8 hours.

Specialized references should be consulted for specific compatibility information. Fludarabine is dephosphorylated in serum, transported intracellularly and converted to the nucleotide fludarabine triphosphate; this 2-fluoro-ara-ATP molecule is thought to be required for the drug's cytotoxic effects. Fludarabine inhibits DNA polymerase, ribonucleotide reductase, DNA primase, and may interfere with chain elongation, and RNA and protein synthesis.

6.2.4 Storage:

Intact vials should be stored refrigerated (2-8°C).

6.2.5 Administration:

Fludarabine is administered as an IV infusion in an appropriate solution over 30 minutes as described in Section **5.3.**

6.2.6 Toxicities:

The primary side effect is myelosuppression; however, thrombocytopenia is responsible for most cases of severe and life-threatening hematologic toxicity. Serious opportunistic infections have occurred in subjects treated with fludarabine. Hemolytic anemia has been reported after one or more courses of fludarabine with or without a prior history of a positive Coomb's test; fatal hemolytic anemia has been reported. In addition, bone marrow fibrosis has been observed after fludarabine therapy. Other common adverse effects include malaise, fever, chills, fatigue, anorexia, nausea and vomiting, and weakness. Irreversible and potentially fatal central nervous system toxicity in the form of progressive encephalopathy, blindness, and coma is only rarely observed at the currently administered doses of fludarabine. More common neurologic side effects at the current doses of fludarabine include weakness, pain, malaise, fatigue, paresthesia, visual or hearing disturbances, and sleep disorders. Adverse respiratory effects of fludarabine include cough, dyspnea, allergic or idiopathic interstitial pneumonitis. Tumor lysis syndrome has been rarely observed in fludarabine treatment of CLL.

6.3 CYCLOPHOSPHAMIDE

(Refer to FDA-approved package insert for complete product information)

6.3.1 Description:

Cyclophosphamide is a nitrogen mustard-derivative alkylating agent. Following conversion to active metabolites in the liver, cyclophosphamide functions as an alkyating agent; the drug also possesses potent immunosuppressive activity. The serum half-life after IV administration ranges from 3-12 hours; the drug and/or its metabolites can be detected in the serum for up to 72 hours after administration.

6.3.2 How Supplied:

Cyclophosphamide will be obtained from commercially available sources by the institution's Pharmacy Department.

6.3.3 Stability:

Following reconstitution as directed with sterile water for injection, cyclophosphamide is stable for 24 hours at room temperature or 6 days when kept at 2-8^{0}C.

6.3.4 Administration:

It will be diluted in an appropriate solution and infused as described in Section **5.3.**

6.3.5 Toxicities:

Hematologic toxicity occurring with cyclophosphamide usually includes leukopenia and thrombocytopenia. Anorexia, nausea and vomiting, rash and alopecia occur, especially after high-dose cyclophosphamide; diarrhea, hemorrhagic colitis, infertility, and mucosal and oral ulceration have been reported. Sterile hemorrhagic cystitis occurs in about 20% of subjects; severity can range from microscopic hematuria to extensive cystitis with bladder fibrosis. Although the incidence of hemorrhagic cystitis associated with cyclophosphamide appears to be lower than that associated with ifosfamide, mesna (sodium 2-mercaptoethanesulfonate) has been used prophylactically as a uroprotective agent in subjects receiving cyclophosphamide. Prophylactic mesna is not effective in preventing hemorrhagic cystitis in all subjects. Subjects who receive high dose cyclophosphamide may develop interstitial pulmonary fibrosis, which can be fatal. Hyperuricemia due to rapid cellular destruction may occur, particularly in subjects with hematologic malignancy. Hyperuricemia may be minimized by adequate hydration, alkalinization of the urine, and/or administration of allopurinol. If allopurinol is administered, subjects should be watched closely for cyclophosphamide toxicity (due to allopurinal induction of hepatic microsomal enzymes). At high doses, cyclophosphamide can result in a syndrome of inappropriate antidiuretic hormone secretion; hyponatremia with progressive weight gain without edema occurs. At high doses, cyclophosphamide can result in cardiotoxicity. Deaths have occurred from diffuse hemorrhagic myocardial necrosis and from a syndrome of acute myopericarditis; in such cases, congestive heart failure may occur within a few days of the first dose. Other consequences of cyclophosphamide cardiotoxicity include arrhythmias, potentially irreversible cardiomyopathy, and pericarditis. Other reported adverse effects of cyclophosphamide include headache, dizziness, and myxedema; faintness, facial flushing, and diaphoresis have occurred following IV administration. Mesna (sodium 2-mercaptoethanesulphonate; given by IV injection) is a synthetic sulfhydryl compound that can chemically interact with urotoxic metabolites of cyclophosphamide (acrolein and 4-hydroxycyclophosphamide) to decrease the incidence and severity of hemorrhagic cystitis, but is not a required premedication for this study.

6.4 ACETAMINOPHEN (TYLENOL):

Will be given as a pre-medication. This agent will be provided by the institution's pharmacy. Please refer to the package insert for complete pharmaceutical information on this product.

6.5 DIPHENHYDRAMINE (BENADRYL):

Will be given as a pre-medication IV or orally. This agent will be provided by the institution's Pharmacy Department. Please refer to the package insert for complete pharmaceutical information on this product.

6.6 ANTIMICROBIAL PROPHYLAXIS

Subjects will receive appropriate antimicrobial prophylaxis (e.g., Bactrim for PCP and acyclovir for HSV and VZV prophylaxis) during fludarabine/cyclophosphamide treatment and for a minimum of 6 months following treatment. This agent will be provided by the institution's Pharmacy Department. Please refer to the package insert for complete pharmaceutical information on this product.

6.7 LEVETIRACETAM (KEPPRA):

Subjects will receive levetiracetam 20 mg/kg/dose orally twice a day (maximum dose 1000 mg/dose) beginning the day before cell infusion (Day -1). This dose may be adjusted based on symptoms of neurotoxicity and/or with recommendations of neurologic consult. Levetiracetam should continue through day 28 once neurotoxicity develops. This agent will be provided by the institution's Pharmacy Department. Please refer to the package insert for complete pharmaceutical information on this product.

6.8 MESNA

Pediatric subjects may be given mesna prophylactically as an uroprotective agent during and after the cyclophosphamide component of the conditioning lymphodepletion chemotherapy as described in Section 5.3. This agent will be provided by the institution's pharmacy. Please refer to the package insert for complete pharmaceutical information on this product.

6.9 AP1903 DIMERIZING AGENT

6.9.1 Description

AP1903, also known as Rimiducid, is a lipid-permeable tacrolimus analogue with homodimerizing activity. AP1903 homodimerizes an analogue of human protein FKBP12 (Fv) which contains a single acid substitution (Phe36Val) so that AP1903 binds to wild-type FKBP12 with 1000-fold lower affinity. This agent is used to homodimerize the Fv-containing drug-binding domains of genetically engineered receptors. Upon binding of AP1903 to the engineered FKBP12, caspase 9 activation ensues leading to endogenous caspase 3 activation and cells undergo apoptosis, beginning 30 minutes after infusion and peaking at 3 hours.

GD2CART contain the 'suicide' gene iCasp9; in the presence of AP1903, the iCasp9 promolecule dimerizes and activates the intrinsic apoptotic pathway, leading to cell death.

6.9.2 How Supplied:

AP1903 is provided as vials of 10 mg (5 mg/mL, 2 mL volume in a 3 mL vial) concentrate for injection. The contents of each vial is composed of the labeled content (10 mg) of AP1903 drug substance dissolved in a sterile, endotoxin free, 24% Solutol HS 15/Water for Injection solution, at an AP1903 concentration of 5 mg/ml and at pH of 5.0-7.5. Each vial is stoppered with a Teflon coated serum stopper and a yellow flip-off seal. The vial contains no preservatives.

AP1903 will be supplied by Bellicum Pharmaceuticals, who have provided authorization to cross reference to their IND.

6.9.3 Stability

Stable for at least 54 months when stored at 2-8°C.

6.9.4 Preparation and Administration

AP1903 should be warmed to room temperature prior to dilution. AP1903 is incompatible with materials containing plasticizer or DEHP and materials sterilized with ethylene oxide. The appropriate dose of AP1903 should be added to a a final concentration of 0.4 mg/mL in 0.9% Sodium Chloride Injection. The final preparation should be stored at room temperature until administered. Since the final product contains no preservatives, the infusion should be completed within 8 hours of preparation. While prolonged direct exposure to light of the AP1903 drug is not encouraged, there is no need to protect from light after preparation prior to administration.

Premedicate with acetaminophen, diphenhydramine and an H2 antagonist (e.g. famotidine) using standard dosing 15-30 minutes prior to AP1903. The diluted infusion should be administered at a dose of 0.4 mg/kg over 2 hours using a DEHP-free solution set that has been provided by Bellicum Pharmaceuticals. At the end of the infusion the line should be flushed with 0.9% Sodium Chloride Inj or 5% Dextrose Injection or any standard combination of these two fluids (e.g., 5% Dexrose in 0.45% Sodium Chloride Injection).

6.9.5 Toxicity:

None expected, the no observed effect level in dogs was 1000 mg/kg, which is much beyond the prescribed 0.4 mg/kg dose. Urticaria and flushing observed in one patient, which did not occur with subsequent AP1903 administration after premedication. In the same trial, one patient experienced a cytokine release reaction after receiving AP1903 following dendritic cell infusions.

7 ADVERSE EVENTS AND REPORTING PROCEDURES

7.1 Potential Adverse Events

7.1.1 GD2.BB.z.iCasp9-CAR T cells (GD2CART)

Because these cells have been previously administered in only a small number of humans, there may be unanticipated adverse events.

Section **2.4** and section **4.2.** discuss the potential risks of this investigational therapy based on previous studies with this or similar preparative regimens or cell products, including the risks of chemotherapy, risk of autoimmunity, risk of neurotoxicity, risk of the gene therapy component, and risk of cytokine storm. Transient, reversible mild effects have been observed with the administration of fresh cells, including chills, fever, rigors, diaphoresis, anorexia, nausea, diarrhea, headache and myalgias. General guidance for treatment of the most common toxicities are included in section **4.2** and section **13.2.2**.

7.1.2 Risk of Apheresis:

Apheresis is a safe procedure that is routinely performed in healthy adults. Participants will be closely monitored and procedures to minimize risks and prevent side effects are incorporated into

all aspects of the protocol. The institutions have broad expertise to adequately manage side effects. The potential risks of apheresis in this trial are as follows:

a. The most common side effects of apheresis are pain and bruising at IV sites. A central venous catheter may be required. Possible side effects include pain, bleeding, bruising, infection, thrombosis, vascular perforation.

b. During apheresis, mild side effects from citrate anticoagulant are common and include chills, numbness and tingling ("pins and needles"), anxiety, muscle cramps, and nausea. More serious side effects due to citrate-induced hypocalcemia are uncommon and include low blood pressure, seizures, weakness, and tetany. Citrate reactions rapidly resolve when the collection is slowed down or stopped. Prophylactic IV $CaCl_2$ and $MgSO_4$ infusions may be administered to subjects deemed to be at high risk of citrate toxicity. Risks of parenteral calcium and magnesium include extravasation necrosis and cardiovascular effects including bradycardia and blood pressure changes. However, side effects are unlikely given the low rate of infusion and use of large bore catheters for apheresis.

c. Transient mild thrombocytopenia is common after apheresis, but bleeding is unlikely.

d. Dilutional anemia occurs during apheresis, but this is unlikely to be clinically significant.

e. Side effects of blood draws include pain and bruising, lightheadedness, and rarely, fainting.

7.2 ADVERSE EVENT DEFINITIONS

7.2.1 Adverse Event

An adverse event is defined as any reaction, side effect, or untoward event that occurs during the course of the clinical trial associated with the use of a drug in humans, whether or not the event is considered related to the treatment or clinically significant. For this study, AEs will include events reported by the subject, as well as clinically significant abnormal findings on physical examination or laboratory evaluation. A new illness, symptom, sign or clinically significant laboratory abnormality or worsening of a pre-existing condition or abnormality is considered an AE.

Adverse events will be collected and documented from the start of lymphodepletion chemotherapy and conclude 30 days after the last dose of study treatment. Prior to start of conditioning lymphodepletion chemotherapy, only unexpected serious adverse events considered related or possibly related to study procedures (i.e. leukapheresis) will be recorded and reported. Additional disease directed therapy after completing apheresis but before the start of lymphodepletion chemotherapy, will not be allowed. Toxicities present at the initiation of lymphodepletion chemotherapy will be considered baseline conditions.

Serious adverse events that occur more than 30 days after the last administration of investigational agent/intervention and have an attribution of at least possibly related to the agent/intervention should be recorded and reported as per this Section.

An abnormal laboratory value will be considered a reportable, recordable AE if the laboratory abnormality is characterized by any of the following:

- Results in discontinuation from the study therapy
- Is associated with clinical signs or symptoms
- Requires treatment or any other therapeutic intervention
- Is associated with death or another serious adverse event, including hospitalization.
- Is judged by the Investigator to be of significant clinical impact
- Is a hematologic abnormality, including WBCs, hemoglobin, neutrophils, lymphocytes, and platelets that constitutes a change in grade from baseline.

If any abnormal laboratory result is considered clinically significant, the investigator will provide details about the action taken with respect to the test drug and about the subject's outcome.

7.2.2 Suspected adverse reaction

Suspected adverse reaction means any adverse event for which there is a reasonable possibility that the investigational therapy caused the adverse event. For the purposes of IND safety reporting, 'reasonable possibility' means there is evidence to suggest a causal relationship between the investigational therapy and the adverse event. A suspected adverse reaction implies a lesser degree of certainty about causality than adverse reaction, which means any adverse event caused by a drug.

7.2.3 Unexpected adverse reaction

An adverse event or suspected adverse reaction is considered "unexpected" if it is not listed in the protocol or informed consent documents or is not listed at the specificity or severity that has been observed; or is not consistent with the risk information described in the general investigational plan or elsewhere in the current application. "Unexpected" also refers to adverse events or suspected adverse reactions that are mentioned in the protocol or informed consent documents as occurring with a class of drugs or as anticipated from the pharmacological properties of the drug, but are not specifically mentioned as occurring with the particular drug under investigation.

7.2.4 Serious

An Unanticipated Problem or Protocol Deviation is serious if it meets the definition of a Serious Adverse Event or if it compromises the safety, welfare or rights of subjects or others.

7.2.5 Serious Adverse Event

An adverse event or suspected adverse reaction is considered serious if in the view of the investigator or the sponsor, it results in any of the following:

- Death,
- A life-threatening adverse drug experience
- In-patient hospitalization or prolongation of existing hospitalization
- Persistent or significant incapacity or substantial disruption of the ability to conduct normal life functions

- A congenital anomaly/birth defect.
- Important medical events that may not result in death, be life-threatening, or require hospitalization may be considered a serious adverse drug experience when, based upon appropriate medical judgment, they may jeopardize the subject or subject and may require medical or surgical intervention to prevent one of the outcomes listed in this definition.

7.2.6 Disability

A substantial disruption of a person's ability to conduct normal life functions.

7.2.7 Life-threatening adverse drug experience

Any adverse event or suspected adverse reaction that places the subject or subject, in the view of the investigator or sponsor, at immediate risk of death from the reaction as it occurred, i.e., it does not include a reaction that had it occurred in a more severe form, might have caused death.

7.2.8 Protocol Deviation

Any change, divergence, or departure from the IRB-approved research protocol.

7.2.9 Non-compliance

The failure to comply with applicable IRB requirements, FDA or other regulatory requirements for the protection of human research subjects.

7.2.10 Unanticipated Problem

Any incident, experience, or outcome that:

- Is unexpected in terms of nature, severity, or frequency in relation to

  (a) the research risks that are described in the IRB-approved research protocol and informed consent document, or other study documents, and

  (b) the characteristics of the subject population being studied; **AND**
- Is related or possibly related to participation in the research; **AND**
- Suggests that the research places subjects or others at a *greater risk of harm* (including physical, psychological, economic, or social harm) than was previously known or recognized.

7.3 ADVERSE EVENT REPORTING

Both Serious and Non-Serious Adverse Events will be clearly noted in source documentation and listed on study specific Case Report Forms (CRFs). The Principal Investigator or designee will assess each Adverse Event (AE) to determine whether it is unexpected according to the Informed Consent, Protocol Document, or related to the investigation. All Serious Adverse Events (SAEs) will be tracked until resolution and until 30 days after the last dose of the study treatment, whichever is later.

Adverse events will be recorded and reported as per section **7.2.1**.

In addition, any suspected serious adverse events that occur after 30 days, during the initial 5 years of follow up, will also be recorded and reported (section **5.5.7**). Suspected serious adverse events occurring in subjects who received cell therapy that are potentially related to the gene therapy nature of this study will be reported at the time of their occurrence during 15 year follow up (section **5.5.7**).

7.3.1 Stanford Reporting

Serious Adverse Events (SAEs) (as defined in section **7.2.4**) graded according to CTCAE v5.0 and Appendix B, section **13.2,** Grade 3 and above, and all subsequent follow-up reports will be reported to the Stanford Cancer Institute Data and Safety Monitoring Committee (DSMC) regardless of the event's relatedness to the investigation for institutional reporting purposes.

Events meeting the IRB definition of 'Unanticipated Problem' will be reported to the IRB using eProtocol within 10 working days of DSMC review, or within 5 working days for deaths or life-threatening experiences.

All SAEs Grade 3 and above attributed possibly, probably or definitely related to GD2CART, regardless of expectedness, will be reported to the APB using eProtocol within 10 working days of determination, or within 5 working days for deaths or life-threatening experiences.

APB and IRB will be notified via an expedited safety report if any stopping rules are triggered, as described in section **12.6**.

Non-serious AEs will be reported to IRB and APB at annual continuing review.

7.4 IND Sponsor Reporting Criteria

The Principal Investigator must **immediately** (within 24 hours of knowledge of event) report to the sponsor, using the Form FDA MedWatch 3500a or institutional SAE report form, any serious adverse event, whether or not considered drug related or expected, including those listed in the protocol or informed consent documents and must include an assessment of whether there is a reasonable possibility that the CAR T cell therapy caused the event. The Principal Investigator is responsible for promptly providing the Sponsor with any information needed to determine whether the SAE requires submission of an IND Safety Report.

Study endpoints that are serious adverse events (e.g. all-cause mortality) must be reported in accordance with the protocol unless there is evidence suggesting a causal relationship between the study therapy and the event (e.g. death from anaphylaxis). In that case, the investigator must immediately report the death to the sponsor.

Non-serious adverse events must be recorded in a timely manner and reported to Sponsor at IRB continuing review and in IND Annual Reports.

Events will be submitted to Sponsor Dr. Crystal Mackall, at:

Crystal Mackall, M.D.

Professor Pediatrics & Medicine

Associate Director, Stanford Cancer Institute

265 Campus Dr G3141A, MC5456

Stanford, CA 94305

650-725-9670

7.4.1 Reporting Pregnancy

✓ Maternal exposure

If a subject becomes pregnant during the course of the study (for 4 months after the last cell infusion and for as long as CAR cells are detectable in the blood), the study treatment should be discontinued immediately and the pregnancy reported to the Sponsor and the Stanford regulatory authorities. The potential risk of exposure of the fetus to the investigational agent(s) or chemotherapy agents (s) should be documented in box B5 of the MedWatch form "Describe Event or Problem".

Pregnancy itself is not regarded as an AE unless there is a suspicion that the study treatment under study may have interfered with the effectiveness of a contraceptive medication. However, as subjects who become pregnant on study risk intrauterine exposure of the fetus to agents which may be teratogenic, the Sponsor is requesting that pregnancy should be reported in an expedited manner as **Grade 3** "***Pregnancy, puerperium and perinatal conditions* - *Other (pregnancy)***" under the ***Pregnancy, puerperium and perinatal conditions*** SOC.

Congenital abnormalities or birth defects and spontaneous miscarriages should be reported and handled as SAEs. Elective abortions without complications should not be handled as AEs. The outcome of all pregnancies (spontaneous miscarriage, elective termination, ectopic pregnancy, normal birth, or congenital abnormality) should be followed up and documented.

If any pregnancy occurs in the course of the study, then the investigator should inform the Sponsor within 1 day, i.e., immediately, but **no later than 24 hours** of when he or she becomes aware of it.

The designated Sponsor representative will work with the investigator to ensure that all relevant information is provided to the Sponsor within 1 to 5 calendar days for SAEs and within 30 days for all other pregnancies.

The same timelines apply when outcome information is available.

✓ Paternal exposure

Male subjects should refrain from fathering a child or donating sperm during the study and for 4 months after the last dose of study treatment and for as long as CAR cells are detectable in the blood.

Pregnancy of the subject's partner is not considered to be an AE. However, the outcome of all pregnancies (spontaneous miscarriage, elective termination, ectopic pregnancy, normal birth, or congenital abnormality) occurring from the date of the first dose until 4 months after the last dose should, if possible, be followed up and documented.

7.5 FDA REPORTING CRITERIA

7.5.1 IND Safety Reports to the FDA (Refer to 21 CFR 312.32)

The Sponsor will be responsible for reporting to the FDA any unexpected fatal or life-threatening suspected adverse reactions as soon as possible but no later than 7 calendar days of initial receipt of the information using appropriate reporting format.

Additionally, any new Grade 3 neurotoxicity (not present at baseline and excluding cranial neuropathies and ataxia which are common in this disease) lasting longer than 96 hours will be submitted to FDA in an expedited fashion.

7.5.2 FDA Annual Reports (Refer to 21 CFR 312.33)

The Sponsor will submit the Annual Report to the FDA according to 21CFR§312.33, and any associated FDA correspondences regarding the IND annual report.

7.5.3 Serious Adverse Event Reporting on Cell Therapy Products to the FDA

A sample from all products that are non-conforming or do not meet release specifications will be used to conduct an out of specification investigation and the remainder either disposed of according to our facility biohazardous material disposal SOP or the FDA will be contacted by the manufacturing team to determine whether the product is suitable for infusion. The manufacturing facility will report all products manufactured including those that did not meet release criteria or were otherwise not infused in the annual IND report to the FDA.

All HCT/P deviations involving 351 cell products will be reported using MedWatch From FDA3500A according to FDA publication "Guidance for Industry: MedWatch Form FDA 3500A: Mandatory Reporting of Adverse Reactions Related to Human Cells, Tissues, and Cellular and Tissue-Based Products (HCT/Ps) available at: http://www.fda.gov/BiologicsBloodVaccines/GuidanceComplianceRegulatoryInformation/Guidances/Tissue/ucm074000.htm.

7.5.4 Action Plan for Positive Results on Cell Product Safety Testing:

In the unlikely event that a positive sterility test or mycoplasma test result is obtained after distribution of a cell product or after administration of the product to the subject, the following steps will be initiated IMMEDIATELY:

a. Stanford Director of Manufacturing and Process Development (or designee) will notify the IND Sponsor (Sponsor's Medical Representative) and principal investigator at 650-721-5750. Both will be updated with any substantive changes, including the final report on the identification and sensitivity from the positive sterility test. The Miltenyi QS personnel will determine the need for quality improvement based on the nature and extent of the incident.
b. If Stanford Center for Cancer Cell Therapy Director of Manufacturing and Process Development (or designee) is unable to reach Principal Investigator within 15 minutes, contact inpatient attending physician caring for the subject on the hospital service via hospital page with direct communication.. NOTE: The Sponsor (Sponsor's Medical Representative), Principal Investigator, and/or designee will contact the attending physician,

who will determine the extent of the work-up of a positive culture in consultation with appropriate infectious disease consultants, as well as determine an appropriate action treatment plan.

c. The Principal Investigator/attending physician will discuss the positive results with the subject, and specify the clinical therapy, antibiotic regimen and/or monitoring plan.

d. A contaminated sample of a product that has been administered to a subject will be handled in the same fashion as a Grade 4/5 toxicity. The Principal Investigator will be responsible for notifying the IRB and APB via an Unanticipated Problem (UP) report within 5 working days, and the Sponsor will notify the FDA via an expedited 7-day IND Safety Report.

In addition to the above, appropriate Safety reporting will be done as per SOP. A sample of each product is retained by Quality Systems and will be sent to the Microbiology Laboratory for repeat testing and speciation. An Out-of-Specification (OOS) Investigation will be conducted by the Quality Systems staff of the manufacturing laboratory including root cause analysis, review of viable environmental monitoring results collected at the time of manufacturing on personnel, equipment and reagents. Whether or not attribution is established, a formal Corrective and Preventive Action plan will be issued by the manufacturing staff and appropriate remediation will be performed including retraining of manufacturing personnel, elimination of any contaminated reagents and re-cleaning of the production facility followed by viable microbiological monitoring to establish effectiveness of cleaning.

8 CORRELATIVE/SPECIAL STUDIES

CAR T cell therapy targeting a single tumor associated antigen has mediated striking remissions in B cell leukemia and lymphoma/DLBCL. The clinical experience to date has demonstrated variable patterns of tumor remission and CAR T cell efficacy and persistence. In subjects treated with singular CD19 specific or CD22 specific CAR T cells, relapse patterns have included both respective CD19 or CD22 negative disease or relapsed disease with persistent, variable surface expression of the targeted antigen. The complex interplay of tumor, T cell and intrinsic CAR properties that influence these outcomes are not well understood. We aim to utilize this study as an opportunity to collect correlative data that will permit extensive study of cell compartments prior to and following CAR T cell therapy. We aim to integrate multi-dimensional technologies to permit complex analyses of the apheresis product, the CAR T cell product pre-infusion and *in vivo* expanded CAR T cells following antigen encounter. We additionally aim to investigate properties of DIPG tumors that render them resistant or susceptible to CAR T cell cytotoxicity.

Overall goal of study correlatives:

- Conduct analyses of the manufactured T cell product and blood and CSF post-infusion to identify biomarkers associated with enhanced CAR T cell expansion, persistence, and/or phenotype.
- Assess whether changes in the level of ctDNA in the cerebrospinal fluid can provide prognostic information and/or information regarding clonal evolution of DIPG over time.
- Evaluate whether antigen expression or tumor microenvironment are correlated with response to CAR T cell

Specific Aims:

1) CAR T cell correlatives:

- Measure expansion, persistence, and phenotype of adoptively transferred GD2CART in the CSF and blood and correlate with antitumor effects.
- Characterize CAR T cell functionality using cytokine analysis of patient samples during the period of highest likelihood of CAR expansion. Compare CSF to peripheral blood cytokine production for evaluation of functional effects of CAR in the CNS space.
- Where possible, use TCR sequencing to fate map cells contained in the manufactured CAR product to evaluate persistent GD2CART and identify subsets with a greater likelihood of T cell persistence in the setting of adoptive cell therapy.
- Evaluate chromatin structure and epigenomic modifications of CAR T cells using ATAC-seq and couple with RNA-seq for transcriptomic evaluation of CAR T cells.
- Assess the impact of T cell subset composition as delineated using flow cytometry, mass cytometry and ATAC-Seq on CAR T cell expansion, persistence, and phenotype.
- Compare persistence of GD2CART administered in this trial to that observed in a simultaneous trial that will administer GD2CART for pediatric solid tumors at similar doses and with the same lymphodepleting regimen.

2) Tumor correlatives:

- Assess whether changes in the level of ctDNA in the cerebrospinal fluid or peripheral blood can provide prognostic information and/or information regarding clonal evolution of DIPG before, during, and after CAR T cell therapy.
- Characterize antigen expression and evaluate tumor microenvironment on relapsed tumor tissue samples prior to and/or following CAR-mediated targeting

8.1 SAMPLE COLLECTION SCHEDULE

8.1.1 Study Correlatives

The samples to be collected and schedule for sample collection is detailed in section **13.7**, Appendix G.

8.1.2 Persistence of genetically engineered transduced cells

The procedures and methodologies for testing persistence of genetically engineered transduced cells are specified in section **13.7**.

If any subject has more than 5% persistence of gene transduced cells at Month 6 using 1A7, an anti-idiotype antibody that recognized the anti-GD2 CAR T cells, samples will be studied for clonality of persisting gene transduced cells. Such techniques may include analysis of BV chain expression, T cell cloning or LAM-PCR. If a predominant or monoclonal T cell clone derived from gene transduced cells is identified during the follow-up, the integration site and sequence will be identified and subsequently analyzed against human genome database to determine whether the sequences are associated with any known human cancers. If a predominant integration site is observed, the T cell cloning or LAM-PCR test will be used at an interval of no more than 3 months after the first

observation to see if the clone persists or is transient. In all instances where monoclonality is persistent and particularly in instances where there is expansion of the clone, regardless of whether or not the sequence is known to be associated with a known human cancer, the subject should be monitored closely for signs of malignancy, so that treatment, if available, may be initiated early.

8.2 Blood Drawing Limits for Research Purposes

8.2.1 Adults

The volume to be drawn per day for research purposes will be limited based on institutional policy. Subjects may undergo a small volume apheresis (approx. one to two blood volumes) in lieu of standard blood draw to obtain peripheral blood lymphocytes for correlative studies including Day 28 (± 1 week), and 3 (± 1 month), 6 (± 1 month), 9 (± 1 month), and 12 (± 2 months) months.

8.2.2 Pediatric Subjects:

The volume to be drawn per day for research purposes will be limited based on institutional policy. Subjects may undergo a small volume apheresis (approx. one to two blood volumes) in lieu of standard blood draw to obtain peripheral blood lymphocytes for correlative studies including Day 28, and 3 (± 14 days), 6 (± 14 days), 9 (± 14 days), and 12 (± 30 days) months. If the volume of blood exceeds that limit, the correlative studies will be prioritized as follows:

- CAR T correlatives/monitoring:
- PCR for RCR
- PCR for CAR
- Flow for CAR
- Cytof (T and B cell panels)
- TCR sequencing/single cell analysis
- Epigenomics/ATAC-Seq
- Circulating tumor DNA

9 STUDY CALENDAR

			Preparative Regimen and Cell Infusion			Post Treatment Assessment				Long Term Follow-Up			
**Procedure**	**Screening[a]**	**Apheresis (up to 48 hours prior, unless noted)**	**Day -4, Day -3, Day -2**	**Prior to cell infusion (< 24 hours, unless noted)**	**Day 0**	**Daily, Day 1-Day 14 (± 2 d)**	**Twice weekly, Day 15-Day 27 (± 4 d)**	**Day 28 (± 4 d)**	**Monthly, Month 2-3 (±2 weeks)**	**Month 6 (±1 mo.) 9, (±1 mo) 12 (±2 mo) q6-12 months to 5 years**	**Annually, Year 6 to 15 (± 3 mos)**	**At Disease Progression**	**Long Term Gene Therapy Follow Up (until Yr 15)**
History (at screening) and Physical Exam	X			X		X	X	X	X	X			
Vital signs	X	X	X	X	X[k]	X	X	X	X	X			
Neurologic exam	X			X		X[n]	X[n]	X	X	X			
Performance Status	X							X	X	X			
Height	X		X[c]										
Weight	X	X	X[c]	X				X					
ICP measure (Ommaya)				X[i]	X[i]	X[i]	X[i]	X[i]					
LP opening pressure					X[i]	X[i]	X[i]	X[i]					
Labs													
• CBC with diff	X	X[b]		X		X[m]	X[m]	X	X	X			
• PT/PTT	X												
• Chemistries[d]	X	X[b]	X[v]	X		X[m]	X[m]	X	X				
• Phosphorus	X	X[b]				X[m]	X[m]						
• Magnesium	X	X[b]		X		X[m]	X[m]						
• CRP	X	X[b]		X		X[m]	X[m]	X					
• Ferritin	X							X					
• Uric acid and LDH						X[m]	X[m]						
• HIV, HBsAg, HBsAb HBcAb, anti-HCV Ab		X[e]											
• Urinalysis	X			X									
• β-HCG pregnancy test on females of child-bearing potential	X	X[b]	X[b]										
• RCR				X[r]					X[r]	X[r]			
ECG	X												
ECHO, MUGA or cardiac MRI	X[f]												

			Preparative Regimen and Cell Infusion			Post Treatment Assessment				Long Term Follow-Up			
**Procedure**	**Screening[a]**	**Apheresis (up to 48 hours prior, unless noted)**	**Day -4, Day -3, Day -2**	**Prior to cell infusion (< 24 hours, unless noted)**	**Day 0**	**Daily, Day 1-Day 14 (± 2 d)**	**Twice weekly, Day 15-Day 27 (± 4 d)**	**Day 28 (± 4 d)**	**Monthly, Month 2-3 (±2 weeks)**	**Month 6 (±1 mo.) 9, (±1 mo) 12 (±2 mo) q6-12 months to 5 years**	**Annually, Year 6 to 15 (± 3 mos)**	**At Disease Progression**	**Long Term Gene Therapy Follow Up (until Yr 15)**
Correlative Research Studies	Correlative samples outlined in Section 13.7, Appendix G												
• Leukapheresis		X[g]						X[o]	X[o]	X[l]			
Disease Evaluation													
• H3K27M mutation documentation	X[q]												
• Clinical Evalution of Neurologic Status				X[w]		X[w]	X[w]	X[w]	X[w]				
• Imaging: Brain MRI or CT, MRI with and w/o gadolinium,	X[h]			X[h]		X[j]	X[j]	X[h,s]	X[h,s]	X[h,s]		X[h,]	
Treatment Regimen													
• Fludarabine			X[l]										
• Cyclophosphamide			X[l]										
• GD2CART infusion					X[p]								
Response Evaluation								X[h,s]	X[h,s]	X[h,s]		X[h,s]	
Adverse Events		X	X[t]	X[t]	X[t]	X[t]	X[t]	X[t]	X[t]	X[t]	X[t]	X[t]	X[t]
Concomitant Medications	X	X	X	X	X	X	X	X	X	X[u]	X[u]		X[u]
Long term follow up questionnaires										X[u]	X[u]		X[u]

a: Within 28 days, unless otherwise specified

b: Once within 7 days of leukapheresis or start of lymphodepleting chemotherapy

c: Day -4 to -6 only (± 5 days)

d: Laboratory evaluation to include; Chemistries: Sodium (Na), Potassium (K), Chloride (Cl), Total CO2 (bicarbonate), Creatinine, Glucose, Urea nitrogen (BUN), Albumin, Calcium total, Alkaline Phosphatase, ALT/GPT and/or AST/GOT, Bilirubin, Total Protein); creatinine clearance may be performed if the serum creatinine is elevated.

e: If collected on this study, within 28 days prior to leukapheresis. If cryopreserved from previous collection, within institutional standard time frame for autologous collection.

f: Testing performed within 180 days may be used for confirmation of eligibility

g: For cell acquisition for product development. This may be skipped if subject has cryopreserved cells that meet IND criteria.

h: Disease evaluations will be specific to the subject's location of disease: (brain MRI or MRI with and w/o gadolinium): Screening, once within 28 days prior to lymphodepleting chemotherapy (at investigator's discretion), Day 28 (±7 days) and Month 3 (± 1 month), 6 (± 1 month), 9 (± 1 month), 12 (± 2 months), then q6-12 (± 2 months) months. If subject's disease has not progressed by Month 24, disease assessments will continue at standard of care frequency

Agent: GD2.BB.z.iCasp9-chimeric antigen receptor (GD2 CAR) retroviral transduced autologous peripheral blood lymphocytes; following fludarabine and cyclophosphamide

i: Measurement of ICP via Ommaya Catheter may be performed at baseline (Day 0 prior to infusion), Day 3, Day 7, Day 10, Day 14, Day 21 and Day 28 or as needed based on investigator's discretion, and with evidence of increased ICP or clinical deterioration. Subjects with spinal DMG, opening pressure measurements via LP may be performed (optional unless clinically indicated): Day 7, Day 10 and Day 14, additional meaurements may be taken at Day 0, Day 21 and Day 28. If any evidence of toxicities that would benefit from a clear determination of pressure, additional LP measurements may be undertaken. This schedule may be altered to provide best clinical decisions and will not be considered protocol deviation.

j: Subjects with DIPG may undergo brain MRI on D7, D14, and D21 only as needed based on investigator discretion. If clinical condition post-infusion prevents MRI, a bedside CT will be obtained on those days.

k: Vital signs (blood pressure, heart rate, respiratory rate, pulse oximetry, temperature) prior to infusion, within 15 minutes after start of infusion (±10 min), and then 30 (±10 min), 60 (±10 min) and 120 (±10 min) minutes after infusion.

l: Fluid support and supportive medications as per section **5.3** and institutional procedures**.**

m: Daily until Day 7 (± 2 days) and then Day 14 (± 2 days) and then Day 21 (± 4 days).

n: Neurologic exam daily with documentation of ICANS Consensus Grading; ICE score / CAPD evaluated every shift (± 2 hours) (at least every 8 hours) and with any change in neurologic functioning until day 7, then daily until day 27 (± 4 days); neurologic exam with each visit after discharge.

o: Subjects may undergo a small volume apheresis (approx. one to two blood volumes) in lieu of standard blood draw to obtain peripheral blood lymphocytes for correlative studies including Day 28 (±7 days), and 3 (± 1 month) , 6 (± 1 month) , 9 (± 1 month) , and 12 (± 2 months) months.

p: Premedications will be provided as described in protocol. Subject may be offered additional CAR T cell treatments if enough cells were manufactured from the initial preparation, the subject experienced at least partial response to the first infusion, and the subject meets all eligibility criteria. Subsequent infusions will follow the same procedures as the first.

q: H3K27M mutation documentation requirement can be met anytime since diagnosis.

r: RCR obtained prior to cell infusion (may be obtained at any time prior to infusion), and at 3 (±1 month), 6 (± 1 month), and 12 months (± 2 months) post cell administration. Subsequent RCR blood samples will be stored annually x 5 years if RCR in first year negative.

s: If no response (i.e., progressive disease) by Day 28, then no further close clinical follow-up required and subject will be followed as per section **5.5.4.1**. If subject has SD, PR or CR monitor by physical exams, disease evaluations, vitals, CBC with diff, Chemistries until PD or subject proceeds to other therapies at investigator's discretion; at which time long-term follow-up will proceed as per section **5.5.4.1**

t: Adverse events will be collected and documented from the start of lymphodepletion chemotherapy and conclude 30 days after the last dose of study treatment. Prior to start of conditioning lymphodepletion chemotherapy, only unexpected serious adverse events considered related or possibly related to study procedures (i.e. leukapheresis) will be recorded and reported. Serious adverse events that occur more than 30 days after the last administration of investigational agent/intervention and have an attribution of at least possibly related to the agent/intervention should be recorded and reported

u: Gene Therapy Follow Up: Annual Physical exam, vital signs and performance status (may be performed by local physician) X 5 years (specifically document any new malignancies, new incidence or exacerbation of a pre-existing neurologic disorder, new incidence or exacerbation of a prior rheumatologic or other autoimmune disorder, new incidence of a hematologic disorder, targeted AEs and targeted con meds). After 5 years, health status data will be obtained from surviving subjects via telephone contact or mailed/emailed questionnaires. The long term follow up period for retroviral vectors is 15 years.

v: Electrolytes ONLY

w: Baseline (anytime before cell infusion), Day 7, D14, D21 and with clinical evaluations as deemed appropriate by the investigator.

.

10 MEASUREMENTS

10.1 OUTCOME MEASURES

10.1.1 Primary Objectives:

10.1.1.1 Determine the feasibility of manufacturing GD2CART for administration in children and young adults with H3K27M DIPG or spinal H3K27M mutated DMG using retroviral vector and dasatinib in the Miltenyi CliniMACS Prodigy® system.

Feasibility is defined by the rate of successful manufacture of the GD2CART produced with the Miltenyi CliniMACS Prodigy® system to satisfy the targeted dose level and meet the required release specifications.

10.1.1.2 Assess the safety and identify the maximum tolerated dose (MTD)/recommended phase 2 dose (RP2D) of GD2CART in subjects with H3K27M+ DIPG and in subjects with spinal H3K27M DMG administered after cyclophosphamide/fludarabine based lymphodepletion regimen using the following dose escalation schedule: DL1: 1e6 transduced T cells/kg; DL2: 3e6 transduced T cells/kg; DL3: 10e6 transduced T cells/kg. The MTD is defined as the dose level below that in which 2/6 subjects with experience DLTs during dose escalation.

10.1.1.3 Safety of GD2CART as evidenced by the incidence and severity of dose limiting toxicities (DLT), adverse events, serious adverse events, laboratory abnormalities, changes in vital signs, and changes in physical examination following infusion of GD2CART graded according to the Common Terminology Criteria for Adverse Events (CTCAE) Version 5.0 and Appendix B, section 13.2 in subjects with H3K27M DIPG and subjects with spinal H3K27M mutated DMG[7].

10.1.2 Secondary Objectives:

10.1.2.1 In a preliminary manner, assess clinical benefit of GD2CART at the RP2D in children and young adults with H3K27M DIPG and spinal H3K27M mutated DMG.

Clinical benefit will be measured by overall survival (OS) in subjects with DIPG and in subjects with DMG. In addition, post progression survival (PPS), progression free survival (PFS), time to progression (TTP), and radiographic and clinical response, including clinical evaluation of neurologic status change from baseline, will be evaluated.

10.1.2.1.1 Clinical Benefit Definitions

- Overall Survival (OS)

OS is defined as the time from the start of the lymphodepleting chemotherapy preparative regimen to the date of death from any cause.

- Post Progression Survival (PPS)

PPS is measured for each subject with DIPG as OS minus PFS, and for each patient with recorded progression as OS minus TTP[7].

- Progression Free Survival (PFS)

PFS is defined as the time from the start of the lymphodepleting chemotherapy preparative regimen to the date of radiographic progression or death from any cause.

- Time to Progression (TTP)

TTP is the time from the start of the lymphodepleting chemotherapy preparative regimen to the date of radiographic progression (death is censored).

10.1.2.1.2 Radiographic Tumor Response Criteria

Complete Response (CR)

Complete disappearance on MR of all evaluable tumor and mass effect, on a stable or decreasing dose of corticosteroids (or receiving only adrenal replacement doses), accompanied by a stable or improving neurologic examination. If CSF was positive, it must be negative.

Partial Response (PR)

Greater than or equal to 50% reduction in tumor size by bi-dimensional measurement, as compared with the baseline measurements, on a stable or decreasing dose of corticosteroids, accompanied by a stable or improving neurologic examination. *Axial T2/FLAIR images will be used for tumor measurements.*

Stable Disease (SD)

Neurologic exam is at least stable and maintenance corticosteroid dose not increased, and MR/CT imaging meets neither the criteria for PR nor the criteria for Progressive Disease

Progressive Disease (PD)

Progressive neurologic abnormalities or worsening neurologic status not explained by causes unrelated to tumor progression (e.g., anticonvulsant or corticosteroid toxicity wean, electrolyte disturbances, sepsis, hyperglycemia, etc.), OR a greater than 25% increase in the bi-dimensional measurement, taking as a reference the smallest disease measurement recorded since the start of protocol therapy, OR the appearance of a new tumor lesion.

Increasing doses of corticosteroids required to maintain stable neurological status should be strongly considered as a sign of clinical progression unless in the context of recent wean or transient neurologic change due e.g. to radiation effects.

10.1.2.1.1 Clinical Evaluation of Neurologic Status

Clinical evaluations as per **section 13.8** will be completed at least weekly during hospitalization, at Day 28 and then with every follow up visit until one of the following:

- Patient did not respond to treatment (i.e., did not achieve a CR, PR, or clinical benefit), or
- Patient progresses following a response, or
- Patient proceeds to alternative disease therapy.

Using the appropriate form, a study investigator will identify deficits at each visit and compare that visit to the baseline document to determine whether symptoms/signs have improved from baseline or worsened from baseline, or not changed. Total score will be calculated by the sum of all positive and negative points to determine if there has been an improvement in neurologic status post GD2CART therapy.

10.1.2.2 If unacceptable toxicity occurs that is possibly, probably or likely related to GD2CART, assess the capacity for AP1903, a dimerizing agent, to mediate clearance of the genetically engineered cells and resolve toxicity.

In the event of unacceptable toxicity (defined as grade 4 life threatening toxicity believed by the investigators to cause substantial risk to the subject) possibly, probably or definitely related to GD2CART, administration of AP1903 will eliminate the persistence of genetically engineered cells, and allow resolution of toxicity.

10.2 Exploratory Objectives

1. Measure expansion/persistence/phenotype of adoptively transferred GD2CART in the CSF and blood and correlate this with antitumor effects.
2. Conduct analyses of the manufactured T cell product and blood and CSF post-infusion to identify biomarkers associated with enhanced CAR T cell expansion, persistence and/or phenotype.
3. Assess whether changes in the level of ctDNA in the cerebrospinal fluid can provide prognostic information and/or information regarding clonal evolution of DIPG over time.
4. Evaluate whether antigen expression or tumor microenvironment are correlated with response to CAR T cell.

10.3 Institutional Review of Protocol

The protocol, the proposed informed consent and all forms of participant information related to the study (e.g. advertisements used to recruit participants) will be reviewed and approved by the Stanford IRB and Stanford Cancer Institute Scientific Review Committee (SRC). Any changes made to the protocol will be submitted as a modification and will be approved by the IRB prior to implementation. The Protocol Director will disseminate the protocol amendment information to all participating investigators.

10.4 Data and Safety Monitoring Plan

10.4.1 Clinical Team

The clinical research team will meet on a regular basis during dose escalation and when subjects are being actively treated on the trial to discuss cell manufacturing, toxicities, eligibility questions, trial accrual, and treatment needs. Decisions about dose level enrollment and dose de-escalation if applicable will be made based on the toxicity data from prior subjects in each disease group. Members from cell processing facilities will join as needed.

All data will be collected in a timely manner and reviewed by the principal investigator or a lead associate investigator. Adverse events will be reported as required above. Any safety concerns,

new information that might affect either the ethical and or scientific conduct of the trial, or protocol deviations will be immediately reported to the IRB, DSMC, APB and to the Sponsor.

The principal investigator will review adverse event and response data on each subject to ensure safety and data accuracy. The principal investigator will personally conduct or supervise the investigation and provide appropriate delegation of responsibilities to other members of the research staff.

10.4.2 Data Safety Monitoring Committee (DSMC)

The Stanford Cancer Center Data and Safety Monitoring Committee (DSMC) will be the monitoring entity for this study. The DSMC will audit study-related activities to determine whether the study has been conducted in accordance with the protocol, local standard operating procedures, FDA regulations, and Good Clinical Practice (GCP). In addition, the DSMC will regularly review serious adverse events, adverse events, and protocol deviations associated with the research to ensure the protection of human subjects. Results of the DSMC audit will be communicated to the IRB and APB and the appropriate regulatory authorities at the time of continuing review, or in an expedited fashion, as needed.

10.4.3 Safety Monitoring Committee (SMC)

The Center for Cancer Cell Therapy (CCT) has assembled an independent SMC consisting of Stanford and external academic investigators who are independent of the clinical trial under review and who are knowledgeable in cellular therapies to review all serious adverse events (SAEs), subject deviations, and internal and external audit/monitoring reports. The SMC will confirm dose escalation decisions based on available AEs and dose limiting toxicity (DLT) determinations for each dose cohort, and determine when the study needs to be halted based on protocol specified safety rules.

10.5 Data Management Plan

Case Report Forms (CRFs) are printed or electronic documents designed to record all protocol-related information on each trial participant. CRFs should summarize the clinical findings and observations necessary to ensure safety of participants on the study, and to document the study outcomes. Data will be entered into the Stanford database.

All data will be kept secure. Personal identifiers will not be used when collecting and storing data. Paper study files will be kept in a locked, secure location. Electronic study data will be stored in encrypted, backed-up, password protected computers. An enrollment log will be maintained in the regulatory binder/file which is the only location of personal identifiers with unique subject identification number.

11 COLLABORATIVE AGREEMENTS

Collaborative research agreement exists for conduct of correlative studies with Adaptive Biosciences, Inc. This collaborative agreement is disclosed in the informed consent document. A contract for manufacturing the cell product exists with Miltenyi Biotec Inc., which is disclosed in the informed consent document.

12 STATISTICAL CONSIDERATIONS

12.1 Statistical Design

A formal statistical analysis plan (SAP) will be prepared and finalized before database lock for the final analysis for the study report. The SAP will provide details regarding the definition of analysis subjects (populations), analysis variables, and analysis methodology to meet all study objectives.

The principle and key elements of the SAP are provided as follows:

- In general, safety and efficacy data will be summarized with descriptive statistics, including means, standard deviations, medians, minimums and maximums for continuous variables, the number of subjects and percent in each category for categorical variables.
- Data from each individual will be tabulated as appropriate. Efficacy and safety endpoints will be tabulated by dose cohort and time point.

The aims of this clinical trial are three-fold:

1. Determine the feasibility of manufacturing GD2CART for administration in subjects with H3K27M DIPG and subjects with spinal H3K27M DMG using a retroviral vector and dasatinib in the the Miltenyi CliniMACS Prodigy® system. The CliniMACS Prodigy® represents the next generation in automated cell processing, combining and streamlining cell processing workflows into one closed system.
2. Assess the safety and identify the maximum tolerated dose (MTD)/recommended phase 2 dose (RP2D) of GD2CART in subjects with H3K27M+ DIPG and in subjects with spinal H3K27M DMG administered after cyclophosphamide/fludarabine based lymphodepletion regimen using the following dose escalation schedule: DL1: 1 X 10^6 transduced T cells/kg; DL2: 3 X 10^6 transduced T cells/kg; DL3: 10 X 10^6 transduced T cells/kg.
3. In a preliminary manner, assess clinical activity of GD2CART at the RP2D in children and young adults with H3K27M DIPG or spinal H3K27M DMG.

The precedent for conducting clinical trials of this scope was established by the ongoing clinical trials CCT5001/IRB-41382 and CCT5007/IRB-41383 conducted by Stanford Center for Cancer Cell Therapy.

12.2 Primary Objectives:

- ✓ Determine the feasibility of manufacturing GD2CART for administration in subjects with H3K27M DIPG and subjects with spinal H3K27M DMG using a retroviral vector in the Miltenyi CliniMACS Prodigy® system.

Feasibility is defined by the rate of successful manufacture of the GD2CART produced with the Miltenyi Prodigy to satisfy the targeted dose level and meet the required release specifications.

- ✓ Assess the safety and identify the maximum tolerated dose (MTD)/recommended phase 2 dose (RP2D) of GD2CART in subjects with H3K27M+ DIPG and in subjects with spinal H3K27M DMG administered after cyclophosphamide/fludarabine based lymphodepletion regimen using the following dose escalation schedule: DL1: 1 X 10^6 transduced T cells/kg; DL2: 3 X 10^6 transduced T cells/kg; DL3: 10 X 10^6 transduced T cells/kg.

✓ Assess safety in children and young adults with spinal H3K27M DMG and in subjects with H3K27M DIPG administered GD2CART at MTD/RP2D after cyclophosphamide/fludarabine based lymphodepletion regimen.

The MTD is a dose level immediately below the level at which the enrollment is stopped due to DLT(s), as explained specifically below:

- If **more than one subject** in the first three subjects included in a dose level experience DLT as defined above, MTD will have been exceeded.
- If DLT develops in **one** of the 3 subjects included in a cohort, the cohort will be then expanded up to six:
- If 2 or more of these 6 included subjects develop DLT, the MTD will have been exceeded.

Safety of GD2CART as evidenced by the incidence and severity of dose limiting toxicities (DLT), adverse events, serious adverse events, laboratory abnormalities, changes in vital signs, and changes in physical examination following infusion of GD2CART at the recommended dose, recorded and graded according to the Common Terminology Criteria for Adverse Events (CTCAE) Version 5.0 and Appendix B: Guidelines Toxicity Assessment and Management **13.2**.

12.2.1 Feasibility

Feasibility will be defined as the successful manufacturing of GD2CART that meet established release criteria to satisfy the targeted dose level. Although we anticipate reaching the targeted cell dose during manufacture, feasibility of manufacturing cells remains a primary objective in this subject population. Subjects will be enrolled at the targeted dose level (until adequate subjects are enrolled to produce the correct number of cells for safety evaluation at that dose level) as long as 3 or more of the first 3 to 6 subjects in the targeted dose level are able to produce adequate cells for evaluation.

For example, this might mean that 6 to 9 subjects will need to be enrolled at a dose level to result in 6 for the safety evaluation. However, if less than 3 of 6 subjects at a given dose level are able to have adequate cells produced, evaluation of that level and beyond for safety and feasibility will not take place. If less than 3 of 6 subjects at dose level 1 are able to have adequate cells produced, enrollment will proceed to dose level -1. If less than 3 of 6 subjects are able to have adequate cells produced at dose level -1, enrollment to this study will stop. If cell growth limitations preclude administration of the targeted cohort cell dose, the subject will receive as many cells as possible, and be considered part of the lower dose cohort. If a minimum of 1.0×10^6 GD2CART per kg cannot be obtained for infusion, the subject may be treated but will not be evaluable for toxicity or response, but will be considered a feasibility failure.

If after the first 6 subjects have been enrolled at a given dose level, more than 3 are unable to have adequate GD2CART (that meet COA for infusion), accrual to that dose level will stop and the dose escalation phase of the study will also end, since the upper 90% one-sided confidence interval about 3/6 is 79.9%; thus, it would be unlikely that the true feasibility rate is 80% or greater for a given, which would be desirable. The evaluation of subjects in the expansion cohort will take place using the highest dose level at which feasibility, as well as safety, was identified. In the expansion

cohorts, the fraction which are able to manufacture the targeted dose level will also be monitored, and beginning with the 6th subject in an expansion cohort, if at any point fewer than half of the enrolled subjects are able to manufacture an acceptable level of cells, the accrual to the expansion cohort will end.

12.2.2 Determination of MTD/RP2D

Assess the safety of administering escalating doses of autologous GD2CART that can be feasibly produced to meet established release specifications in subjects with H3K27M DIPG and in subjects with spinal H3K27M DMG following a cyclophosphamide/fludarabine conditioning regimen. Dose escalation will proceed as outlined in **Table 3**

.

The endpoint for determination of MTD/RP2D in subjects is evidenced by the incidence and severity of dose limiting toxicities (DLTs) (i.e. laboratory abnormalities, changes in vital signs, and changes in physical examination) following chemotherapy preparative regimen and infusion of GD2CART, recorded and graded according to the Common Terminology Criteria for Adverse Events (CTCAE) Version 5 (and Appendix B: Guidelines Toxicity Assessment and Management, section **13.2**) at three dose levels until the maximum tolerated dose (MTD) is determined. If Dose level 3 can be feasibly manufactured and is administered in up to 6 subjects without evidence of DLT, this dose will be considered RP2D, or if no efficacy is observed, consideration will be given to amending the clinical trial to include additional dose levels.

The dose escalation procedure follows a 3+3 design beginning with dose level 1 in each disease group, based on the DLT count in each cohort, with escalation also constrained by the feasibility of producing the doses called for. The purpose of the design is not to invert the dose-toxicity curve at a target DLT rate, but rather to proceed with appropriate caution to dose level 3, which we expect will be reached without observing any DLT.

Because we do not anticipate the potentially life threatening intracranial complications of pontine DIPG in the subjects with spinal DMG, we propose to allow the safety of GD2CART in the pontine DIPG arm to inform the safety of dose escalation in spinal cord arm. Spinal cord DMG is rarer than pontine DIPG, so the spinal cord arm is expected to accrue more slowly and we anticipate fewer participants and may not accrue sufficiently to allow efficient identification of the RP2D. Hence, if the first subject with pontine DIPG receives dose level 1 of GD2CART and 28 days elapses without DLT, subsequent subjects with spinal DMG may receive GD2CART if 14 days elapse between subject infusions without DLT. Similarly, if 3 subjects with pontine DIPG complete 28 days of monitoring without DLT at dose level 1, the next subject with spinal DMG may enroll to dose level 2. The reverse of this rule will not apply to subjects with pontine DIPG given the unique nature of their potential risk for on-target, on-tumor effects in the pons.

Safety monitoring will continue throughout the study in the expanded cohorts of up to 20 subjects with pontine DMG and 10 subjects with spinal DMG at the final RP2D.

12.2.3 Safety assessment in Subjects with DIPG and Subjects with Spinal DMG (expansion cohorts)

As noted above, up to a total of 20 subjects with DIPG and 10 subjects with spinal DMG will receive the investigational regimen at MTD/RP2D.

The expansion cohort of subjects with DIPG treated at MTD/RP2D and the cohort of subjects with spinal DMG, including those evaluable during dose escalation, , the GD2CART dose will be decreased by one dose level if the tabulated threshold of adverse events that meet the criteria for Dose Limiting Toxicity (DLT) is met in either one of the expansion cohorts of subjects treated at RP2D.

- Specifications

  The maximum acceptable rate for DLT is 30%. We will perform safety monitoring to evaluate this DLT rate after 10 cell infusions in subjects with DIPG at the RP2D, and additionally after every 5 thereafter until we reach a total enrollment of 20 subjects with DIPG. The safety analyses are designed to reduce the dose if the observed rate at any evaluation is such that the lower one-sided 80% confidence limit (LCL80) exceeds the acceptable rate. We use the Wilson score method (Brown, Cai, and DasGupta 2001; Brown, Cai, and DasGupta 2002) to compute the LCL80.

  Based on these specifications, we will reduce the dose if the observed rate for DLT meets the criteria tabulated below for each level of enrollment. The actual LCL80 estimates show that the performance of this rule closely matches the maximum acceptable rate, and is not overly conservative.

  Dose De-escalation occurs if DLT meets these criteria

Number of DLTs	Current Enrollment	LCL 80
5	10	0.312
7	15	0.313
9	20	0.317

  In the event that dose de-escalation occurs, the new cohort treated at the lower dose will be monitored under the same rule, but starting again at 10 subjects treated at the lower dose for the first look. If the rule is triggered in the de-escalated cohort, enrollment will stop. In operation, the rule is implemented with the convention that if the number of DLTs reaches the critical level before the monitoring cohort is completely ascertained (so that the result is completely foreseeable), the de-escalation will be triggered in subjects with spinal DMG. So, for example, if there are 5 DLTs out of the first 8 subjects treated, the de-escalation will be triggered. The same rule applies to stopping in the de-escalated cohort, if appropriate.

12.3 SAFETY ENDPOINTS

All subjects who receive experimental treatment (GD2CART infusion) will be considered evaluable and will be analyzed for safety and efficacy.

Subjects not treated for any reason will be included in the disposition tabulation but will be considered inevaluable and excluded from the safety and efficacy analysis.

The safety and tolerability of GD2CART regimen will be assessed by:

- Suspected adverse events, and
- Suspected serious adverse events

As evidenced by:

- Changes in clinical laboratory tests (clinical chemistry, hematology, etc).
- Changes in vital signs (blood pressure, pulse, respiratory rate and body temperature).
- Changes in physical exams. Signs and symptoms assessed may require additional testing as clinically indicated such as ECG, PFT, radiographic studies, etc.
- Subject reported signs and symptoms

Safety data will be analyzed per standard methods and interpreted descriptively. Safety data will be summarized for each dose level separately and for the dose cohorts as a whole. Adverse events will be assessed using the CTCAE version 5.0 and Appendix B, **Section 13.2** for type and severity of event. Serious Adverse Events will be summarized for the targeted dose level. Reasons for discontinuation of study therapy will be tabulated.

Laboratory testing includes hematology, serology, serum chemistry, and urinalysis. Baseline laboratory testing will be those results obtained prior to initiating the conditioning lymphodepletion chemotherapy regimen. The study will utilize local lab for all clinical laboratory testing. Laboratory data will be tabulated based on the following result class.

- Normal: result is within the local lab normal range
- Abnormal: result is either higher or lower than the normal range

All abnormal values will be assessed for clinical significance; only clinically significant laboratory values will be captured in the case report form.

Vital signs collected immediately prior to receiving study drug will be the baseline vital signs. Observed vital sign values and change from baseline in vital signs at each visit will be summarized without formal statistical testing.

Vital sign result may also be tabulated based on the following result class.

- Normal: result is within the normal range
- Abnormal: result is either higher or lower than the normal range

All abnormal values will be assessed for clinical significance; clinical significance will be captured in the case report form. Number and percent of subjects within each result class will be tabulated by time point for each vital sign.

Findings of physical examinations will be tabulated by dose cohorts without formal statistical analysis.

12.4 Efficacy Endpoint

- ✓ In a preliminary manner, assess efficacy of GD2CART at the RP2D in children and young adults with H3K27M DIPG and children and young adults with spinal H3K27M DMG.

The main goal for the dose expansion portion of the trial is to collect data about safety and tolerability from a larger group of patients receiving therapy at RP2D. A secondary goal is to collect data about efficacy such as overall survival of children and young adults with H3K27M DIPG, in order to compute point, interval and quantile estimates. Any patients from the dose escalation cohort whose disease is amenable for response evaluation and who were treated at the

MTD/RP2D will be included in the expansion cohort numbers. Assuming 20% censoring and 10% loss-to-followup, enrollment of 20 evaluable patients with H3K27M DIPG would provide our study 80% power to detect a three-fold increase in median overall survival time (from 11 months to 33 months) with a 5% type I error rate. This is within both the total study time of 36 months (3 years) and the long-term follow-up time. It is possible that a lesser increase in median overall survival time would be adequate for future study of this regimen in this patient population, depending upon safety and feasibility. Thus the efficacy parameter will be regarded as a secondary objective for the trial.

The number of subjects with spinal DMG is not anticipated to be significant, hence the clinical activity in up to 10 subjects with spinal DMG receiving cell therapy at the RP2D will be reported separately and descriptively, including progression free survival (PFS) and overall survival (OS).

In addition, clinical response will be evaluated at Day 28, 3 months, and every 3 months up to 1 year, as per section **9**, in all subjects, until disease progression at which time subjects will be followed as per section **5.5.6** until off-study criteria are met.

12.5 Capacity for AP1903 to Mediate Clearance of Genetically Engineered Cells and Resolve Toxicity

Subjects who have unacceptable toxicity, defined as life-threatening/grade 4 toxicity believed by the investigators to cause substantial risk to the subject, which is possibly, probably or definitely related to the cellular therapy, that triggers administration of AP1903 will have the levels of CAR+ T cells in the blood measured at the time of AP1903 administration, then 1 hour, 2 hours, 4 hours, 8 hours, 24 hours and 48 hours after AP1903 dosing. The changes in CAR+ T cells will be reported descriptively. Patients will also be monitored clinically for changes in symptomatology using standard approaches and the changes will be documented and reported descriptively.

12.6 Protocol Stopping Rules

The study will be halted pending discussions with the FDA, IRB and APB if any of the following conditions are met:

a. Two DLTs occurs in dose -1 cohort in a subject group (subjects with DIPG or spinal DMG). If a DLT occurs in dose -1 cohort of one disease group, the other disease group may continue to enroll.

b. Development of EBV lymphoma or polyclonal lymphoproliferative disease (PLPD).

c. Any Grade 5 event at least possibly related to the research regimen.

d. If the targeted number of cells cannot be produced in 3 of the first 6 subjects or 5 of the first 10 subjects on this trial, further enrollment will be paused pending evaluation of the manufacturing process regardless of the safety evaluation.

12.7 Exploratory Analysis

- ✓ Measure expansion/persistence of adoptively transferred GD2CART in the CSF and blood and correlate this with antitumor effects.

- ✓ Conduct analyses of the manufactured T cell product and blood and CSF post-infusion to identify biomarkers associated with enhanced CAR T cell expansion, persistence and/or phenotype.
- ✓ Assess whether changes in the level of ctDNA in the cerebrospinal fluid can provide prognostic information and/or information regarding clonal evolution of DIPG over time.
- ✓ Evaluate whether antigen expression or tumor microenvironment are correlated with response to CAR T cell.

12.7.1 Persistence of GD2CART Analyses

- ✓ Measure expansion/persistence of adoptively transferred GD2CART in the CSF and blood and correlate this with antitumor effects.

Peripheral blood and CSF will be collected when available and separately analyzed for the presence of GD2CART. The percentage of all CD3+ cells in a sample that are positive by flow cytometry for GD2-CAR containing T cells will be analyzed and reported as time from T cell infusion. Correlation analyses will be performed to determine relationship to responders vs. non-responders.

12.7.2 Conduct analyses of the manufactured T cell product and blood and CSF post-infusion to identify biomarkers associated with enhanced CAR T cell expansion, persistence and/or phenotype.

Measurements of expansion and persistence in subjects with DIPG and DMG who have received GD2CART will be analyzed to identify biomarkers associated with expansion, persistence and/or phenotype. It will be difficult to draw conclusions from this analysis, but rather this analysis will be hypothesis generating.

12.7.3 Assess whether changes in the level of ctDNA in the cerebrospinal fluid can provide prognostic information and/or information regarding clonal evolution of DIPG over time.

12.7.4 Evaluate whether antigen expression or tumor microenvironment are correlated with response to CAR T cell.

This testing will provide an assessment of the tumor immune environment and will contribute to understanding mechanisms of tumor evasion through modulation of antigen density and/or a suppressive tumor environment.

12.8 Sample Size

12.8.1 Accrual estimates

We anticipate enrollment of 1-2 subjects per month during the dose escalation portion of this study, but expect to enroll 2 subjects per month during enrollment to the expansion cohorts, given the number of subjects with DIPG and DMG treated in the local/regional area. The recruitment period for this study is expected to be up to 3 years. Subjects will be followed for 1 year post treatment.

The total duration of this study to meet primary objectives is expected to be approximately 4 years of active treatment and short term follow up, and a total of 17 years of long term follow up after the last subject completes study therapy.

12.8.2 Sample size justification

The primary objectives of this study are safety and feasibility. Initially 3-6 evaluable subjects with H3K27M DIPG or spinal H3K27M DMG may be enrolled sequentially in 3 dose levels of GD2CART to establish MTD/RP2D, for a minimum of 8 subjects and a maximum of 36.

Once RP2D is established, up to a total of 20 evaluable subjects treated at MTD with DIPG and 10 evaluable subjects treated at MTD with spinal H3K27M DMG will be treated at the RP2D dose (including any from the dose escalation phase) to further assess safety and perform a preliminary analysis of clinical activity. In addition, we will allow for replacement of 6 total inevaluable subjects (subjects enrolled but who cannot receive cells, either due to physical deterioration or withdrawn consent during cell growth).

Thus, a maximum of 66 (36 in the dose escalation + 18 in the expansion cohorts + 6 replaced for manufacturing feasibility and 6 replaced for inevaluability following enrollment) subjects may be enrolled to determine the objectives of this study.

13 APPENDICES

13.1 APPENDIX A: Appendix A: Performance Status Criteria

**PERFORMANCE STATUS CRITERIA** *Karnofsky and Lansky performance scores are intended to be multiples of 10.*					
**ECOG (Zubrod)**		**Karnofsky**		**Lansky**	
**Score**	**Description**	**Score**	**Description**	**Score**	**Description**
0	Fully active, able to carry on all pre-disease performance without restriction.	100%	Normal, no complaints, no evidence of disease.	100%	Fully active, normal.
		90%	Able to carry on normal activity; minor sighs of symptoms of disease.	90%	Minor restrictions in physically strenuous activity.
1	Restricted in physically strenuous activity but ambulatory, able to carry out light or sedentary work, e.g., light housework, office work.	80%	Able to carry on normal activity with effort; some signs or symptoms of disease.	80%	Active, but tires more quickly.
		70%	Cares for self, unable to carry on normal activity or do active work.	70%	Both greater restriction of, and less time spent in, play activities.
2	Ambulatory and capable of all self-care but unable to carry out any work activities. Up and about more than 50% of waking hours.	60%	Requires occasional assistance but is able to care for most of own needs.	60%	Up and around, but minimal active play; keeps busy with quieter activities.
		50%	Requires considerable assistance and frequent medical care.	50%	Gets dressed, but lies around much of the day; no active play; able to participate in quiet play and activities.
3	Capable of only limited self-care, confined to bed or chair more than 50% of waking hours	40%	Disabled; requires special care and assistance.	40%	Mostly in bed; participates in quiet activities.
		30%	Severely disabled; hospitalization indicated, although death not imminent.	30%	In bed; needs assistance even for quiet play.
4	Completely disabled. Cannot carry on any self-care. Totally confined to a bed or chair	20%	Very ill; hospitalization necessary; active supportive treatment required.	20%	Often sleeping; play entirely limited to very passive activities.
		10%	Moribund, fatal process progressing rapidly	10%	No play; does not get out of bed
5	Dead	0%	Patient expired	0%	Unresponsive; Dead

Subjects who are unable to walk because of paralysis, but who are up in a wheelchair, will be considered ambulatory for the purpose of assessing the performance score for this study.

13.2 APPENDIX B: GUIDELINES TOXICITY ASSESSMENT AND MANAGEMENT

13.2.1 Guidelines for Grading Suspected Cytokine Release Syndrome according to ASTCT CRS Consensus Grading[52]#

CRS Parameter	Grade 1	Grade 2	Grade 3	Grade 4
**Fever**†	Temperature ≥ 38° C	Temperature ≥ 38° C	Temperature ≥ 38° C	Temperature ≥ 38° C

With either:

**Hypotension**	None	Not requiring vasopressors	Requiring one vasopressor with or without vasopressin	Requiring multiple vasopressors (excluding vasopressin)

And/or§:

**Hypoxia**	None	Requiring low-flow nasal cannula^ or blow-by	Requiring high-flow nasal cannula, facemask, non-rebreather mask, or Venturi mask	Requiring positive pressure (e.g.: CPAP, BiPAP, intubation and mechanical ventilation

CPAP: Continuous positive airway pressure; BiPAP: Bilevel positive airway pressure

# Organ toxicities associated with CRS may be graded according to CTCAE v5.0 but they do not influence CRS grading.

† Fever is defined as temperature ≥ 38° C not attributable fo any other cause. In patients who have CRS then receive anti-yretics or anti-cytokine therapy such as tocilizumab or steroids, fever is no long required to grade subsequent CRS severity. In this case, CRS grading is driven by hypotension and/or hypoxia.

§ CRS grade is determined by the more severe event: hypotension or hypoxia not attributable to any other cause. For example, a patient with a temperature of 39.5 ° C, hypotension requiring one vasopressor and hypoxia requiring low-flow nasal cannula is classified as having Grade 3 CRS.

^ Low-flow nasal cannula is defined as oxygen delivered at ≤ 6 liters/minute. Low flow also includes blow-by oxygen delivery, sometimes used in pediatrics. High-flow nasal cannula is defined as oxygen delivered at > 6 liters/minute.

13.2.2 Guidelines for Treating Suspected Cytokine Release Syndrome

Grade 1	*Symptoms are not life threatening and require symptomatic treatment only*<br>Treatment:<br>✓ Assess for infection<br>✓ Treat fever and neutropenia if present, monitor fluid balance, antipyretics, analgesics as needed.
Grade 2	*Symptoms require and respond to moderate intervention*<br>Treatment:<br>✓ As above for Grade 1<br>✓ Monitor organ function closely (incl. cardiac, respiratory, renal, neurologic, liver)<br>✓ Provide very limited bolus fluids only after discussion with Attending on Team C and the neuro-oncologist.<br>✓ If persistent hypotension, administer tocilizumab and anakinra (bolus 4 mg/kg [max 100 mg] over 1 hour, then 16 mg/kg over 24 hours by continuous IV)<br>✓ If no improvement within 24 hours, consider corticosteroids
Grade 3<br>or Grade 2 with co-morbidity	*Symptoms require and respond to aggressive intervention*<br>Treatment:<br>✓ As above for Grade 2<br>✓ Consider repeating tocilizumab and corticosteroids<br>✓ Consider vasopressors, oxygen supplementation
Grade 4	*Life-threatening symptoms*<br>Treatment:<br>✓ As above<br>✓ Positive pressure airway assistance or Mechanical ventilation<br>✓ Multiple vasopressors or high dose vasopressors

[1]high dose vasopressor doses shown in Table 2.

Tocilizumab is administered at a dose of 4-8 mg/kg infused over 1 hour (not to exceed 800 mg).

Table 2. High-Dose Vasopressors (all doses are required for ≥ 3 hours)

Norepinephrine monotherapy	≥ 0.2 mcg/kg/min
Dopamine monotherapy	≥ 10 mcg/kg/min
Phenylephrine monotherapy	≥ 200 mcg/min
Epinephrine monotherapy	≥ 0.1 mcg/kg/min
If on vasopressin	[1]Vasopressin + NE equivalent of ≥ 0.1mcg/kg/min
If on combination vasopressors (not vasopressin)	[1]Norepinephrine equivalent of ≥ 20 mcg/kg/min

[1]*VASST Trial Vasopressor Equivalent Equation:*

Norepinephrine equivalent dose = [norepinephrine (mcg/min)] + [dopamine (mcg/kg/min) ÷ 2] + [epinephrine (mcg/min)] + [phenylephrine (mcg/min) ÷10]

13.2.3 Encephalopathy assessment tools for grading Immune effector Cell-Associated Neurotoxicity Syndrome (ICANS)

13.2.3.1 Immune effector Cell-associated Encephalopathy (ICE) Assessment

Directions: Answer whether each task was performed correctly (Not Done/Yes/No). If you answer YES put 1 in the Score column; If you answer NO (or Not Done) put 0 in the Score column.

Tasks	Performed correctly?	Score
**Orientation**		
1. What is the current year?		**0**
2. What is the current month?		**0**
3. What is the current city?		**0**
4. What hospital are you in?		**0**
**Naming**		
5. Name this object (*point to an object in the room)*		**0**
6. Name this object (*point to an object in the room)*		**0**
7. Name this object (*point to an object in the room)*		**0**
**Following commands**		
8. Show me (insert object, e.g. 2 fingers) or Close your eyes and stick out your tongue.		**0**
**Writing**		
9. Write a simple sentence (*provide paper and pencil)*		**0**
**Attention**		
10. Count backwards from 100 in 10's.		**0**
**Total Score**		**0**

Scoring ICE

No impairment: Score 10

Grade 1 ICANS: Score 7-9

Grade 2 ICANS: Score 3-6

Grade 3 ICANS: Score 0-2

Grade 4 ICANS: Score 0 due to patient unarousable and unable to perform ICE assessment

13.2.3.2 ASBMT Immune effector Cell-Associated Neurotoxicity Syndrome (ICANS) Consensus Grading for Adults

Neurotoxicity Domain	Grade 1	Grade 2	Grade 3	Grade 4
**ICE Score^**	7-9	3-6	0-2	0 (patient is unarousable and unable to perform ICE)
**Depressed level of consciousness⁑**	Awakens spontaneously	Awakens to voice	Awakens only to tactile stimulus	Patient is unarousable or requires vigorous or repetitive tactile stimuli to arouse. Stupor or coma
**Seizure**	N/A	N/A	Any clinical seizure focal or generalized that resolves rapidly; or<br>Non-convulsive seizures on EEG that resolve with intervention	Life-threatening prolonged seizure (> 5 min); or<br>Repetitive clinical or electrical seizures without return to baseline in between.
**Motor findings§**	N/A	N/A	N/A	Deep focal motor weakness such as hemiparesis or paraparesis
**Raised ICP / Cerebral edema**	N/A	N/A	Focal/local edema on neuroimaging#	Diffuse cerebral edema on neuroimaging;<br>Decerebrate or decorticate posturing; or<br>Cranial nerve VI palsy; or<br>Papilledema; or<br>Cushing's triad

ICANS grade is determined by the most severe event (ICE score, level of consciousness, seizure, motor findings, raised ICP/cerebral edema) not attributable to any other cause. For example, a patient with and ICE score of 3 who has a generalized seizure is classified as having a Grade 3 ICANS.

^A patient with an ICE score of 0 may be classified as having Grade 3 ICANS if the patient is awake with global aphasia. But a patient with an ICE score of 0 may be classified as having a Grade 4 ICANS if the patient is unarousable.

⁑Depressed level of consciousness should be attributable to no other cause (e.g. no sedating medication).

§Tremors and myoclonus associated with immune effector cell therapies may be graded according to CTCAE v5.0 but they do not influence ICANS grading.

#Intracranial hemorrhage with or without associated edema is not considered a neurotoxicity feature and is excluded from ICANS grading. It may be graded according to CTCAE v5.0.

ICE: Immune effector Cell-associated Encephalopathy; **ICP:** Intracranial pressure; **EEG:** electroencephalogram

13.2.3.3 Encephalopathy Assessment for Children < 12 years using Cornell Assessment of Pediatric Delirium **(CAPD)**[67],[68]

Adapted from Taube et al.[67] and reproduced in [52] with permission from Wolters Kluwer.

**Answer the following based on interactions with the child over the course of the shift**	**Never**<br>**4**	**Rarely**<br>**3**	**Sometimes**<br>**2**	**Often**<br>**1**	**Always**<br>**0**
1. Does the child make eye contact with the caregiver?					
2. Are the child's actions purposeful?					
3. Is the child aware of his/her surroundings?					
4. Does the child communicate needs and wants?					
	**Never**<br>**0**	**Rarely**<br>**1**	**Sometimes**<br>**2**	**Often**<br>**3**	**Always**<br>**4**
5. Is the child restless?					
6. Is the child able to be consoled??					
7. Is the child underactive-very little movement while awake?					
8. Does it take the child a long time to respond to interactions?					

For patients age 1-2 year, the following serve as guidelines to the corresponding questions:

1. Holds gaze. Prefers primary parent. Looks at speaker.
2. Reaches and manipulates objects, tries to change position, if mobile may try to get up.
3. Prefers primary parent, upset when separated from preferred caregivers. Comforted by familiar objects (i.e., blanket or stuffed animal)
4. Uses single words or signs
5. No sustained calm state
6. Not soothed by usual comforting actions, for example, singing, holding, talking, and reading
7. Little if any play, efforts to sit up, pull up, and if mobile crawl or walk around
8. Not following simple directions. If verbal, not engaging in simple dialogue with words or jargon.

13.2.3.4 ASTCT Immune effector Cell-Associated Neurotoxicity Syndrome (ICANS) Consensus Grading for Children

Neurotoxicity Domain	Grade 1	Grade 2	Grade 3	Grade 4
**ICE Score for children > 12 years^**	7-9	3-6	02	0 (patient is unarousable and unable to perform ICE)
**CAPD score for children ≤ 12 years**	<9	<9	≥9	Unable to perform CAPD
**Depressed level of consciousness⁑**	Awakens spontaneously	Awakens to voice	Awakens only to tactile stimulus	Patient is unarousable or requires vigorous or repetitive tactile stimuli to arouse. Stupor or coma
**Seizure (any age)**	N/A	N/A	Any clinical seizure focal or generalized that resolves rapidly; or<br>Non-convulsive seizures on EEG that resolve with intervention	Life-threatening prolonged seizure (> 5 min); or<br>Repetitive clinical or electrical seizures without return to baseline in between.
**Motor weakness (any age)§**	N/A	N/A	N/A	Deep focal motor weakness such as hemiparesis or paraparesis
**Raised ICP / Cerebral edema (any age)**	N/A	N/A	Focal/local edema on neuroimaging#	Decerebrate or decorticate posturing; or<br>Cranial nerve VI palsy; or<br>Papilledema; or<br>Cushing's triad; or<br>Signs of diffuse cerebral edema on neuroimaging

ICANS grade is determined by the most severe event (ICE or CAPD score, level of consciousness, seizure, motor findings, raised ICP/cerebral edema) not attributable to any other cause.

^A patient with an ICE score of 0 may be classified as having Grade 3 ICANS if the patient is awake with global aphasia. But a patient with an ICE score of 0 may be classified as having a Grade 4 ICANS if the patient is unarousable.

⁑Depressed level of consciousness should be attributable to no other cause (e.g. no sedating medication).

§Tremors and myoclonus associated with immune effector cell therapies may be graded according to CTCAE v5.0 but they do not influence ICANS grading.

#Intracranial hemorrhage with or without associated edema is not considered a neurotoxicity feature and is excluded from ICANS grading. It may be graded according to CTCAE v5.0.

ICE: **I**mmune effector **C**ell-associated **E**ncephalopathy; **CAPD**: **C**ornell **A**ssessment of **P**ediatric **D**elirium; **ICP:** **I**ntra**c**ranial **p**ressure; **EEG**: **e**lectro**e**ncephalo**g**ram

13.2.4 Protocol for managing neurologic symptoms in patients with pontine DIPG receiving GD2CART

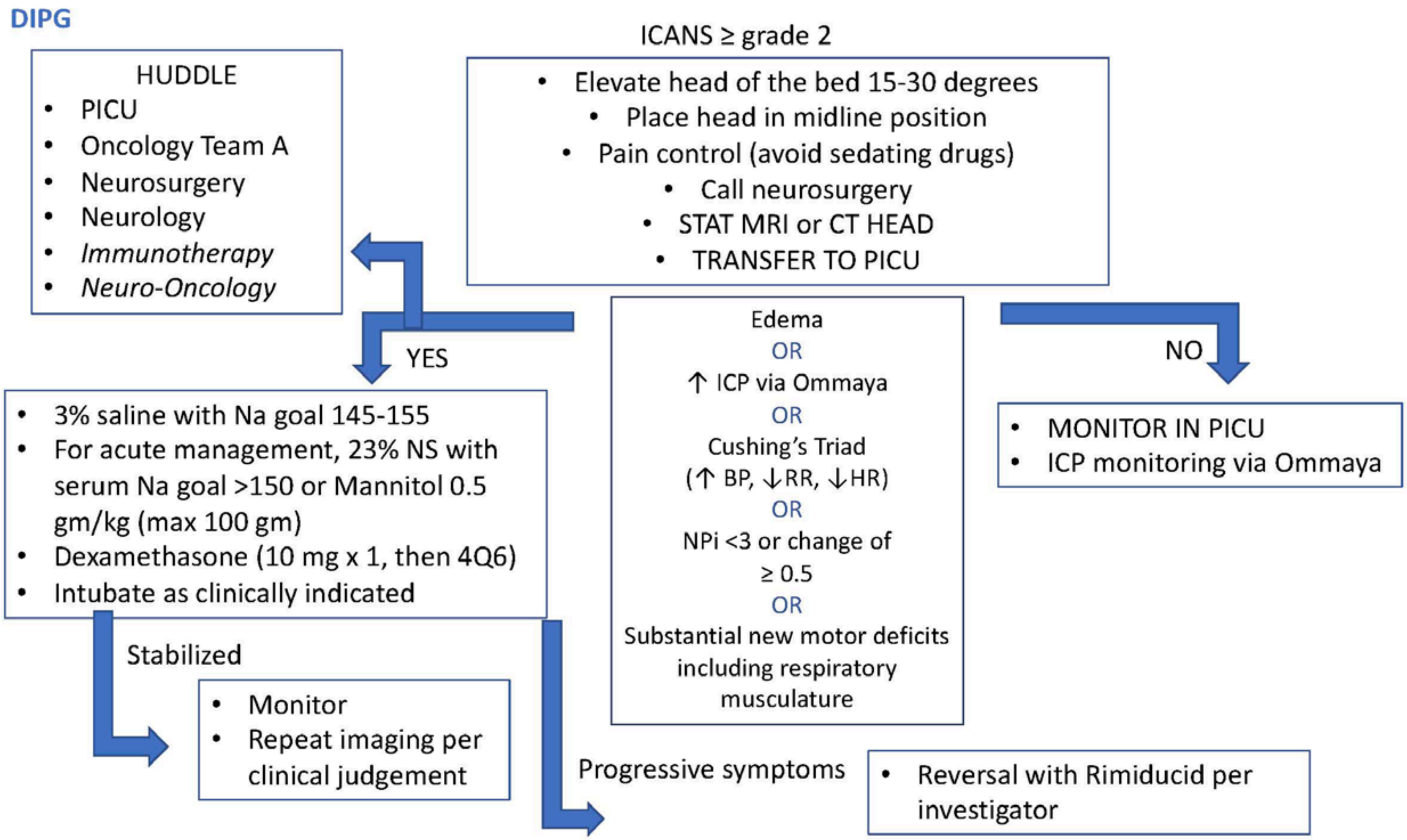

13.2.5 Protocol for managing neurologic symptoms in patients with spinal DMG receiving GD2CART

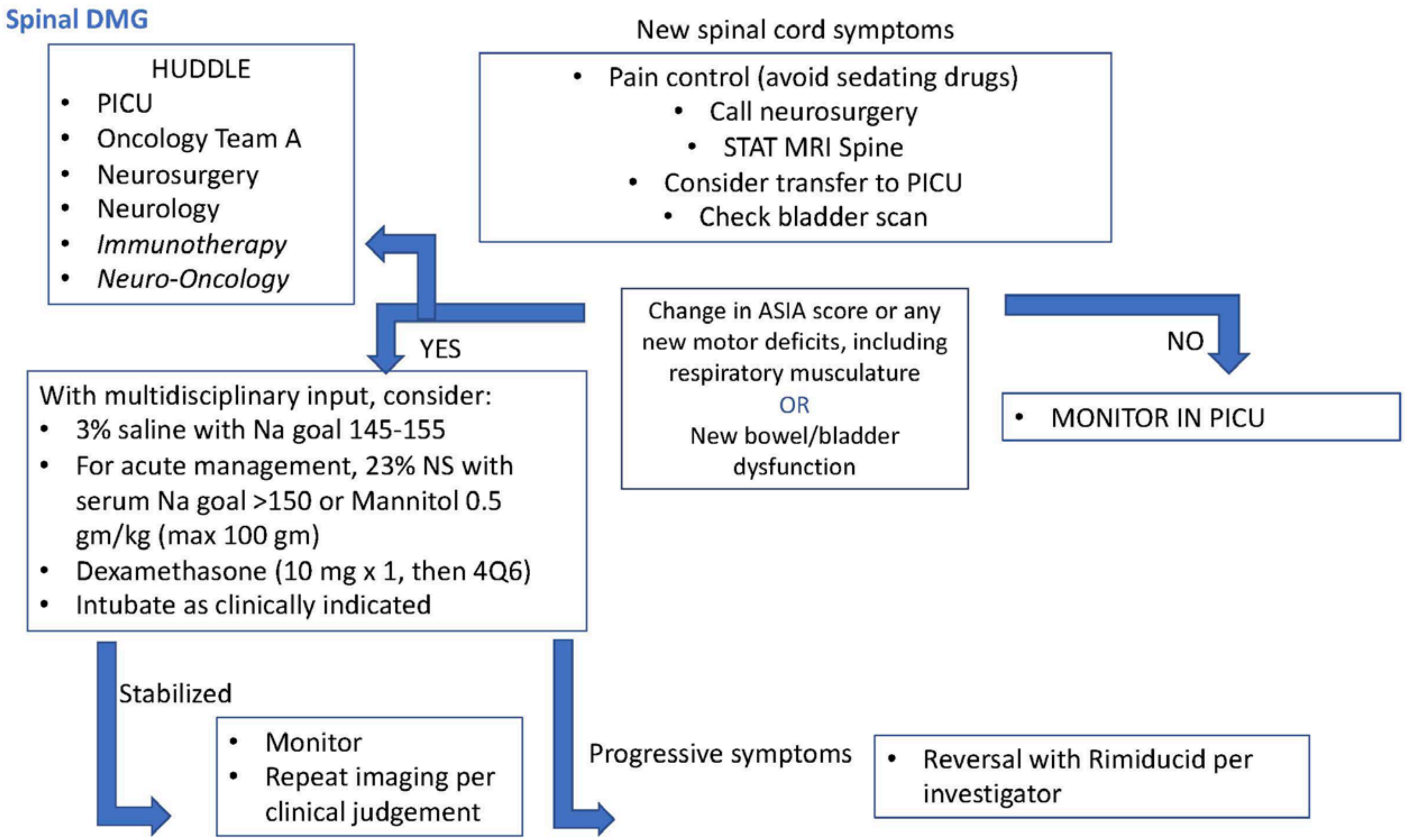

13.2.5.1 Spinal cord assessment

Spinal cord function in patients with spinal DMG should be conducted with neurologic exams according to American Spinal Injury Association (ASIA) International standards for neurological classification of spinal cord injury ASIA Impairment Scale (https://asia-spinalinjury.org/wp-content/uploads/2016/02/International_Stds_Diagram_Worksheet.pdf).

American Spinal Injury Association (ASIA) score

- A = Complete: No sensory or motor function is preserved in sacral segments S4-S5
- B = Incomplete: Sensory, but not motor, function is preserved below the neurologic level and extends through sacral segments S4-S5
- C = Incomplete: Motor function is preserved below the neurologic level, and most key muscles below the neurologic level have a muscle grade of less than 3
- D = Incomplete: Motor function is preserved below the neurologic level, and most key muscles below the neurologic level have a muscle grade that is greater than or equal to 3
- E = Normal: Sensory and motor functions are normal

13.3 Appendix C: Calculation of Weight for Cell Dose Calculation in Morbidly Obese Candidates

Formulation for deriving the weight to be used in targeting cell doses in morbidly obese cell candidates.

1. **Definition**

Obesity is defined as a BMI > 30.

BMI = wgt (kg) / [hgt (M)]2

2. **Calculation of ideal body weight** is performed using the standard, published formula:

Male: 50 + 2.3(Hgt - 60) where Hgt is in inches, and the result is expressed in kg.

ex. The ideal weight of a 5'10" male = 50 + 2.3(10) = 73 kg.

Female: 45.5 + 2.3(Hgt - 60), where height is in inches, and the result is in kg.

3. **Calculation of the "practical weight."**

Calculate the midway point, halfway between the actual and ideal body weights (ie the average of the two numbers). This is the "practical weight" to be used in calculating the targeted cell dose.

4. **Example**:

Subject's actual weight = 143 kg.

Subject' s actual height 173 cm = 69 in

BMI = 48

IBW formula = 50 + 2.3(9) = 70.7 kg

Midway point between 70.0 and 143 = 107 kg.

The weight we would use in targeting cell dose is 107 kg.

1 dose by weight with adjustment:

- IBW + 50%(Weight-IBW)
- Practical body weight = (IDW + actual BW)/2

2 Formula

IBW (men)

- 52 kg + 1.9 kg/inch above 5 feet
- 50 kg + 2.39 (height in inches – 60)

IBW (women)

- 49 kg + 1.7 kg/inch above 5 feet
- 45.5 kg + 2.39 (height in inches – 60)

13.4 Appendix D: Monitoring Gene Therapy Trials: Replication Competent Retrovirus (RCR)

Subject Testing for RCR PCR

Subject blood samples will be obtained at the following time points:

1. Prior to cell infusion
2. 3 months(± 2 weeks)
3. 6 months (± 1 month)
4. 1 year (±2 months)
5. Archive samples annually thereafter for 5 years (+/- 3 months), if previous samples have been negative.

All Samples are scheduled from the most recent cell infusion.

Procedures:

Samples will be collected from subjects returning to clinic or collected by a local physician and shipped and sent to Indiana University according to the Laboratory Manual.

Document Retention

1. RCR Reports from Indiana University will be sent to the Sponsor Investigator; the study coordinator will obtain the report from the study PI.
2. Scan the report and save results of RCR PCR (or S+L-) testing in a password protected subject file associated with the gene therapy protocol. Hard copies will be retained in the subject's research record in a locked file cabinet.
3. Record the sample result on the Master S+L-/RCR PCR Spreadsheet saved in the study files.

13.5 Appendix E: Draft Letter and Questionnaire to Subjects for Long Term Follow-up for Delayed Adverse Events

[date]

[name and address]

Dear [subject name],

You have participated in a clinical research study that requires that the study doctors and nurses monitor your health for 15 years. In addition to the annual visits you will be attending, **we would like for you to report certain events listed below to your study doctor or nurse if they occur**:

1. Your doctor tells you that you have been diagnosed with any new type of cancer, including blood disorders such as leukemia or lymphoma (this would be separate from your cancer diagnosis).
2. You develop loss of feeling in any part of your body, especially hands and feet; you develop a loss of control of any body part (arms, legs…); you have a seizure; you experience memory loss. In addition, if you experience a worsening of any of the symptoms listed, please contact your study nurse or doctor. These types of symptoms are called neurological disorders. If your primary doctor or specialist tells you that you have developed neurological symptoms, contact your study doctor or nurse.
3. You develop arthritis or autoimmune disease, or worsening of any previously experienced arthritis or autoimmune disease which you were experiencing prior to participation in the study. If you are experiencing symptoms of arthritis or have been told by your doctor that you have an autoimmune disease, contact your study doctor or nurse.

Please complete the attached questionnaire and return it in the Fed-Ex envelope to the study coordinator.

If you experience any of the events listed above during the upcoming year, please contact your study physician or the study nurse listed below as soon as you can. They may ask you questions about your health and will record your symptoms/disease and then monitor your health if they decide that it is necessary. When you call, please mention that you participated in a gene therapy clinical trial at the < put your institution here> . Your subject identification number under this protocol is (#XXX).

Study Coordinator:

Name
Address
Phone
Email

If you have any questions about this letter or the follow up procedures for the study itself, please do not hesitate to contact the above study nurse.

Thank you for your continued participation in our clinical research study. Best regards,

[study coordinator]

Questionnaire to Subjects for Long Term Follow-up for Delayed Adverse Events

Subject Identification [put subject study number here]

Within the past year, have you:

1. **Had any problems with your health?** ☐ YES ☐ NO

If Yes, please explain: ______________________________

____

2. **Required any hospitalizations?** ☐ YES ☐ NO

If Yes, please describe when and the reason: ______________________________

____

____

3. **Seen any healthcare provider?** ☐ YES ☐ NO

If Yes, please describe when and the reason: ______________________________

____

____

4. **Started on any new medications?** ☐ YES ☐ NO

If Yes, please list: ______________________________

____

5. **Developed any new conditions or illnesses?** ☐ YES ☐ NO

If Yes, please describe: ______________________________

____

Please share any other new health concerns or problems:

____

When you have completed this questionnaire, please return it to:

Study Coordinator:

Name
Address
Phone
Fax
Email

A pre-addressed stamped envelope has been enclosed for your convenience, if you choose to mail this questionnaire. We will also accept faxed or e-mailed completed questionnaires as well.

Thank you very much for your participation.

13.6 Appendix F: Physician (Local Medical Provider) Letter

[date]

[name and address]

Dear [physician name],

Your subject [subject name] has participated in a clinical research study that requires 15 year monitoring for adverse events. To aid in reporting adverse events that are possible related to the clinical research study, we are asking the subjects on our research study to designate a primary care or infectious disease physician that may help in the monitoring and reporting of adverse events. Your subject has designated you. **If upon any of your visits with your subject, any of the following events are reported or discovered, please contact the study nurse or physician as soon as possible**:

1. New malignancies
2. New incidence of exacerbation of a pre-existing neurologic disorder
3. New incidence or exacerbation of a prior rheumatologic or other autoimmune disorder
4. New incidence of a hematologic disorder.

If your subject experiences any of these events, please contact the study coordinator below as soon as you can so that they can record the event and then monitor your subject's health if necessary. When you call, please mention that the subject has participated in a gene therapy clinical trial in the < designate location, institution and sponsor investigator> .

Study Coordinator

Name

Address

Phone

Email

If you have any questions about this letter or the study itself, please do not hesitate to contact the above study nurse.

Thank you for your support in helping us to monitor for delayed adverse events. Best regards,

13.7 APPENDIX G: CORRELATIVE SAMPLE SCHEDULE

Priority	mL	type	purpose	Apheresis	Product	Baseline (prior to LD chemo)	D0	D3 (± 2 d)	D7 (± 2 d)	D10 (± 2 d)	D14 (± 2 d)	D17 (± 4 d)	D21 (± 4 d)	D24 (± 4 d)	D28 (± 7 d)	M2 (± 2 w)	M3 (± 1 m)	M4 (± 2 w)	M5 (± 2 w)	M6 (± 1 m)	M9 (± 1 m)	M12 (± 2 m)	At Progression or Death	Minimum Cell
		**Apheresis Product**																						
		collected by manufacturer		X																				
		collected by Stanford													X[1]		X[1]			X[1]	X[1]	X[1]		
		**CAR T cell Product**																						
		collected by manufacturer			X																			
		**PBMCs**																						
1	4	Green top (Heparin)	HiD CAR FACS panel[4]		X		X	X	X	X	X	X	X	X	X	X	X	X	X	X	X	X	X	2 x10^{5}
2	5	Lavender top (EDTA)	CAR qPCR		X		X	X	X	X	X	X	X	X	X	X	X	X	X	X	X	X	X	
3	5	Green top (Heparin)	CyTOF	X	X	X			X		X[2]		X		X[2]	X	X[2]	X	X	X[2]	X	X	X[2]	4-5 x10^{5}
4	5	Lavender top (EDTA)	TCR sequencing/ CAR-T cell fate	X	X						X				X		X			X	X	X	X	
5	5	Lavender top (EDTA)	ATAC-Seq/RNA-Seq	X	X						X				X		X			X	X	X	X	
7	5	Green top (Heparin)	Sample banking			X	X		X		X		X		X	X	X	X	X	X	X	X	X	
8	5	Lavender top (EDTA)	Sample banking			X	X		X		X		X		X	X	X	X	X	X	X	X	X	
		**Plasma**																						
9	5	Streck tube	Cell-free DNA[8]			X					X				X		X			X	X	X	X	
		Lavender top (EDTA)	Cytokines[3,4]			X		X	X	X	X	X	X	X	X									
		Lavender top (EDTA)	Sample banking[3]			X	X		X		X		X		X	X	X	X	X	X	X	X	X	
		**CSF**																						
1		Falcon tube	PCR (tumor & CAR)					X	ł	X		X	ł	X		X[6]	X[6]	X[6]	X[6]	X[6]	X[6]	X[6]	X[6]	
1	5	Falcon tube	HiD CAR FACS panel[4]					X	ł	X		X	ł	X		X[6]	X[6]						X[6]	
2	5	Falcon tube	scRNAseq		X	X,ł			X		X,ł		X		X,ł									
3		Falcon tube	Cytokines[4,7]			X,ł	X	X	X,ł	X	X,ł	X	X,ł	X	X,ł								X[6]	
4		Falcon tube	Cell-free DNA[7]			X,ł			ł		X,ł		ł		X,ł	X[6]	X[6]	X[6]	X[6]	X[6]	X[6]	X[6]	X[6]	
5	5	Falcon tube	Sample banking				X																	
		**Tumor**																						
1		FFPE	Sample banking			X[6]																	X[6]	
2		Frozen Tissue	Sample banking			X[6]																	X[6]	
Total Blood Volume per visit (mL)																								
						24	14	9	19	9	39	9	19	9	39	19	39	19	19	39	34	34	39	

Notes

1	A small volume apheresis (1-2 blood volumes) may be collected in lieu of peripheral blood tubes listed below
2	If possible, collect 10 mL
3	Plasma separated from Lavendar top (EDTA) collection tube
4	Collect at any time of neurotoxicity or ommaya access, at discretion of investigator
5	If patient sample volume limits are reached, collection tubes may be volume reduced and/or pooled and aliquoted based on minimum cell number requirements and the correlative priority number
6	When available, plan to collect these samples
7	To be isolated from supernatant of CSF sample
8	Collect 10 ml, whenever able, minimum must be 5ml
ł	For spinal DMG patients, collect these timepoints via lumbar puncture, when possible

If CSF samples are collected on patients who are not being seen at Stanford, CSF samples should be placed in EDTA tubes for shipment to Stanford.

Target sample volumes are ideal, and may be adjusted for age, size and condition of patient. If blood volumes are limited, testing completion will be prioritized according to **Section 8.2.2.**

13.8 APPENDIX H: CLINICAL EVALUATION OF NEUROLOGIC STATUS

Stanford
Cancer Institute

Patient ID: Click here to enter text.

FORM: Clinical Evaluation of Neurologic Status-DIPG (pontine DMG)

DATE of ASSESSMENT (MM/DD/YYYY): Click here to enter a date. **DAY POST CAR INFUSION:** ______

Time of Assessment: : ☐ am ☐ pm

Name of Person Conducting the Assessment: ______

Instructions: Document deficits in "Details" column at baseline. Use Baseline Form as reference for completing the following table at the protocol specified timepoints. Assign 1 point for symptoms/signs that are improved from baseline, and -1 points for symptoms/signs that are worse than baseline. This form should be used for subjects who are not on an increased dose of steroids.

	Worse (-1)	Same (0)	Better (+1)	No deficit at baseline (0)	details
R Third nerve palsy					
L Third nerve palsy					
R Fourth nerve palsy					
L Fourth nerve palsy					
R Sixth nerve palsy					
L Sixth nerve palsy					
R Facial sensation					
L Facial sensation					
Muscles of mastication (trismus)					
R Seventh nerve palsy/facial droop					
L Seventh nerve palsy/facial droop					
R hearing					
L hearing					
Palate symmetrical					
Speech articulation					
Tongue alignment					
Swallowing					
RUE motor					
LUE motor					
RLE motor					
LLE motor					
RUE sensory					
LUE sensory					
RLE sensory					
LLE sensory					
RUE dysmetria					
LUE dysmetria					
RLE dysmetria					

Page | 1 of 2

Stanford Cancer Institute

Patient ID: Click here to enter text.

FORM: Clinical Evaluation of Neurologic Status-DIPG (pontine DMG)

LLE dysmetria					
Gait					
Tandem Gait					
Pseudobulbar affect					
**Total score =**					

Directions for Calculating Score: Sum of all positive and negative points = total score.

Present steroid dose (if none, write "none"):
Baseline steroid dose (if none, write "none"):

Signature (person completing assessment):	Date:
Printed Name:	

Page | 2 of 2

Stanford
Cancer Institute

Patient ID: Click here to enter text.

FORM: Clinical Evaluation of Neurologic Status – Spinal cord DMG

DATE of ASSESSMENT (MM/DD/YYYY): Click here to enter a date. **DAY POST CAR INFUSION:** ______
Time of Assessment: : ☐ am ☐ pm
Name of Person Conducting the Assessment: ______

Instructions: Document deficits in "Details" column at baseline. Use Baseline Form as reference for completing the following table at the protocol specified timepoints. Assign 1 point for symptoms/signs that are improved from baseline, and -1 points for symptoms/signs that are worse than baseline. This form should be used for subjects who are not on an increased dose of steroids.

	Worse (-1)	Same (0)	Better (+1)	No deficit at baseline (0)	details
RUE motor					
LUE motor					
RLE motor					
LLE motor					
RUE sensory					
LUE sensory					
RLE sensory					
LLE sensory					
Trunk sensory					
Bowel function					
Bladder function					
**Total score =**					

Directions for Calculating Score: Sum of all positive and negative points = total score.

Present steroid dose (if none, write "none"):
Baseline steroid dose (if none, write "none"):

Signature (person completing assessment):	Date:
Printed Name:	

13.9 Appendix I: Intracerebroventricular Catheter (ICV) Administration via Ommaya Reservoir (Adult/Peds)

(Adapted from Stanford Standardized Procedure: Intraventricular Chemotherapy Via Ommay Reservoir (Adult, Peds)

Definition:

The administration of cell therapy via Ommaya Reservoir into cerebrospinal fluid (CSF) for treatment of previously diagnosed central nervous system (CNS) involvement by malignancy.

Background Information:

The necessity principal investigator, supervising physician or his/her designee. Designee is defined as another attending physician who works directly with the investigator(s) or supervising physician.

Precautions/Contraindications:

1. Evidence of increased intracranial pressure: increased blood pressure with widening pulse pressure, papilledema, bulging Ommaya or significant decrease in the level of consciousness until imaging studies have ruled out mass effect.
2. Focal neurological findings and/or lesions or imaging studies with significant mass effect.
3. Cutaneous infection at the site of puncture.

Materials:

1. GD2CART in preservative free normal saline in sterile syringe
2. Standard LP tray
3. Sterile gloves
4. Povidone iodine solution
5. 23 gauge needle and stopcock.

Intraventricular Cell Administration via Ommaya Reservoir Procedure

A. Pre-treatment evaluation:
   1. Complete neurologic examination, including focal neurologic and mental status examination, and verify that the Ommaya reservoir is intact with no evidence of erythema or swelling.
   2. Vital signs, with evaluation for fever.
   3. Evaluate for evidence of increased intracranial pressure: high blood pressure, widening pulse pressure, papilledema, decreased level of consciousness, and bulging of Ommaya.
   4. Evaluate for evidence of localized infection or metabolic abnormalities.

B. Procedure:
   1. Review the purpose, risks and benefits, and steps of the procedure with the patient or appropriate legal designee. Ensure ICV consent for additional infusions has been signed.
   2. Obtain GD2CART from BMT-CTF.
   3. Assemble supplies.
   4. Compress Ommaya bulb twice to re-inflate with fresh CSF for analysis

5. Set up the LP tray.
6. Don sterile gloves.
7. Using sponge applicators from LP tray, scrub skin over reservoir vigorously three times with povidone iodine solution – allow to dry 2 minutes.
8. Drape the patient.
9. A 23 gauge butterfly needle is inserted directly into reservoir at a perpendicular angle to skin, and a volume of cerebrospinal fluid equal to GD2CART drug volume (usually 2 – 6 ml) is removed. Withdraw CSF slowly over approximately 1 minute.
10. Attach chemotherapy syringe and stopcock and inject GD2CART over approximately 3-5 minutes.
11. Turn stopcock, remove syringe. Attach syringe containing preservative free normal saline and flush needle and tubing with approximately 1 ml preservative free saline.
12. Remove needle and cleanse skin with saline and apply spot bandage to site.
13. Place CSF samples in each of the correlative sample specimen tubes and send to appropriate labs for analysis.

Post-Procedure:

1. Assess patient for possible side-effects.
2. Document pretreatment evaluation, procedure, including type and size of needle, patient response, characteristics of CSF, cell product administered, amount of CSF withdrawn, what tests ordered on specimens, patient follow-up instructions, including anti-emetics, if necessary, as well as any complications

14 REFERENCES

1. Louis, D.N., et al., *The 2016 World Health Organization Classification of Tumors of the Central Nervous System: a summary.* Acta Neuropathol, 2016. **131**(6): p. 803-20.

2. Khuong-Quang, D.A., et al., *K27M mutation in histone H3.3 defines clinically and biologically distinct subgroups of pediatric diffuse intrinsic pontine gliomas.* Acta Neuropathol, 2012. **124**(3): p. 439-47.

3. Wu, G., et al., *Somatic histone H3 alterations in pediatric diffuse intrinsic pontine gliomas and non-brainstem glioblastomas.* Nat Genet, 2012. **44**(3): p. 251-3.

4. Castel, D., et al., *Histone H3F3A and HIST1H3B K27M mutations define two subgroups of diffuse intrinsic pontine gliomas with different prognosis and phenotypes.* Acta Neuropathol, 2015. **130**(6): p. 815-27.

5. Warren, K.E., *Diffuse intrinsic pontine glioma: poised for progress.* Front Oncol, 2012. **2**: p. 205.

6. Ostrom, Q.T., et al., *CBTRUS Statistical Report: Primary brain and other central nervous system tumors diagnosed in the United States in 2010-2014.* Neuro Oncol, 2017. **19**(suppl_5): p. v1-v88.

7. Cooney, T., et al., *Contemporary survival endpoints: an International Diffuse Intrinsic Pontine Glioma Registry study.* Neuro Oncol, 2017. **19**(9): p. 1279-1280.

8. Fisher, P.G., et al., *A clinicopathologic reappraisal of brain stem tumor classification. Identification of pilocystic astrocytoma and fibrillary astrocytoma as distinct entities.* Cancer, 2000. **89**(7): p. 1569-76.

9. Bouffet, E., et al., *Radiotherapy followed by high dose busulfan and thiotepa: a prospective assessment of high dose chemotherapy in children with diffuse pontine gliomas.* Cancer, 2000. **88**(3): p. 685-92.

10. Cohen, K.J., et al., *Temozolomide in the treatment of children with newly diagnosed diffuse intrinsic pontine gliomas: a report from the Children's Oncology Group.* Neuro Oncol, 2011. **13**(4): p. 410-6.

11. Dunkel, I.J., B. O'Malley, and J.L. Finlay, *Is there a role for high-dose chemotherapy with stem cell rescue for brain stem tumors of childhood?* Pediatr Neurosurg, 1996. **24**(5): p. 263-6.

12. Finlay, J.L., et al., *High-dose multi-agent chemotherapy followed by bone marrow 'rescue' for malignant astrocytomas of childhood and adolescence.* J Neurooncol, 1990. **9**(3): p. 239-48.

13. Hargrave, D., U. Bartels, and E. Bouffet, *Diffuse brainstem glioma in children: critical review of clinical trials.* Lancet Oncol, 2006. **7**(3): p. 241-8.

14. Jalali, R., et al., *Prospective evaluation of radiotherapy with concurrent and adjuvant temozolomide in children with newly diagnosed diffuse intrinsic pontine glioma.* Int J Radiat Oncol Biol Phys, 2010. **77**(1): p. 113-8.

15. Jansen, M.H., et al., *Diffuse intrinsic pontine gliomas: a systematic update on clinical trials and biology.* Cancer Treat Rev, 2012. **38**(1): p. 27-35.

16. Jennings, M.T., et al., *Preradiation chemotherapy in primary high-risk brainstem tumors: phase II study CCG-9941 of the Children's Cancer Group.* J Clin Oncol, 2002. **20**(16): p. 3431-7.

17. Maude, S.L., *Tisagenlecleucel in pediatric patients with acute lymphoblastic leukemia.* Clin Adv Hematol Oncol, 2018. **16**(10): p. 664-666.

18. Neelapu, S.S., Frederick L. Locke, Nancy L. Bartlett, Lazaros Lekakis, David Miklos, Caron A. Jacobson, Ira Braunschweig, Olalekan Oluwole, Tanya Siddiqi, Yi Lin, John Timmerman, Patrick J. Stiff, Jonathan Friedberg, Ian Flinn, Andre Goy, Mitchell Smith, Abhinav Deol, Umar Farooq, Peter McSweeney, Javier Munoz, Irit Avivi, Januario E. Castro, Jason R. Westin, Julio C. Chavez, Armin Ghobadi, Krishna V. Komanduri, Ronald Levy, Eric D. Jacobsen, Patrick Reagan, Adrian Bot, John M. Rossi, Lynn Navale, Yizhou Jiang, Jeff S. Aycock, Meg Elias, Jeff Wiezorek and William Y. Go, *Kte-C19 (anti-CD19 CAR T Cells) Induces Complete Remissions in Patients with Refractory Diffuse Large B-Cell Lymphoma (DLBCL): Results from the Pivotal Phase 2 Zuma-1*, in *Blood*. 2016, American Society of Hematology: San Diego, CA.

19. Yu, A.L., et al., *Anti-GD2 antibody with GM-CSF, interleukin-2, and isotretinoin for neuroblastoma.* N Engl J Med, 2010. **363**(14): p. 1324-34.

20. Mody, R., et al., *Irinotecan-temozolomide with temsirolimus or dinutuximab in children with refractory or relapsed neuroblastoma (COG ANBL1221): an open-label, randomised, phase 2 trial.* Lancet Oncol, 2017. **18**(7): p. 946-957.

21. Mount, C.W., et al., *Potent antitumor efficacy of anti-GD2 CAR T cells in H3-K27M(+) diffuse midline gliomas.* Nat Med, 2018. **24**(5): p. 572-579.

22. Gubin, M.M., et al., *Tumor neoantigens: building a framework for personalized cancer immunotherapy.* J Clin Invest, 2015. **125**(9): p. 3413-21.

23. Rizvi, N.A., et al., *Cancer immunology. Mutational landscape determines sensitivity to PD-1 blockade in non-small cell lung cancer.* Science, 2015. **348**(6230): p. 124-8.

24. Merchant, M.S., et al., *Phase I Clinical Trial of Ipilimumab in Pediatric Patients with Advanced Solid Tumors.* Clin Cancer Res, 2016. **22**(6): p. 1364-70.

25. Davis, R.J., et al., *Anti-PD-L1 Efficacy Can Be Enhanced by Inhibition of Myeloid-Derived Suppressor Cells with a Selective Inhibitor of PI3Kdelta/gamma.* Cancer Res, 2017. **77**(10): p. 2607-2619.

26. Pugh, T.J., et al., *The genetic landscape of high-risk neuroblastoma.* Nat Genet, 2013. **45**(3): p. 279-84.

27. Grasso, C.S., et al., *Functionally defined therapeutic targets in diffuse intrinsic pontine glioma.* Nat Med, 2015. **21**(7): p. 827.

28. Mackay, A., et al., *Integrated Molecular Meta-Analysis of 1,000 Pediatric High-Grade and Diffuse Intrinsic Pontine Glioma.* Cancer Cell, 2017. **32**(4): p. 520-537 e5.

29. Lee, D.W., et al., *T cells expressing CD19 chimeric antigen receptors for acute lymphoblastic leukaemia in children and young adults: a phase 1 dose-escalation trial.* Lancet, 2015. **385**(9967): p. 517-28.

30. Long, A.H., et al., *4-1BB costimulation ameliorates T cell exhaustion induced by tonic signaling of chimeric antigen receptors.* Nat Med, 2015. **21**(6): p. 581-90.

31. Monje, M. and P.G. Fisher, *Neurological complications following treatment of children with brain tumors.* J Pediatr Rehabil Med, 2011. **4**(1): p. 31-6.

32. Weng, J., et al., *IL-15 enhances the antitumor effect of human antigen-specific CD8+ T cells by cellular senescence delay.* Oncoimmunology, 2016. **5**(12): p. e1237327.

33. Pule, M.A., et al., *Virus-specific T cells engineered to coexpress tumor-specific receptors: persistence and antitumor activity in individuals with neuroblastoma.* Nat Med, 2008. **14**(11): p. 1264-70.

34. Louis, C.U., et al., *Antitumor activity and long-term fate of chimeric antigen receptor-positive T cells in patients with neuroblastoma.* Blood, 2011. **118**(23): p. 6050-6.

35. Locatelli, F., *Phase 1 trial with GD2-directed CAR T cell therapy in neuroblastoma patients*, in *EHA-EBMT 2nd European CAR T Cell Meeting*. 2020: Barcelona, Spain.

36. Panditharatna, E., et al., *Clinically Relevant and Minimally Invasive Tumor Surveillance of Pediatric Diffuse Midline Gliomas Using Patient-Derived Liquid Biopsy.* Clin Cancer Res, 2018. **24**(23): p. 5850-5859.

37. Freeman, C.R. and J.P. Farmer, *Pediatric brain stem gliomas: a review.* Int J Radiat Oncol Biol Phys, 1998. **40**(2): p. 265-71.

38. Ostrom, Q.T., et al., *Epidemiology of Intracranial Gliomas.* Prog Neurol Surg, 2018. **30**: p. 1-11.

39. Cohen, K.J., A. Broniscer, and J. Glod, *Pediatric glial tumors.* Curr Treat Options Oncol, 2001. **2**(6): p. 529-36.

40. Langmoen, I.A., et al., *Management of pediatric pontine gliomas.* Childs Nerv Syst, 1991. **7**(1): p. 13-5.

41. Schwartzentruber, J., et al., *Driver mutations in histone H3.3 and chromatin remodelling genes in paediatric glioblastoma.* Nature, 2012. **482**(7384): p. 226-31.

42. Qin, E.Y., et al., *Neural Precursor-Derived Pleiotrophin Mediates Subventricular Zone Invasion by Glioma.* Cell, 2017. **170**(5): p. 845-859 e19.

43. Ali, N., et al., *Xenogeneic graft-versus-host-disease in NOD-scid IL-2Rgammanull mice display a T-effector memory phenotype.* PLoS One, 2012. **7**(8): p. e44219.

44. Nagaraja, S., et al., *Transcriptional Dependencies in Diffuse Intrinsic Pontine Glioma.* Cancer Cell, 2017. **31**(5): p. 635-652 e6.

45. Louveau, A., et al., *Structural and functional features of central nervous system lymphatic vessels.* Nature, 2015. **523**(7560): p. 337-41.

46. Till, B.G., et al., *Adoptive immunotherapy for indolent non-Hodgkin lymphoma and mantle cell lymphoma using genetically modified autologous CD20-specific T cells.* Blood, 2008. **112**(6): p. 2261-71.

47. Di Stasi, A., et al., *Inducible apoptosis as a safety switch for adoptive cell therapy.* N Engl J Med, 2011. **365**(18): p. 1673-83.

48. Heczey, A., et al., *CAR T Cells Administered in Combination with Lymphodepletion and PD-1 Inhibition to Patients with Neuroblastoma.* Mol Ther, 2017. **25**(9): p. 2214-2224.

49. D'Angelo, S.P., et al., *Antitumor Activity Associated with Prolonged Persistence of Adoptively Transferred NY-ESO-1 (c259)T Cells in Synovial Sarcoma.* Cancer Discov, 2018. **8**(8): p. 944-957.

50. Weber, E.W., et al., *Pharmacologic control of CAR-T cell function using dasatinib.* Blood Adv, 2019. **3**(5): p. 711-717.

51. Schade, A.E., et al., *Dasatinib, a small-molecule protein tyrosine kinase inhibitor, inhibits T-cell activation and proliferation.* Blood, 2008. **111**(3): p. 1366-77.

52. Lee, D.W., et al., *ASTCT Consensus Grading for Cytokine Release Syndrome and Neurologic Toxicity Associated with Immune Effector Cells.* Biol Blood Marrow Transplant, 2019. **25**(4): p. 625-638.

53. Davila, M.L., et al., *Efficacy and toxicity management of 19-28z CAR T cell therapy in B cell acute lymphoblastic leukemia.* Sci Transl Med, 2014. **6**(224): p. 224ra25.

54. Lee, D.W., et al., *Current concepts in the diagnosis and management of cytokine release syndrome.* Blood, 2014. **124**(2): p. 188-95.

55. Maude, S.L., et al., *Chimeric antigen receptor T cells for sustained remissions in leukemia.* N Engl J Med, 2014. **371**(16): p. 1507-17.

56. Weber, J., et al., *A phase I trial of intravenous interleukin-6 in patients with advanced cancer.* J Immunother Emphasis Tumor Immunol, 1994. **15**(4): p. 292-302.

57. Taraseviciute, A., et al., *Chimeric Antigen Receptor T Cell-Mediated Neurotoxicity in Nonhuman Primates.* Cancer Discov, 2018. **8**(6): p. 750-763.

58. Saleh, M.N., et al., *Phase I trial of the chimeric anti-GD2 monoclonal antibody ch14.18 in patients with malignant melanoma.* Hum Antibodies Hybridomas, 1992. **3**(1): p. 19-24.

59. Murray, J.L., et al., *Phase I trial of murine monoclonal antibody 14G2a administered by prolonged intravenous infusion in patients with neuroectodermal tumors.* J Clin Oncol, 1994. **12**(1): p. 184-93.

60. Richman, S.A., et al., *High-Affinity GD2-Specific CAR T Cells Induce Fatal Encephalitis in a Preclinical Neuroblastoma Model.* Cancer Immunol Res, 2018. **6**(1): p. 36-46.

61. Majzner, R.G., et al., *Neurotoxicity Associated with a High-Affinity GD2 CAR-Letter.* Cancer Immunol Res, 2018. **6**(4): p. 494-495.

62. Lynn, R.C., et al., *c-Jun overexpression in CAR T cells induces exhaustion resistance.* Nature, 2019. **576**(7786): p. 293-300.

63. Hoseini, S.S., et al., *Bispecific antibody does not induce T-cell death mediated by chimeric antigen receptor against disialoganglioside GD2.* Oncoimmunology, 2017. **6**(6): p. e1320625.

64. Richards, R.M., E. Sotillo, and R.G. Majzner, *CAR T Cell Therapy for Neuroblastoma.* Frontiers in Immunology, 2018. **9**(2380).

65. Rajasekaran, S., et al., *Therapeutic role of anakinra, an interleukin-1 receptor antagonist, in the management of secondary hemophagocytic lymphohistiocytosis/sepsis/multiple organ dysfunction/macrophage activating syndrome in critically ill children*.* Pediatr Crit Care Med, 2014. **15**(5): p. 401-8.

66. Shakoory, B., et al., *Interleukin-1 Receptor Blockade Is Associated With Reduced Mortality in Sepsis Patients With Features of Macrophage Activation Syndrome: Reanalysis of a Prior Phase III Trial.* Crit Care Med, 2016. **44**(2): p. 275-81.

67. Traube, C., et al., *Cornell Assessment of Pediatric Delirium: a valid, rapid, observational tool for screening delirium in the PICU*.* Crit Care Med, 2014. **42**(3): p. 656-63.

68. Silver, G., et al., *Delirium screening anchored in child development: The Cornell Assessment for Pediatric Delirium.* Palliat Support Care, 2015. **13**(4): p. 1005-11.
