# Supplementary material for: GD2-CAR T cell therapy for H3K27M-mutated diffuse midline gliomas
Source: Nature. 2022 Feb 7;603(7903):934–41. doi: 10.1038/s41586-022-04489-4 (PMC8967714; doi:10.1038/s41586-022-04489-4)
Supplement: Supplementary file 2 — Reporting Summary [file 41586_2022_4489_MOESM2_ESM.pdf]

nature portfolio

Corresponding author(s): Michelle Monje and Crystal Mackall

Last updated by author(s): Dec 2, 2021

Reporting Summary

Nature Portfolio wishes to improve the reproducibility of the work that we publish. This form provides structure for consistency and transparency in reporting. For further information on Nature Portfolio policies, see our Editorial Policies and the Editorial Policy Checklist.

Statistics

For all statistical analyses, confirm that the following items are present in the figure legend, table legend, main text, or Methods section.

n/a	Confirmed	
☐	☒	The exact sample size (*n*) for each experimental group/condition, given as a discrete number and unit of measurement
☐	☒	A statement on whether measurements were taken from distinct samples or whether the same sample was measured repeatedly
☐	☒	The statistical test(s) used AND whether they are one- or two-sided<br>*Only common tests should be described solely by name; describe more complex techniques in the Methods section.*
☐	☒	A description of all covariates tested
☒	☐	A description of any assumptions or corrections, such as tests of normality and adjustment for multiple comparisons
☒	☐	A full description of the statistical parameters including central tendency (e.g. means) or other basic estimates (e.g. regression coefficient) AND variation (e.g. standard deviation) or associated estimates of uncertainty (e.g. confidence intervals)
☒	☐	For null hypothesis testing, the test statistic (e.g. *F*, *t*, *r*) with confidence intervals, effect sizes, degrees of freedom and *P* value noted<br>*Give P values as exact values whenever suitable.*
☒	☐	For Bayesian analysis, information on the choice of priors and Markov chain Monte Carlo settings
☒	☐	For hierarchical and complex designs, identification of the appropriate level for tests and full reporting of outcomes
☒	☐	Estimates of effect sizes (e.g. Cohen's *d*, Pearson's *r*), indicating how they were calculated

Our web collection on statistics for biologists contains articles on many of the points above.

Software and code

Policy information about availability of computer code

Data collection	N/A
Data analysis	Analysis of single cell RNA sequencing data was performed with referenced custom R scripts. Data analysis was performed in Graphpad Prism and Microsoft Excel.

For manuscripts utilizing custom algorithms or software that are central to the research but not yet described in published literature, software must be made available to editors and reviewers. We strongly encourage code deposition in a community repository (e.g. GitHub). See the Nature Portfolio guidelines for submitting code & software for further information.

Data

Policy information about availability of data

All manuscripts must include a data availability statement. This statement should provide the following information, where applicable:

- Accession codes, unique identifiers, or web links for publicly available datasets
- A description of any restrictions on data availability
- For clinical datasets or third party data, please ensure that the statement adheres to our policy

All raw data are provided in the source data file. The single cell RNA-sequencing data has been uploaded to GEO (GSE186802).

Field-specific reporting

Please select the one below that is the best fit for your research. If you are not sure, read the appropriate sections before making your selection.

☒ Life sciences ☐ Behavioural & social sciences ☐ Ecological, evolutionary & environmental sciences

For a reference copy of the document with all sections, see nature.com/documents/nr-reporting-summary-flat.pdf

Life sciences study design

All studies must disclose on these points even when the disclosure is negative.

Sample size	Four patients were enrolled on the first dose level of this clinical trial.
Data exclusions	No data was excluded from the analysis.
Replication	ctDNA, and RT-PCR for CAR transgene were performed in triplicate, Cytokine analyses were performed in duplicate.
Randomization	No randomization was performed in this Phase 1 clinical trial.
Blinding	No blinding was performed in this Phase 1 clinical trial.

Reporting for specific materials, systems and methods

We require information from authors about some types of materials, experimental systems and methods used in many studies. Here, indicate whether each material, system or method listed is relevant to your study. If you are not sure if a list item applies to your research, read the appropriate section before selecting a response.

Materials & experimental systems

n/a	Involved in the study	
☐	☒	Antibodies
☒	☐	Eukaryotic cell lines
☒	☐	Palaeontology and archaeology
☒	☐	Animals and other organisms
☐	☒	Human research participants
☐	☒	Clinical data
☒	☐	Dual use research of concern

Methods

n/a	Involved in the study	
☒	☐	ChIP-seq
☐	☒	Flow cytometry
☐	☒	MRI-based neuroimaging

Antibodies

Antibodies used

CD3 FITC UCHT1 BioLegend 300406
CD8 PerCP Cy5.5 SK1 BD Pharmingen 565310
CD45 BV785 2D1 BioLegend 368528
CD4 BV711 RPA-T4 BioLegend 300558
CD95 BV650 DX2 BioLegend 305624
CD39 Bv605 A1 BioLegend 328236
Cell viability BV510 N/A Invitrogen L-34965
CD57 BV421 NK-1 BDBiosciences 563896
CCR7 BUV805 2L1A BDBiosciences 749673
CD45RA Alx700 HI100 BioLegend 304120
GD2CAR DyLight650 1A7 Custom
CD14 PE-Cy7 63D3 BioLegend 367112
CD11b APC-Cy7 ICRF44 BioLegend 301352
CD33 PE-Dazzle WM53 BIolegend 303432
GD2 PE 14G2A BioLegend 357304
CD4 BUV395 SK3 BD Biosciences 563550
CD8 BUV795 SK1 BD Biosciences 564912
CD45 PerCP-Cy5.5 HI30 eBioscience 45-0459-41
GD2 BV510 14g2a BioLegend 357316
B7-H3 PE AF R&D FAP1027P
CD14 PE-Cy7 63D3 BioLegend 367112
CD11b APC-Cy7 ICRF44 BioLegend 301352
Cell viability DAPI N/A ThermoFisher Scientific 62247
GD2CAR DyLight650 1A7 Custom

Validation	All antibodies were validated as per the manufacturer except 1A7. In the case of 1A7, the anti-GD2 CAR idiotype antibody, untransduced T cells were used as a biologic control.<br>For RNAscope analysis of the GD2 CAR construct, validation was performed using a positive control (cultured GD2-CAR T-cells) and a negative control (autopsy brain tissue sample from an untreated patient).

Human research participants

Policy information about studies involving human research participants

Population characteristics	Beginning in June 2020, four subjects were enrolled on DL1 (1e6 GD2-CAR T/kg; 3 DIPG, 1 spinal cord DMG; ages 5-25; 1M/3F). Results are reported here with a data cut of March 2021.
Recruitment	Patients on this Phase 1 clinical trial were recruited through physician and self-referral.
Ethics oversight	The Stanford University IRB approved this clinical study.

Note that full information on the approval of the study protocol must also be provided in the manuscript.

Clinical data

Policy information about clinical studies

All manuscripts should comply with the ICMJE guidelines for publication of clinical research and a completed CONSORT checklist must be included with all submissions.

Clinical trial registration	NCT04196413
Study protocol	Study protocol provided with ammendments.
Data collection	June 2020- March 2021
Outcomes	Primary objectives assessed: feasibility of manufacturing, safety and tolerability, and identifying the maximally tolerated dose or recommended phase II dose. Assessment of clinical activity is the secondary objective and identifying correlative biomarkers of response is an exploratory objective.

Flow Cytometry

Plots

Confirm that:

☒ The axis labels state the marker and fluorochrome used (e.g. CD4-FITC).

☒ The axis scales are clearly visible. Include numbers along axes only for bottom left plot of group (a 'group' is an analysis of identical markers).

☒ All plots are contour plots with outliers or pseudocolor plots.

☒ A numerical value for number of cells or percentage (with statistics) is provided.

Methodology

Sample preparation	PBMC were isolated from fresh whole blood by gradient centrifugation on ficoll (Ficoll paque Plus, GE Healthcare, SigmaAldrich). Two to five million PBMC were stained with fixable Live/Dead aqua (Invitrogen) amine-reactive viability stain. Cells were then preincubated with Fc block (trustain, Biolegend) for 5 min, then stained at room temperature with the following fluorochrome conjugated mAb in an 15-color, 17-parameter staining combination.
Instrument	LSR (BD BioSciences)
Software	Analysis was performed in FlowJo
Cell population abundance	At least, 106 cells were acquired unless restricted by the number of cells isolated from 8 ml of whole blood or when acquiring CSF isolated cells. The assay limit of detection for cells calculated as 1 in 104 of total acquired PBMCs.
Gating strategy	Tumor resection material: Viable cells->singlets->cells (FSC/SSC)->CD45-/B7-H3+.<br>GD2 expression compared to FMO control<br><br>CAR T-cells in CSF and PBMC: Singlets->viable cells->CD45+->CD14-, CD3+ -> CD4 or CD8<br>CAR positivity gated based on control PBMC.

☒ Tick this box to confirm that a figure exemplifying the gating strategy is provided in the Supplementary Information.

Magnetic resonance imaging

Experimental design

Design type	Clinical studies
Design specifications	Clinical studies
Behavioral performance measures	NA

Acquisition

Imaging type(s)	MRI brain and spine (clinical protocols)
Field strength	3T
Sequence & imaging parameters	T2 sequences shown
Area of acquisition	Brain and Spine

Diffusion MRI ☐ Used ☒ Not used

Preprocessing

Preprocessing software	NA
Normalization	NA
Normalization template	NA
Noise and artifact removal	NA
Volume censoring	NA

Statistical modeling & inference

Model type and settings	NA
Effect(s) tested	NA

Specify type of analysis: ☒ Whole brain ☐ ROI-based ☐ Both

Statistic type for inference (See Eklund et al. 2016)	NA
Correction	NA

Models & analysis

n/a	Involved in the study	
☒	☐	Functional and/or effective connectivity
☒	☐	Graph analysis
☒	☐	Multivariate modeling or predictive analysis
