# Supplementary material for: GD2-CAR T cell therapy for H3K27M-mutated diffuse midline gliomas
Source: Nature. 2022 Feb 7;603(7903):934–41. doi: 10.1038/s41586-022-04489-4 (PMC8967714; doi:10.1038/s41586-022-04489-4)
Supplement: Supplementary file 4 — This file contains Supplementary Tables 1-10. [file 41586_2022_4489_MOESM4_ESM.docx]

**Supplementary Table 1: Clinical characteristics**

| Subject | Age at Diagnosis | Sex | Tumor Molecular Characteristics | Treatment History | Time from prior treatment to CAR infusion |
| --- | --- | --- | --- | --- | --- |
| Spinal DMG-1 | 24 years | F | H3K27M-mutant (WHO grade IV), negative for H3 K27me3    H3F3A p.K28M, positive for S-100, GFAP, TP53, ATRX, elevated Ki67/MIB-1 index | Spinal radiation to 45 Gy with a boost to 54 Gy with concurrent temozolomide for 3 cycles | 65 days |
| DIPG-1 | 13 years | F | H3K27M-mutant (WHO grade IV), negative for H3 K27me3  H3F3A p.K28M, positive for TP53 p. R276G, KIT amplification, MET amplification, and NBN rearrangement | Focal radiation to brainstem to 54 Gy, followed by panobinostat for 5 cycles | 120 days |
| DIPG-2 | 20 years | M | H3K27M-mutant (WHO grade IV), negative for H3 K27me3  H3F3A p.K28M, positive for TP53 p.R273H, ATRX, RB1 p.E373fs | Focal radiation to brainstem to 60 Gy, followed by ONC- 201 for 11 cycles | 41 days |
| DIPG-3 | 4 years | F | H3K27M-mutant (WHO grade IV), negative for H3 K27me3  H3F3A p.K28M, positive for TP53 p. R248Q, DAXX (NM_001350) rearrangement | Focal radiation to brainstem to 54 Gy | 373 days |

**Supplementary Table 1:** Clinical characteristics of treated patients. DMG: diffuse midline glioma; DIPG: diffuse intrinsic pontine glioma; H3K27M: histone 3 lysine 27 for methionine substation; WHO: world health organization; Gy: gray.

**Supplementary Table 2: Cell Manufacturing**

| Subject | CD4% of CD4/CD8 Enriched | CD8% of CD4/CD8 Enriched | CAR T% | CD4% of product | CD8% of product | VCN (per CAR T) | Days in Culture | TNC Expansion |
| --- | --- | --- | --- | --- | --- | --- | --- | --- |
| Spinal DMG-1 | 53.11 | 24.69 | 63.61 | 81.57 | 8.69 | 4.14 | 7 | 2.07E+09 |
| DIPG-1 | 43.05 | 31.15 | 34.11 | 77.02 | 15.58 | 5.04 | 7 | 1.38E+09 |
| DIPG-2 | 54.3 | 29.02 | 83.8 | 85.21 | 8.24 | 7.2 | 7 | 2.10E+09 |
| DIPG-3 | 55.09 | 29.53 | 73 | 86.21 | 7.56 | 3.34 | 7 | 2.34E+09 |

**Supplementary Table 2:** Quality attributes of manufactured products. TNC: Total Nucleated Cells; VCN: Vector Copy Number

**Supplementary Table 3: Anti-inflammatory interventions**

| Subject | Infusion 1 (IV) | | | | Infusion 2 (ICV) | | | |
| --- | --- | --- | --- | --- | --- | --- | --- | --- |
|  | Tocilizumab | Dexamethasone | Methylprednisolone | Anakinra | Dexamethasone | Anakinra | Dasatinib | Siltuximab |
| Spinal DMG-1 | D8 |  | D8-11 |  | D2-24 | D1-19 | D2-13 | D5 |
| DIPG-1 | D7 | D7-87 | D9-15 | D9-15 |  | | | |
| DIPG- 2 | D8 |  |  | D8-14 | D2-6 | D2-8 |  |  |
| DIPG-3 | D8 |  |  | D8-13 |  |  |  |  |

**Supplementary Table 3:** Description of anti-inflammatory agents used in clinical management of patients. D = day post-infusion.

**Supplementary Table 4: Clinical Improvement Score**

| Subject | Infusion 1 (Intravenous) | | | Infusion 2 (Intracerebroventricular) | |
| --- | --- | --- | --- | --- | --- |
|  | Month 1 | Month 2 | Month 3 | Week 2 | Week 4 |
| Spinal DMG-1 | 0 | +2 | 0 | * | 0 |
| DIPG-1 | * | | |  |  |
| DIPG-2 | +4 | +2 | -1 | +10 | +9 |
| DIPG-3 | 0 | +8 | +8 | +3 | 0 |

*Could not assess due to steroid use

**Supplementary Table 4:** Changes in neurological symptoms and signs from pre-infusion baseline were assessed using a “clinical improvement score” to quantify changes in the neurological exam (Please see Extended Data Tables 6 and 7 for blank examples). (Baseline was determined by neurological exam prior to each infusion (IV and ICV). The clinical improvement scale can only be assessed if the subject is off steroids for at least 7 days; timepoints during which clinical change could not be assessed due to recent steroid use are denoted by an asterisk*. DMG: diffuse midline glioma; DIPG: diffuse intrinsic pontine glioma.

**Supplementary Table 5: Unique Myeloid Cluster Gene Expression Profile**

**Upregulated**

| Number | Gene | p_val | avg_log2FC | pct.1 | pct.2 | p_val_adj |
| --- | --- | --- | --- | --- | --- | --- |
| 1 | TNFAIP6 | 3.10E-15 | 11.286358 | 0.45 | 0.012 | 6.19E-12 |
| 2 | SOWAHC | 4.20E-07 | 10.77336463 | 0.55 | 0.017 | 0.000840592 |
| 3 | CCL8 | 2.64E-07 | 9.791419482 | 0.3 | 0.052 | 0.000527945 |
| 4 | KIAA1257 | 1.04E-06 | 8.889234398 | 0.65 | 0.075 | 0.002071299 |
| 5 | SECTM1 | 5.12E-14 | 8.635789368 | 0.65 | 0.035 | 1.02E-10 |
| 6 | AQP9 | 5.45E-07 | 8.188441725 | 0.8 | 0.249 | 0.001089514 |
| 7 | SPHK1 | 2.21E-11 | 5.8032713 | 0.55 | 0.11 | 4.41E-08 |
| 8 | OLFM1 | 1.54E-15 | 5.043282157 | 0.95 | 0.081 | 3.08E-12 |
| 9 | ATF5 | 7.46E-07 | 4.763354638 | 0.8 | 0.318 | 0.001492543 |
| 10 | AREG | 6.63E-11 | 4.667589457 | 0.3 | 0.087 | 1.33E-07 |
| 11 | RETN | 5.96E-11 | 4.554428072 | 0.85 | 0.127 | 1.19E-07 |
| 12 | FLT1 | 5.73E-11 | 3.626104938 | 0.85 | 0.092 | 1.15E-07 |
| 13 | MIR222HG | 1.85E-09 | 3.475769799 | 0.65 | 0.023 | 3.70E-06 |
| 14 | CXCL2 | 6.55E-13 | 3.416145171 | 1 | 0.173 | 1.31E-09 |
| 15 | G0S2 | 2.65E-06 | 3.395995497 | 0.7 | 0.173 | 0.005307888 |
| 16 | THBD | 5.80E-06 | 3.104082728 | 0.8 | 0.092 | 0.011598805 |
| 17 | VCAN | 2.19E-07 | 2.982992029 | 0.8 | 0.121 | 0.000437658 |
| 18 | MPEG1 | 3.12E-07 | 2.454664864 | 0.85 | 0.127 | 0.000624189 |
| 19 | LYZ | 1.15E-07 | 2.398663654 | 0.9 | 0.618 | 0.000230262 |
| 20 | BCL11A | 9.11E-10 | 2.391376169 | 0.9 | 0.185 | 1.82E-06 |
| 21 | AP1S2 | 2.17E-06 | 2.097932038 | 0.85 | 0.509 | 0.00433856 |

**Downregulated**

| Number | Gene | p_val | avg_log2FC | pct.1 | pct.2 | p_val_adj |
| --- | --- | --- | --- | --- | --- | --- |
| 1 | AFF3 | 1.36E-10 | -11.70819361 | 0.5 | 0.035 | 2.72E-07 |
| 2 | FLT3 | 3.17E-09 | -10.33837244 | 0.75 | 0.023 | 6.35E-06 |
| 3 | GDF15 | 5.26E-08 | -10.33348199 | 0.85 | 0.197 | 0.000105138 |
| 4 | CRIP2 | 7.38E-12 | -10.02341217 | 0.85 | 0.104 | 1.48E-08 |
| 5 | RARRES1 | 4.55E-06 | -9.959611947 | 0.65 | 0.168 | 0.009105388 |
| 6 | SDS | 2.05E-06 | -9.726648435 | 0.75 | 0.214 | 0.004091248 |
| 7 | TPPP3 | 3.09E-11 | -9.407251189 | 0.95 | 0.104 | 6.18E-08 |
| 8 | SERPINE1 | 4.53E-06 | -9.364241667 | 0.85 | 0.162 | 0.009062112 |
| 9 | CLLU1OS | 1.76E-11 | -9.240999137 | 0.75 | 0.208 | 3.52E-08 |
| 10 | DHRS9 | 6.27E-07 | -9.089360631 | 0.8 | 0.197 | 0.001253207 |
| 11 | AC011611.3 | 7.90E-16 | -9.062776128 | 0.95 | 0.081 | 1.58E-12 |
| 12 | IGFBP6 | 2.59E-14 | -9.038620149 | 0.75 | 0.075 | 5.19E-11 |
| 13 | CD1E | 4.86E-10 | -9.010083876 | 0.5 | 0.092 | 9.71E-07 |
| 14 | SCG5 | 4.39E-12 | -9.002312392 | 0.3 | 0.075 | 8.77E-09 |
| 15 | CD1A | 9.85E-12 | -8.980560869 | 0.65 | 0.04 | 1.97E-08 |
| 16 | CCL18 | 4.71E-12 | -8.93092064 | 0.65 | 0.098 | 9.42E-09 |
| 17 | COL4A2-AS2 | 2.36E-09 | -8.930630464 | 0.7 | 0.156 | 4.73E-06 |
| 18 | AC131944.1 | 6.54E-13 | -8.929620665 | 1 | 0.139 | 1.31E-09 |
| 19 | CCL23 | 9.57E-08 | -8.720934999 | 0.85 | 0.156 | 0.000191357 |
| 20 | OCSTAMP | 4.66E-13 | -8.585042624 | 0.35 | 0.081 | 9.31E-10 |
| 21 | COL6A1 | 1.20E-11 | -8.579775691 | 0.7 | 0.098 | 2.39E-08 |
| 22 | AC003093.1 | 1.07E-08 | -8.57813702 | 0.35 | 0.133 | 2.14E-05 |
| 23 | LINC01235 | 4.67E-09 | -8.528507074 | 0.35 | 0.162 | 9.34E-06 |
| 24 | MT1H | 5.93E-16 | -8.493062202 | 0.6 | 0.064 | 1.19E-12 |
| 25 | CCL3L1 | 1.09E-10 | -8.447347558 | 0.8 | 0.127 | 2.17E-07 |
| 26 | ENHO | 6.12E-11 | -8.426976643 | 0.8 | 0.064 | 1.22E-07 |
| 27 | AC092484.1 | 2.17E-13 | -8.312749818 | 0.85 | 0.058 | 4.33E-10 |
| 28 | CRABP2 | 8.54E-14 | -8.303814306 | 0.75 | 0.075 | 1.71E-10 |
| 29 | AL139351.2 | 6.54E-16 | -8.093066598 | 0.7 | 0.046 | 1.31E-12 |
| 30 | ANGPTL4 | 3.04E-06 | -7.998692576 | 0.5 | 0.127 | 0.006086225 |
| 31 | L1CAM | 2.49E-11 | -7.979225409 | 0.5 | 0.029 | 4.98E-08 |
| 32 | MT3 | 4.02E-14 | -7.967419454 | 0.55 | 0.069 | 8.04E-11 |
| 33 | BACE2 | 1.41E-11 | -7.913108074 | 0.85 | 0.15 | 2.82E-08 |
| 34 | HSPA1B | 2.06E-06 | -7.647695938 | 0.3 | 0.208 | 0.004114749 |
| 35 | MYL9 | 1.16E-13 | -7.595280924 | 1 | 0.156 | 2.32E-10 |
| 36 | FCGBP | 1.18E-06 | -7.505294721 | 0.75 | 0.214 | 0.002360512 |
| 37 | INHBA | 1.19E-07 | -7.39501866 | 0.65 | 0.121 | 0.000238472 |
| 38 | SLC16A10 | 8.11E-11 | -7.368586114 | 0.65 | 0.098 | 1.62E-07 |
| 39 | AK1 | 5.09E-11 | -7.158251728 | 0.8 | 0.116 | 1.02E-07 |
| 40 | FOSB | 1.75E-14 | -7.030836555 | 0.9 | 0.069 | 3.49E-11 |
| 41 | ERBB3 | 1.40E-18 | -7.022760344 | 0.25 | 0.006 | 2.80E-15 |
| 42 | TPSB2 | 1.25E-15 | -7.006541234 | 0.3 | 0.012 | 2.49E-12 |
| 43 | TRGV4 | 2.12E-18 | -6.997236463 | 0.5 | 0.006 | 4.25E-15 |
| 44 | LINC00167 | 6.58E-17 | -6.98457933 | 0.55 | 0.023 | 1.32E-13 |
| 45 | AC007906.2 | 1.01E-07 | -6.984245175 | 0.5 | 0.191 | 0.000201454 |
| 46 | GHRLOS | 2.43E-10 | -6.966684857 | 0.55 | 0.121 | 4.85E-07 |
| 47 | ITGB8 | 1.17E-06 | -6.9651544 | 0.75 | 0.058 | 0.002330914 |
| 48 | CLEC4C | 1.86E-18 | -6.964636417 | 0.3 | 0.017 | 3.73E-15 |
| 49 | MAG | 8.70E-11 | -6.963917799 | 0.9 | 0.15 | 1.74E-07 |
| 50 | FABP4 | 1.07E-13 | -6.963239732 | 0.75 | 0.087 | 2.15E-10 |
| 51 | COL22A1 | 2.27E-10 | -6.960012118 | 0.65 | 0.127 | 4.54E-07 |
| 52 | MMP7 | 2.94E-07 | -6.952093445 | 0.6 | 0.092 | 0.000587851 |
| 53 | AL359979.2 | 1.25E-15 | -6.940470793 | 0.8 | 0.064 | 2.49E-12 |
| 54 | ITGA2B | 4.04E-17 | -6.936710603 | 0.8 | 0.035 | 8.08E-14 |
| 55 | MT1M | 5.55E-16 | -6.922849783 | 0.55 | 0.075 | 1.11E-12 |
| 56 | HCAR3 | 1.27E-06 | -6.922428924 | 0.3 | 0.023 | 0.002543356 |
| 57 | AK5 | 2.86E-08 | -6.920352646 | 0.7 | 0.046 | 5.73E-05 |
| 58 | PRSS57 | 1.17E-16 | -6.905334251 | 1 | 0.064 | 2.33E-13 |
| 59 | EGR2 | 7.13E-11 | -6.843165198 | 0.5 | 0.116 | 1.43E-07 |
| 60 | TIMP4 | 1.12E-13 | -6.841860425 | 0.65 | 0.046 | 2.23E-10 |
| 61 | FP671120.7 | 8.05E-10 | -6.833651957 | 0.8 | 0.046 | 1.61E-06 |
| 62 | F3 | 3.15E-18 | -6.799243163 | 1 | 0.023 | 6.30E-15 |
| 63 | LINC00996 | 2.59E-17 | -6.789700926 | 0.3 | 0.012 | 5.17E-14 |
| 64 | CES1 | 5.86E-08 | -6.753873639 | 0.95 | 0.208 | 0.000117266 |
| 65 | UCHL1 | 1.05E-15 | -6.743844671 | 0.9 | 0.133 | 2.10E-12 |
| 66 | ITLN1 | 1.41E-15 | -6.683521261 | 1 | 0.092 | 2.83E-12 |
| 67 | S100P | 1.76E-18 | -6.542208085 | 0.85 | 0.012 | 3.53E-15 |
| 68 | FP671120.6 | 4.29E-10 | -6.498411918 | 0.7 | 0.035 | 8.59E-07 |
| 69 | TNFRSF9 | 2.23E-16 | -6.46461001 | 0.75 | 0.035 | 4.45E-13 |
| 70 | TPD52L1 | 1.99E-13 | -6.379441406 | 0.7 | 0.04 | 3.98E-10 |
| 71 | HCAR2 | 3.03E-16 | -5.905497179 | 0.3 | 0.04 | 6.06E-13 |
| 72 | TRIM2 | 7.16E-16 | -5.891018186 | 0.45 | 0.04 | 1.43E-12 |
| 73 | MDK | 1.06E-11 | -5.728816874 | 0.5 | 0.139 | 2.12E-08 |
| 74 | TNFSF9 | 1.08E-11 | -5.709882861 | 0.9 | 0.121 | 2.15E-08 |
| 75 | TLE1 | 7.48E-16 | -5.677904907 | 0.75 | 0.052 | 1.50E-12 |
| 76 | HIST1H2BJ | 1.07E-16 | -5.621666118 | 0.35 | 0.029 | 2.15E-13 |
| 77 | PRG2 | 1.20E-15 | -5.497982049 | 0.4 | 0.017 | 2.39E-12 |
| 78 | TMEM158 | 2.43E-10 | -5.457232897 | 0.35 | 0.104 | 4.85E-07 |
| 79 | AC024909.1 | 1.67E-15 | -5.304105089 | 0.6 | 0.075 | 3.35E-12 |
| 80 | HAMP | 4.21E-07 | -4.706761153 | 0.1 | 0.272 | 0.000842951 |
| 81 | MATK | 9.46E-07 | -3.896034999 | 0.7 | 0.185 | 0.001891791 |
| 82 | TNF | 3.81E-11 | -3.753802016 | 0.25 | 0.029 | 7.61E-08 |
| 83 | AC008982.2 | 1.90E-19 | -3.554226631 | 0.75 | 0.006 | 3.81E-16 |
| 84 | TBC1D4 | 3.17E-06 | -3.355464214 | 0.3 | 0.127 | 0.006338289 |
| 85 | TRGV7 | 1.57E-14 | -3.332103002 | 0.7 | 0.104 | 3.14E-11 |
| 86 | LRG1 | 1.46E-15 | -2.752226991 | 0.85 | 0.104 | 2.92E-12 |
| 87 | CCL7 | 1.11E-13 | -2.606949247 | 0.65 | 0.035 | 2.23E-10 |
| 88 | RCAN1 | 5.63E-13 | -2.416070805 | 0.95 | 0.133 | 1.13E-09 |
| 89 | EGR3 | 2.39E-09 | -2.304390566 | 0.5 | 0.029 | 4.77E-06 |
| 90 | HBEGF | 4.99E-09 | -2.197375448 | 0.85 | 0.173 | 9.99E-06 |
| 91 | AC011603.2 | 9.41E-10 | -2.135122571 | 0.5 | 0.156 | 1.88E-06 |

**Supplementary Table 6: Form for Clinical Evaluation of Neurologic Status, DIPG**

|  | Worse (-1) | Same (0) | Better (+1) | No deficit at baseline (0) | details |
| --- | --- | --- | --- | --- | --- |
| R Third nerve palsy |  |  |  |  |  |
| L Third nerve palsy |  |  |  |  |  |
| R Fourth nerve palsy |  |  |  |  |  |
| L Fourth nerve palsy |  |  |  |  |  |
| R Sixth nerve palsy |  |  |  |  |  |
| L Sixth nerve palsy |  |  |  |  |  |
| R Facial sensation |  |  |  |  |  |
| L Facial sensation |  |  |  |  |  |
| Muscles of mastication (trismus) |  |  |  |  |  |
| R Seventh nerve palsy/facial droop |  |  |  |  |  |
| L Seventh nerve palsy/facial droop |  |  |  |  |  |
| R hearing |  |  |  |  |  |
| L hearing |  |  |  |  |  |
| Palate symmetrical |  |  |  |  |  |
| Speech articulation |  |  |  |  |  |
| Tongue alignment |  |  |  |  |  |
| Swallowing |  |  |  |  |  |
| RUE motor |  |  |  |  |  |
| LUE motor |  |  |  |  |  |
| RLE motor |  |  |  |  |  |
| LLE motor |  |  |  |  |  |
| RUE sensory |  |  |  |  |  |
| LUE sensory |  |  |  |  |  |
| RLE sensory |  |  |  |  |  |
| LLE sensory |  |  |  |  |  |
| RUE dysmetria |  |  |  |  |  |
| LUE dysmetria |  |  |  |  |  |
| RLE dysmetria |  |  |  |  |  |
| LLE dysmetria |  |  |  |  |  |
| Gait |  |  |  |  |  |
| Tandem Gait |  |  |  |  |  |
| Pseudobulbar affect |  |  |  |  |  |
| **Total score =** |  |  |  |  |  |

**Supplementary Table 7: Form for Clinical Evaluation of Neurologic Status, Spinal DMG**

|  | Worse (-1) | Same (0) | Better (+1) | No deficit at baseline (0) | details |
| --- | --- | --- | --- | --- | --- |
| RUE motor |  |  |  |  |  |
| LUE motor |  |  |  |  |  |
| RLE motor |  |  |  |  |  |
| LLE motor |  |  |  |  |  |
| RUE sensory |  |  |  |  |  |
| LUE sensory |  |  |  |  |  |
| RLE sensory |  |  |  |  |  |
| LLE sensory |  |  |  |  |  |
| Trunk sensory |  |  |  |  |  |
| Bowel function |  |  |  |  |  |
| Bladder function |  |  |  |  |  |
| **Total score =** |  |  |  |  |  |

**Supplementary Table 8: qPCR Primer Sequences**

| **qPCR Reagent** | **Sequence** |
| --- | --- |
| Albumin Probe | 5’ - /56-  FAM/CCTGTCATG/ZEN/CCCACACAA ATCTCTCC/3IABkFQ/ - 3’ |
| Albumin Forward Primer | 5’ GCTGTCATCTCTTGTGGGCTGT 3’ |
| Albumin Reverse Primer | 5’ ACTCATGGGAGCTGCTGGTTC 3’ |
| GD2 FAM Probe | 5' /56- FAM/TCATGTTGT/ZEN/AGCCGGTGA AGGAGC/3IABkFQ/ 3' |
| Forward Primer GD2 | 5' CTCTGTGATGATCTCCTGCAA 3' |
| Reverse Primer GD2 | 5' CGATCCATTCCAGGCTCTT 3' |
| GD2 Albumin Minigene® Plasmid (does not include Proprietary backbone sequence) | 5' GCTGGCCTTTTGCTCACAAGCTTGG GGTTGCTGTCATCTCTTGTGGGCTG TAATC ATCGTCTAGGCTTAAGAGTAATATTG CAAAACCTGTCATGCCCACACAAATC TCT CCCTGGCATTGTTGTCTTTGCAGATG TCAGTGAAAGAGAACCAGCAGCTCC CATG AGTCCCAAGCTATGTTCTTTCCTGCG TTTCTCTGGTGGAACCTGGCGCCTC TGTG ATGATCTCCTGCAAGGCCAGCGGCA GCTCCTTCACCGGCTACAACATGAA CTGGG TGCGCCAGAACATCGGCAAGAGCCT GGAATGGATCGGCGCCATCGACCCC TACTA  CGGCGGCACCAGCT |

**Supplementary Table 9: Real-time flow cytometry assay reagents**

| Antigen | Fluorochrome | Clone | Supplier | Catalogue Number |
| --- | --- | --- | --- | --- |
| CD3 | FITC | UCHT1 | BioLegend | 300406 |
| CD8 | PerCP Cy5.5 | SK1 | BD Pharmingen | 565310 |
| CD45 | BV785 | 2D1 | BioLegend | 368528 |
| CD4 | BV711 | RPA-T4 | BioLegend | 300558 |
| CD95 | BV650 | DX2 | BioLegend | 305624 |
| CD39 | Bv605 | A1 | BioLegend | 328236 |
| Cell viability | BV510 | N/A | Invitrogen | L-34965 |
| CD57 | BV421 | NK-1 | BDBiosciences | 563896 |
| CCR7 | BUV805 | 2L1A | BDBiosciences | 749673 |
| CD45RA | Alx700 | HI100 | BioLegend | 304120 |
| GD2CAR | DyLight650 | 1A7 |  | Custom |
| CD14 | PE-Cy7 | 63D3 | BioLegend | 367112 |
| CD11b | APC-Cy7 | ICRF44 | BioLegend | 301352 |
| CD33 | PE-Dazzle | WM53 | BIolegend | 303432 |
| GD2 | PE | 14G2A | BioLegend | 357304 |

**Supplementary Table 10: Reagents for flow cytometry of resected tumor tissue**

| Antigen | Fluorochrome | Clone | Supplier | Catalogue Number |
| --- | --- | --- | --- | --- |
| CD4 | BUV395 | SK3 | BD Biosciences | 563550 |
| CD8 | BUV795 | SK1 | BD Biosciences | 564912 |
| CD45 | PerCP-Cy5.5 | HI30 | eBioscience | 45-0459-41 |
| GD2 | BV510 | 14g2a | BioLegend | 357316 |
| B7-H3 | PE | AF | R&D | FAP1027P |
| CD14 | PE-Cy7 | 63D3 | BioLegend | 367112 |
| CD11b | APC-Cy7 | ICRF44 | BioLegend | 301352 |
| Cell viability | DAPI | N/A | ThermoFisher Scientific | 62247 |
| GD2CAR | DyLight650 | 1A7 | NCI Biological Resources Branch | Custom |
